# Transition-Metal Free Photocatalytic Synthesis of Acylsulfonamides

**DOI:** 10.1021/acs.orglett.5c01129

**Published:** 2025-04-30

**Authors:** Long Yin Lam, Cong Ma

**Affiliations:** State Key Laboratory of Chemical Biology and Drug Discovery, Department of Applied Biology and Chemical Technology, PolyU Marshall Research Centre for Medical Microbial Biotechnology, The Hong Kong Polytechnic University, Kowloon, Hong Kong SAR, China

## Abstract

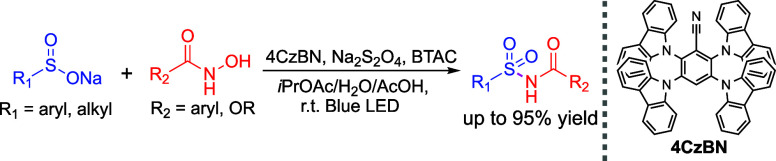

We have developed a transition-metal-free photocatalytic S–N coupling reaction utilizing sodium organosulfinate and hydroxamic acid to synthesize acylsulfonamides. Employing 2,3,5,6-tetra(9H-carbazol-9-yl)benzonitrile (4CzBN) as a photocatalyst, this method enables the preparation of a wide range of acylsulfonamides from arylhydroxamic acids or *N*-hydroxycarbamates. Mechanistic studies indicate that the generation of singlet oxygen (^1^O_2_) via the Energy Transfer Process (EnT) is crucial for facilitating the reaction. This approach offers a sustainable and efficient pathway for acylsulfonamide synthesis under mild conditions.

Acylsulfonamides are fundamental scaffolds in pharmaceutical development due to their broad range of biological activities.^1^ They are widely recognized as bioisosteres of carboxylic acid groups, attributed to their similar acidity and resistance to chemical and enzymatic hydrolysis. In addition to serving as direct drug motifs, acylsulfonamide scaffolds are extensively utilized in solid-phase peptide synthesis^2^ and as acylating agents for amines.^3^ These versatile applications underscore their significance in medicinal chemistry and synthetic methodologies.

The prominent role of acylsulfonamides in medicinal chemistry has spurred extensive research into their synthesis. Traditional methods for synthesizing acylsulfonamides typically involve the acylation of primary sulfonamides using various acylating agents, such as acyl chlorides,^1,4^ acid anhydrides,^5,6^ or carboxylic acids with the assistance of coupling reagents (Scheme 1a).^7−9^ Lewis acids, including TiCl_4_,^10^ Bi(OTf)_3_,^11^ Fe_3_O_4_–diatomite,^12^ and Fe_3_O_4_/SnO nanoparticles,^13^ have been employed as efficient catalysts for the *N*-acylation of sulfonamides. However, the use of acyl chlorides and anhydrides is unfavored and declining due to their susceptibility to hydrolysis and chemoselectivity issues with important pharmaceutical functional groups, such as −OH and −NH_2_, which are prone to nucleophilic substitution.

**Scheme 1:**
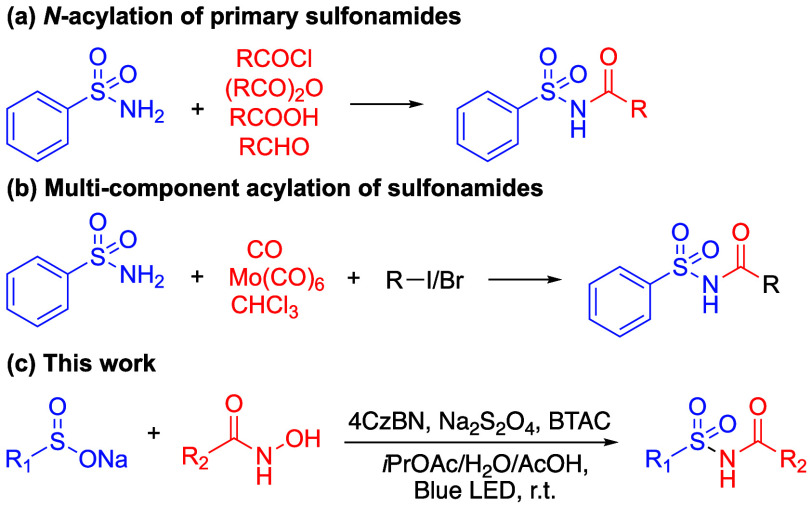
Methods for Preparing Acylsulfonamides

To avoid the use of acyl chlorides and anhydrides, innovative protocols have been developed that utilize aldehydes as efficient acyl surrogates for the *N*-acylation of sulfonamides, facilitated by Rh(II) catalysts^14^ or organocatalysts (Scheme 1a).^15^ Additionally, multicomponent reactions have been explored for the acylation of sulfonamides. By employing carbonyl sources such as CO,^16^ Mo(CO)_6_,^17,18^ or CHCl_3_,^19^ sulfonamides can be coupled with aryl halides to produce acylsulfonamides through a sequential formation of N–C and C–C bonds (Scheme 1b). Despite these advancements, many methods still depend on the nucleophilic substitution of the sulfonamide −NH_2_ group or require catalytic or stoichiometric amounts of transition metals to drive the reaction. Moreover, current synthetic protocols often begin with primary sulfonamides, which limits the diversity of available methods for accessing acylsulfonamides. The preparation of acylsulfonamides via the transfer of a sulfamoyl group has also been reported using Burgess inner salts^20,21^ or chlorosulfonylcarbamates.^22,23^ However, these methods often face challenges due to the need for reactive starting materials or restricted substrate tolerability.

We are interested in expanding the synthetic applications of sodium organosulfinate salts to prepare a variety of pharmaceutically important sulfur-containing scaffolds.^24−27^ In our previous study on the preparation of arylsulfonamides, we observed that benzamide remained intact under persulfate oxidation, highlighting the challenge of directly oxidizing benzamide.^26^ This finding prompted us to explore the synthesis of acylsulfonamides using alternative amide surrogates to circumvent the high activation barrier associated with benzamide. Among the various amide surrogates, we selected hydroxamic acid for investigation due to its higher oxidation state of the nitrogen atom, compared to benzamide and its ease of preparation from carboxylic acids. While hydroxamic acid is often used as a synthon in cycloaddition reactions,^28−30^ its application in other synthetic areas has been underexplored. Herein, we present a new synthetic protocol for the preparation of acylsulfonamides via a photocatalytic reaction with sodium organosulfinates and hydroxamic acid (Scheme 1c).

To begin, sodium *p*-toluenesulfinate (**1a**) and 4-methoxybenzohydroxamic acid (**2a**) were selected as model substrates for the optimization study. Following preliminary screening of reaction conditions (see Tables S1 and S2 in the Supporting Information), the desired acylsulfonamide (**3a**) was obtained in a 38% yield using 1,2,3,5-tetrakis(carbazol-9-yl)4,6-dicyanobenzene (4CzIPN)^31,32^ as the photocatalyst (PC) and Na_2_S_2_O_4_ as the reductant under 10 W blue LED irradiation (Table 1, entry 1). We then evaluated other photocatalysts (Table 1, entries 2–4), but neither acridinium-, Ru- nor Ir-based photocatalysts produced satisfactory results. To further improve the reaction, several cyanoarene-based catalysts were synthesized via S_N_Ar reactions between polyfluorinated cyanoarenes and substituted carbazoles to fine-tune the photocatalyst’s redox properties.^33,34^ Among these, 2,3,5,6-tetra(9H-carbazol-9-yl)benzonitrile (4CzBN) exhibited the highest catalytic activity, achieving a 58% yield with 5.0 equiv of Na_2_S_2_O_4_ (Table 1, entry 6). Given the biphasic nature of the reaction, we anticipated that adding a phase-transfer catalyst (PTC) would enhance mass transfer between phases. Indeed, the addition of benzyl trimethylammonium chloride (BTAC) increased the yield to 75% (Table 1, entry 7). Finally, the addition of acetic acid further enhanced the reaction, affording **3a** in an 82% yield (Table 1, entry 8). In this reaction, the photocatalyst is indispensable, and the presence of Na_2_S_2_O_4_ and water can dramatically improve reaction yields (Table 1, entries 9–11 and the SI). In the absence of Na_2_S_2_O_4_, compound **3a** can still be obtained in moderate yield. This is likely due to the excess of **1a** acting as a reductant in the reaction.^35^

**Table 1:**
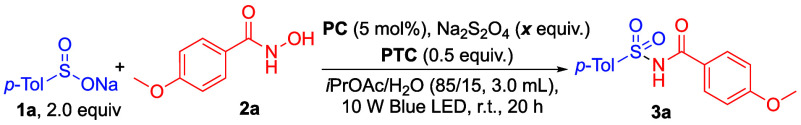
Optimization Study^a^

entry	PC	*x*	PTC	yield^b^ (%)
1	4CzIPN	2.0	/	38
2	MesAcr-ClO_4_	2.0	/	7
3	*f*ac-Ir(ppy)_3_	2.0	/	trace
4	Ru(bpy)_3_Cl_2_	2.0	/	trace
5	4CzBN	2.0	/	46
6	4CzBN	5.0	/	58
7^c^	4CzBN	6.0	BTAC	75
8^c^^,^^d^	4CzBN	6.0	BTAC	82
9^c^^,^^d^	4CzBN	0	BTAC	27
10^c^^,^^d^	/	6.0	BTAC	N.P.
11^d^^,^^e^	4CzBN	6.0	BTAC	trace

a Reaction conditions: **1a** (0.6 mmol), **2a** (0.3 mmol), PC (5 mol %), Na_2_S_2_O_4_ (0.3 × *x* mmol), PTC (0.15 mmol) and *i*PrOAc/H_2_O (85/15, 3.0 mL) were irradiated with 10 W blue LED at room temperature (rt) for 20 h.

b HPLC yield.

c 3.1 mL solvent (*i*PrOAc 2.55 mL + H_2_O 0.55 mL).

d Addition of 100 μL AcOH.

e Only *i*PrOAc is used as a solvent.

With the optimal conditions established, we proceeded to investigate the functional group tolerance of hydroxamic acids and sodium organosulfinates for the synthesis of acylsulfonamides (Table 2). Benzohydroxamic acids with various *para*-substituents were successfully converted to acylsulfonamides with good yields (**3a**, **3c**–**3h**), notably achieving a 95% yield with the -*t*Bu substituent (**3d**). Remarkably, the −OH substituent, typically susceptible to nucleophilic substitution, was also compatible with this reaction **(3h** and **3i)**. We examined steric effects on reaction performance using *o*-OMe and *o*–OH substituted benzohydroxamic acids (**3b** and **3i**). The yields for these substrates were reduced compared to their *para*-substituted counterparts, with *o*-OMe showing a particularly pronounced effect (**3b**). Additionally, polyaromatic and heteroaryl hydroxamic acids were employed to produce the corresponding acylsulfonamides with satisfactory yields (**3k**–**3n**). Encouraged by the results with benzohydroxamic acids, we extended this reaction to *N*-hydroxycarbamates. Common alkyl *N*-hydroxycarbamates, such as benzyl, 9-fluorenylmethyl, and -*t*Bu, were well-tolerated, yielding satisfactory results (**3o–3q**). High electron-withdrawing pentafluorobenzohydroxamic acid or alkyl hydroxamic acid did not yield the desired product. Neither *N*-methylated nor *O*-methylated hydroxamic acids underwent the reaction, with most of the starting materials remaining unreacted. This suggests that methylation at either the nitrogen or oxygen position may hinder the reactivity of hydroxamic acids under the reaction conditions.

**Table 2:**
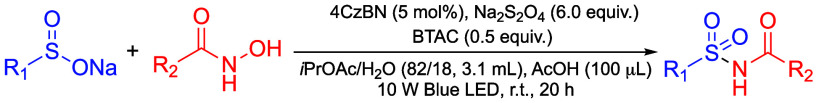

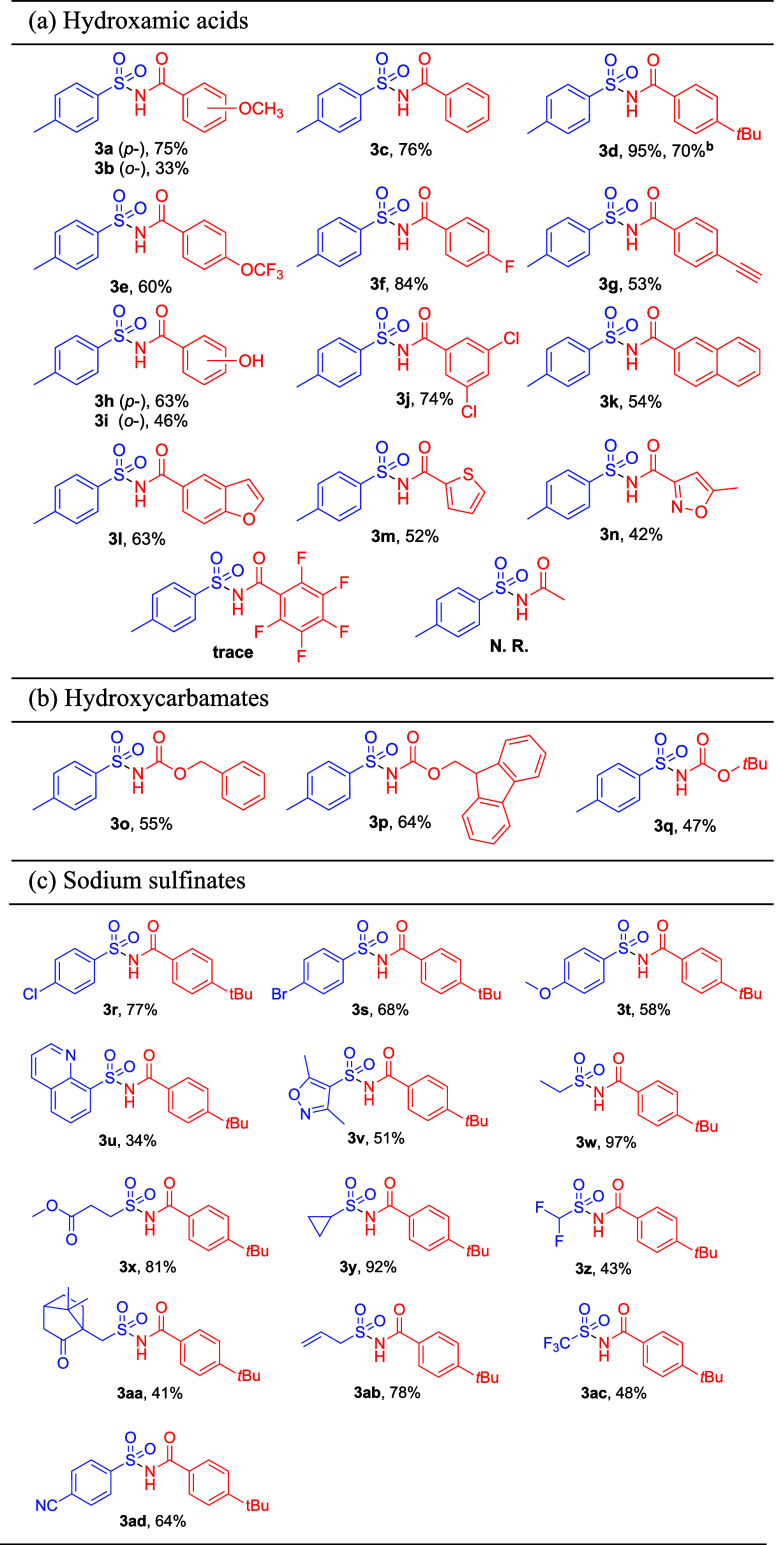
Substrate Scope of the Reaction^*a*^

*^a^*Isolated yield. Reaction conditions: sodium organosulfinate (0.6 mmol), hydroxamic acid (0.3 mmol), 4CzBN (5 mol %), Na_2_S_2_O_4_ (1.8 mmol), BTAC (0.15 mmol), *i*PrOAc (2.55 mL), H_2_O (0.55 mL) and AcOH (100 uL) were stirred under 10 W blue LED irradiation for 20 h at rt. *^b^*1.5 mmol scale.

The scope of sodium arylsulfinate was also evaluated. Halogenated and *p*-OMe-substituted sodium benzenesulfinates served effectively as sulfonyl surrogates, yielding acylsulfonamides in satisfactory to good yields (**3r**–**3t**, **3ad**). When using sodium heteroarylsulfinates, the desired products were obtained in moderate to acceptable yields (**3u** and **3v**). Notably, sodium alkylsulfinates demonstrated compatibility with this reaction, outperforming sodium arylsulfinates. Simple alkyl groups were well-tolerated, achieving excellent yields (**3w**–**3y**). Additionally, more challenging alkyl groups, such as difluoromethyl and trifluoromethyl, camphoric groups, and propyl-2-ene, were successfully utilized to produce the corresponding acylsulfonamides with moderate to good yields (**3z**–**3ac**).

To elucidate the reaction mechanism, a series of control experiments was conducted (see Scheme 2 and the SI). Initially, the reaction was performed under a nitrogen atmosphere, which hindered the formation of **3a**, with over 90% of **2a** recovered from the reaction mixture. This underscores the crucial role of oxygen in the reaction (Scheme 2a). The addition of TEMPO also interfered with the formation of **3a**, resulting in only a 32% yield (Scheme 2b). Time trace analysis of the reaction mixture led to the isolation and identification of *N*-hydroxylacylsulfonamide (**3a′**) as a key intermediate in the formation of **3a** (Scheme 2c and SI). In a radical trap experiment with TEMPO, an *N*-centered radical species was trapped and detected by HRMS, suggesting a radical mechanism for the dehydroxylation of **3a′** to **3a** (Scheme 2d). Upon the addition of 4-phenylbutene as a trapping agent, a [2 + 2] cycloaddition product derived from **2a** was detected by HRMS (an exemplary *exo* product is shown in Scheme 2e). This observation suggests the formation of an acylnitroso as a reaction intermediate.^30^

**Scheme 2:**
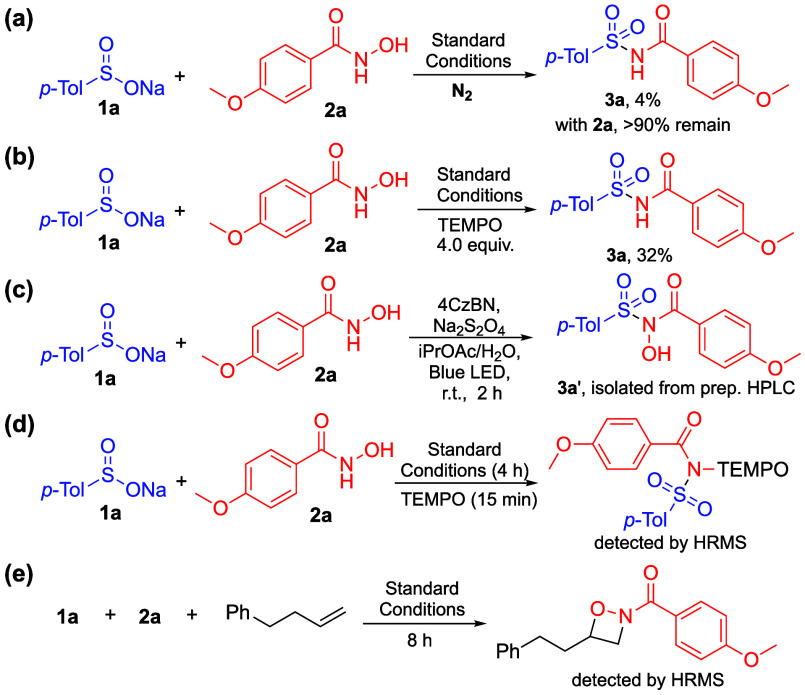
Control Experiments

Additionally, a fluorescence quenching experiment was conducted to identify the potential quenchers of 4CzBN. The result indicated that neither **1a** nor **2a** is an efficient quencher of 4CzBN (Scheme 3a and Figure S1). To further explore the role of oxygen in the reaction, 9,10-anthracenediyl-bis(methylene)dimalonic acid (ABDA) was used as a probe to assess the potential generation of singlet oxygen (^1^O_2_) (Scheme 3b). The formation of ^1^O_2_ would oxidize ABDA to endoperoxide species, resulting in decreased ABDA absorbance.^36^ By irradiating 4CzBN with ABDA at different time intervals, a decrease in ABDA absorbance was observed, suggesting that 4CzBN generates ^1^O_2_.

**Scheme 3:**
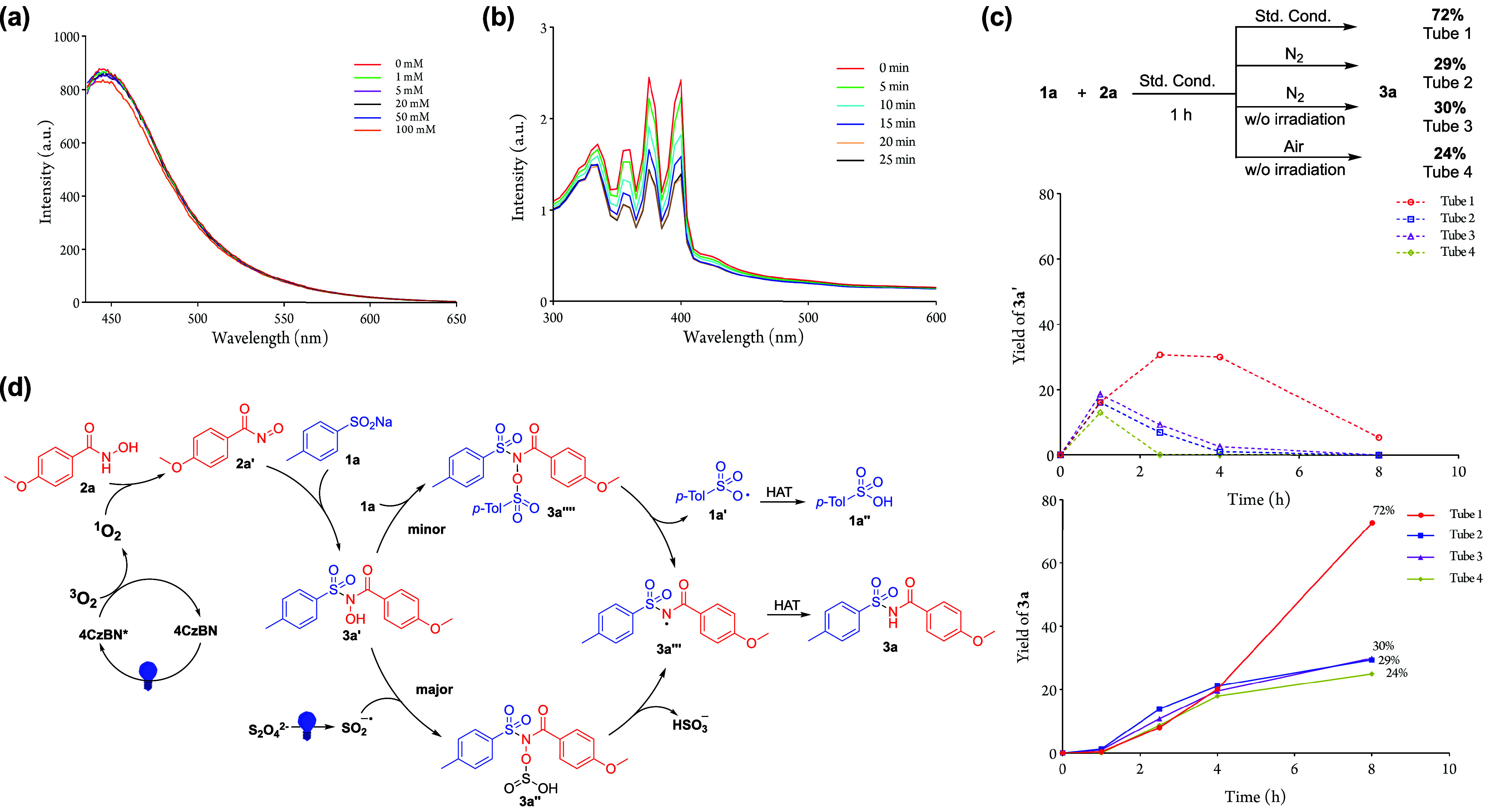
Mechanistic Studies: (a) Fluorescence Spectra of 4CzBN with Various Concentrations of **2a**; (b) UV-Visible Spectra of ABDA with 4CzBN with Various Irradiation Time; (c) Time Trace Analysis of **3a** and **3a′** Formation under Different Conditions; and (d) Plausible Mechanism

Time trace analysis was conducted to study the dehydroxylation process from **3a′** to **3a** (Scheme 3c). Initially, the reaction was irradiated for 1 h to generate **3a′**, followed by continuation under various reaction conditions. The results showed that dehydroxylation proceeded smoothly, regardless of the presence of oxygen or irradiation, suggesting a nonphotocatalytic pathway.

Based on the control experiments and previous literature, a plausible mechanism is proposed (Scheme 3d). The reaction begins with the generation of singlet oxygen ^1^O_2_ via an energy transfer pathway (EnT) from the excited state of 4CzBN (4CzBN*), induced by photoirradiation. The ^1^O_2_ then oxidizes **2a** into a nitroso carbonyl intermediate (**2a′**),^30,37^ which subsequently reacts with **1a** to produce **3a′**.^38^ Next, **3a′** is reduced to an *N*-centered radical species **3a′′′**, by a sulfur dioxide radical anion generated from the decomposition of Na_2_S_2_O_4_,^39^ through the formation of an *N*-sulfinic acid acylsulfonamide adduct **3a′′**. Excess **1a** may also participate in the reduction of **3a′** through the formation of **3a′′′′**, which subsequently leads to **3a′′′** and generates a sulfonyloxyl radical **1a′**.^35^ Finally, the desired product, **3a**, is obtained through a hydrogen atom transfer (HAT) process involving **3a′′′**.

In conclusion, we have developed a photocatalytic S–N coupling reaction between hydroxamic acid and sodium sulfinate for the synthesis of acylsulfonamides using the cyanoarene-based photocatalyst, 4CzBN. a diverse range of acylsulfonamides was successfully synthesized. Mechanistic studies suggest that EnT occurs between 4CzBN* and O_2_, generating ^1^O_2_ to oxidize hydroxamic acid into a nitrosocarbonyl intermediate, facilitating further reaction.

## Data Availability

The data underlying this study are available in the published article and its Supporting Information.

## Supporting Information Available

Procedures, figures, full optimization conditions, characterization, and NMR spectra (PDF)
