# Supplementary material for: Transition-Metal Free Photocatalytic Synthesis of Acylsulfonamides
Source: Org Lett. 2025 Apr 30;27(18):4732–6. doi: 10.1021/acs.orglett.5c01129 (PMC12070461; doi:10.1021/acs.orglett.5c01129)
Supplement: Supplementary file 1 — ol5c01129_si_001.pdf [file ol5c01129_si_001.pdf]

Supporting Information

Transition-Metal Free Photocatalytic Synthesis of Acylsulfonamides

Long Yin Lam, Cong Ma*

State Key Laboratory of Chemical Biology and Drug Discovery, Department of Applied Biology and Chemical Technology, PolyU Marshall Research Centre for Medical Microbial Biotechnology, The Hong Kong Polytechnic University, Kowloon, Hong Kong SAR, China

Table of Contents

General Information **S2**

Experimental Procedures **S3-S6**

A. General Procedure for the Synthesis of Acylsulfonamide S3
B. General Procedure for the Synthesis of 4CzBN S4
C. General Procedure for the Synthesis of Starting Materials S5
D. General Procedure for Optimization study and HRMS analysis S6

Experimental Results **S7-S17**

A. Optimization Study S7-S8
B. HPLC analysis of Reactions S9-S12
C. HRMS analysis of Reaction crude S13-S15
D. Spectrophotometric Analysis S16-S17

Compound Characterization Data **S18-S32**

NMR spectra **S33-S77**

References **S78**

General Information:

All chemicals were of chemical pure grade quality and used without further purification, all organic solvents were analytical pure grade quality. All the reaction required heating were performed using magnetic stirrer (DLAB MS7-H550-S) with mantle. All the photocatalytic reactions were performed using 450 nm LEDs (10 W x 4) illumination instrument from Puri Materials. Reactions were monitored by TLC (Merck silica gel 60 F254), column chromatography was performed on silica gel of 300~400 mesh. All ^{1}H NMR, ^{13}C NMR spectra were recorded on a Bruker Avance 600 spectrometer or Bruker 400 spectrometer in Acetone-d_6, DMSO-d_6 or CD_3Cl and reported in parts per million (ppm, δ). The following abbreviations were used to designate chemical shift multiplicities: s = singlet, d = doublet, t = triplet, q = quartet, m = multiplet. High-resolution mass spectrum (HRMS) was measured by the ESI-TOF or EI-TOF method. The optimization study is performed using HPLC (Waters semi-preparative high performance liquid chromatography system with 2535 quaternary gradient module, 2707 autosampler, 2998 photodiode array detector) on reverse phase column (XBridge C18, 5 µm particle size, 4.6 x 250 mm) with Water/Acetonitrile as eluent. The UV-visible spectrum and fluorescence was recorded with Agilent Cary 60 UV-visible spectrometer and Agilent Cary Eclipsed Fluorescence Spectrometer.

Experimental Procedures

A. General Procedure for the Synthesis of Acylsulfonamides

1. 0.3 mmol scale

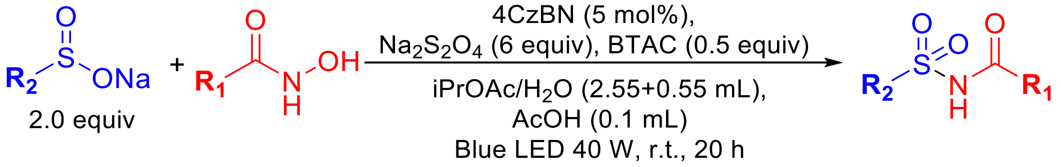

To 35 mL oven-dried heavy wall cylindrical vessels equipped with magnetic stir bar, hydroxamic acid (0.3 mmol, 1 equiv.), 4CzBN[1, 2] (12.0 mg, 0.015 mmol, 0.05 equiv.), $Na_2S_2O_4$ (298.0 mg, 1.8 mmol, 6.0 equiv,), BTAC (30.0 mg, 0.15 mmol, 0.5 equiv.) and sodium organosulfinate (0.6 mmol, 2.0 equiv.) were added in *i*PrOAc (2.55 mL), followed by H_2O (0.55 mL) and AcOH (100 µL). The reaction system was subsequently left open and irradiated with a 10 W 450 nm LED positioned 1 cm from the light source, without using any filter, for a duration of 20 hours. After the reaction complete, as monitored by TLC, was added $NaHCO_3$ powder to neutralize the remaining AcOH in the reaction system. The reaction mixture was then diluted with acetone and filtered. The filtrate was then concentrated under reduced pressure and purified with column chromatography (silica gel, DCM/MeOH) to afford the desired product.

2. 1.5 mmol scale

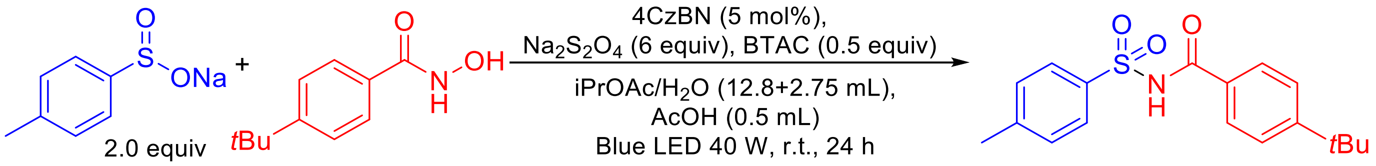

To 35 mL oven-dried heavy wall cylindrical vessels equipped with magnetic stir bar, hydroxamic acid (1.5 mmol, 1 equiv.), 4CzBN (60.0 mg, 0.075 mmol, 0.05 equiv.), $Na_2S_2O_4$ (1.49 g, 9.0 mmol, 6.0 equiv,), BTAC (150 mg, 0.75 mmol, 0.5 equiv.) and sodium organosulfinate (3.0 mmol, 2.0 equiv.) were added in *i*PrOAc (12.8 mL), followed by H_2O (2.75 mL) and AcOH (500 µL). The reaction system was subsequently left open and irradiated with a 10 W 450 nm LED positioned 1 cm from the light source, without using any filter, for a duration of 20 hours. After the reaction complete, as monitored by TLC, was added $NaHCO_3$ powder to neutralize the remaining AcOH in the reaction system. The reaction mixture was then diluted with acetone and filtered. The filtrate was then concentrated under reduced pressure and purified with column chromatography (silica gel, DCM/MeOH) to afford the desired acylsulfonamide **3d** in 70% yield (348.1 mg) as a white solid.

B. General Procedure for Synthesis of ***4CzBN***[3]

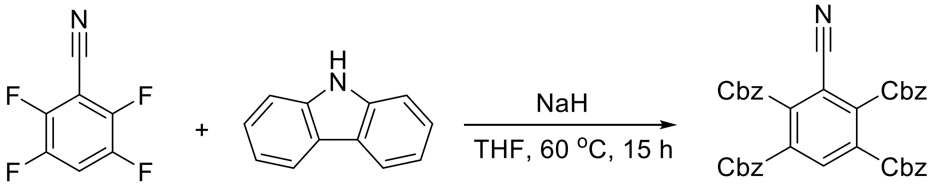

To a 250 mL two-necked round bottom flask equipped with magnetic stir bar was charged with carbazole (1.2 equiv. with respect to each F-atom on cyanobenzene) in dry THF (30 mL) under N_2 protection. Sodium hydride (60% suspension on mineral oil, 2.0 equiv. with respect to each F-atom on cyanobenzene) was added to the slowly to the stirred solution. After stirring for 45 min, 2,3,5,6-tetrafluorobenzonitrile (875 mg, 5 mmol, 1.0 eq) was added and the resulting solution was stirred at 60 °C for further 15 h. Water was added to the reaction mixture to quench the excess NaH, followed by the addition of MeOH to precipitate the solid out. After filtration, the solid was then washed with hexane three times. The solid collected was triturated with ACN/Acetone (9/1) at r.t. for 2 h followed by filtration to afford the desired 4CzBN which then used without further purification.

C. General Procedure for Synthesis of Starting Materials

1. Preparation of sodium organosulfinates

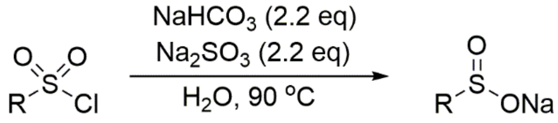

To a 100 mL round-bottom flask equipped with magnetic stir bar, 1848 mg $NaHCO_3$ (22.0 mmol, 2.2 equiv.) and 2772 mg Na_2SO_3 (22.0 mmol, 2.2 equiv.) were added in 12 mL of H_2O and stirred at 90 ℃ for 1 h. Aryl sulfonyl chloride (10 mmol, 1.0 equiv.) was then added into the reaction system and stirred for 3 hours. After the reaction was completed, the solvent was removed under reduced pressure. 50 mL EtOH were added into the flask and stirred for 2 hours. The solution was then filtered, and the filtrate was concentrated *in vacuo* and was further recrystallized using EtOH two times to afford the desired sodium aryl sulfinate.

2. Preparation of hydroxamic acids / hydroxycarbamates (Method A)

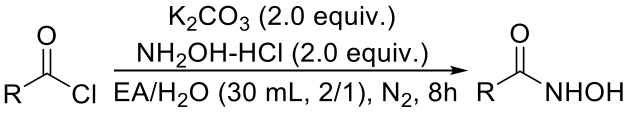

To a 250 mL two-necked round bottom flask equipped with magnetic stir bar was charged with K_2CO_3 (2.0 equiv) and NH_2OH-HCl (2.0 equiv.) in EA/H_2O (30 mL, 2/1). After stirring for 30 min, acyl chloride (15 mmol, 1.0 equiv.) dissolved in minimum amount of EA was added slowly into it, and the resulting solution was stirred for further 8 h. Upon completion of reaction, the reaction mixture was washed with EA twice. The combined organic layer was dried over anhydrous Na_2SO_4 and concentrated under reduced pressure. The residue was then purified by recrystallization using Hexane/EA to provide the desired hydroxamic acid which was used directly without further purification.

3. Preparation of hydroxamic acids (Method B)

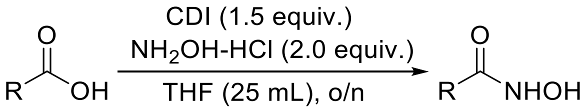

To a 100 mL round bottom flask equipped with magnetic stir bar was charged with the carboxylic acid (15 mmol, 1.0 equiv.) and CDI (1.5 equiv.) in THF (25 mL). After stirring for 1 h at r.t., NH_2OH-HCl was added to the reaction mixture and stirred overnight. Upon completion, HCl was added to quench the reaction, and the mixture was washed with EA twice. The combined organic layer was dried over anhydrous Na_2SO_4 and concentrated under reduced pressure. The residue was then purified by column chromatography (Hexane/EA) to afford the desired hydroxamic acid.

D. General Procedure for Optimization Study and HRMS analysis

To 35 mL oven-dried heavy wall cylindrical vessels equipped with magnetic stir bar, 4-methoxybenzohydroxamic acid (0.3 mmol, 1 equiv.), photocatalyst (0.015 mmol, 0.05 equiv.), reductant, phase-transfer catalyst and sodium *p*-toluenesulfinate (0.6 mmol, 2.0 equiv.) were added in the solvent. The reaction system was then left open and irradiated under 10 W 450 nm LED for 20 h. After the reaction completed, EtOAc was added into the reaction system for dilution to 4 mL and filtered with syringe filter (13 mm x 0.45 μm). 200 μL of solution was taken and diluted with 0.9 mL of HPLC grade acetonitrile, then 100 μL of this diluted solution was taken and diluted with 1 mL of HPLC grade acetonitrile. Finally, 10 μL of internal standard (*p*-toluenesulfonamide, 0.3 M in DMSO) was added. The final solution was then used for HPLC analysis using photodiode array detector for absorbance at 240 nm with the following gradient or as a sample in HRMS analysis.

HPLC gradient:

	Time (min)	Flow rate (mL/min)	% Water	% Acetonitrile
1	initial	1	95.0	5.0
2	6.5	1	15.0	85.0
3	13.0	1	10.0	90.0
4	15.0	1	95.0	5.0
5	20.0	1	95.0	5.0

Experimental Results

A. Optimization Study

Table S1. Optimization Study (photocatalysts, reductants, additives, and solvents)

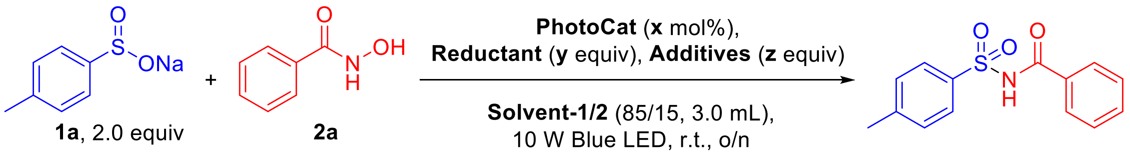

Entry	PhotoCat	x	Reductant	y	Additives	z	Solvent-1/2		Yield (%)
1	4CzIPN	5	$Na_2S_2O_4$	2.0	/	/	**EtOAc**	AcOH	16
2	4CzIPN	5	$Na_2S_2O_4$	2.0	/	/	***n*BuOAc**	AcOH	17
3	4CzIPN	5	$Na_2S_2O_4$	2.0	/	/	**CF_3EAA**	AcOH	9
4	4CzIPN	5	$Na_2S_2O_4$	2.0	/	/	***i*PrOAc**	AcOH	21
5	4CzIPN	5	$Na_2S_2O_4$	2.0	/	/	**THF**	AcOH	16
6	4CzIPN	5	$Na_2S_2O_4$	2.0	/	/	**DME**	AcOH	Trace
7	4CzIPN	5	$Na_2S_2O_4$	2.0	/	/	**Sulfolane**	AcOH	20
8	4CzIPN	5	$Na_2S_2O_4$	2.0	/	/	***i*PrOAc**	AcOH	24
9	4CzIPN	5	$Na_2S_2O_4$	2.0	/	/	*i*PrOAc	**EtOH**	Trace
10	4CzIPN	5	$Na_2S_2O_4$	2.0	/	/	*i*PrOAc	**DMF**	Trace
11	4CzIPN	5	$Na_2S_2O_4$	2.0	/	/	*i*PrOAc	**HCO_2H**	Trace
12	4CzIPN	5	$Na_2S_2O_4$	2.0	/	/	*i*PrOAc	**H_2O**	38
13	4CzIPN	5	$Na_2S_2O_4$	2.0	/	/	***i*PrOAc**		Trace
14	4CzIPN	5	**AscNa**	2.0	/	/	*i*PrOAc	H_2O	Trace
15	4CzIPN	5	**K_2SO_3**	2.0	/	/	*i*PrOAc	H_2O	Trace
16	4CzIPN	5	**Na_2SO_3**	2.0	/	/	*i*PrOAc	H_2O	Trace
17	4CzIPN	5	**$K_2S_2O_3$**	2.0	/	/	*i*PrOAc	H_2O	Trace
18	4CzIPN	5	**DABCO**	2.0	/	/	*i*PrOAc	H_2O	Trace
19	4CzIPN	5	**TEA**	2.0	/	/	*i*PrOAc	H_2O	Trace
20	4CzIPN	5	$Na_2S_2O_4$	2.0	**Hantzsch**	**1.0**	*i*PrOAc	H_2O	Trace
21	4CzIPN	5	$Na_2S_2O_4$	2.0	**K_2CO_3**	**3.0**	*i*PrOAc	H_2O	Trace
22	**$Ru(bpy)_3Cl_2$**	5	$Na_2S_2O_4$	2.0	/	/	*i*PrOAc	H_2O	20
23	**$Ru(bpy)_3(PF_6)_2$**	5	$Na_2S_2O_4$	2.0	/	/	*i*PrOAc	H_2O	17
24	**$Ru(phen)_3Cl_2$**	5	$Na_2S_2O_4$	2.0	/	/	*i*PrOAc	H_2O	14
25	**$Ru(phen)_3(PF_6)_2$**	5	$Na_2S_2O_4$	2.0	/	/	*i*PrOAc	H_2O	17
26	**MesAcr-ClO_4**	5	$Na_2S_2O_4$	2.0	/	/	*i*PrOAc	H_2O	7
27	**4CzIPN**	5	$Na_2S_2O_4$	2.0	/	/	*i*PrOAc	H_2O	21
28	***fac*-$Ir(ppy)_3$**	5	$Na_2S_2O_4$	2.0	/	/	*i*PrOAc	H_2O	6
29	**$Ru(bpz)_3(PF_6)_2$**	5	$Na_2S_2O_4$	2.0	/	/	*i*PrOAc	H_2O	24
30	**4CzIPN-*t*Bu**	5	$Na_2S_2O_4$	2.0	/	/	*i*PrOAc	H_2O	33
31	**4CzIPN-Ph**	5	$Na_2S_2O_4$	2.0	/	/	*i*PrOAc	H_2O	42
32	**4CzBN**	5	$Na_2S_2O_4$	2.0	/	/	*i*PrOAc	H_2O	46
33	**5CzBN**	5	$Na_2S_2O_4$	2.0	/	/	*i*PrOAc	H_2O	40
34	4CzBN	5	$Na_2S_2O_4$	**1.0**	/	/	*i*PrOAc	H_2O	27
35	4CzBN	5	$Na_2S_2O_4$	**3.0**	/	/	*i*PrOAc	H_2O	45
36	4CzBN	5	$Na_2S_2O_4$	**5.0**	/	/	*i*PrOAc	H_2O	58
37	4CzBN	5	$Na_2S_2O_4$	**7.5**	/	/	*i*PrOAc	H_2O	31

Table S2. Optimization Study (additives, solvents)

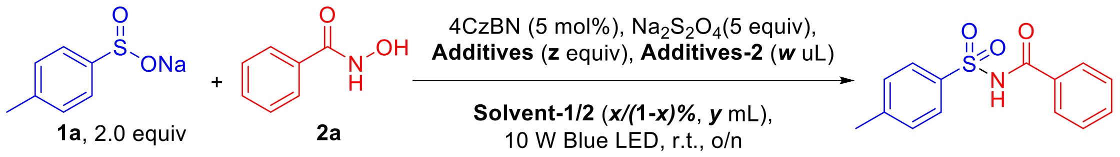

Entry	Additives	z	Additives-2	*w*	Solvent-1/2		*x*	*y*	Yield (%)
1	BTAC	0.5	/	/	*i*PrOAc	H_2O	85	3.0	58
2	**TBAB**	0.5	/	/	*i*PrOAc	H_2O	85	3.0	40
3	**Triton B**	0.5	/	/	*i*PrOAc	H_2O	85	3.0	37
4	**18-crown-6**	0.5	/	/	*i*PrOAc	H_2O	85	3.0	62
5	**CTAB**	0.5	/	/	*i*PrOAc	H_2O	85	3.0	57
6	**TBA-**	0.5	/	/	*i*PrOAc	H_2O	85	3.0	60
7	**TBA-NO_3**	0.5	/	/	*i*PrOAc	H_2O	85	3.0	46
8	**TBA-PF_6**	0.5	/	/	*i*PrOAc	H_2O	85	3.0	23
9	**BTAC**	0.5	/	/	*i*PrOAc	H_2O	85	3.0	65
10	BTAC	**0.2**	/	/	*i*PrOAc	H_2O	85	3.0	60
11	BTAC	**1.0**	/	/	*i*PrOAc	H_2O	85	3.0	61
12	BTAC	0.5	/	/	*i*PrOAc	H_2O	**82**	**3.1**	71
13[a]	BTAC	0.5	/	/	*i*PrOAc	H_2O	82	3.1	75
14[a]	BTAC	0.5	**AcOH**	**50**	*i*PrOAc	H_2O	82	3.1	75
15[a]	BTAC	0.5	**AcOH**	**100**	*i*PrOAc	H_2O	82	3.1	82
16[a]	BTAC	0.5	**AcOH**	**200**	*i*PrOAc	H_2O	82	3.1	33
17[a,b]	BTAC	0.5	AcOH	100	*i*PrOAc	H_2O	82	3.1	14
18[a,c]	BTAC	0.5	AcOH	100	*i*PrOAc	H_2O	82	3.1	trace

[a] 6.0 equivalent of $Na_2S_2O_4$ is used. Abbreviation: TBAB = Tetrabutylammonium bromide; CTAB = Cetyltrimethylammonium bromide; TBA = Tetrabutylammonium; BTAC = Benzyltrimethylammonium chloride. [b] Rose Bengal as photocatalyst. [c] Methylene blue as photocatalyst.

B. HPLC Analysis of Reaction

1. HPLC analysis for preliminary mechanistic study

1.1 HPLC analysis by reaction time

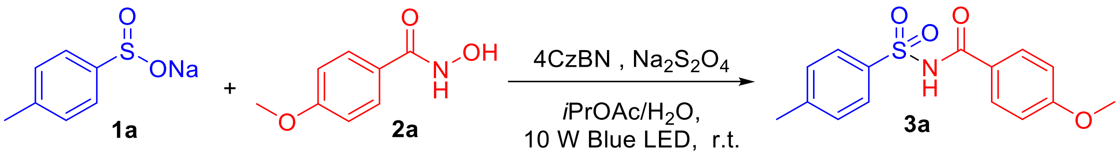

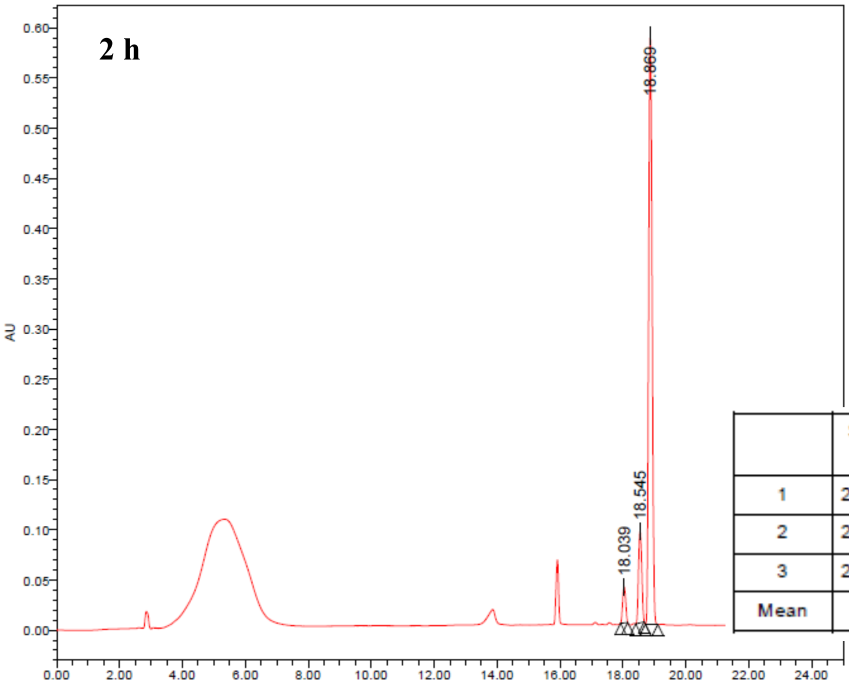

	Sample Name	Vial	Inj	Retention Time (min)	Area	% Area	Height
1	2 h	1:A,2	1	18.039	239396	4.47	35755
2	2 h	1:A,2	1	18.869	4460632	83.29	587181
3	2 h	1:A,2	1	18.545	655792	12.24	90949
Mean				18.484			

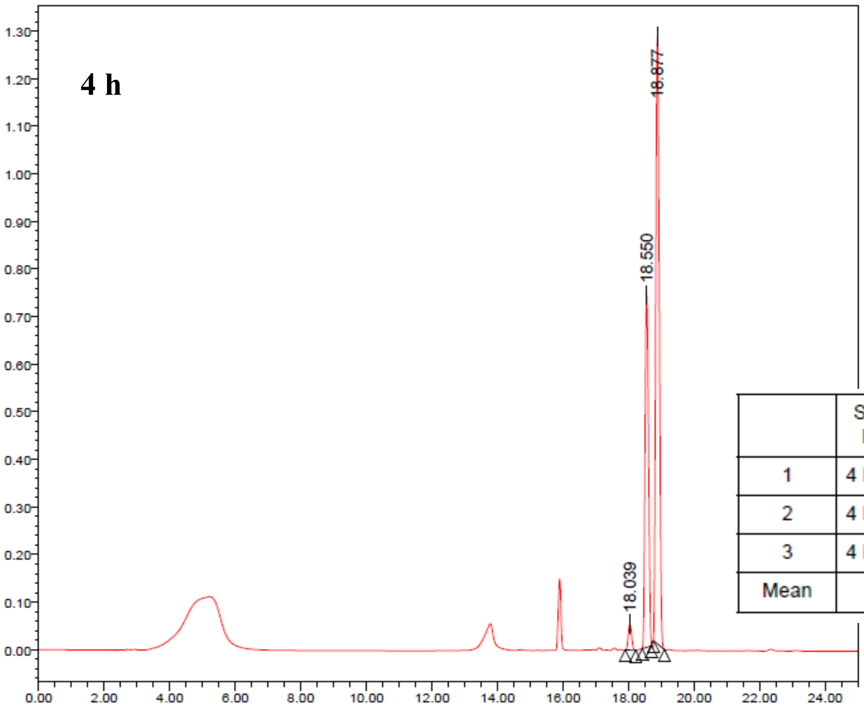

	Sample Name	Vial	Inj	Retention Time (min)	Area	% Area	Height
1	4 h	1:A,4	1	18.039	389845	2.52	54753
2	4 h	1:A,4	1	18.877	9629974	62.29	1275717
3	4 h	1:A,4	1	18.550	5441017	35.19	740291
Mean				18.489			

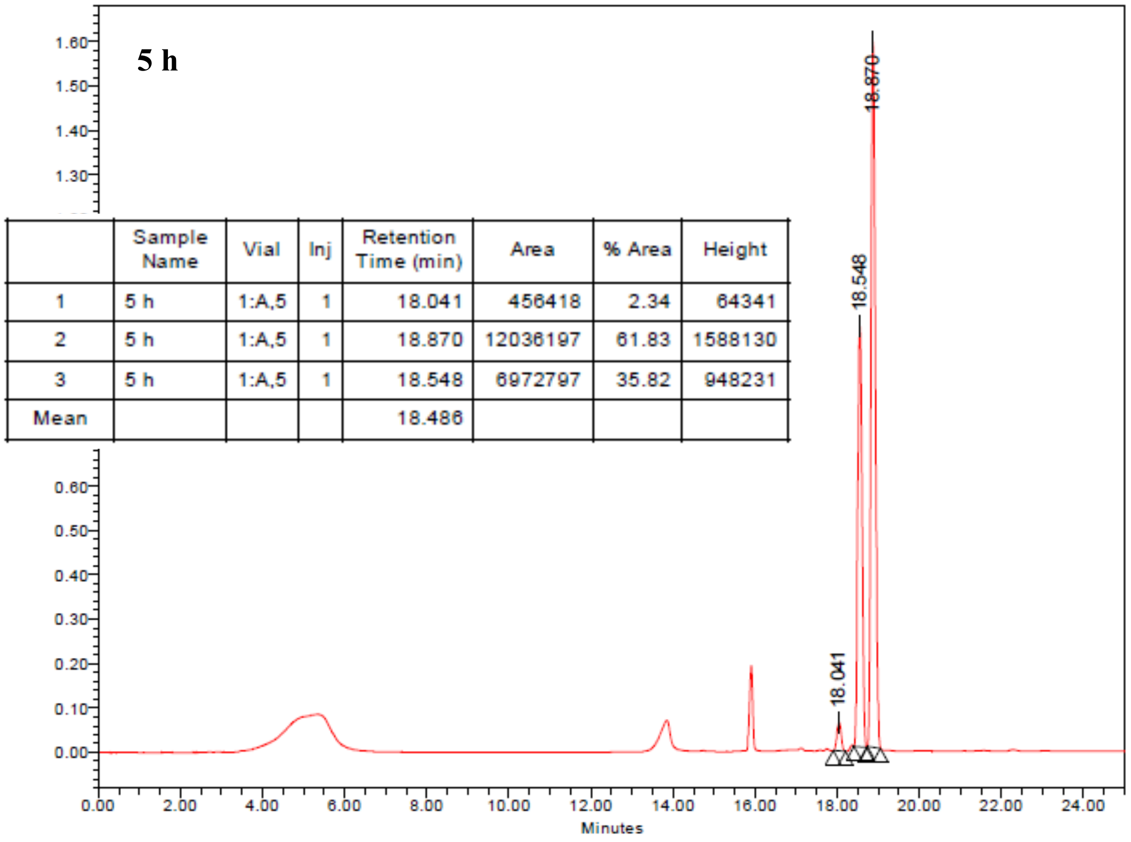

	Sample Name	Vial	Inj	Retention Time (min)	Area	% Area	Height
1	5 h	1:A,5	1	18.041	456418	2.34	64341
2	5 h	1:A,5	1	18.870	12036197	61.83	1588130
3	5 h	1:A,5	1	18.548	6972797	35.82	948231
Mean				18.486			

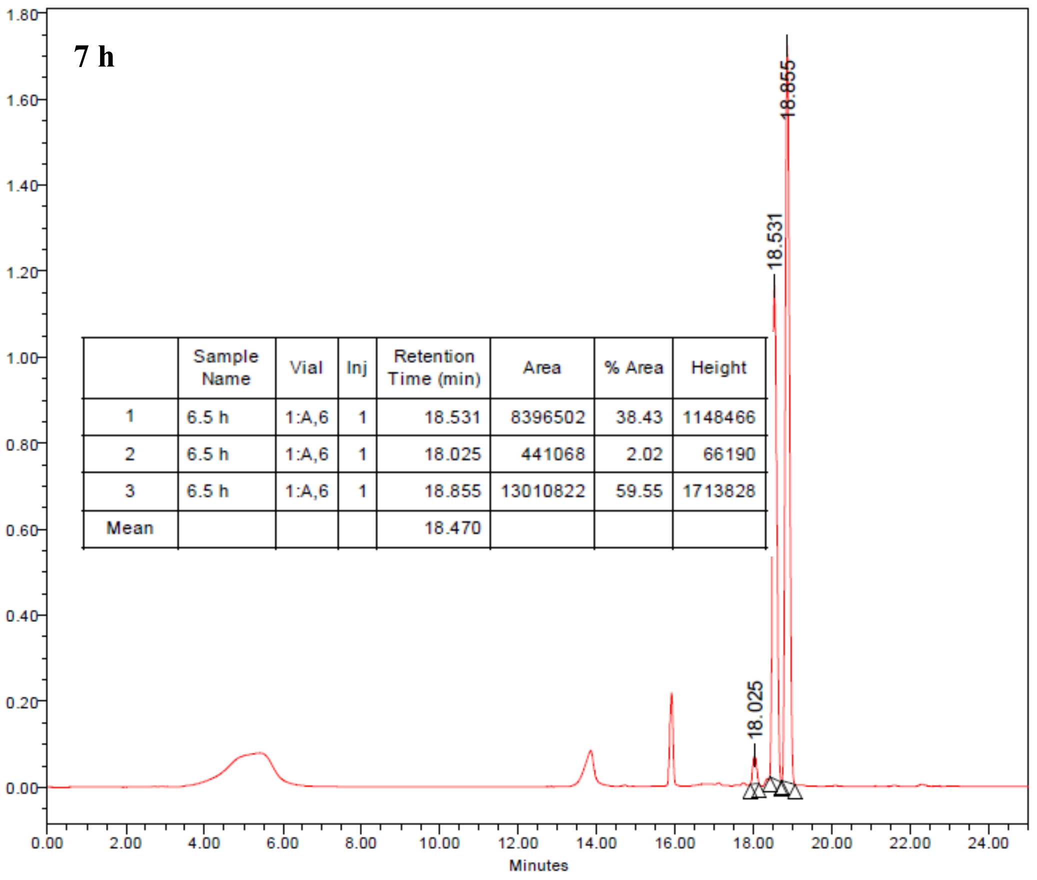

	Sample Name	Vial	Inj	Retention Time (min)	Area	% Area	Height
1	6.5 h	1:A,6	1	18.531	8396502	38.43	1148466
2	6.5 h	1:A,6	1	18.025	441068	2.02	66190
3	6.5 h	1:A,6	1	18.855	13010822	59.55	1713828
Mean				18.470			

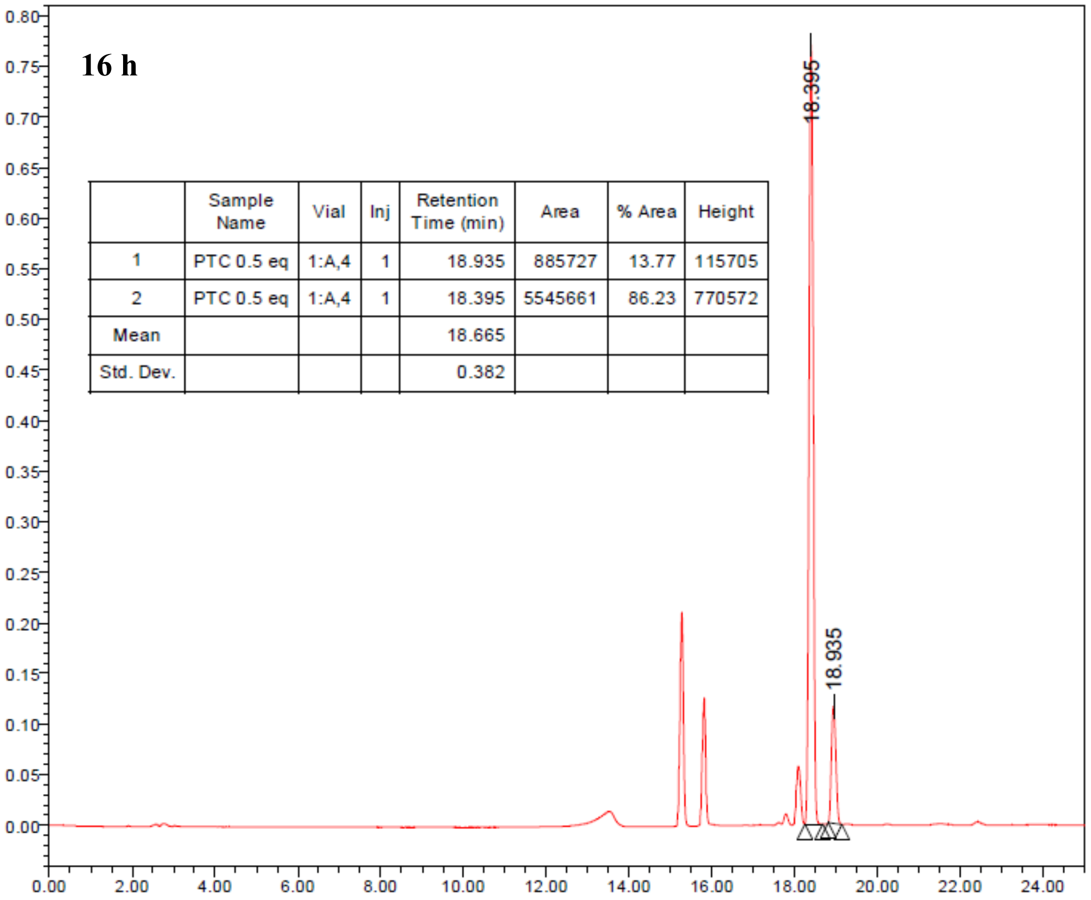

	Sample Name	Vial	Inj	Retention Time (min)	Area	% Area	Height
1	PTC 0.5 eq	1:A,4	1	18.935	885727	13.77	115705
2	PTC 0.5 eq	1:A,4	1	18.395	5545661	86.23	770572
Mean				18.665			
Std. Dev.				0.382			

Based on the result of time trace analysis, the compound with retention time (t_R) 18.9 min would be the dominant product throughout the first 2 hours of reaction, while peak corresponding to **3a** (t_R = 18.5 min) was not obvious. However, as reaction continues, the ratio of these two peaks varies, suggesting the formation of intermediate followed by further conversion to **3a.**

1.2 Reaction mixture without BTAC and AcOH:

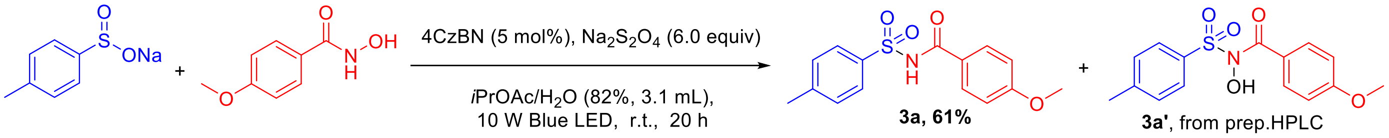

Based on the HPLC analysis on the reaction crude without BTAC, instead of the product **3a**, significant amount of intermediate was obtained. After the isolation of the intermediate with preparative HPLC (Decomposition into **3a** when subjected to column chromatography), the intermediate was characterized as *N*-hydroxy-4-methoxy-*N*-tosylbenzamide (**3a**'). This result suggested the addition of BTAC and AcOH would facilitate the reduction of **3a'** to **3a**. As AcOH is commonly known as H^+ source, we believed the BTAC acted as a phase transfer catalyst for mass transfer of $Na_2S_2O_4$ and **3a**' and hence increased the reduction rate.

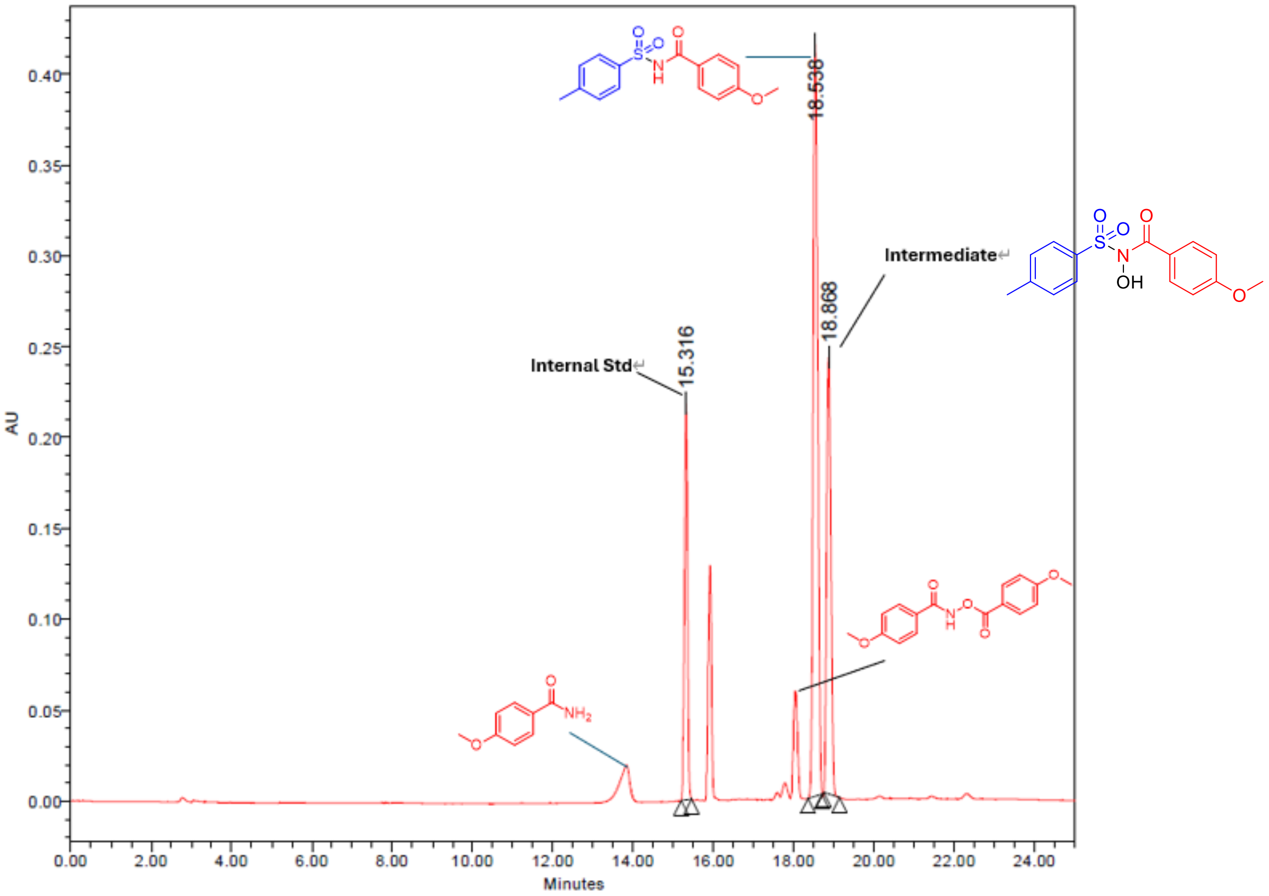

C. HRMS analysis of Trapping Experiment

1. Trapping experiment of radicals

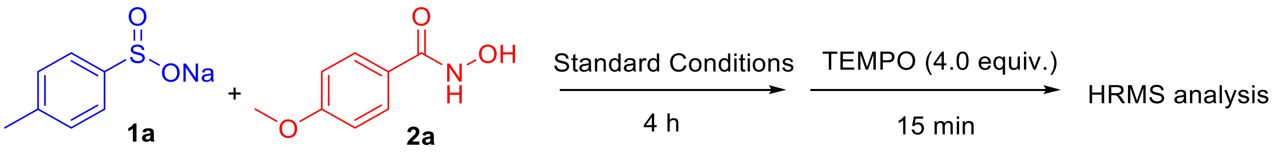

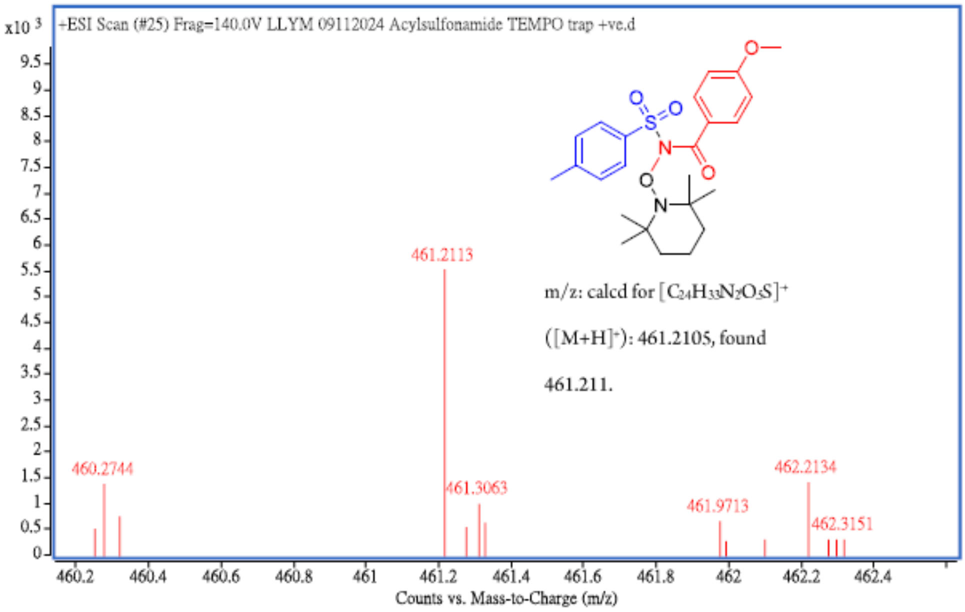

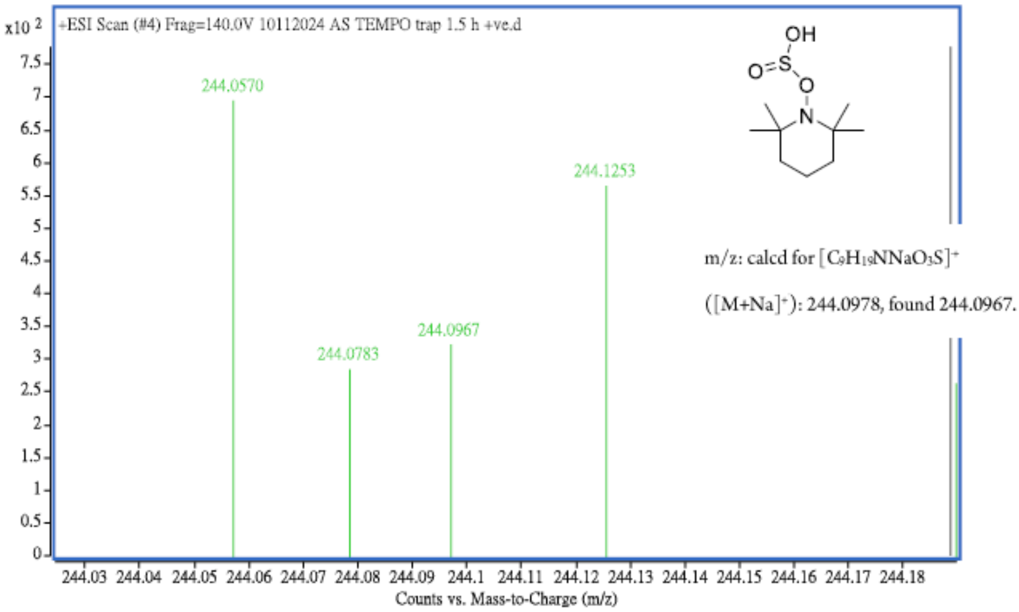

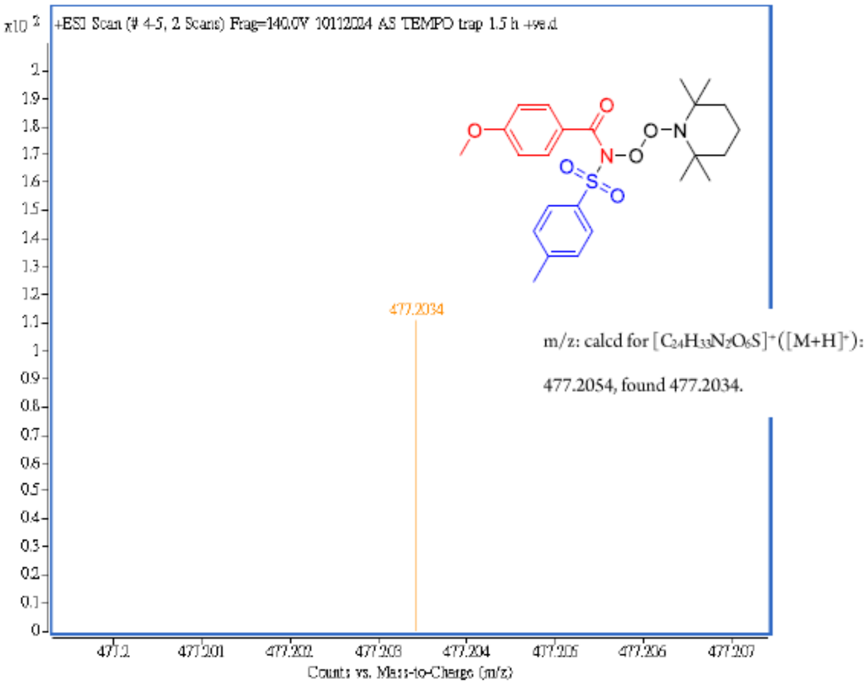
x10 2
+ESI Scan (# 4-5, 2 Scans) Frag=140.0V 10112024 AS TEMPO trap 1.5 h +ve.d
477.2034
m/z: calcd for [$C_{24}H_{33}N_2O_6S$]$^+$([M+H]$^+$):
477.2054, found 477.2034.
Counts vs. Mass-to-Charge (m/z)

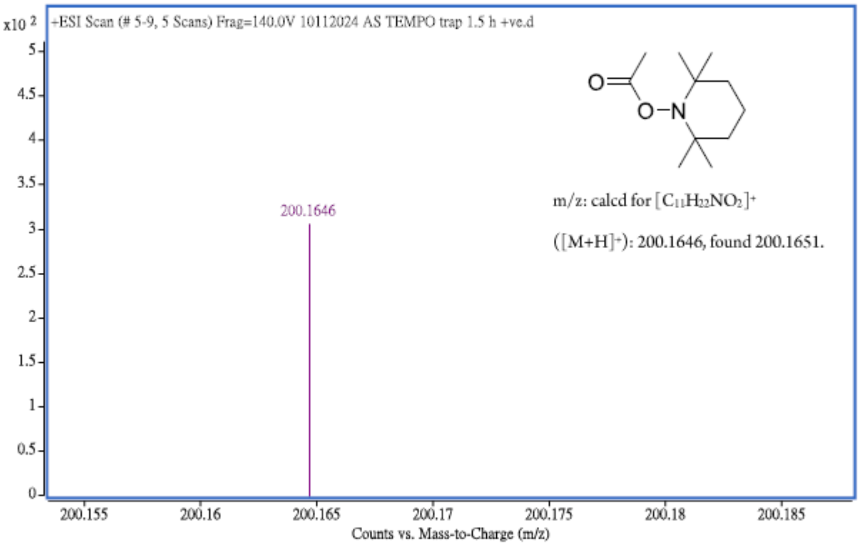
x10 2
+ESI Scan (# 5-9, 5 Scans) Frag=140.0V 10112024 AS TEMPO trap 1.5 h +ve.d
200.1646
m/z: calcd for [$C_{11}H_{22}NO_2$]$^+$
([M+H]$^+$): 200.1646, found 200.1651.
Counts vs. Mass-to-Charge (m/z)

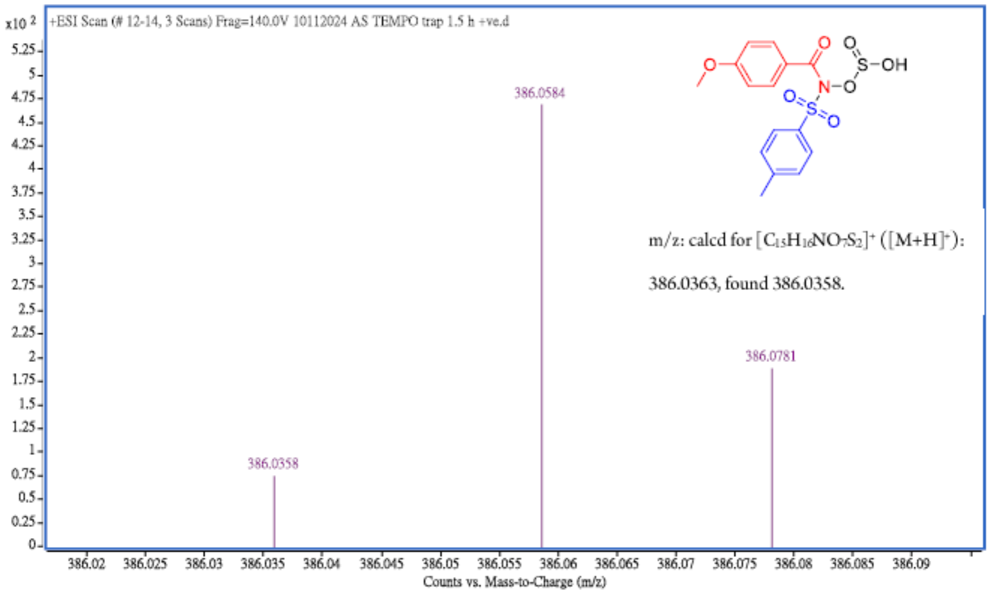
x10 2
+ESI Scan (# 12-14, 3 Scans) Frag=140.0V 10112024 AS TEMPO trap 1.5 h +ve.d
386.0584
386.0781
386.0358
m/z: calcd for [$C_{15}H_{16}NO_7S_2$]$^+$ ([M+H]$^+$):
386.0363, found 386.0358.
Counts vs. Mass-to-Charge (m/z)

2. Trapping experiment of acylnitroso intermediate

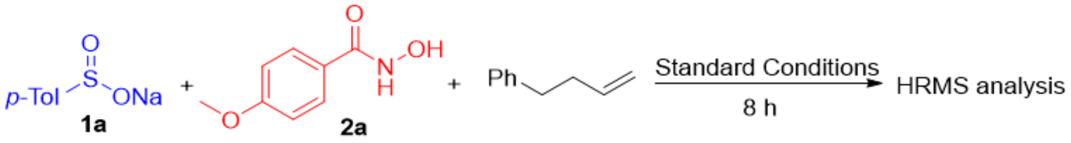

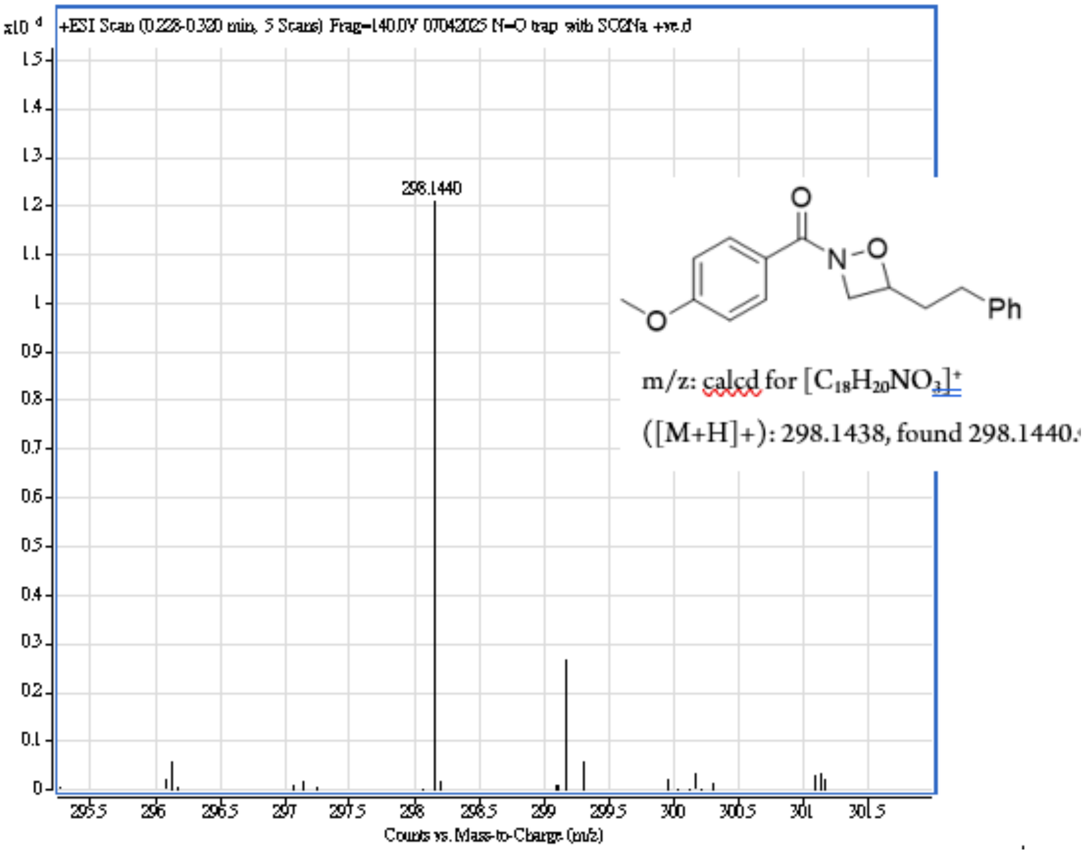

D. Spectrophotometric Analysis

1. Fluorescence Quenching Studies

The stock solutions of 4CzIPN at 0.1 mM in *i*PrOAc, **1a** at 2 mM in *i*PrOAc with 3% AcOH and **2a** at 2 mM in *i*PrOAc were prepared separately. The test solutions were prepared from the stock solutions as tabulated below in a screw-top quartz cuvette at room temperature. After degassing with N_2 for 10 min, the test solutions were excited at 425 nm and fluorescence emission was measured from 435 nm to 650 nm.

Table S3 Volume of stock solution

Test sample	Volume of solution (μL)		
	**4CzBN**	**Sample**	***i*PrOAc**
0 mM	400	0	1600
1 mM	400	10	1590
2 mM	400	20	1580
5 mM	400	50	1550
10 mM	400	100	1500
20 mM	400	200	1400
50 mM	400	500	1100
100 mM	400	1000	600
150 mM	400	1500	100

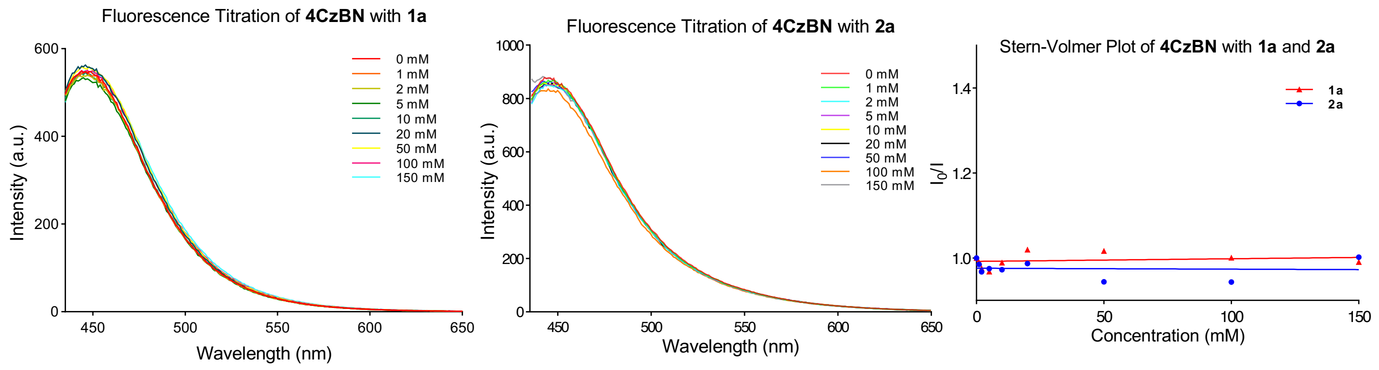

Figure S1. Fluorescence quenching of 4CzBN against varying concentrations of 1a (left) and 2a (middle) and Stern-Volmer plot illustrating quenching behavior of 4CzBN with 1a and 2a (right)

2. UV-visible analysis for $^1O_2$ generation

A stock solution of ABDA with concentration of 2.5 mM in MeOH and 4CzBN with concentration of 0.1 mM in *i*PrOAc were prepared separately. The test solution was prepared in cuvette at room temperature as tabulated below. Afterwards, the absorption of the solution was recorded after different duration of irradiation with the photoreactor.

Table S4. Volumn of each stock solution

Test solution	Volume of stock solution (µL)		
	4CzBN	ABDA	*i*PrOAc
Sample	100	400	2500
Blank	0	400	2600

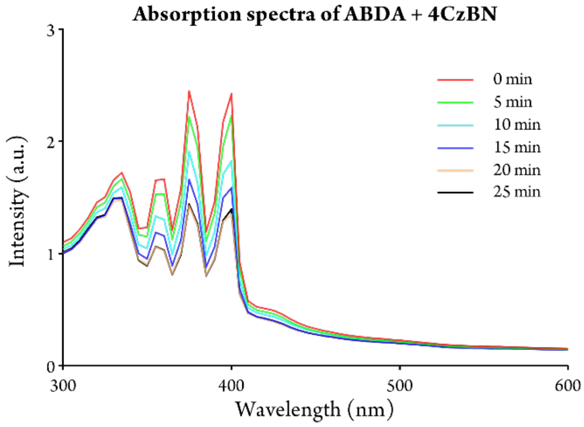

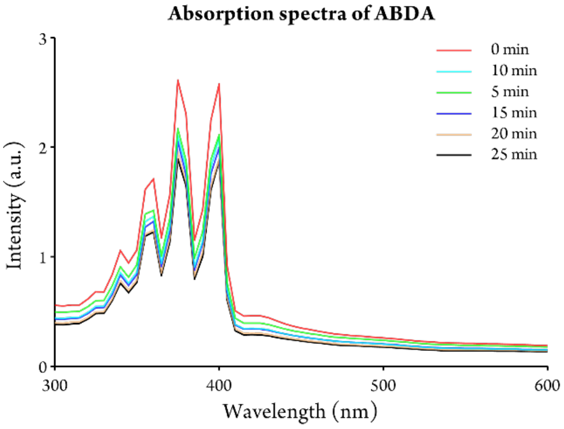

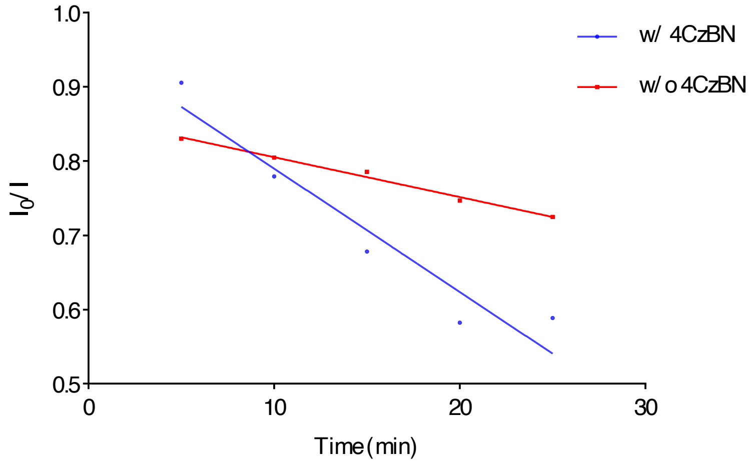

Figure S2. Absorbance plot at 375 nm

Compound Characterization Data

A. Acylsulfonamides

4-methoxy-*N*-tosylbenzamide (3a)

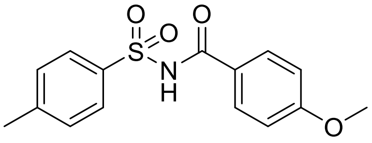

Purification by column chromatography (DCM/MeOH = 100/1); White solid (68.7 mg, 75%)

^{1}H NMR (400 MHz, Acetone-*d*$_6$) δ 10.80 (s, 1H), 7.96 (dd, *J* = 27.1, 8.7 Hz, 4H), 7.43 (d, *J* = 8.1 Hz, 2H), 7.02 (d, *J* = 8.9 Hz, 2H), 3.87 (s, 3H), 2.43 (s, 3H). ^{13}C NMR (151 MHz, DMSO-*d*$_6$) δ 165.4, 163.5, 144.2, 137.7, 131.0, 129.9, 129.8, 128.1, 124.5, 114.3, 56.0, 21.5. HRMS (ESI-TOF) m/z: calcd for $[C_{15}H_{15}NNaO_4S]^+$ ($[M+Na]^+$): 328.0614, found 328.0624.

2-methoxy-*N*-tosylbenzamide (3b)

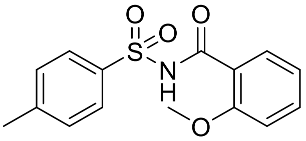

Purification by column chromatography (DCM/MeOH = 100/1); White solid (30.1 mg, 33%)

^{1}H NMR (600 MHz, Acetone-*d*$_6$) δ 8.05 – 8.00 (m, 2H), 7.85 (dd, *J* = 7.8, 1.9 Hz, 1H), 7.60 (ddd, *J* = 8.8, 7.3, 1.9 Hz, 1H), 7.45 (d, *J* = 8.1 Hz, 2H), 7.24 (d, *J* = 8.4 Hz, 1H), 7.10 – 7.07 (m, 1H), 4.08 (s, 3H), 2.44 (s, 3H). ^{13}C NMR (151 MHz, Acetone-*d*$_6$) δ 162.5, 158.0, 144.7, 136.8, 134.8, 131.4, 129.4, 128.4, 121.2, 119.7, 112.3, 56.1, 20.6. HRMS (ESI-TOF) m/z: calcd for $[C_{15}H_{15}NNaO_4S]^+$ ($[M+Na]^+$): 328.0614, found 328.0625.

***N*-tosylbenzamide (3c)**

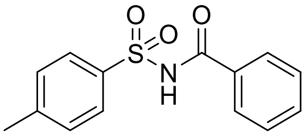

Purification by column chromatography (DCM/MeOH = 100/1); White solid (62.7 mg, 76%)

^{1}H NMR (600 MHz, Acetone-*d*$_6$) δ 8.01 (dd, J = 8.4, 2.3 Hz, 2H), 7.94 (dd, J = 8.2, 1.5 Hz, 2H), 7.66 – 7.61 (m, 1H), 7.51 (t, J = 7.7 Hz, 2H), 7.45 (d, J = 8.0 Hz, 2H), 2.45 (s, 3H). ^{13}C NMR (151 MHz, Acetone-*d*$_6$) δ 164.8, 144.6, 137.0, 133.1, 132.1, 129.4, 128.7, 128.4, 128.1, 20.6. HRMS (ESI-TOF) m/z: calcd for $[C_{14}H_{13}NNaO_3S]^+$ ($[M+Na]^+$): 298.0514, found 298.0520.

4-(tert-butyl)-*N*-tosylbenzamide (3d)

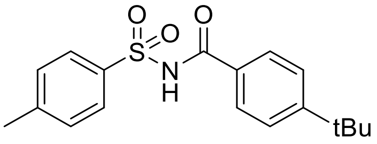

Purification by column chromatography (DCM/MeOH = 100/1); White solid (94.4 mg, 95%) (348.1 mg, 70%, 1.5 mmol scale)

^{1}H NMR (600 MHz, Acetone-d_6) δ 8.02 – 7.98 (m, 2H), 7.90 – 7.87 (m, 2H), 7.56 – 7.54 (m, 2H), 7.44 (d, J = 8.1 Hz, 2H), 2.45 (s, 3H), 1.33 (s, 9H). ^{13}C NMR (151 MHz, Acetone-d_6) δ 164.6, 156.7, 144.5, 137.1, 129.3, 129.2, 128.4, 128.1, 125.6, 125.6, 34.8, 30.4, 20.7. HRMS (ESI-TOF) m/z: calcd for $[C_{18}H_{21}NNaO_3S]^+$ ($[M+Na]^+$): 354.1134, found 354.1144.

***N*-tosyl-4-(trifluoromethoxy)benzamide (3e)**

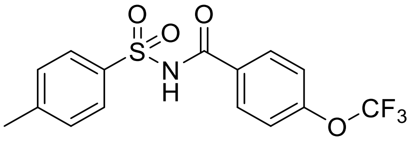

Purification by column chromatography (DCM/MeOH = 100/1); White solid (65.0 mg, 60%)

^{1}H NMR (600 MHz, Acetone-d_6) δ 8.19 – 8.14 (m, 2H), 7.88 (d, J = 8.1 Hz, 2H), 7.23 (dd, J = 17.3, 8.1 Hz, 4H), 2.35 (s, 3H). ^{13}C NMR (151 MHz, Acetone-d_6) δ 170.6, 150.5, 143.3, 140.5, 138.3, 130.8, 128.4, 126.4, 123.0, 120.5 (d, J = 255.7 Hz), 119.6, 118.0, 20.4. ^{19}F NMR (565 MHz, Acetone-d_6) δ -58.4. HRMS (ESI-TOF) m/z: calcd for $[C_{15}H_{12}F_3NNaO_4S]^+$ ($[M+Na]^+$): 382.0331, found 382.0342.

4-fluoro-*N*-tosylbenzamide (3f)

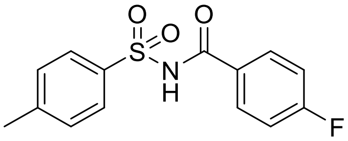

Purification by column chromatography (DCM/MeOH = 100/1); White solid (73.5 mg, 84%)

^{1}H NMR (600 MHz, Acetone-d_6) δ 8.10 – 8.04 (m, 2H), 7.84 (d, J = 8.0 Hz, 2H), 7.17 (d, J = 8.0 Hz, 2H), 7.03 – 6.98 (m, 2H), 2.31 (s, 3H). ^{13}C NMR (151 MHz, Acetone-d_6) δ 171.0, 164.2 (d, J = 256.7 Hz), 143.5, 140.3, 135.8, 135.7, 131.2, 131.16 (d, J = 8.7 Hz), 126.4, 113.83 (d, J = 21.5 Hz), 20.4. ^{19}F NMR (565 MHz, Acetone-d_6) δ -113.3. HRMS (ESI-TOF) m/z: calcd for $[C_{14}H_{12}FNNaO_3S]^+$ ($[M+Na]^+$): 316.0414, found 316.0420.

4-ethynyl-*N*-tosylbenzamide (3g)

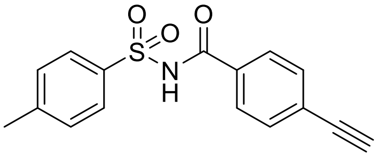

Purification by column chromatography (DCM/MeOH = 100/1); White solid (47.7 mg, 53%)

^{1}H NMR (600 MHz, Acetone-d_6) δ 12.15 (s, 1H), 7.43 (d, *J* = 8.2 Hz, 2H), 7.40 (d, *J* = 8.3 Hz, 2H), 7.13 (d, *J* = 8.2 Hz, 2H), 6.99 (d, *J* = 8.1 Hz, 2H), 4.03 (s, 1H), 1.95 (s, 3H). ^{13}C NMR (151 MHz, DMSO-d_6) δ 164.8, 144.2, 136.7, 134.3, 131.8, 129.5, 128.7, 127.8, 126.3, 84.0, 82.6, 21.1. HRMS (ESI-TOF) m/z: calcd for $[C_{16}H_{13}NNaO_3S]^+$ ($[M+Na]^+$): 322.0508, found 322.0518.

4-hydroxy-*N*-tosylbenzamide (3h)

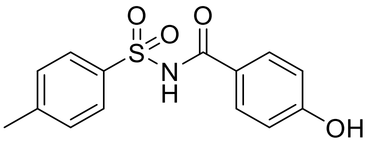

Purification by column chromatography (DCM/MeOH = 100/1); Purple solid (54.7 mg, 63%)

^{1}H NMR (600 MHz, Acetone-d_6) δ 7.83 (d, *J* = 8.3 Hz, 2H), 7.71 (d, *J* = 8.7 Hz, 2H), 7.27 (d, *J* = 8.0 Hz, 2H), 6.76 (d, *J* = 8.7 Hz, 2H), 2.28 (s, 3H). ^{13}C NMR (151 MHz, Acetone-d_6) δ 164.3, 161.9, 144.3, 137.4, 130.5, 129.2, 128.3, 123.2, 115.3, 20.6. HRMS (ESI-TOF) m/z: calcd for $[C_{14}H_{13}NNaO_4S]^+$ ($[M+Na]^+$): 314.0457, found 314.0465.

2-hydroxy-*N*-tosylbenzamide (3i)

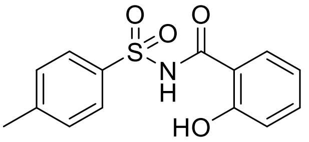

Purification by column chromatography (DCM/MeOH = 100/1); Orange solid (40.0 mg, 46%)

^{1}H NMR (600 MHz, Acetone-d_6) δ 8.05 – 7.99 (m, 2H), 7.94 (dd, *J* = 8.1, 1.7 Hz, 1H), 7.51 (ddd, *J* = 8.6, 7.2, 1.7 Hz, 1H), 7.46 (d, *J* = 8.1 Hz, 2H), 7.01 (dd, *J* = 8.4, 1.1 Hz, 1H), 6.97 (ddd, *J* = 8.2, 7.2, 1.1 Hz, 1H), 2.45 (s, 3H). ^{13}C NMR (151 MHz, Acetone-d_6) δ 166.4, 159.6, 144.8, 136.8, 135.6, 129.6, 129.4, 128.4, 119.8, 117.6, 114.9, 20.6. HRMS (ESI-TOF) m/z: calcd for $[C_{14}H_{13}NNaO_4S]^+$ ($[M+Na]^+$): 314.0457, found 314.0469.

3,5-dichloro-*N*-tosylbenzamide (3j)

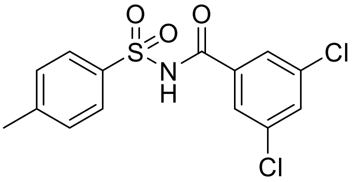

Purification by column chromatography (DCM/MeOH = 100/1); White solid (76.1 mg, 74%)

^{1}H NMR (600 MHz, Acetone-d_6) δ 7.95 (d, J = 2.0 Hz, 2H), 7.93 (d, J = 8.0 Hz, 2H), 7.50 (d, J = 1.6 Hz, 1H), 7.24 (d, J = 7.9 Hz, 2H), 2.35 (s, 3H). ^{13}C NMR (151 MHz, Acetone-d_6) δ 169.1, 141.7, 141.5, 141.4, 134.1, 130.2, 128.9, 127.5, 126.2, 20.4. HRMS (ESI-TOF) m/z: calcd for $[C_{14}H_{11}Cl_2NNaO_3S]^+$ ($[M+Na]^+$): 365.9729, found 365.9735.

***N*-tosyl-2-naphthamide (3k)**

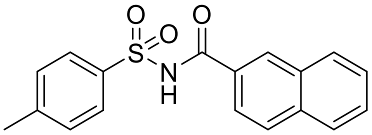

Purification by column chromatography (DCM/MeOH = 100/1); White solid (53.1 mg, 54%)

^{1}H NMR (600 MHz, DMSO-d_6) δ 8.47 (s, 1H), 8.04 – 7.95 (m, 2H), 7.92 – 7.87 (m, 1H), 7.82 (d, *J* = 8.6 Hz, 1H), 7.76 (d, *J* = 7.9 Hz, 2H), 7.51 (td, *J* = 6.7, 6.1, 1.6 Hz, 2H), 7.20 (d, *J* = 7.9 Hz, 2H), 2.32 (s, 3H). ^{13}C NMR (151 MHz, DMSO-d_6) δ 170.2, 144.0, 139.8, 137.3, 134.3, 132.8, 129.3, 128.6, 128.5, 127.9, 127.4, 127.1, 127.1, 126.4, 126.4, 21.3. HRMS (ESI-TOF) m/z: calcd for $[C_{18}H_{15}NNaO_3S]^+$ ($[M+Na]^+$): 348.0665, found 348.0674.

***N*-tosylbenzofuran-5-carboxamide (3l)**

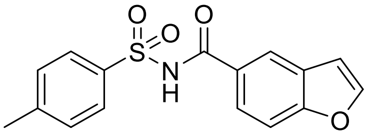

Purification by column chromatography (DCM/MeOH = 100/1); White solid (59.3 mg, 63%)

^{1}H NMR (600 MHz, Acetone-d_6) δ 8.39 (d, *J* = 1.7 Hz, 1H), 8.10 (dd, *J* = 8.7, 1.7 Hz, 1H), 7.92 (d, *J* = 7.9 Hz, 2H), 7.83 (d, *J* = 2.2 Hz, 1H), 7.42 (d, *J* = 8.6 Hz, 1H), 7.21 (d, *J* = 7.9 Hz, 2H), 6.90 (d, *J* = 2.2 Hz, 1H), 2.34 (s, 3H). ^{13}C NMR (151 MHz, Acetone-d_6) δ 171.0, 156.6, 146.1, 142.3, 141.1, 133.0, 128.6, 127.0, 126.8, 125.6, 122.6, 109.9, 107.1, 20.4. HRMS (ESI-TOF) m/z: calcd for $[C_{16}H_{13}NNaO_4S]^+$ ($[M+Na]^+$): 338.0457, found 338.0446.

***N*-tosylthiophene-2-carboxamide (3m)**

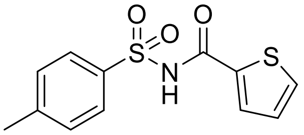

Purification by column chromatography (DCM/MeOH = 100/1); White solid (44.0 mg, 52%)

^{1}H NMR (600 MHz, Acetone-*d*6) δ 7.71 (d, *J* = 7.9 Hz, 2H), 7.39 (d, *J* = 3.6 Hz, 1H), 7.29 (d, *J* = 5.0 Hz, 1H), 7.07 (d, *J* = 7.8 Hz, 2H), 6.84 (t, *J* = 4.2 Hz, 1H), 2.21 (s, 3H). ^{13}C NMR (151 MHz, Acetone-*d*6) δ 167.4, 145.4, 143.2, 140.5, 129.2, 129.2, 128.4, 126.9, 126.5, 20.4. HRMS (ESI-TOF) m/z: calcd for $[C_{12}H_{11}NNaO_3S_2]^+$ ($[M+Na]^+$): 304.0073, found 304.0083.

5-methyl-*N*-tosylisoxazole-3-carboxamide (3n)

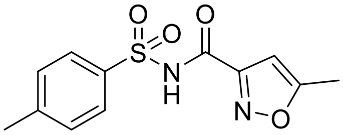

Purification by column chromatography (DCM/MeOH = 100/1); White solid (35.1 mg, 42%)

^{1}H NMR (600 MHz, Acetone-*d*6) δ 7.84 (d, *J* = 7.8 Hz, 2H), 7.19 (d, *J* = 7.8 Hz, 2H), 6.38 (s, 1H), 2.41 (s, 3H), 2.35 (s, 3H). ^{13}C NMR (151 MHz, Acetone-*d*6) δ 171.8, 142.4, 140.0, 137.5, 129.0, 126.7, 101.7, 83.0, 20.5, 11.3. HRMS (ESI-TOF) m/z: calcd for $[C_{12}H_{12}N_2NaO_4S]^+$ ($[M+Na]^+$): 303.0410, found 303.0417.

benzyl tosylcarbamate (3o)

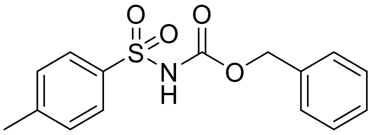

Purification by column chromatography (DCM/MeOH = 100/1); White solid (50.1 mg, 55%)

^{1}H NMR (600 MHz, Acetone-*d*6) δ 7.75 (d, *J* = 8.1 Hz, 2H), 7.27 (d, *J* = 8.0 Hz, 2H), 7.23 – 7.18 (m, 3H), 7.17 (dd, *J* = 7.6, 2.0 Hz, 2H), 4.94 (s, 2H), 2.30 (s, 3H). ^{13}C NMR (151 MHz, Acetone-*d*6) δ 151.4, 144.4, 137.2, 135.8, 129.4, 128.4, 128.2, 128.1, 128.0, 67.3, 20.6. HRMS (ESI-TOF) m/z: calcd for $[C_{15}H_{15}NNaO_4S]^+$ ($[M+Na]^+$): 328.0614, found 328.0627.

(9H-fluoren-9-yl)methyl tosylcarbamate (3p)

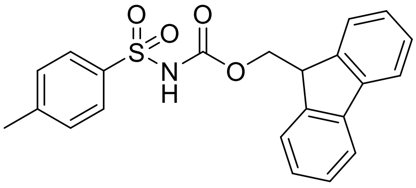

Purification by column chromatography (DCM/MeOH = 100/1); White solid (75.2 mg, 64%)

^{1}H NMR (600 MHz, Acetone-d_6) δ 7.72 (dd, J = 8.1, 1.9 Hz, 4H), 7.58 – 7.55 (m, 2H), 7.27 (t, J = 7.5 Hz, 2H), 7.17 (td, J = 7.5, 1.1 Hz, 2H), 7.08 (d, J = 7.9 Hz, 2H), 4.08 (t, J = 7.6 Hz, 1H), 3.98 (d, J = 7.6 Hz, 2H), 2.23 (s, 3H). ^{13}C NMR (151 MHz, Acetone-d_6) δ 160.6, 144.9, 143.6, 141.1, 140.0, 128.3, 127.3, 126.9, 126.5, 125.5, 119.7, 66.6, 47.3, 20.4. HRMS (ESI-TOF) m/z: calcd for $[C_{22}H_{19}NNaO_4S]^+$ ($[M+Na]^+$): 416.0927, found 416.0936.

***tert*-butyl tosylcarbamate (3q)**

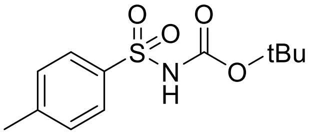

Purification by column chromatography (DCM/MeOH = 100/1); White solid (38.2 mg, 47%)

^{1}H NMR (600 MHz, Acetone-d_6) δ 7.90 (d, *J* = 8.0 Hz, 2H), 7.46 (d, *J* = 8.0 Hz, 2H), 2.46 (s, 3H), 1.36 (s, 9H). ^{13}C NMR (151 MHz, Acetone-d_6) δ 149.7, 144.3, 137.3, 129.4, 127.9, 82.2, 27.1, 20.6. HRMS (ESI-TOF) m/z: calcd for $[C_{12}H_{17}NNaO_4S]^+$ ($[M+Na]^+$): 294.0770, found 294.0779.

4-(tert-butyl)-*N*-((4-chlorophenyl)sulfonyl)benzamide (3r)

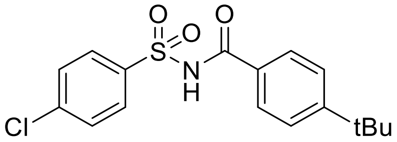

Purification by column chromatography (DCM/MeOH = 100/1); White solid (81.0 mg, 77%)

^{1}H NMR (600 MHz, Acetone-d_6) δ 12.57 (s, 1H), 8.00 (d, J = 8.2 Hz, 2H), 7.81 (d, J = 8.1 Hz, 2H), 7.73 (d, J = 8.3 Hz, 2H), 7.51 (d, J = 8.1 Hz, 2H), 1.27 (s, 9H). ^{13}C NMR (151 MHz, DMSO-d_6) δ 165.3, 156.6, 138.6, 138.4, 129.7, 129.3, 128.6, 128.4, 125.5, 34.9, 30.8. HRMS (ESI-TOF) m/z: calcd for $[C_{17}H_{18}ClNNaO_3S]^+$ ($[M+Na]^+$): 374.0588, found 374.0599.

N-((4-bromophenyl)sulfonyl)-4-(tert-butyl)benzamide (3s)

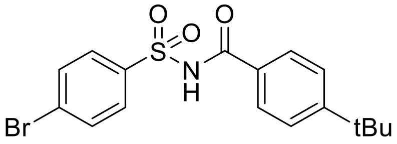

Purification by column chromatography (DCM/MeOH = 100/1); White solid (81.2 mg, 68%)

^{1}H NMR (600 MHz, Acetone-*d*$_6$) δ 7.80 (d, J = 8.4 Hz, 2H), 7.75 – 7.69 (m, 2H), 7.60 – 7.54 (m, 2H), 7.31 (d, J = 8.4 Hz, 2H), 1.26 (s, 9H). ^{13}C NMR (151 MHz, DMSO-*d*$_6$) δ 169.9, 152.5, 145.8, 136.4, 130.5, 129.1, 128.2, 124.1, 123.0, 34.4, 31.1. HRMS (ESI-TOF) m/z: calcd for $[C_{17}H_{18}BrNNaO_3S]^+$ ($[M+Na]^+$): 418.0083, found 418.0092.

4-(*tert*-butyl)-*N*-((4-methoxyphenyl)sulfonyl)benzamide (3t)

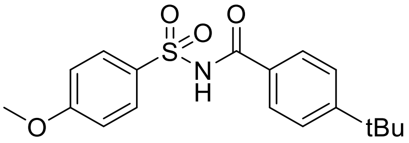

Purification by column chromatography (DCM/MeOH = 100/1); White solid (60.0 mg, 58%)

^{1}H NMR (600 MHz, Acetone-*d*$_6$) δ 8.06 (d, J = 8.8 Hz, 2H), 7.89 (d, J = 8.5 Hz, 2H), 7.55 (d, J = 8.5 Hz, 2H), 7.13 (d, J = 9.0 Hz, 2H), 3.92 (s, 3H), 1.34 – 1.32 (m, 9H). ^{13}C NMR (151 MHz, Acetone-*d*$_6$) δ 164.5, 163.7, 156.6, 131.4, 130.7, 129.3, 128.0, 125.6, 113.9, 55.3, 34.7, 30.4. HRMS (ESI-TOF) m/z: calcd for $[C_{18}H_{21}NNaO_4S]^+$ ($[M+Na]^+$): 370.1083, found 370.1091.

4-(*tert*-butyl)-*N*-(quinolin-8-ylsulfonyl)benzamide (3u)

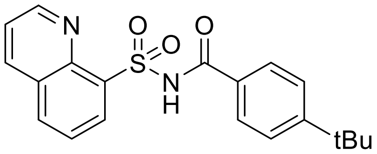

Purification by column chromatography (DCM/MeOH = 100/1); Yellow solid (38.0 mg, 34%)

^{1}H NMR (600 MHz, Acetone-*d*$_6$) δ 9.04 (dd, *J* = 4.3, 1.7 Hz, 1H), 8.50 (dd, *J* = 8.4, 1.7 Hz, 1H), 8.41 (dd, *J* = 7.3, 1.4 Hz, 1H), 8.30 (dd, *J* = 8.3, 1.4 Hz, 1H), 7.75 (dd, *J* = 8.3, 7.3 Hz, 1H), 7.69 (dd, *J* = 8.4, 4.3 Hz, 1H), 7.51 – 7.46 (m, 2H), 7.34 – 7.30 (m, 2H), 1.13 (s, 9H). ^{13}C NMR (151 MHz, Acetone-*d*$_6$) δ 163.1, 157.9, 151.8, 142.7, 137.7, 135.3, 133.2, 132.7, 129.2, 128.9, 126.1, 125.8, 124.0, 123.1, 34.8, 30.2. HRMS (ESI-TOF) m/z: calcd for $[C_{20}H_{20}N_2NaO_3S]^+$ ($[M+Na]^+$): 391.1087, found 391.1076.

4-(*tert*-butyl)-*N*-((3,5-dimethylisoxazol-4-yl)sulfonyl)benzamide (3v)

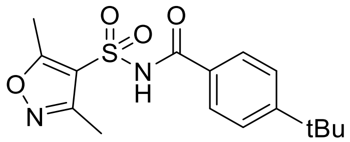

Purification by column chromatography (DCM/MeOH = 100/1); White solid (51.5 mg, 51%)

^{1}H NMR (600 MHz, Acetone-d_6) δ 7.77 (d, J = 8.5 Hz, 2H), 7.43 (d, J = 8.6 Hz, 2H), 2.63 (s, 3H), 2.29 (s, 3H), 1.20 (s, 9H). ^{13}C NMR (151 MHz, Acetone-d_6) δ 175.7, 165.6, 157.9, 156.9, 129.1, 128.2, 125.6, 115.5, 34.8, 30.4, 12.2, 9.9. HRMS (ESI-TOF) m/z: calcd for $[C_{16}H_{20}N_2NaO_4S]^+$ ($[M+Na]^+$): 359.1036, found 359.1043.

4-(*tert*-butyl)-*N*-(ethylsulfonyl)benzamide (3w)

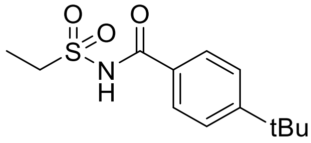

Purification by column chromatography (DCM/MeOH = 100/1); White solid (78.1 mg, 97%)

^{1}H NMR (600 MHz, Acetone-d_6) δ 7.98 (d, J = 8.4 Hz, 2H), 7.60 (d, J = 8.4 Hz, 2H), 3.59 – 3.55 (m, 2H), 1.36 (d, J = 2.1 Hz, 12H). ^{13}C NMR (151 MHz, Acetone-d_6) δ 165.8, 156.8, 129.3, 128.2, 125.6, 47.3, 34.8, 30.4, 7.4. HRMS (ESI-TOF) m/z: calcd for $[C_{13}H_{19}NNaO_3S]^+$ ($[M+Na]^+$): 292.0978, found 292.0986.

methyl 3-(*N*-(4-(*tert*-butyl)benzoyl)sulfamoyl)propanoate (3x)

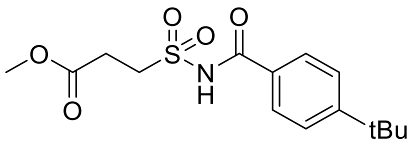

Purification by column chromatography (DCM/MeOH = 100/1); White solid (79.5 mg, 81%)

^{1}H NMR (600 MHz, Acetone-d_6) δ 8.00 (d, J = 8.4 Hz, 2H), 7.52 (d, J = 7.9 Hz, 2H), 3.69 (s, 2H), 3.63 (s, 3H), 2.89 (t, J = 7.4 Hz, 3H), 1.35 (s, 9H). ^{13}C NMR (151 MHz, Acetone-d_6) δ 170.9, 129.5, 128.6, 127.4, 125.1, 51.4, 48.2, 34.7, 30.5, 28.3. HRMS (ESI-TOF) m/z: calcd for $[C_{15}H_{21}NNaO_5S]^+$ ($[M+Na]^+$): 350.1033, found 350.1044.

4-(*tert*-butyl)-*N*-(cyclopropylsulfonyl)benzamide (3y)

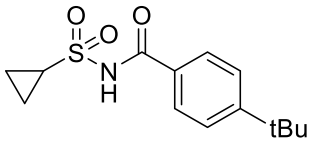

Purification by column chromatography (DCM/MeOH = 100/1); White solid (78.0 mg, 92%)

^{1}H NMR (600 MHz, Acetone-d_6) δ 7.97 (d, J = 8.2 Hz, 2H), 7.60 (d, J = 8.2 Hz, 2H), 3.19 (td, J = 8.1, 4.1 Hz, 1H), 1.36 (s, 9H), 1.26 (dd, J = 4.8, 2.6 Hz, 2H), 1.15 (dd, J = 8.0, 2.8 Hz, 2H). ^{13}C NMR (151 MHz, Acetone-d_6) δ 165.5, 156.7, 129.4, 128.2, 125.6, 34.8, 30.9, 30.4, 5.3. HRMS (ESI-TOF) m/z: calcd for $[C_{14}H_{19}NNaO_3S]^+$ ($[M+Na]^+$): 304.0978, found 304.0989.

4-(*tert*-butyl)-*N*-((difluoromethyl)sulfonyl)benzamide (3z)

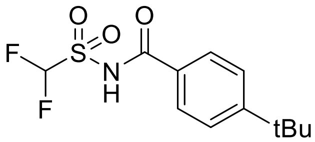

Purification by column chromatography (DCM/MeOH = 100/1); White solid (38.0 mg, 43%)

^{1}H NMR (600 MHz, Acetone-d_6) δ 7.86 (d, J = 8.4 Hz, 2H), 7.28 (d, J = 8.4 Hz, 2H), 6.48 (t, J = 55.0 Hz, 1H), 1.19 (s, 9H). ^{13}C NMR (151 MHz, Acetone-d_6) δ 172.2, 154.2, 134.7, 128.9, 124.5, 114.28 (*t*, J = 277.2 Hz). 34.5, 30.6. ^{19}F NMR (565 MHz, Acetone-d_6) δ -126.2. HRMS (ESI-TOF) m/z: calcd for $[C_{12}H_{15}F_2NNaO_3S]^+$ ($[M+Na]^+$): 314.0633, found 314.0639.

4-(*tert*-butyl)-*N*-(((7,7-dimethyl-2-oxobicyclo[2.2.1]heptan-1-yl)methyl)sulfonyl)benzamide (3aa)

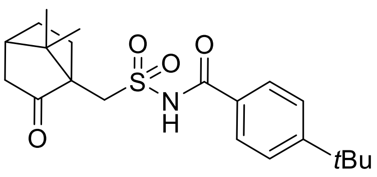

Purification by column chromatography (DCM/MeOH = 100/1); White solid (48.1 mg, 41%)

^{1}H NMR (600 MHz, DMSO-d_6) δ 12.01 (s, 1H), 7.91 – 7.86 (m, 2H), 7.55 – 7.52 (m, 2H), 3.67 (dd, J = 251.4, 15.2 Hz, 2H), 2.39 – 2.32 (m, 2H), 2.07 (t, J = 4.5 Hz, 1H), 1.99 – 1.89 (m, 2H), 1.60 (ddd, J = 13.9, 9.4, 4.7 Hz, 1H), 1.42 (ddd, J = 12.8, 9.3, 3.9 Hz, 1H), 1.31 (s, 9H), 1.02 (s, 3H), 0.82 (s, 3H). ^{13}C NMR (151 MHz, DMSO-d_6) δ 214.5, 166.7, 156.6, 128.9, 125.9, 58.5, 50.0, 48.3, 42.5, 42.4, 35.3, 31.3, 26.8, 24.9, 19.8, 19.8. HRMS (ESI-TOF) m/z: calcd for $[C_{21}H_{29}NNaO_4S]^+$ ($[M+Na]^+$): 414.1710, found 414.1723.

N-(allylsulfonyl)-4-(tert-butyl)benzamide (3ab)

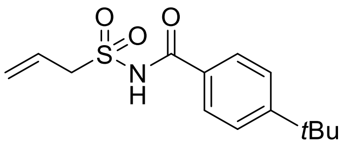

Purification by column chromatography (DCM/MeOH = 100/1); Colorless oil (64.0 mg, 78%)

^{1}H NMR (600 MHz, Acetone-d_6) δ 7.83 (d, J = 8.5 Hz, 2H), 7.42 (d, J = 8.5 Hz, 2H), 5.83 – 5.75 (m, 1H), 5.29 – 5.21 (m, 2H), 4.11 (d, J = 7.3 Hz, 2H), 1.20 (s, 9H). ^{13}C NMR (151 MHz, Acetone-d_6) δ 167.1, 167.0, 156.5, 130.0, 128.3, 126.2, 125.5, 123.1, 56.8, 34.8, 30.5. HRMS (ESI-TOF) m/z: calcd for $[C_{14}H_{19}NNaO_3S]^+$ ($[M+Na]^+$): 304.0978, found 304.0990.

4-(tert-butyl)-N-((trifluoromethyl)sulfonyl)benzamide (3ac)

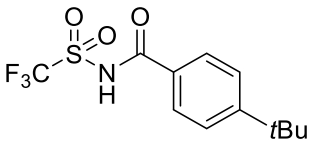

Purification by column chromatography (DCM/MeOH = 100/1); Colorless crystal (44.8 mg, 48%)

^{1}H NMR (600 MHz, Acetone-d_6) δ 8.02 (d, J = 8.5 Hz, 2H), 7.44 (d, J = 8.4 Hz, 2H), 1.33 (s, 9H). ^{13}C NMR (151 MHz, Acetone-d_6) δ 172.8, 154.4, 135.0, 129.0, 124.5, 120.87 (q, J = 323.0 Hz), 34.5, 30.6. ^{19}F NMR (565 MHz, Acetone-d_6) δ -79.8. HRMS (ESI-TOF) m/z: calcd for $[C_{12}H_{14}F_3NNaO_3S]^+$ ($[M+Na]^+$): 332.0539, found 332.0533.

4-(tert-butyl)-N-((4-cyanophenyl)sulfonyl)benzamide (3ad)

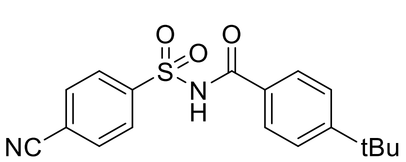

Purification by column chromatography (DCM/MeOH = 100/1); White solid (66.0 mg, 64%)

^{1}H NMR (400 MHz, Acetone-d_6) δ 8.16 (d, J = 8.7 Hz, 2H), 7.94 (d, J = 8.7 Hz, 2H), 7.75 (d, J = 8.6 Hz, 2H), 7.42 (d, J = 8.7 Hz, 2H), 1.18 (s, 9H). ^{13}C NMR (101 MHz, Acetone-d_6) δ 165.8, 158.0, 144.7, 133.8, 130.0, 129.6, 129.2, 126.6, 118.1, 117.8, 35.7, 31.3. HRMS (ESI-TOF) m/z: calcd for $[C_{18}H_{18}N_2NaO_3S]^+$ ($[M+Na]^+$): 365.0930, found 365.0938.

B. Cyanoarene-based Photocatalyst

2,3,5,6-tetra(9H-carbazol-9-yl)benzonitrile (4CzBN)

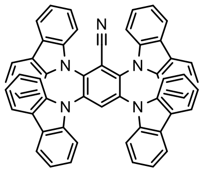

^{1}H NMR (600 MHz, $CDCl_3$) δ 8.42 (s, 1H), 7.80 – 7.75 (m, 8H), 7.35 (d, *J* = 8.1 Hz, 4H), 7.32 – 7.29 (m, 4H), 7.19 – 7.16 (m, 4H), 7.14 – 7.08 (m, 12H). ^{13}C NMR (151 MHz, $CDCl_3$) δ 139.2, 138.8, 137.8, 136.9, 136.6, 125.7, 124.2, 123.9, 121.3, 121.0, 120.3, 120.2, 118.2, 113.0, 109.9, 109.2, 1.9. HRMS (ESI-TOF) m/z: calcd for $[C_{55}H_{33}N_5Na]^+$ ($[M+Na]^+$): 786.2628, found 786.2640.

C. Sodium Organosulfinate

Sodium (7,7-dimethyl-2-oxobicyclo[2.2.1]heptan-1-yl)methanesulfinate[4]

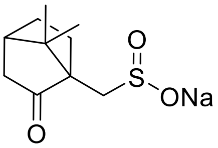

^{1}H NMR (600 MHz, DMSO-d_6) δ 3.16 (d, J = 4.2 Hz, 1H), 2.32 – 2.19 (m, 3H), 1.98 (t, J = 4.5 Hz, 1H), 1.86 (dt, J = 7.7, 3.8 Hz, 1H), 1.80 – 1.69 (m, 2H), 1.32 – 1.27 (m, 2H), 0.96 (s, 3H), 0.77 (s, 3H). HRMS (ESI-TOF) m/z: calcd for $[C_{10}H_{15}Na_2O_3S]^+$ ($[M+Na]^+$): 261.0532, found 261.0537.

D. Benzohydroxamic acids / Hydroxycarbamates

N-hydroxy-4-methoxybenzamide (1a)[5]

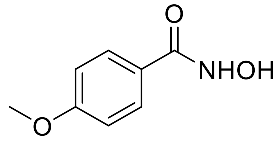

Method A; Purification by recrystallization; pale pink crystalline solid (2.08 g, 83%).

^{1}H NMR (600 MHz, Acetone-d_6) δ 10.68 (s, 1H), 8.40 (s, 1H), 7.83 (d, J = 8.8 Hz, 2H), 7.01 (d, J = 8.8 Hz, 2H), 3.87 (s, 3H). HRMS (ESI-TOF) m/z: calcd for $[C_8H_9NNaO_3]^+$ ($[M+Na]^+$): 190.0475, found 190.0478.

N-hydroxy-2-methoxybenzamide (1b)[6]

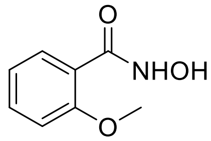

Method A; Purification by recrystallization; pale pink powder (1.81 g, 72%)

^{1}H NMR (400 MHz, DMSO-d_6) δ 10.63 (s, 1H), 9.09 (s, 1H), 7.57 (d, *J* = 9.4 Hz, 1H), 7.45 (t, *J* = 6.9 Hz, 1H), 7.11 (d, *J* = 8.3 Hz, 1H), 7.02 (t, *J* = 7.5 Hz, 1H), 3.84 (s, 3H). HRMS (ESI-TOF) m/z: calcd for $[C_8H_9NNaO_3]^+$ ($[M+Na]^+$): 190.0475, found 190.0476.

4-(*tert*-butyl)-N-hydroxybenzamide (1d)[5]

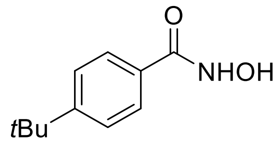

Method A; Purification by recrystallization; white crystalline solid (2.20 g, 76%)

^{1}H NMR (400 MHz, DMSO-d_6) δ 11.15 (s, 1H), 8.99 (s, 1H), 7.70 (d, *J* = 8.5 Hz, 2H), 7.47 (d, *J* = 8.5 Hz, 2H), 1.30 (s, 9H). HRMS (ESI-TOF) m/z: calcd for $[C_{11}H_{15}NNaO_2]^+$ ($[M+Na]^+$): 216.0995, found 216.1000.

N-hydroxy-4-(trifluoromethoxy)benzamide (1e)[7]

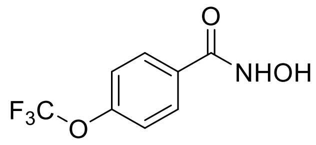

Method A; Purification by recrystallization; white crystalline solid (2.51 g, 76%)

^{1}H NMR (600 MHz, DMSO-d_6) δ 11.32 (s, 1H), 9.18 (s, 1H), 7.88 (d, J = 8.7 Hz, 2H), 7.46 (d, J = 9.1 Hz, 2H). HRMS (ESI-TOF) m/z: calcd for $[C_8H_6F_3NNaO_3]^+$ ($[M+Na]^+$): 244.0192, found 244.0193.

4-fluoro-*N*-hydroxybenzamide (1f)[5]

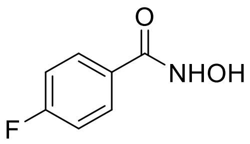

Method A; Purification by recrystallization; white crystalline solid (1.63 g, 70%)
^{1}H NMR (600 MHz, Acetone-d_6) δ 10.66 (s, 1H), 8.59 (s, 1H), 7.96 – 7.88 (m, 2H), 7.25 (t, *J* = 8.8 Hz, 2H). HRMS (ESI-TOF) m/z: calcd for $[C_7H_6FNNaO_2]^+$ ($[M+Na]^+$): 178.0275, found 178.0275.

4-ethynyl-*N*-hydroxybenzamide (1g)[8]

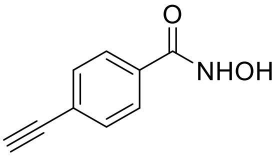

Method A; Purification by recrystallization; white solid (1.91 g, 79%)
^{1}H NMR (600 MHz, DMSO-d_6) δ 11.32 (s, 1H), 9.13 (s, 1H), 7.75 (d, *J* = 8.2 Hz, 2H), 7.56 (d, *J* = 8.2 Hz, 2H), 4.38 (s, 1H). HRMS (ESI-TOF) m/z: calcd for $[C_9H_7NNaO_2]^+$ ($[M+Na]^+$): 184.0369, found 184.0371.

***N*,4-dihydroxybenzamide (1h)[9]**

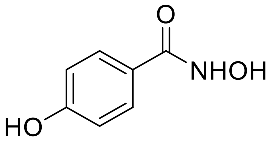

Method B; Purification by column chromatography (Hexane/EA); white solid (0.92 g, 40%)
^{1}H NMR (600 MHz, DMSO-d_6) δ 10.96 (s, 1H), 9.96 (s, 1H), 8.83 (s, 1H), 7.61 (d, *J* = 8.7 Hz, 2H), 6.78 (d, *J* = 8.6 Hz, 2H). HRMS (ESI-TOF) m/z: calcd for $[C_7H_7NNaO_3]^+$ ($[M+Na]^+$): 176.0318, found 176.0316.

3,5-dichloro-*N*-hydroxybenzamide (1j)[10]

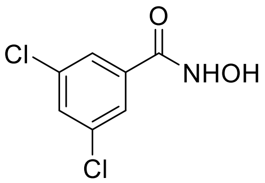

Method A; Purification by recrystallization; white crystalline solid (2.38 g, 77%)

^{1}H NMR (600 MHz, DMSO-d_6) δ 11.45 (s, 1H), 9.31 (s, 1H), 7.84 – 7.79 (m, 1H), 7.77 (d, *J* = 1.9 Hz, 2H). HRMS (ESI-TOF) m/z: calcd for $[C_7H_5Cl_2NNaO_2]^+$ ($[M+Na]^+$): 227.959, found 227.9586.

***N*-hydroxy-2-naphthamide (1k)[5]**

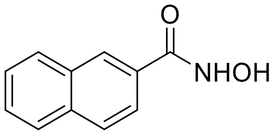

Method A; Purification by recrystallization; pale pink solid (2.36 g, 84%)

^{1}H NMR (400 MHz, DMSO-d_6) δ 11.38 (s, 1H), 9.14 (s, 1H), 8.37 (d, *J* = 1.8 Hz, 1H), 8.06 – 7.94 (m, 3H), 7.85 (dd, *J* = 8.5, 1.8 Hz, 1H), 7.61 (tt, *J* = 6.9, 5.1 Hz, 2H). HRMS (ESI-TOF) m/z: calcd for $[C_{11}H_9NNaO_2]^+$ ($[M+Na]^+$): 210.0525, found 210.0526.

***N*-hydroxybenzofuran-5-carboxamide (1l)**

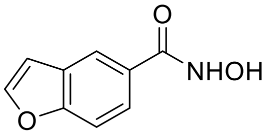

Method A; Purification by recrystallization; white solid (1.73 g, 65%)

^{1}H NMR (600 MHz, Acetone-d_6) δ 10.80 (s, 1H), 8.30 (s, 1H), 8.18 (d, *J* = 1.8 Hz, 1H), 7.95 (d, *J* = 2.2 Hz, 1H), 7.84 (dd, *J* = 8.6, 1.8 Hz, 1H), 7.63 (d, *J* = 8.6 Hz, 1H), 7.02 (dd, *J* = 2.2, 1.0 Hz, 1H). ^{13}C NMR (101 MHz, DMSO) δ 164.5, 155.6, 147.2, 127.9, 127.1, 123.4, 120.5, 111.1, 107.1. HRMS (ESI-TOF) m/z: calcd for $[C_9H_7NNaO_3]^+$ ($[M+Na]^+$): 200.0318, found 200.0322.

***N*-hydroxythiophene-2-carboxamide (1m)[6]**

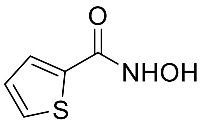

Method A; Purification by recrystallization; pale pink solid (1.40 g, 65%)

^{1}H NMR (600 MHz, DMSO-d_6) δ 11.23 (s, 1H), 9.14 (s, 1H), 7.76 (d, *J* = 5.0 Hz, 1H), 7.67 – 7.55 (m, 1H), 7.13 (t, *J* = 4.3 Hz, 1H). HRMS (ESI-TOF) m/z: calcd for $[C_5H_5NNaO_2S]^+$ ($[M+Na]^+$): 165.9933, found 165.9934.

N-hydroxy-5-methylisoxazole-3-carboxamide (1n)

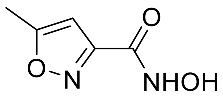

Method A; Purification by recrystallization; white solid (1.28 g, 60%)

^{1}H NMR (400 MHz, DMSO-d_6) δ 9.77 (s, 1H), 6.31 (d, J = 1.1 Hz, 1H), 2.40 (d, J = 0.9 Hz, 3H). ^{13}C NMR (101 MHz, DMSO) δ 169.0, 159.7, 157.0, 100.6, 11.7. HRMS (ESI-TOF) m/z: calcd for $[C_5H_6N_2NaO_3]^+$ ($[M+Na]^+$): 165.0271, found 165.0267.

(9H-fluoren-9-yl)methyl hydroxycarbamate (1p)[11]

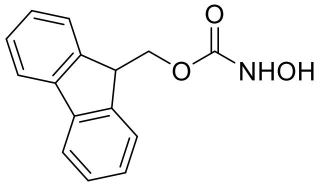

Method A; Purification by recrystallization; white crystalline solid (2.80 g, 73%)

^{1}H NMR (400 MHz, DMSO-d_6) δ 9.79 (s, 1H), 8.80 (s, 1H), 7.91 (d, J = 7.5 Hz, 2H), 7.71 (d, J = 7.4 Hz, 2H), 7.43 (td, J = 7.5, 1.2 Hz, 2H), 7.34 (td, J = 7.4, 1.2 Hz, 2H), 4.35 (d, J = 7.1 Hz, 2H), 4.24 (t, J = 7.0 Hz, 1H). HRMS (ESI-TOF) m/z: calcd for $[C_{15}H_{13}NNaO_3]^+$ ($[M+Na]^+$): 278.0788, found 278.0793.

NMR spectra

A. Acylsulfonamides

4-methoxy-*N*-tosylbenzamide (3a)

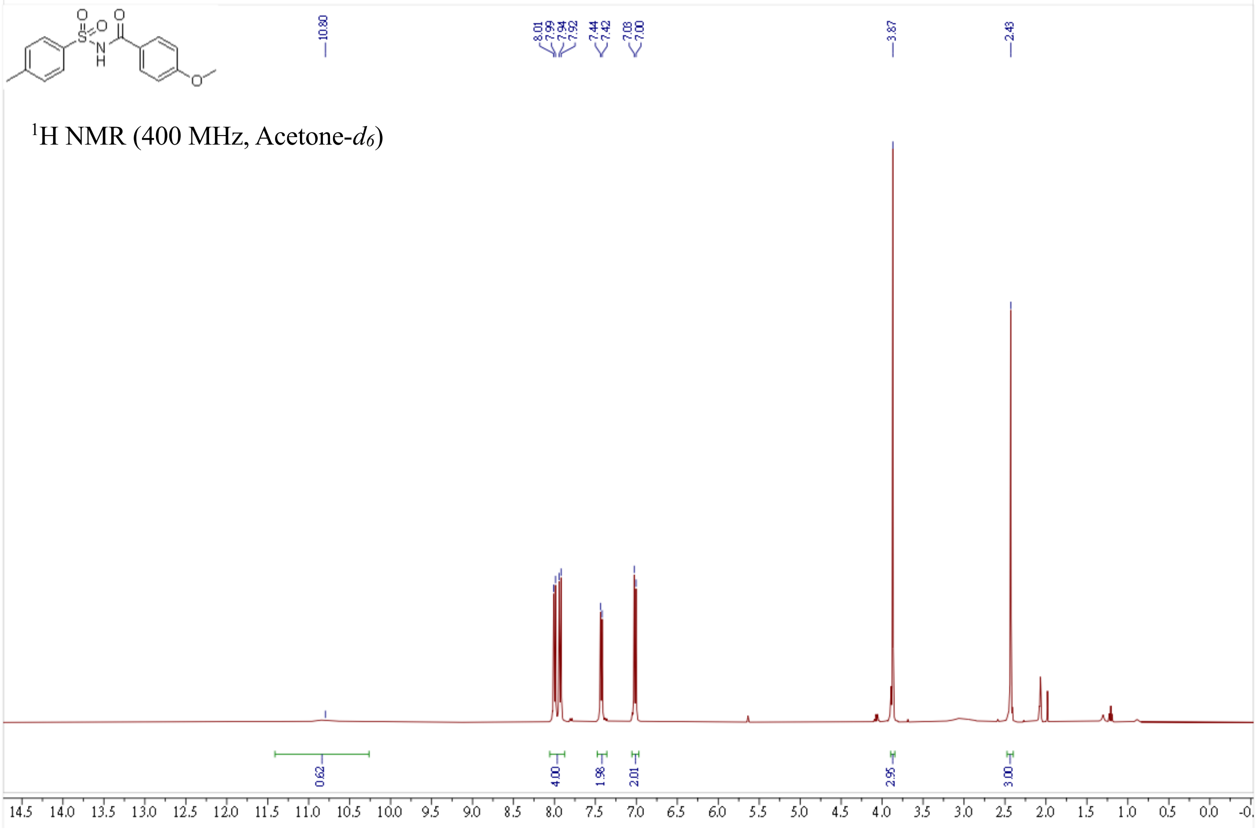

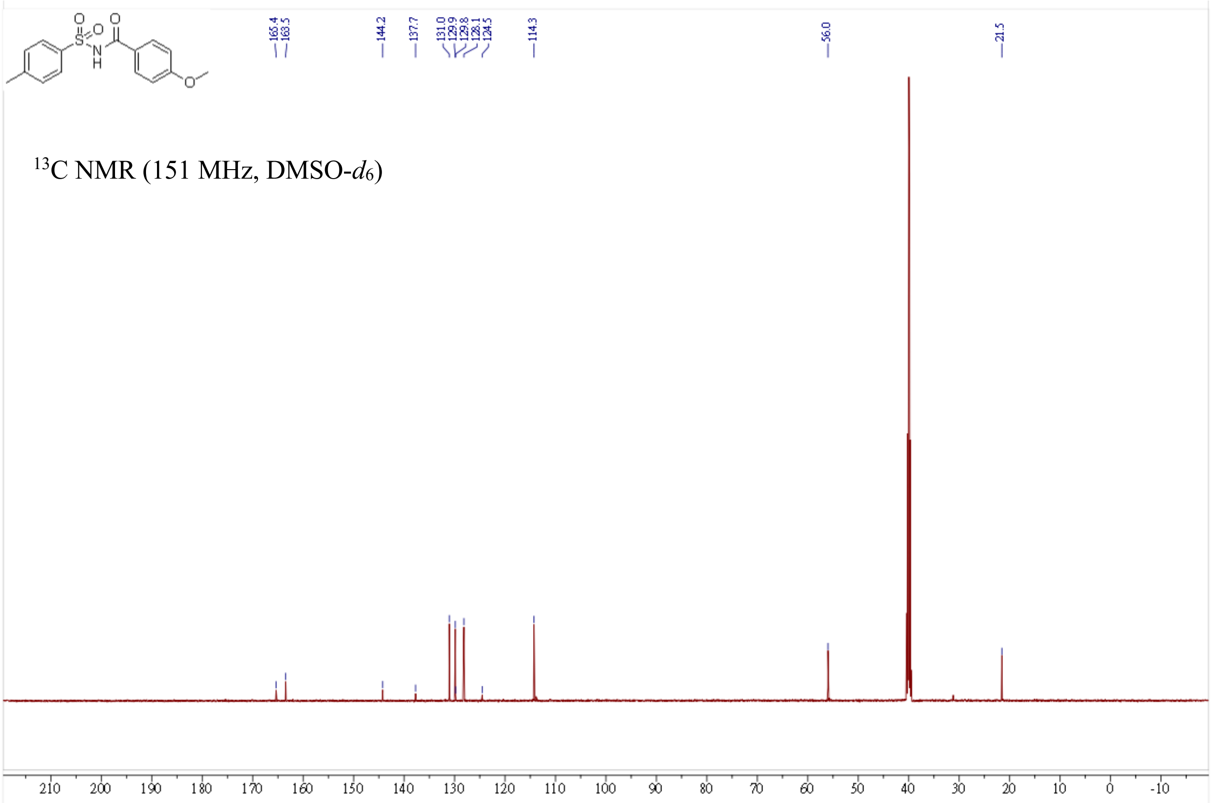

2-methoxy-*N*-tosylbenzamide (3b)

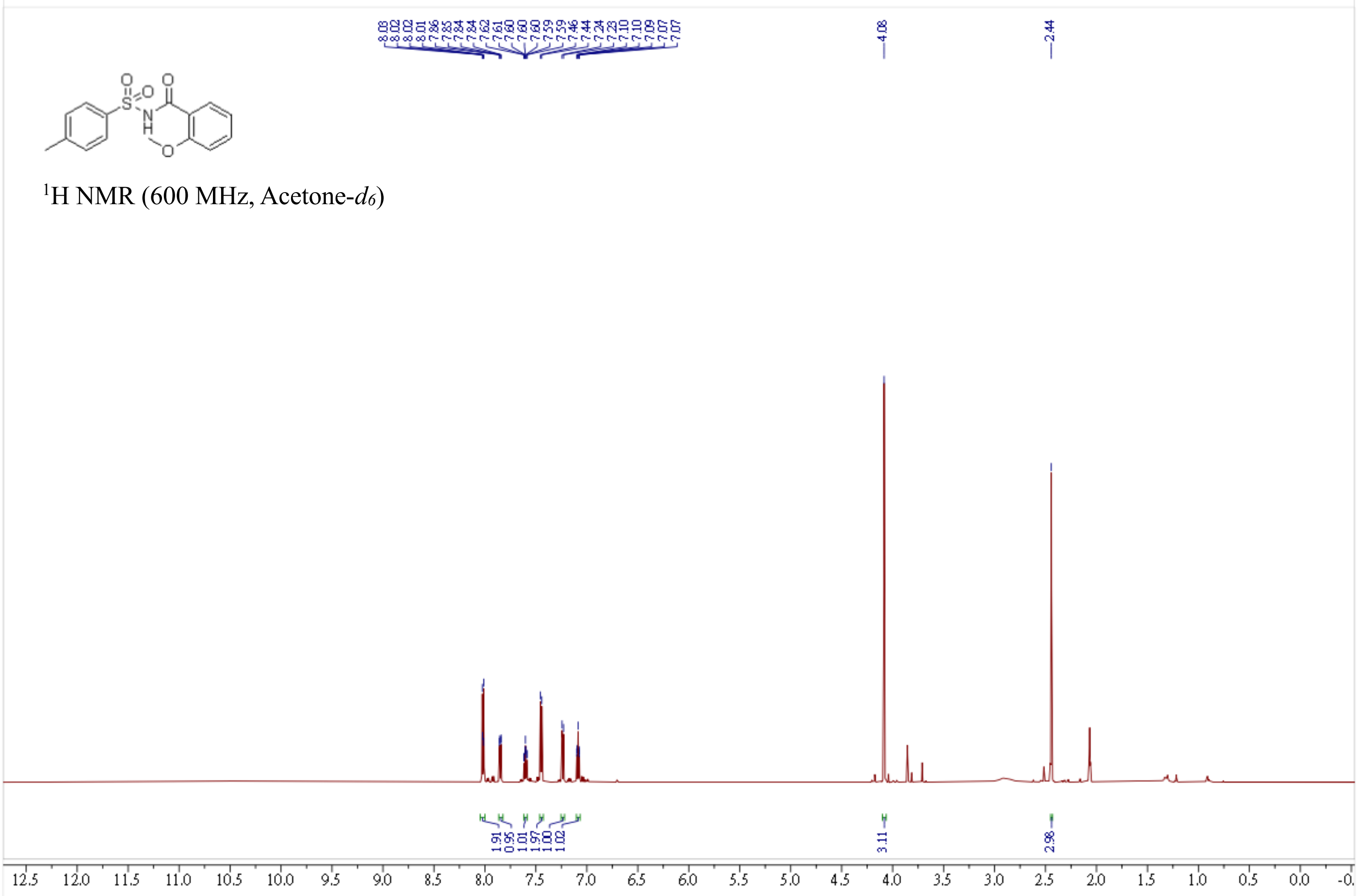

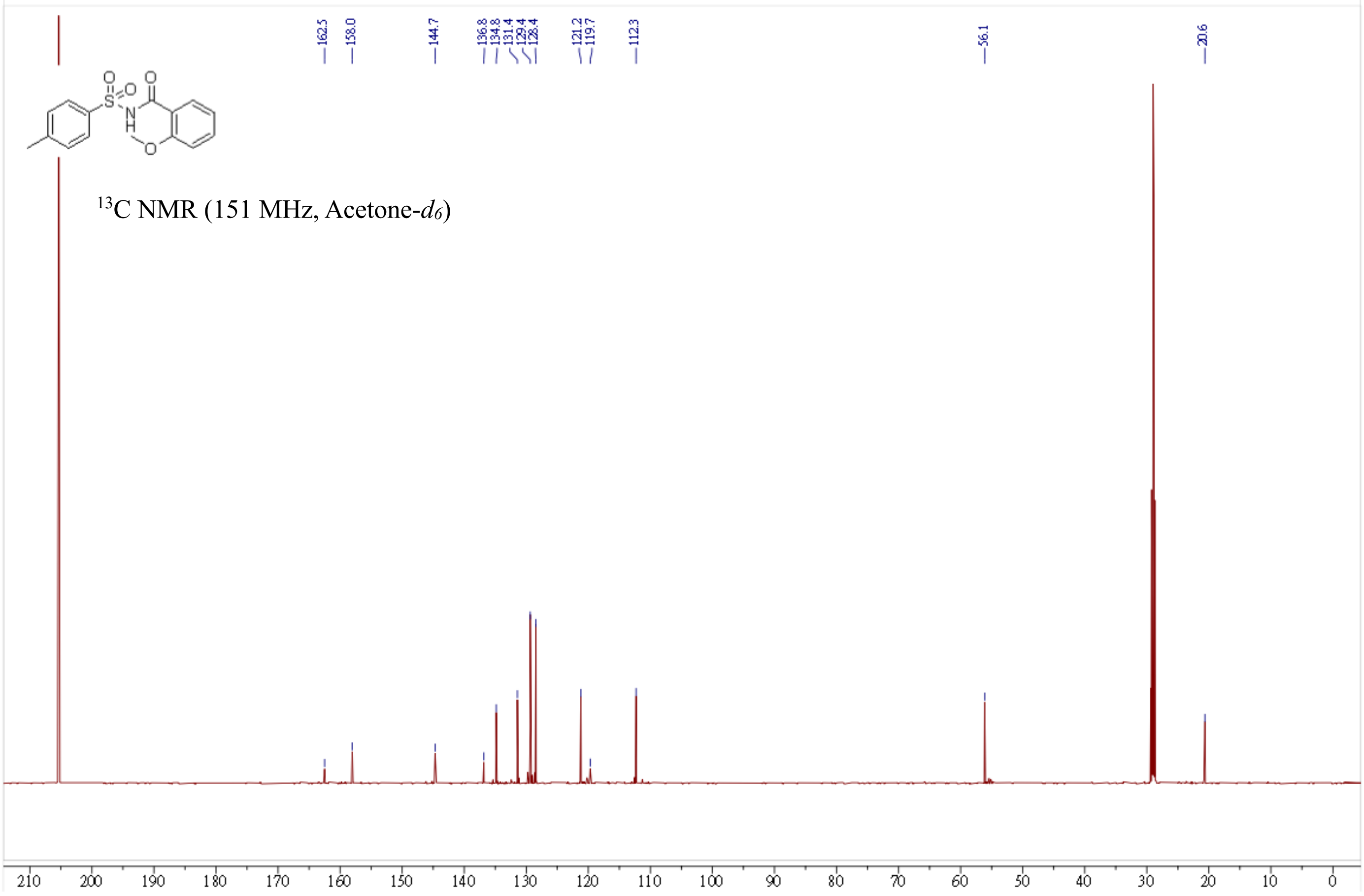

***N*-tosylbenzamide (3c)**

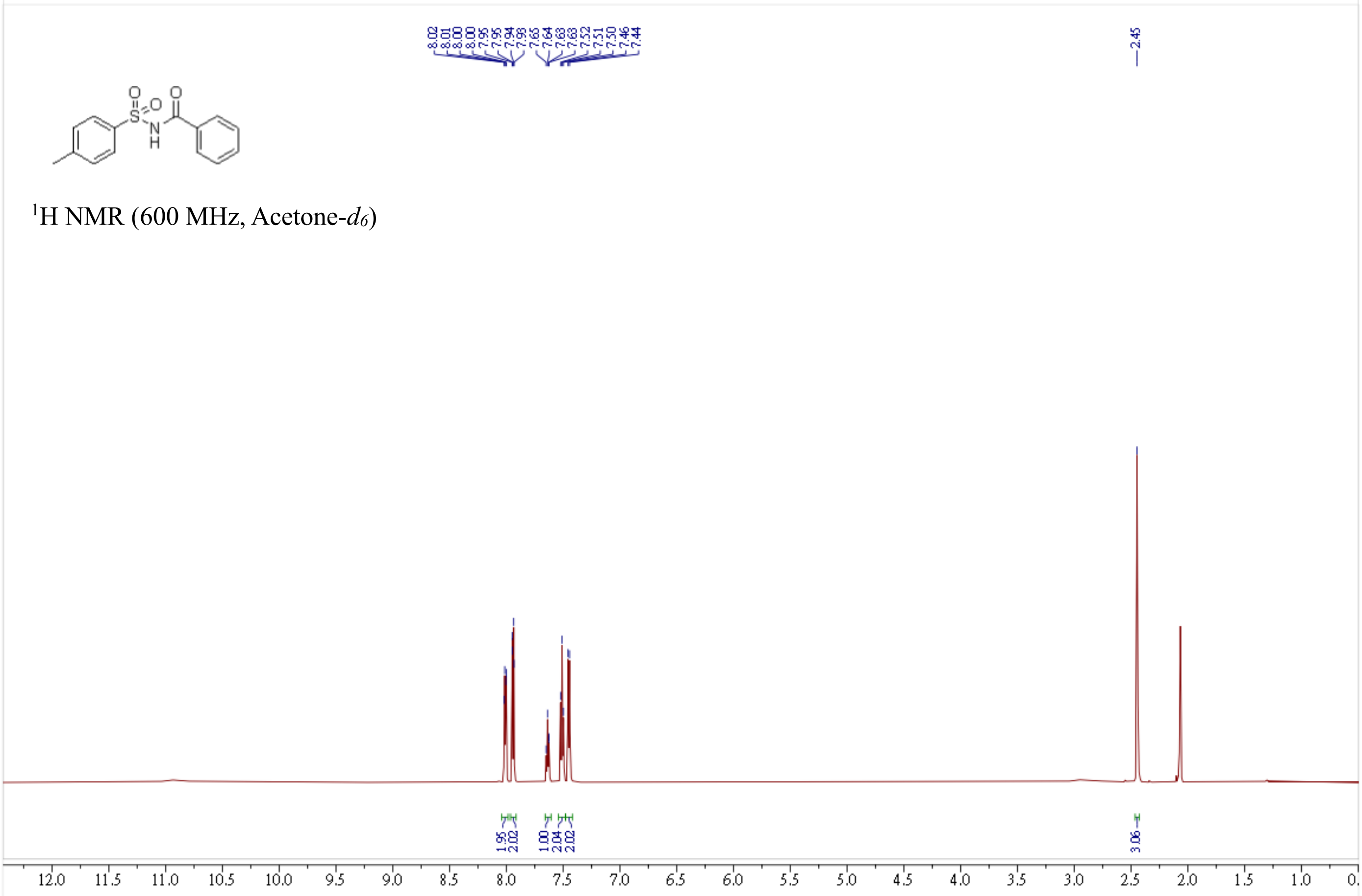

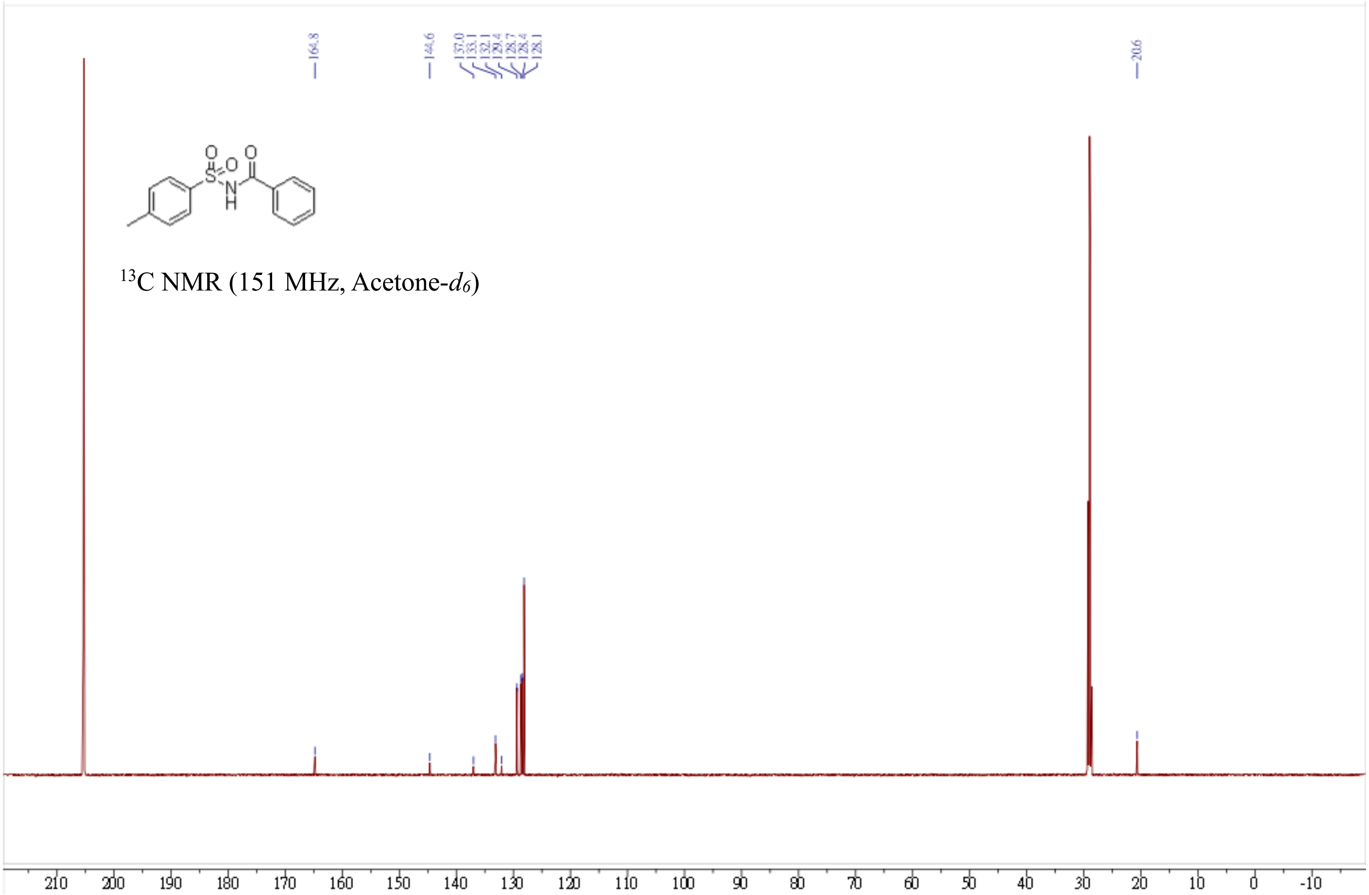

4-(tert-butyl)-*N*-tosylbenzamide (3d)

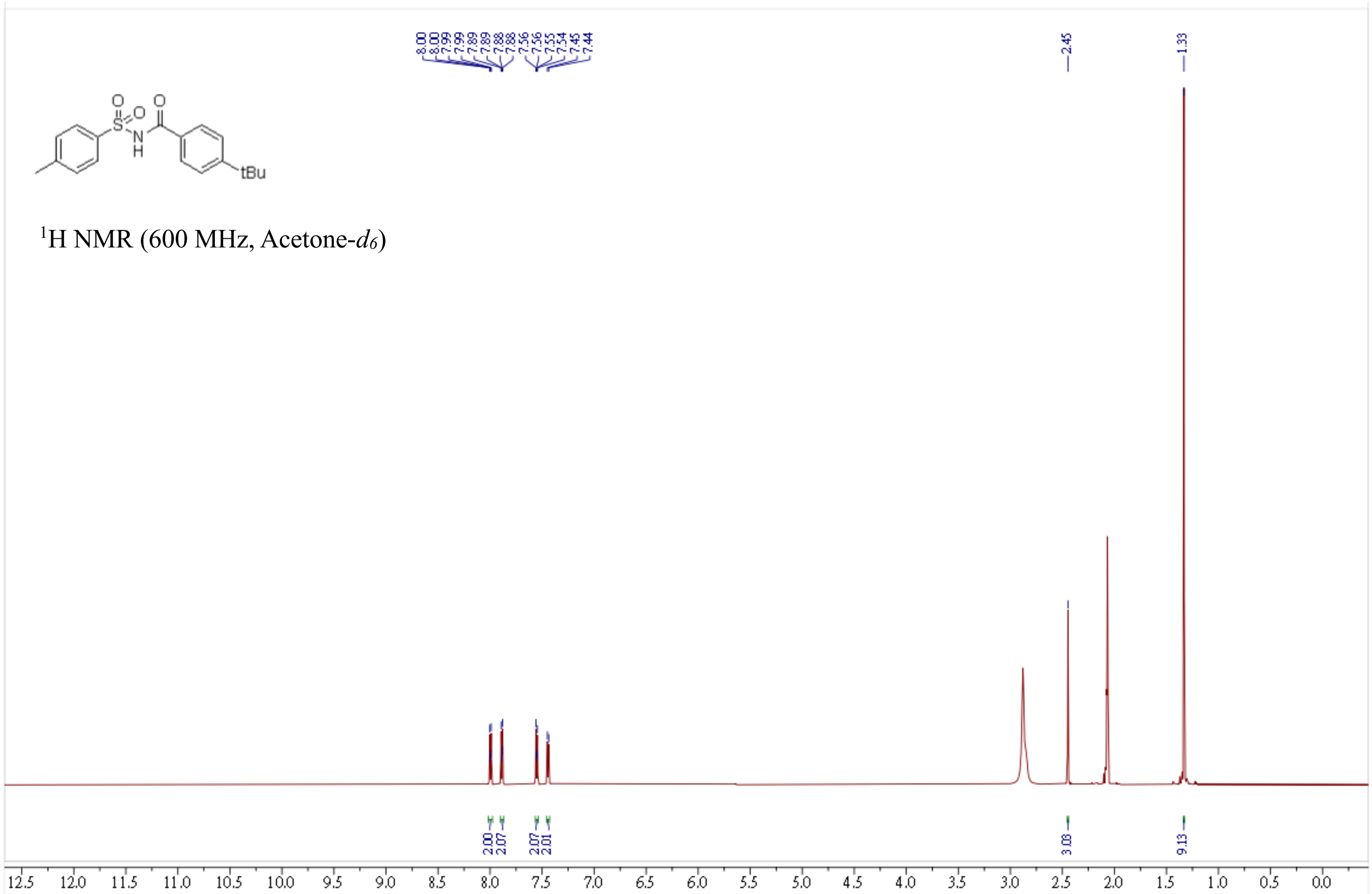

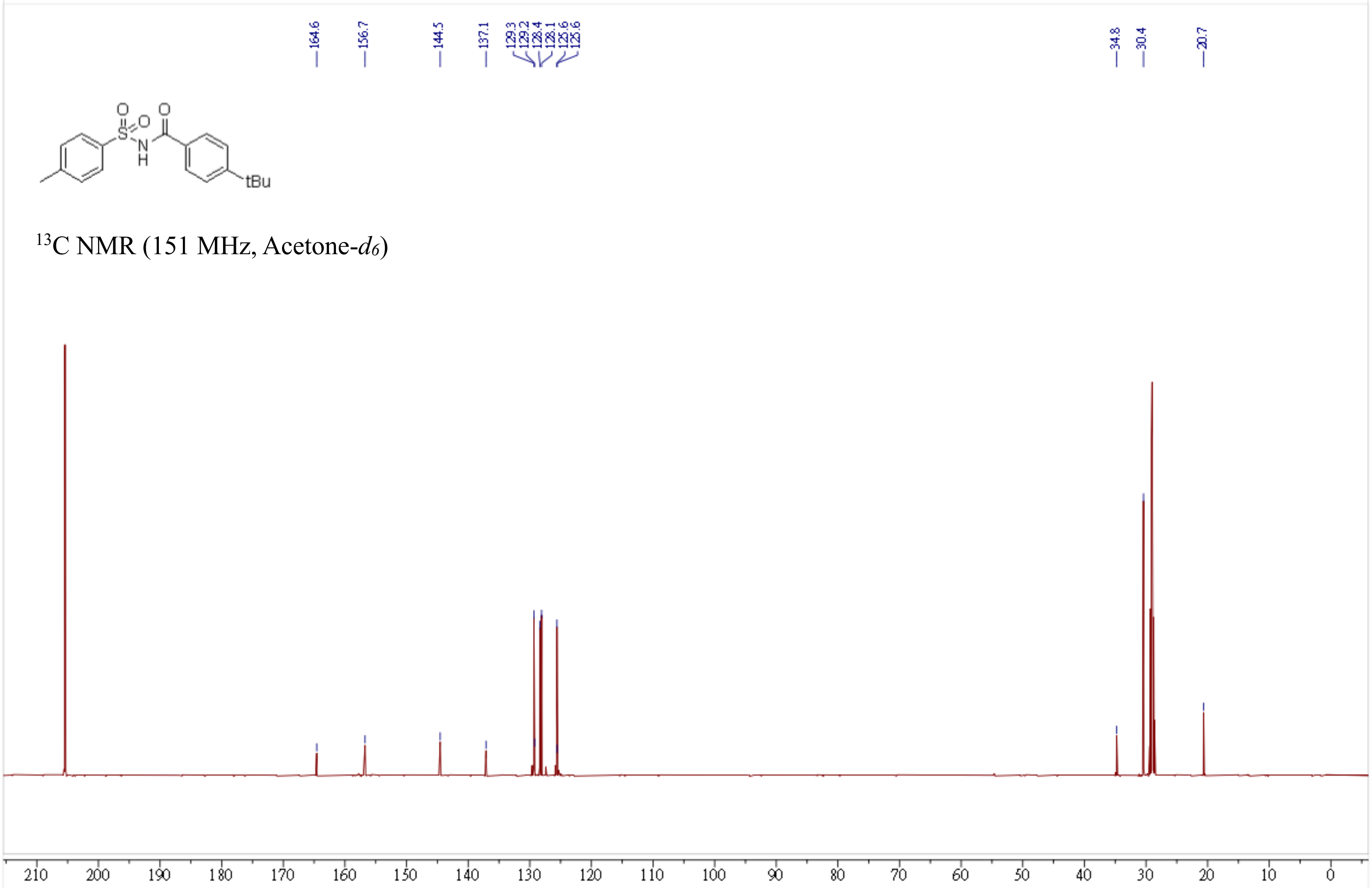

***N*-tosyl-4-(trifluoromethoxy)benzamide (3e)**

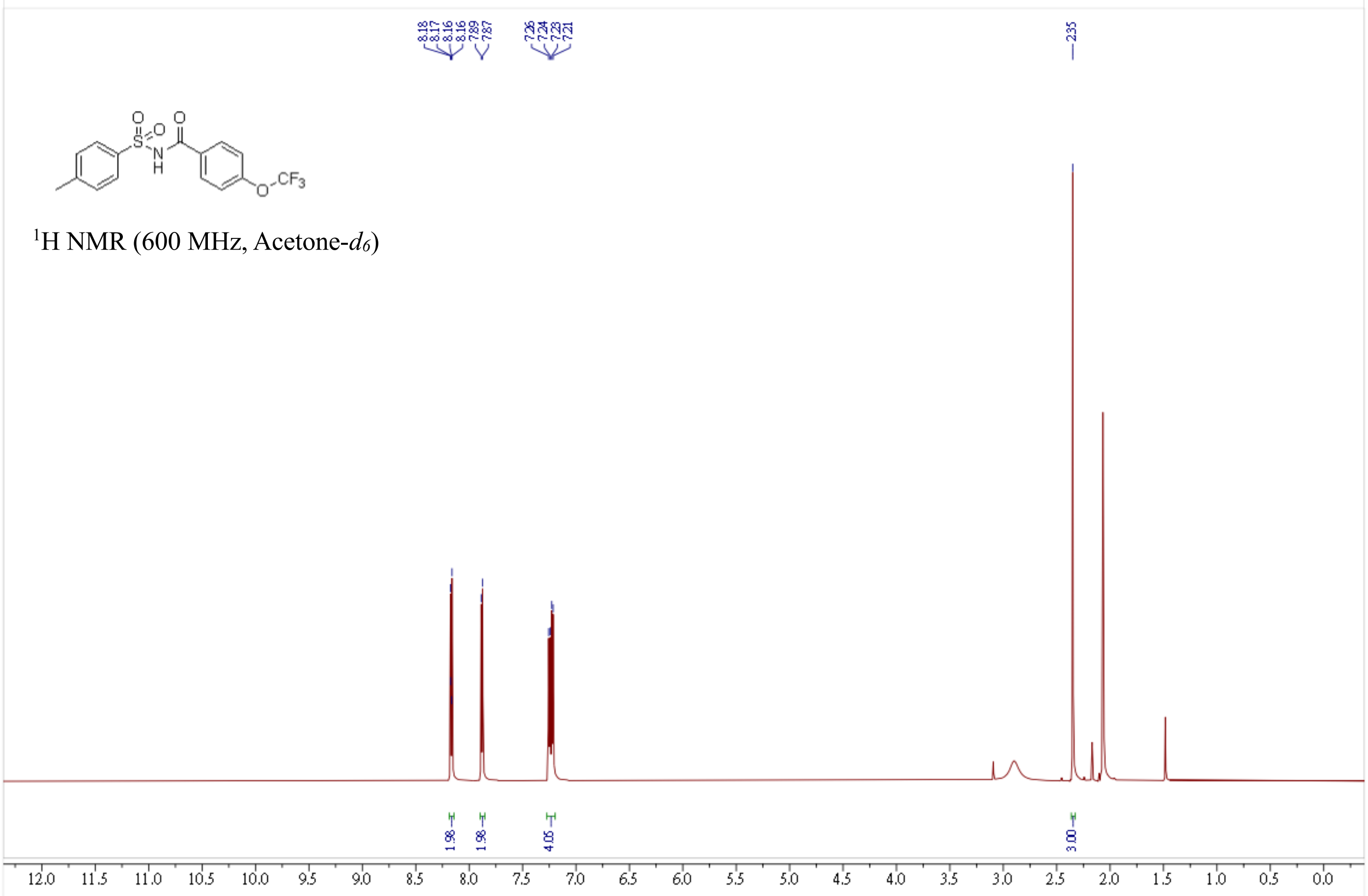

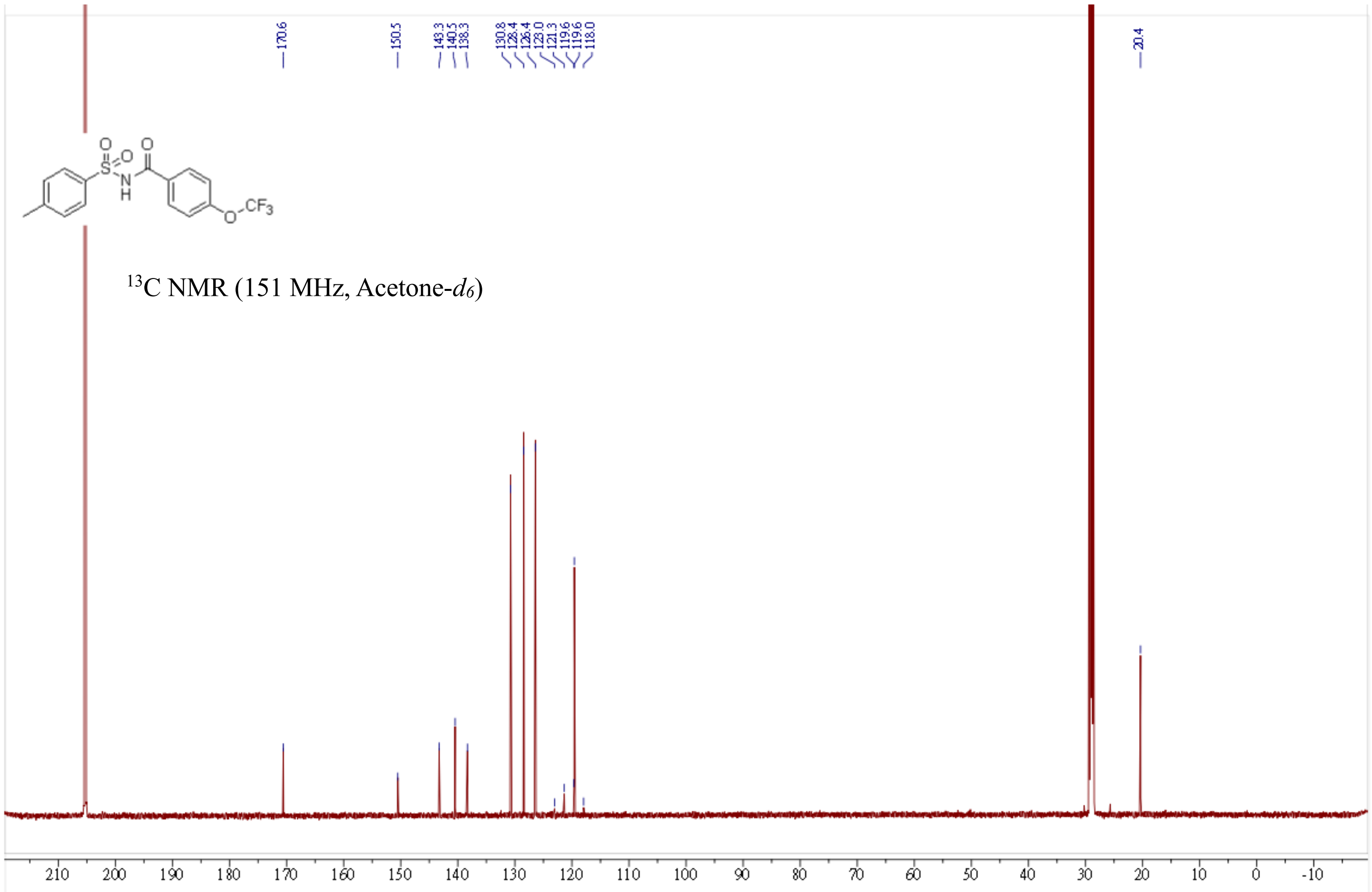

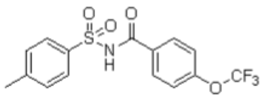

^{19}F NMR (565 MHz, Acetone-d_6)

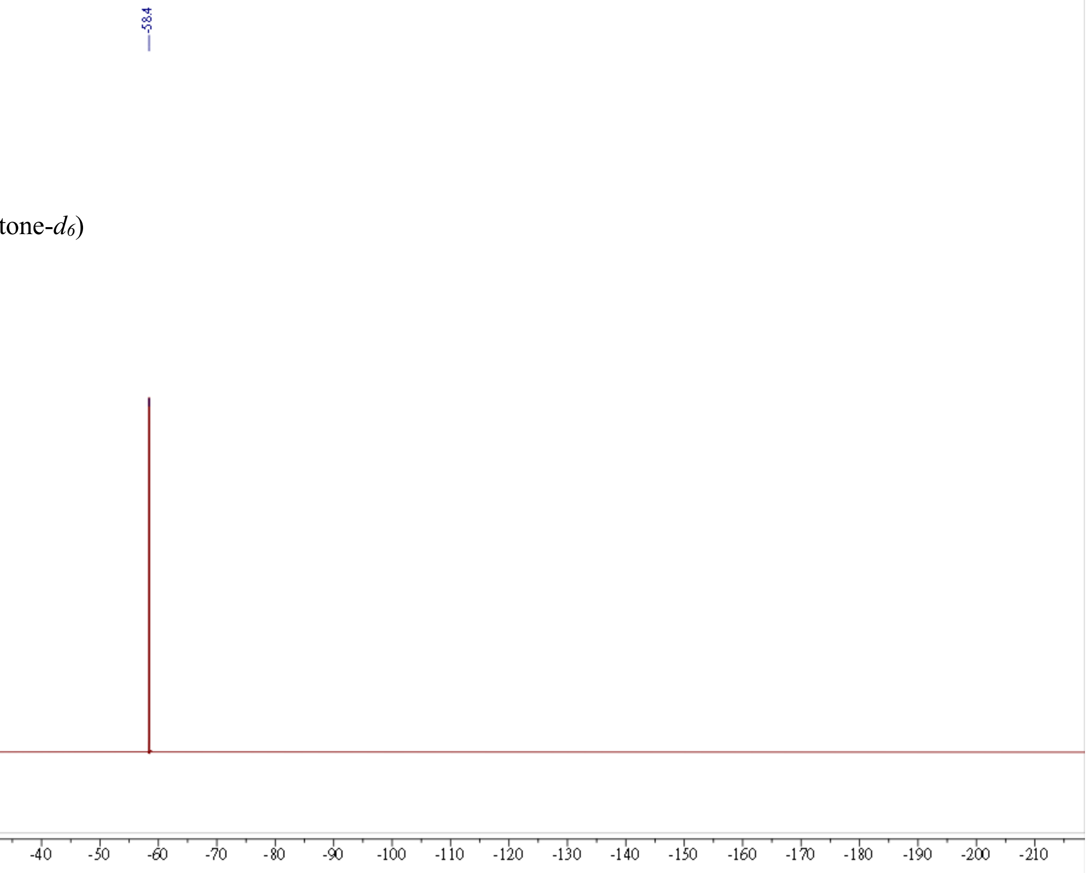

4-fluoro-*N*-tosylbenzamide (3f)

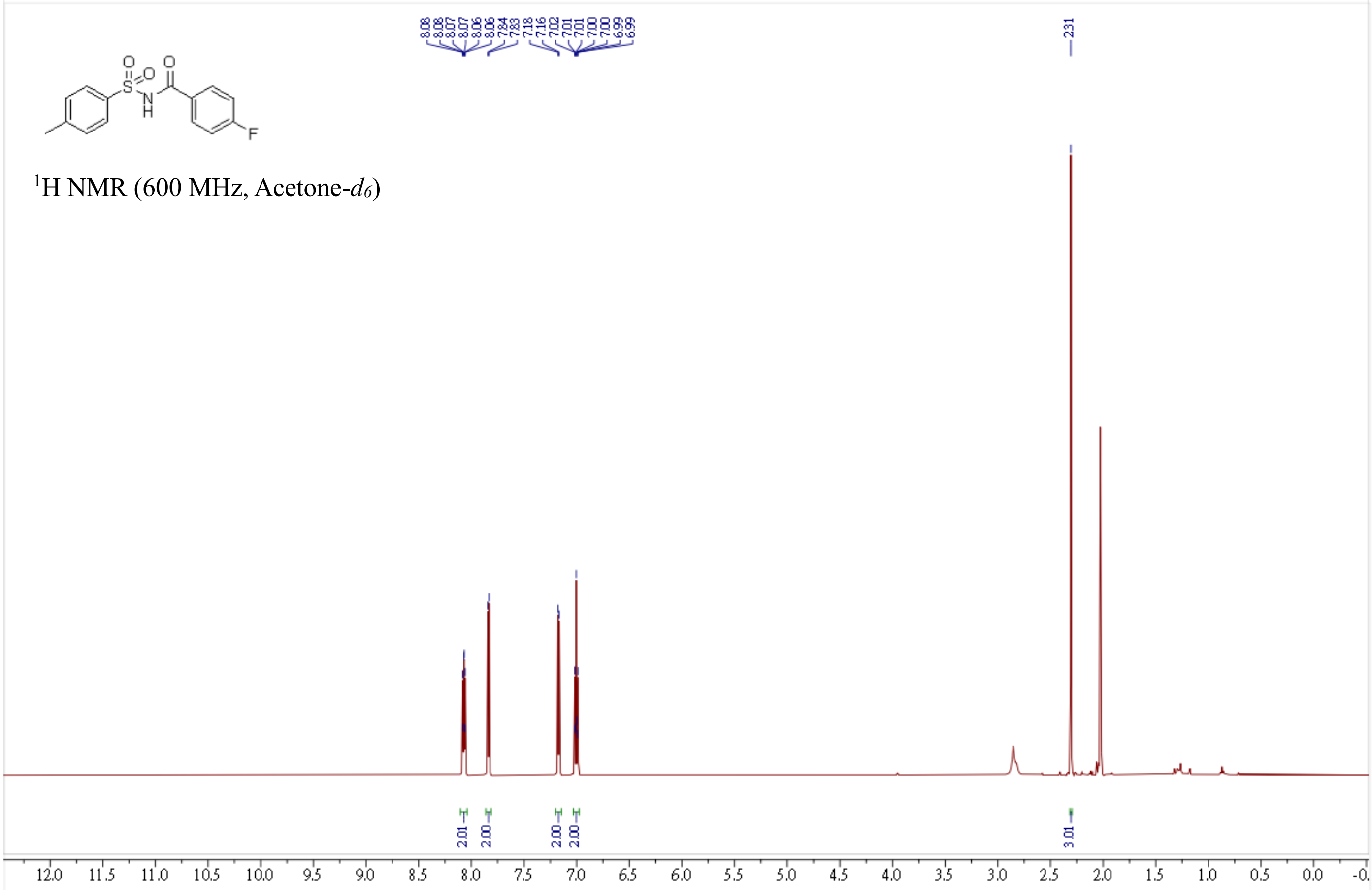

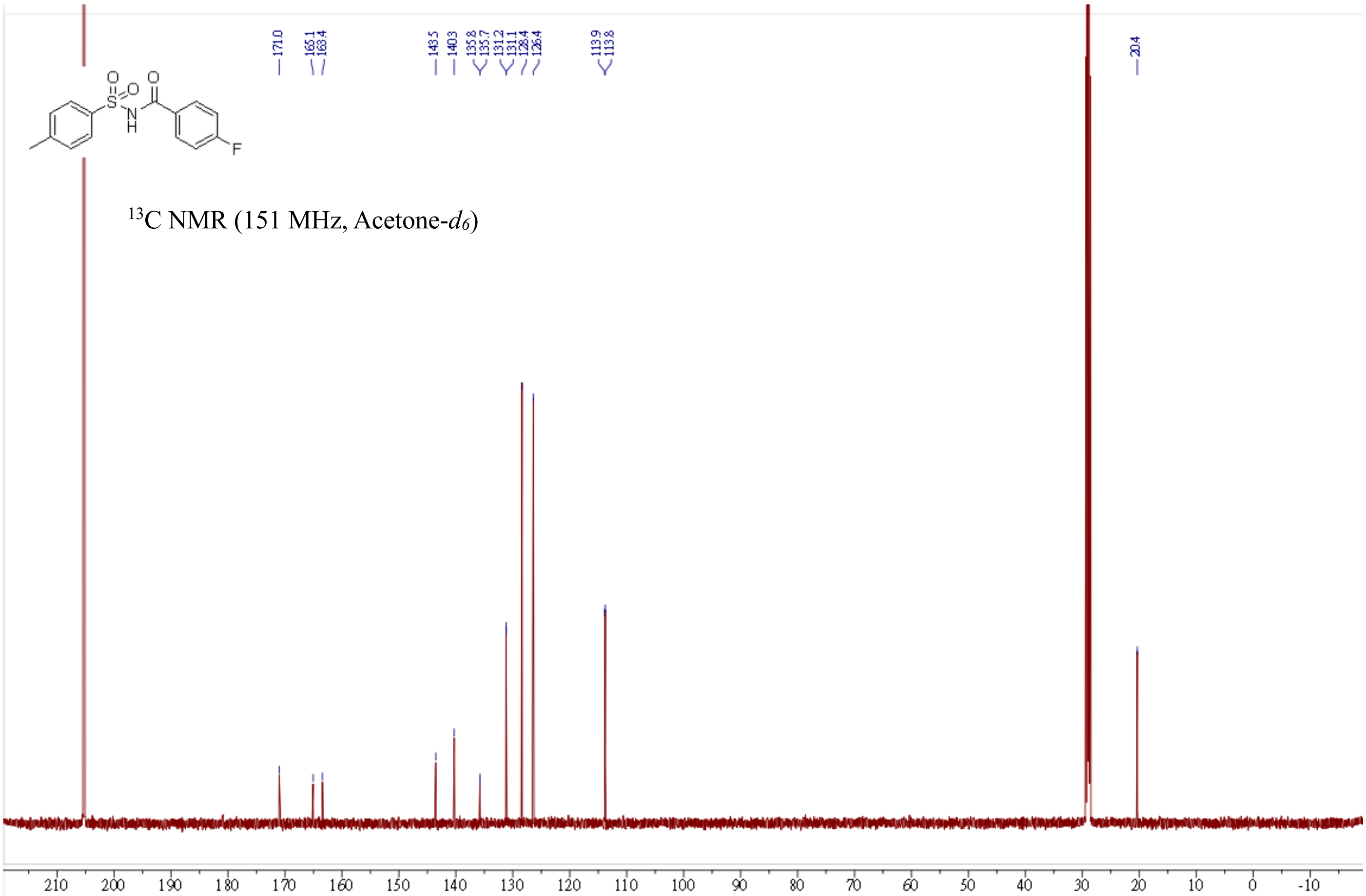

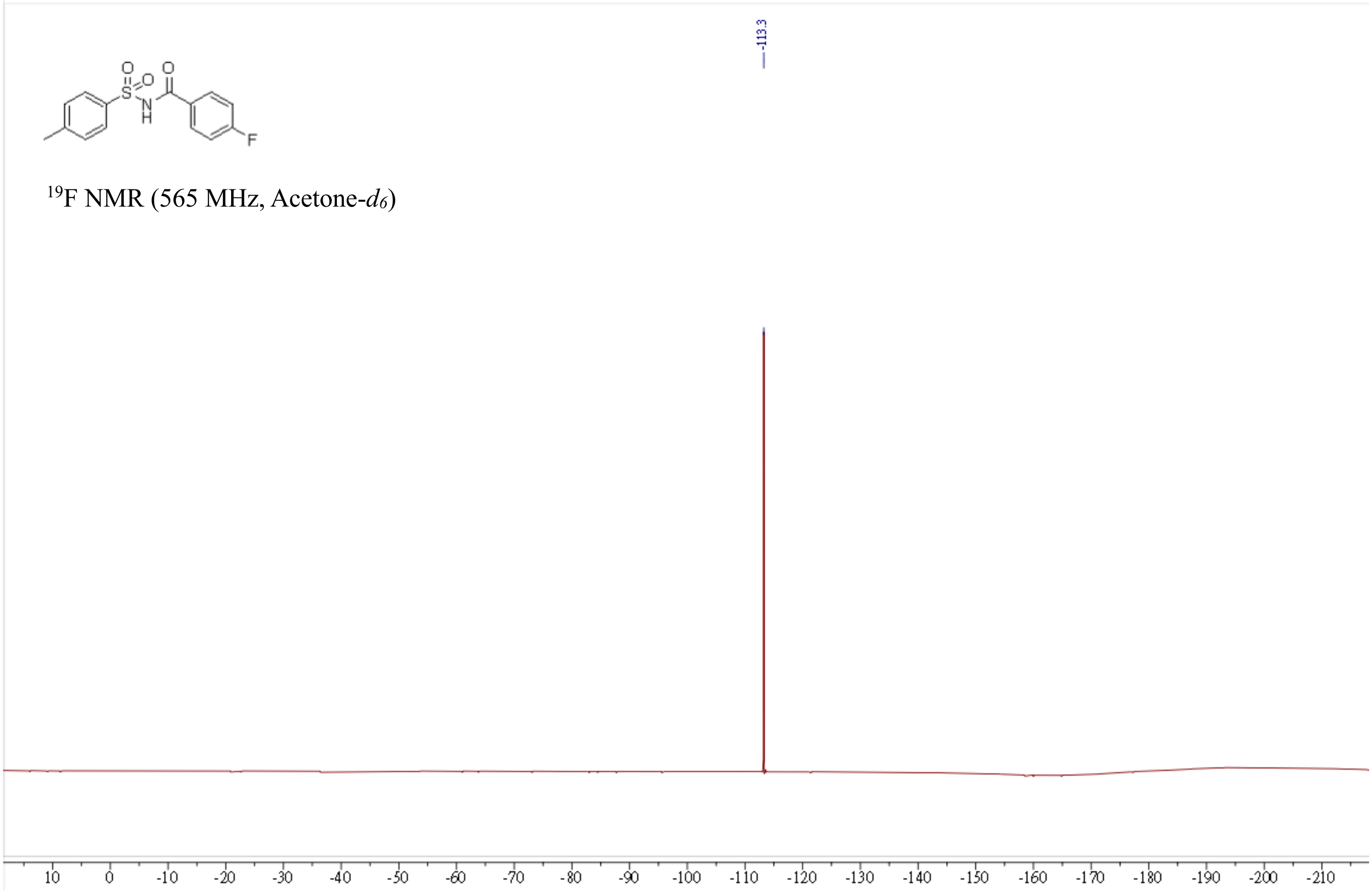
-113.3
19F NMR (565 MHz, Acetone-d6)
10 0 -10 -20 -30 -40 -50 -60 -70 -80 -90 -100 -110 -120 -130 -140 -150 -160 -170 -180 -190 -200 -210

4-ethynyl-*N*-tosylbenzamide (3g)

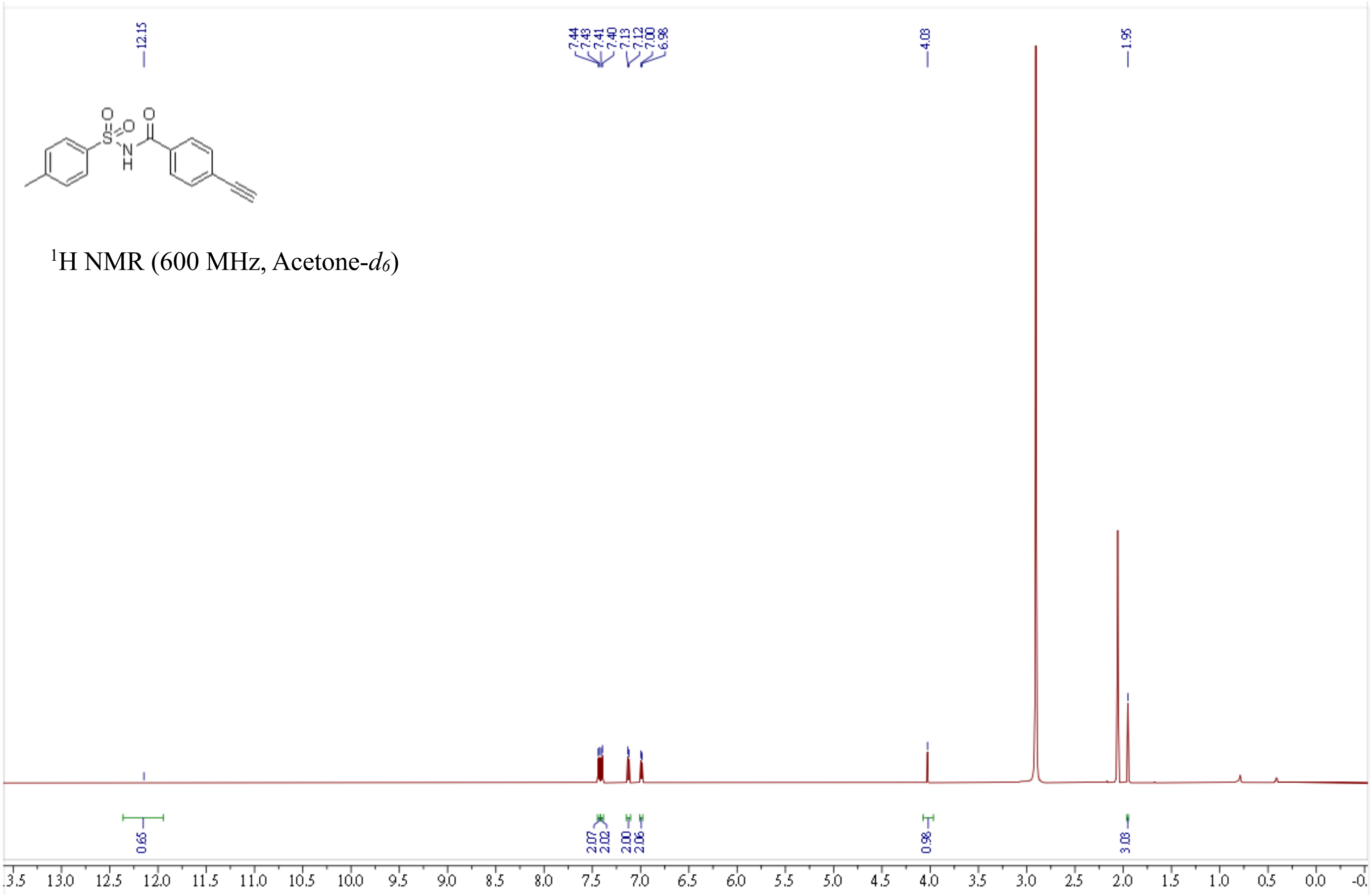

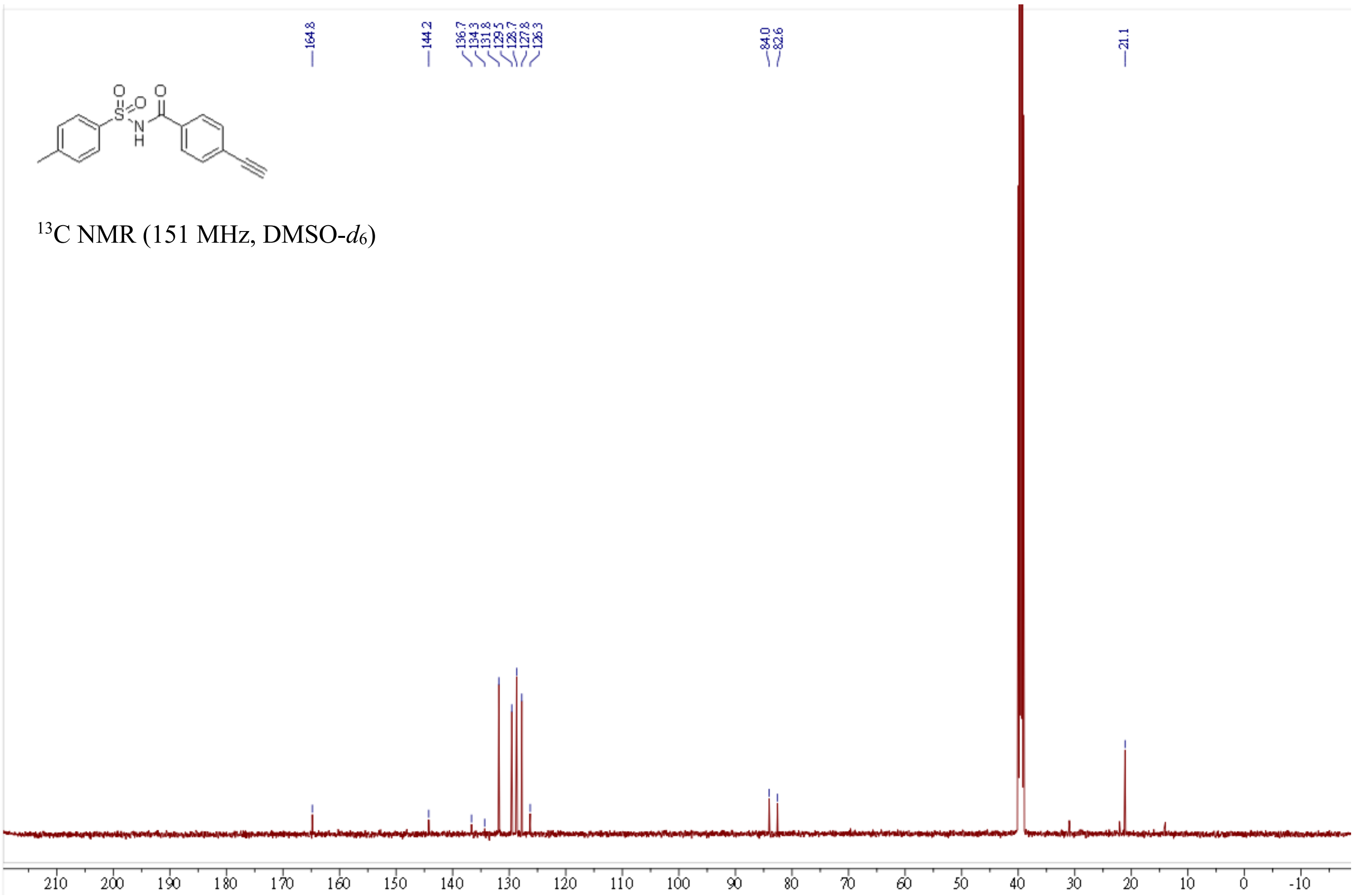

4-hydroxy-*N*-tosylbenzamide (3h)

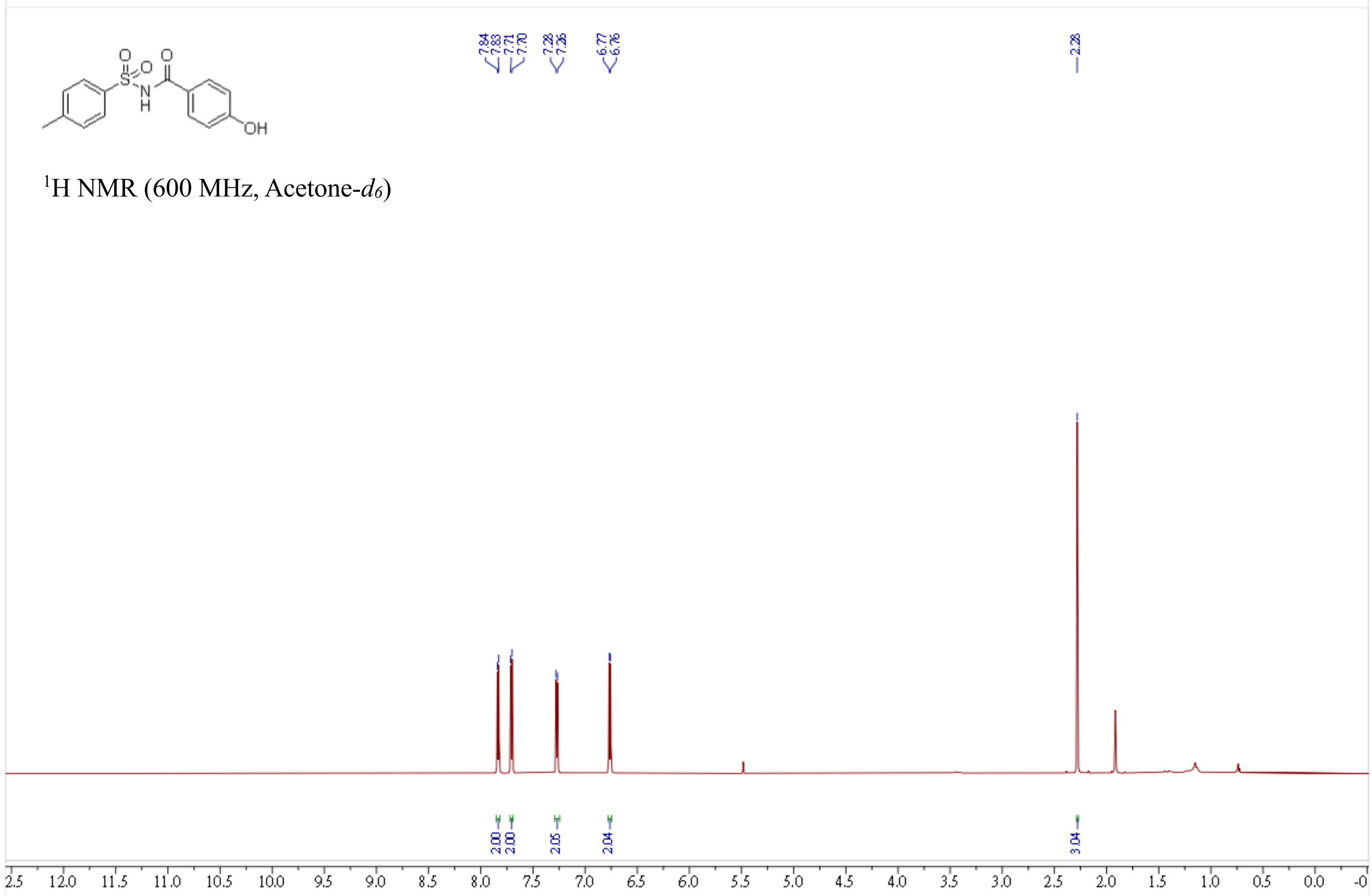

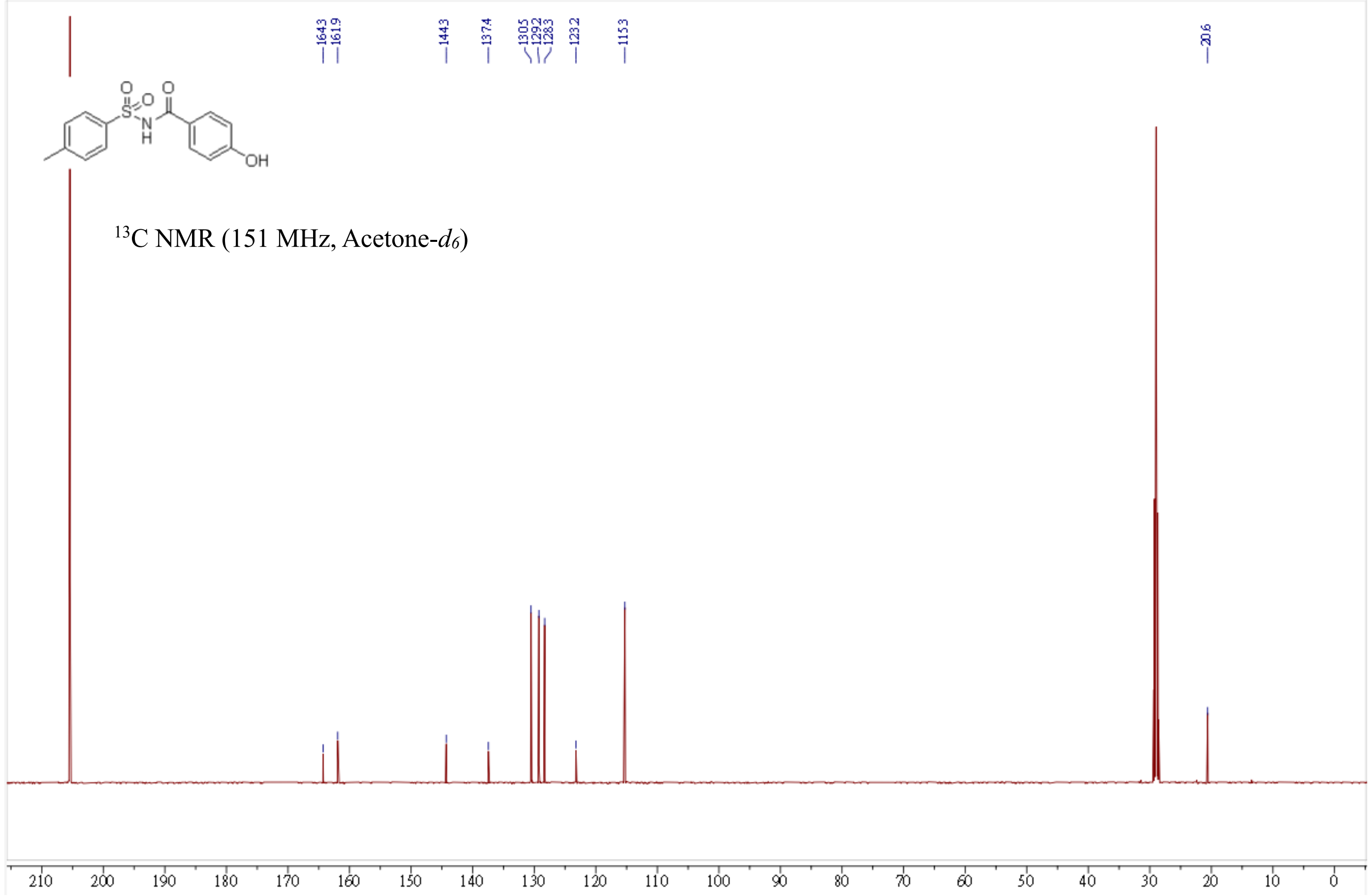

2-hydroxy-*N*-tosylbenzamide (3i)

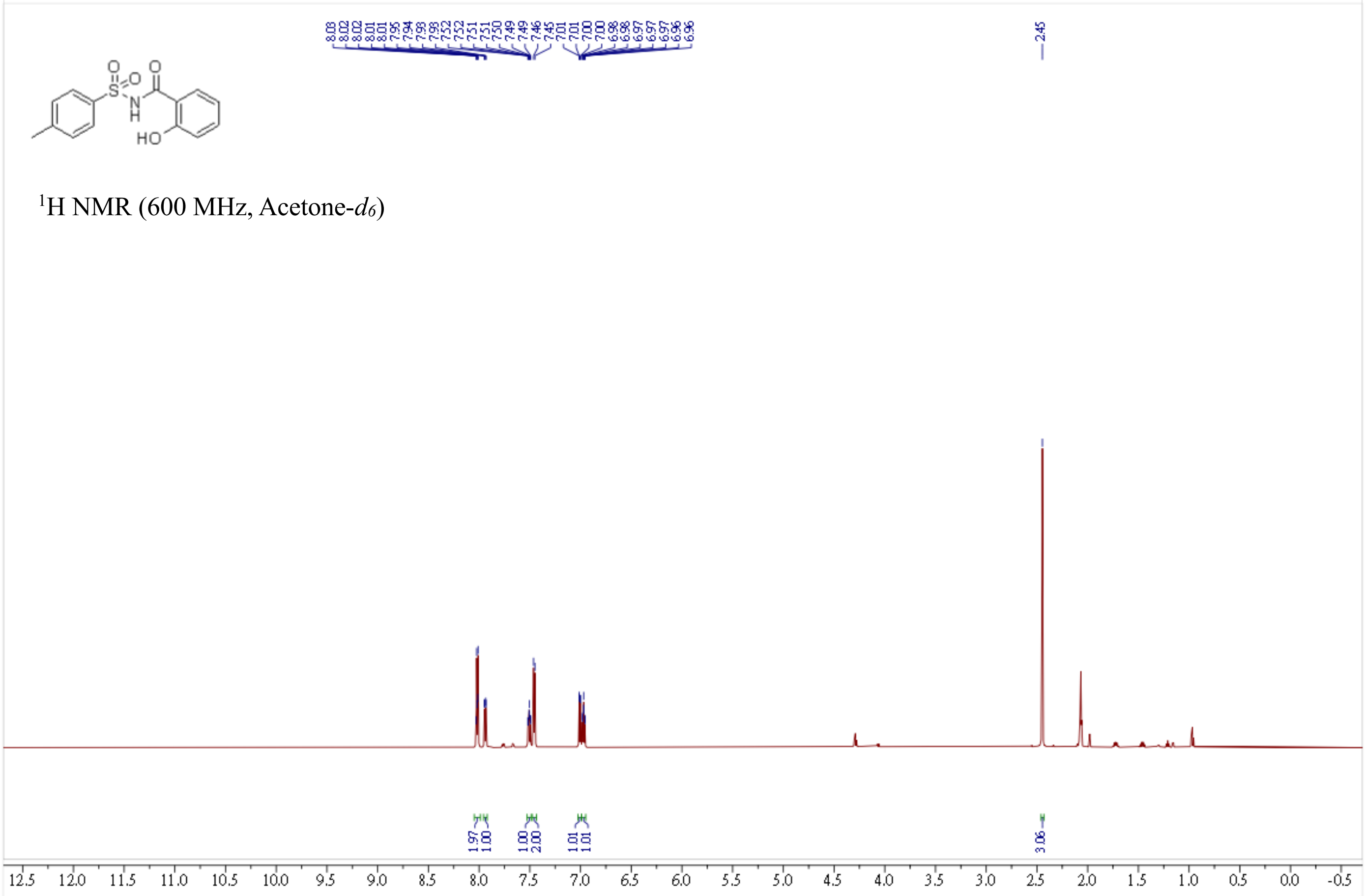

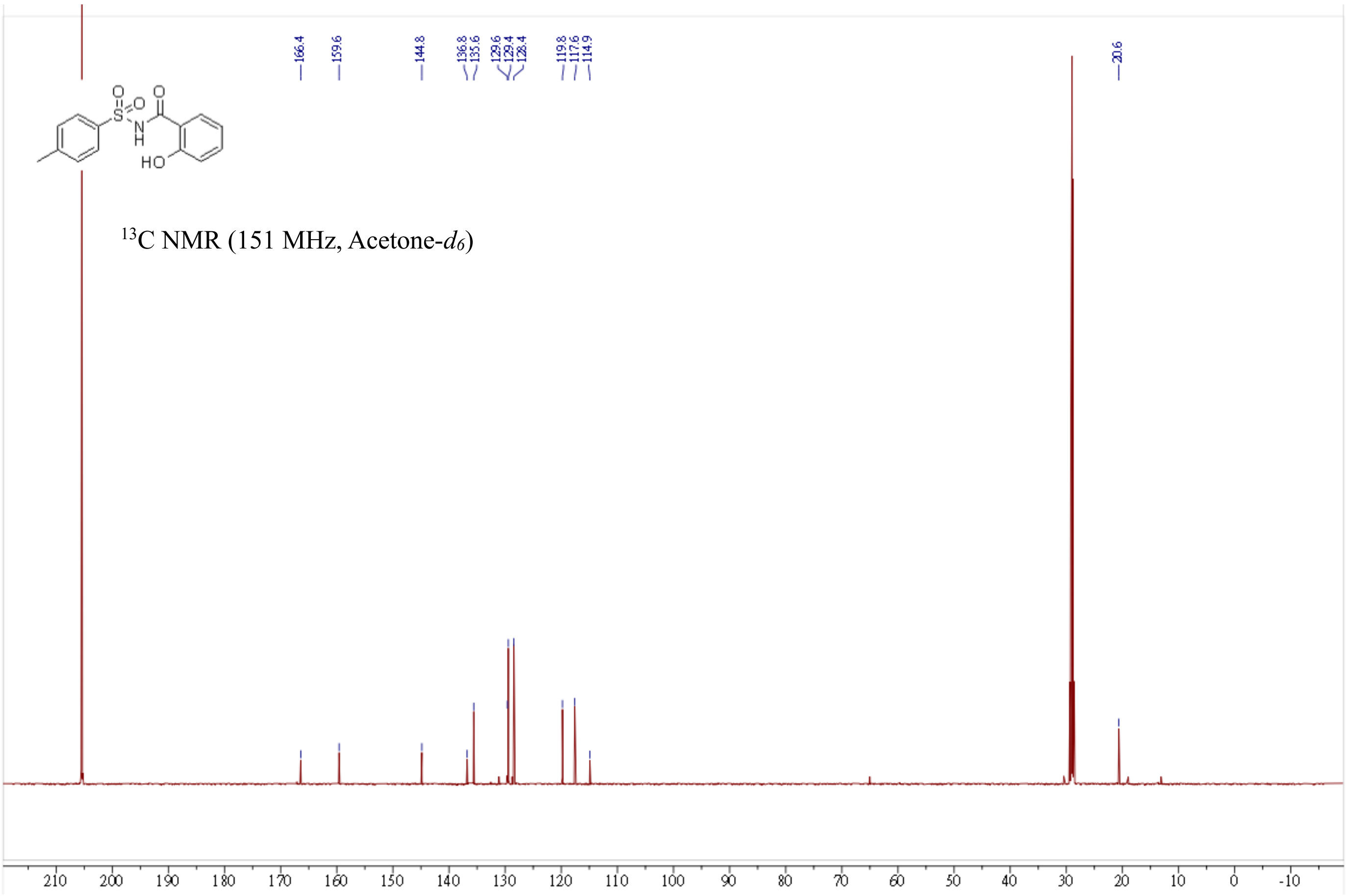

3,5-dichloro-*N*-tosylbenzamide (3j)

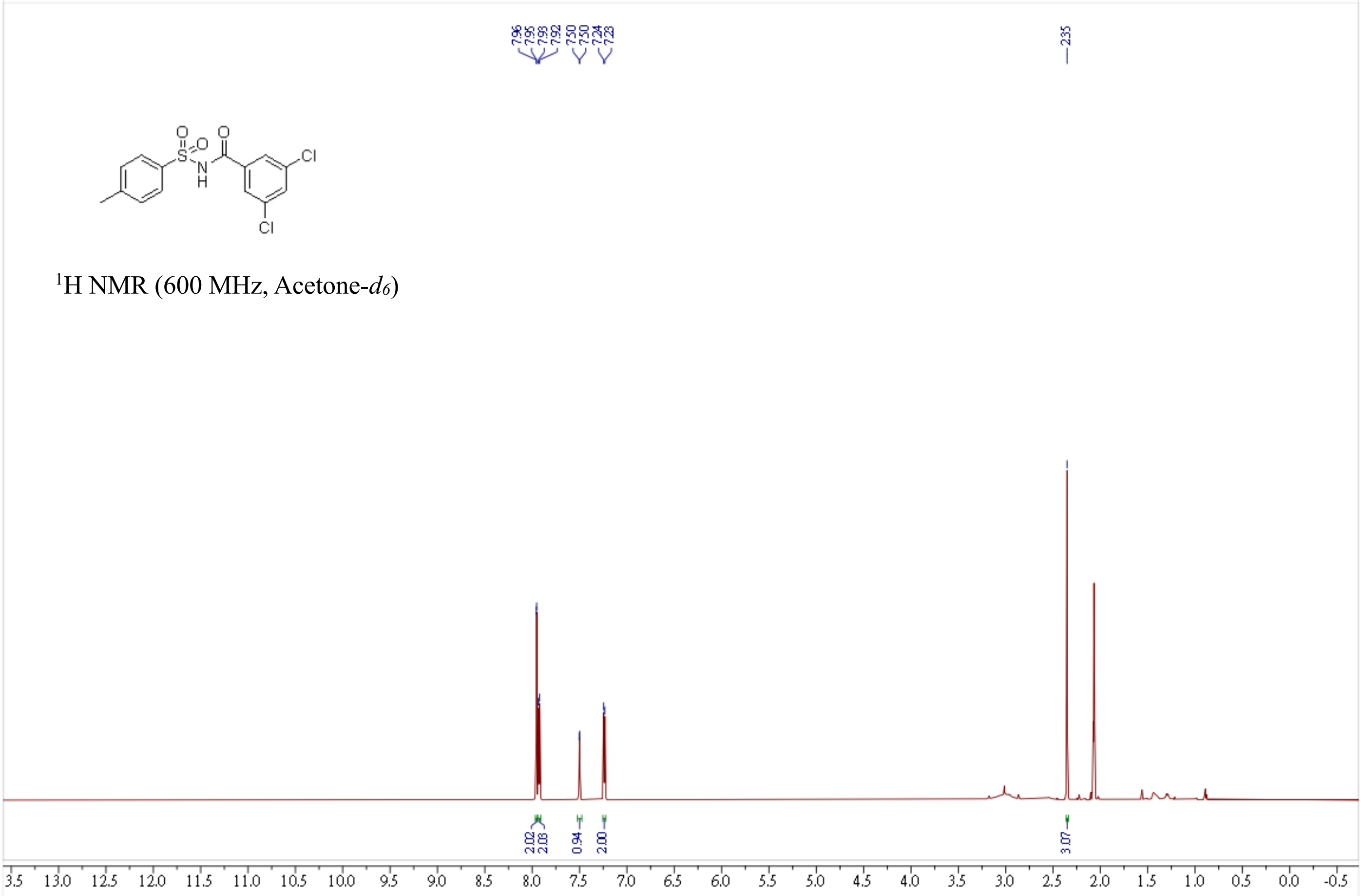

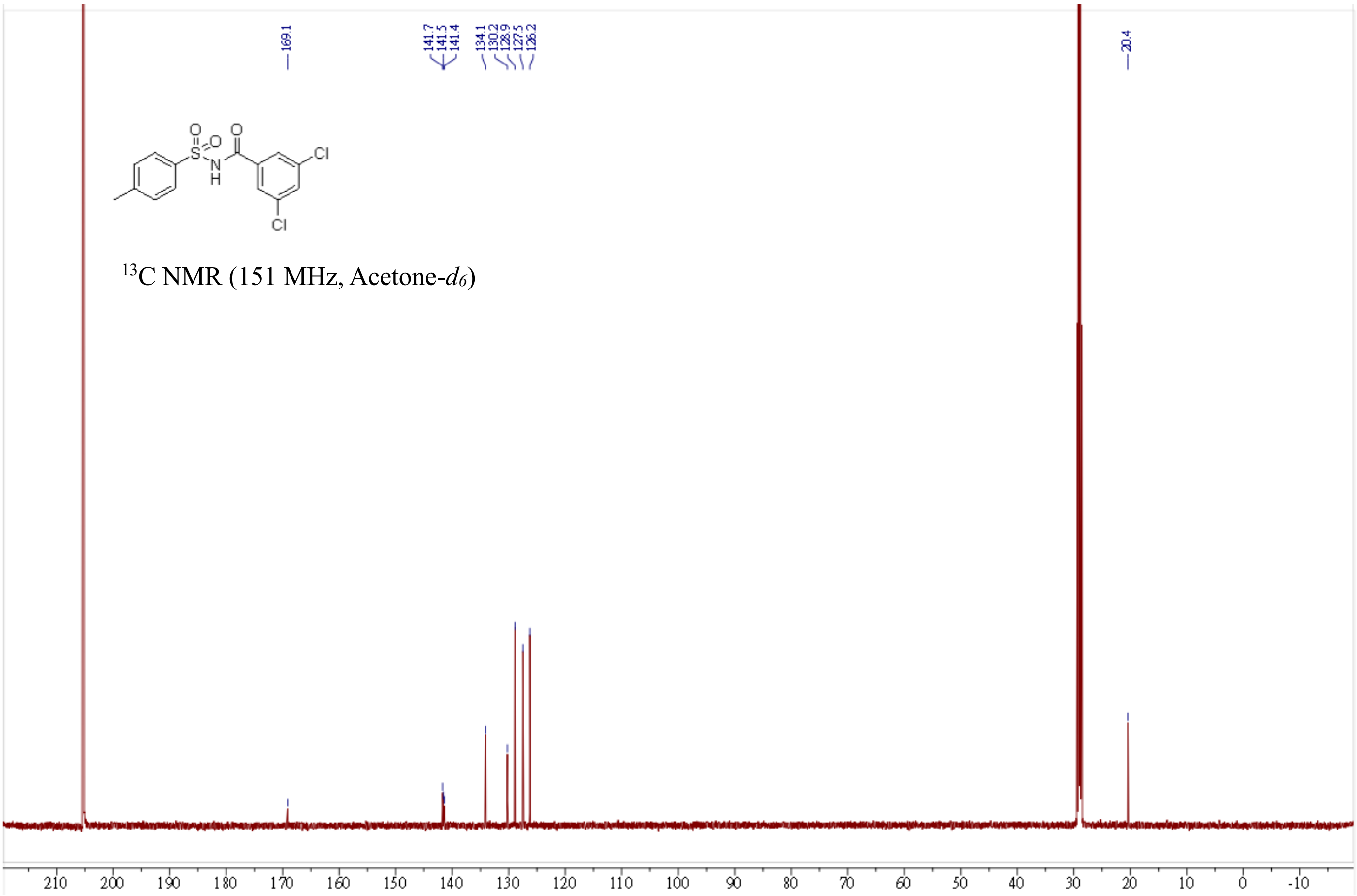

N-tosyl-2-naphthamide (3k)

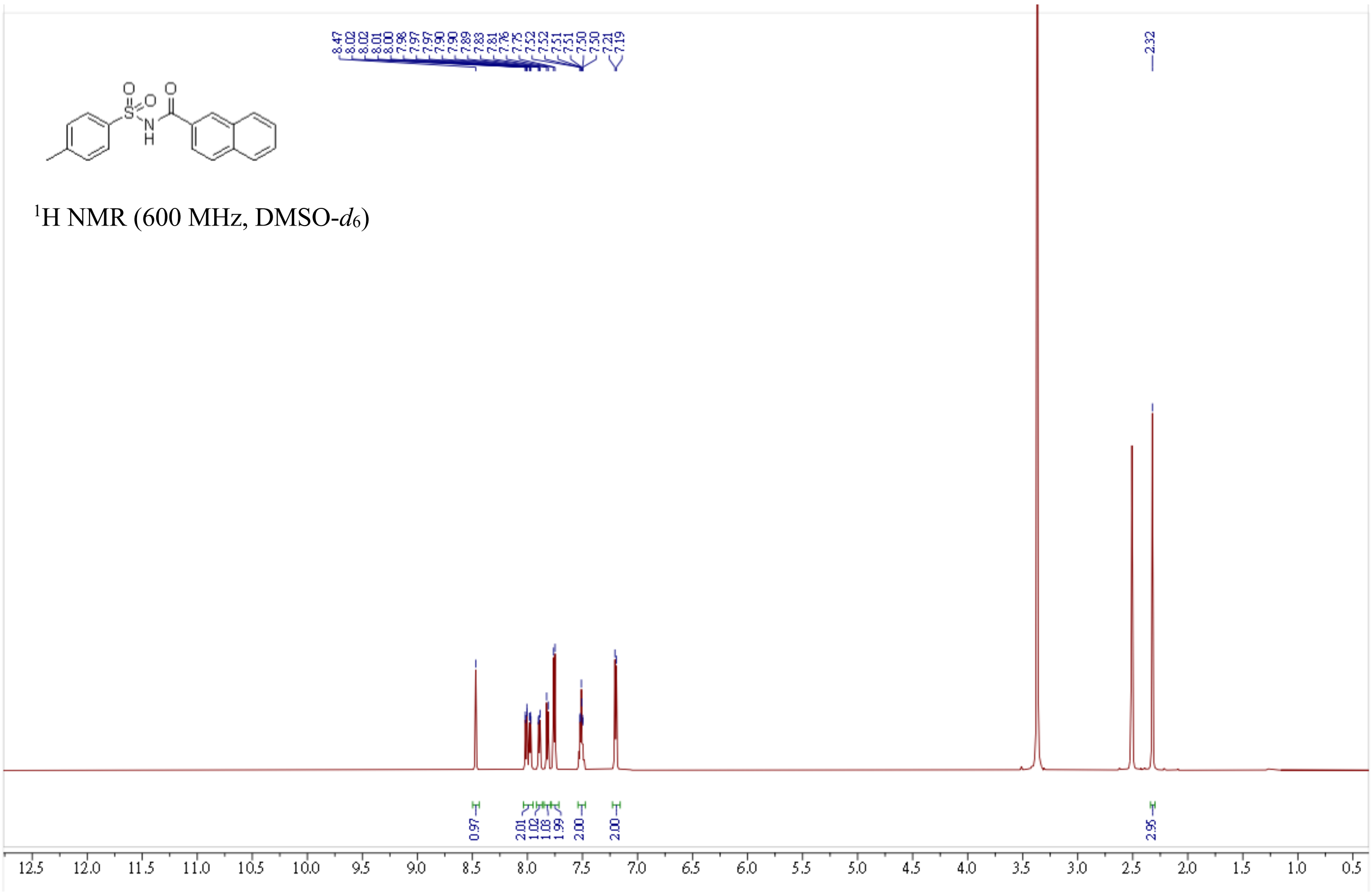

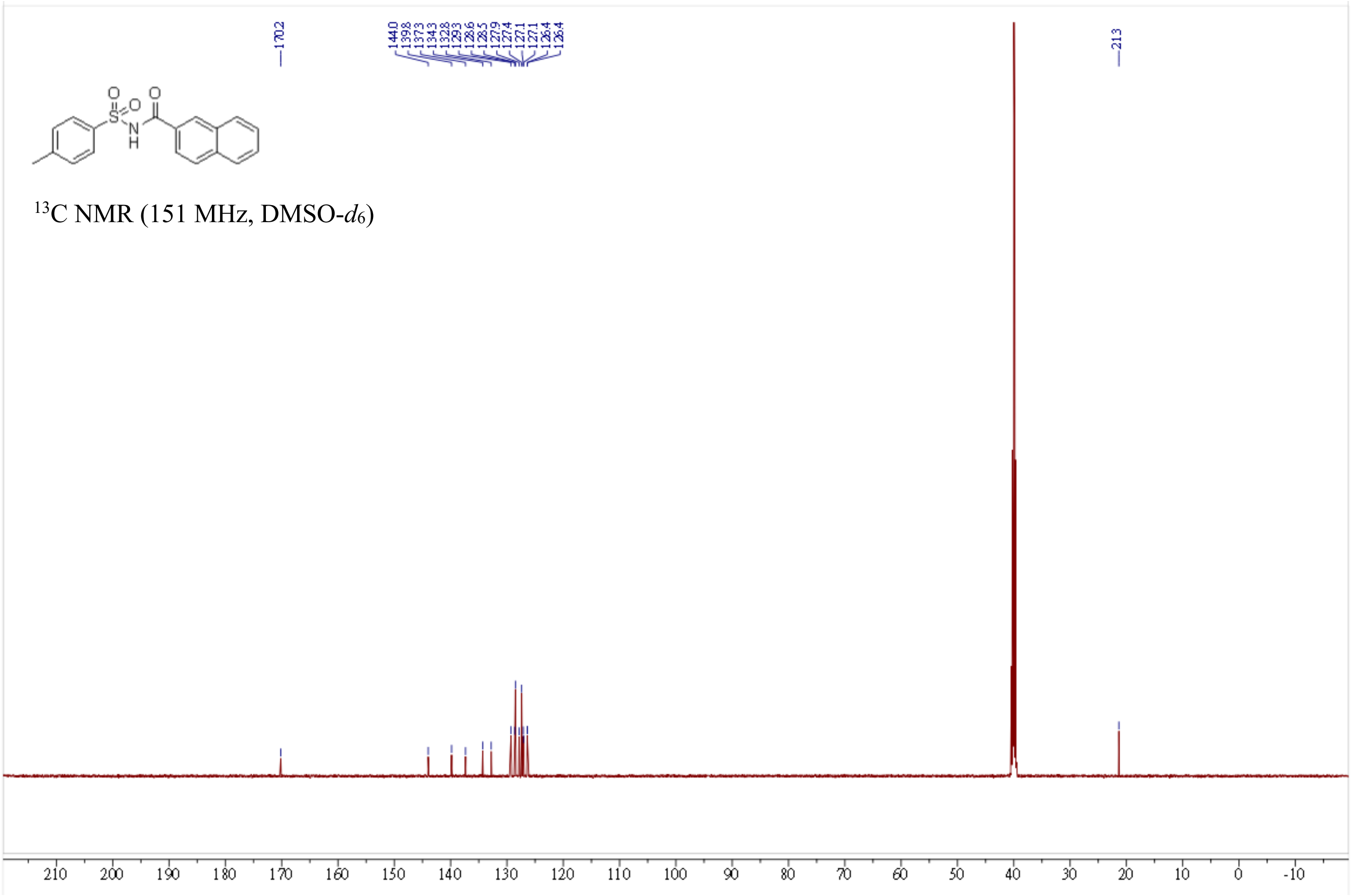

N-tosylbenzofuran-5-carboxamide (3l)

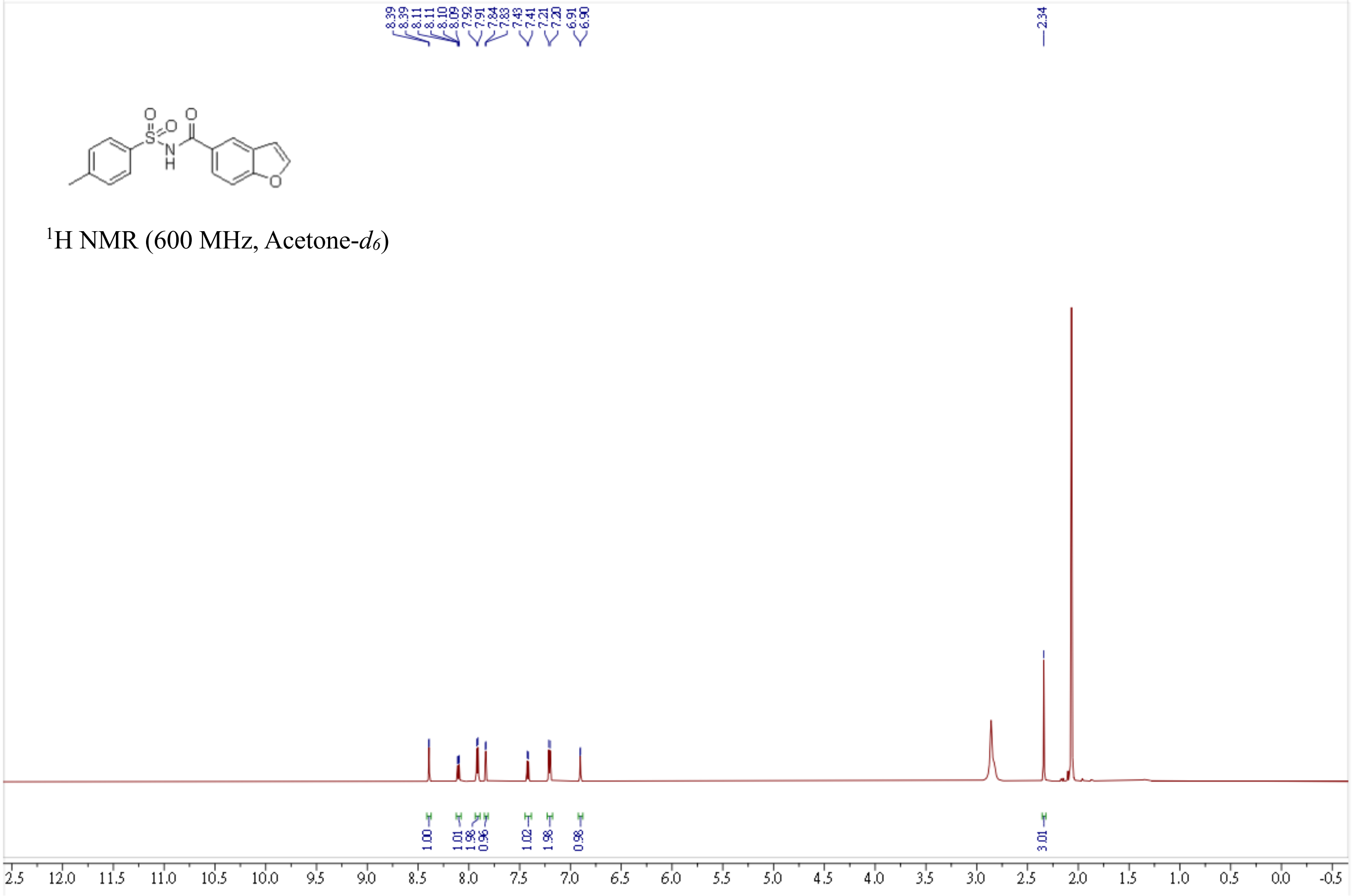

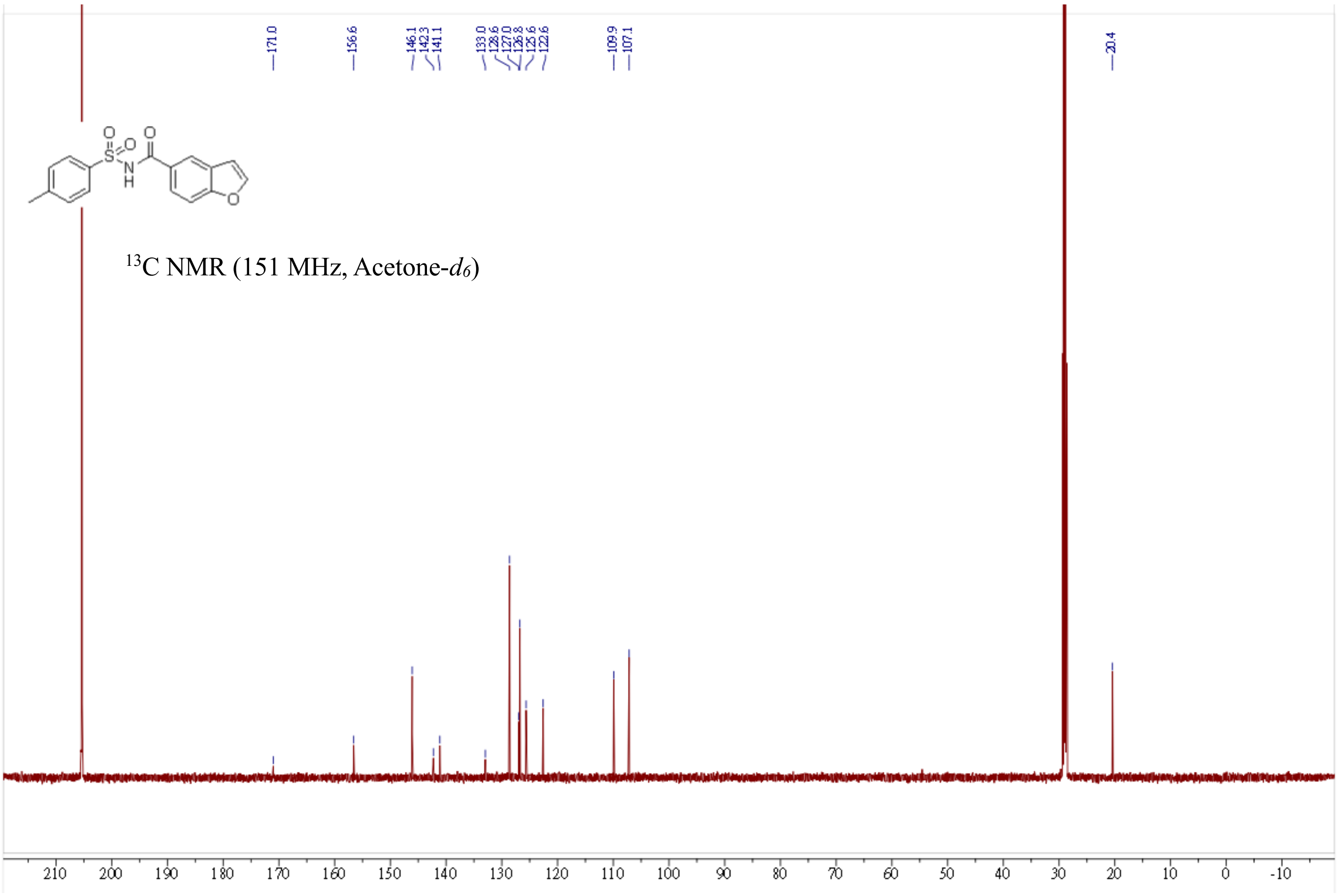

N-tosylthiophene-2-carboxamide (3m)

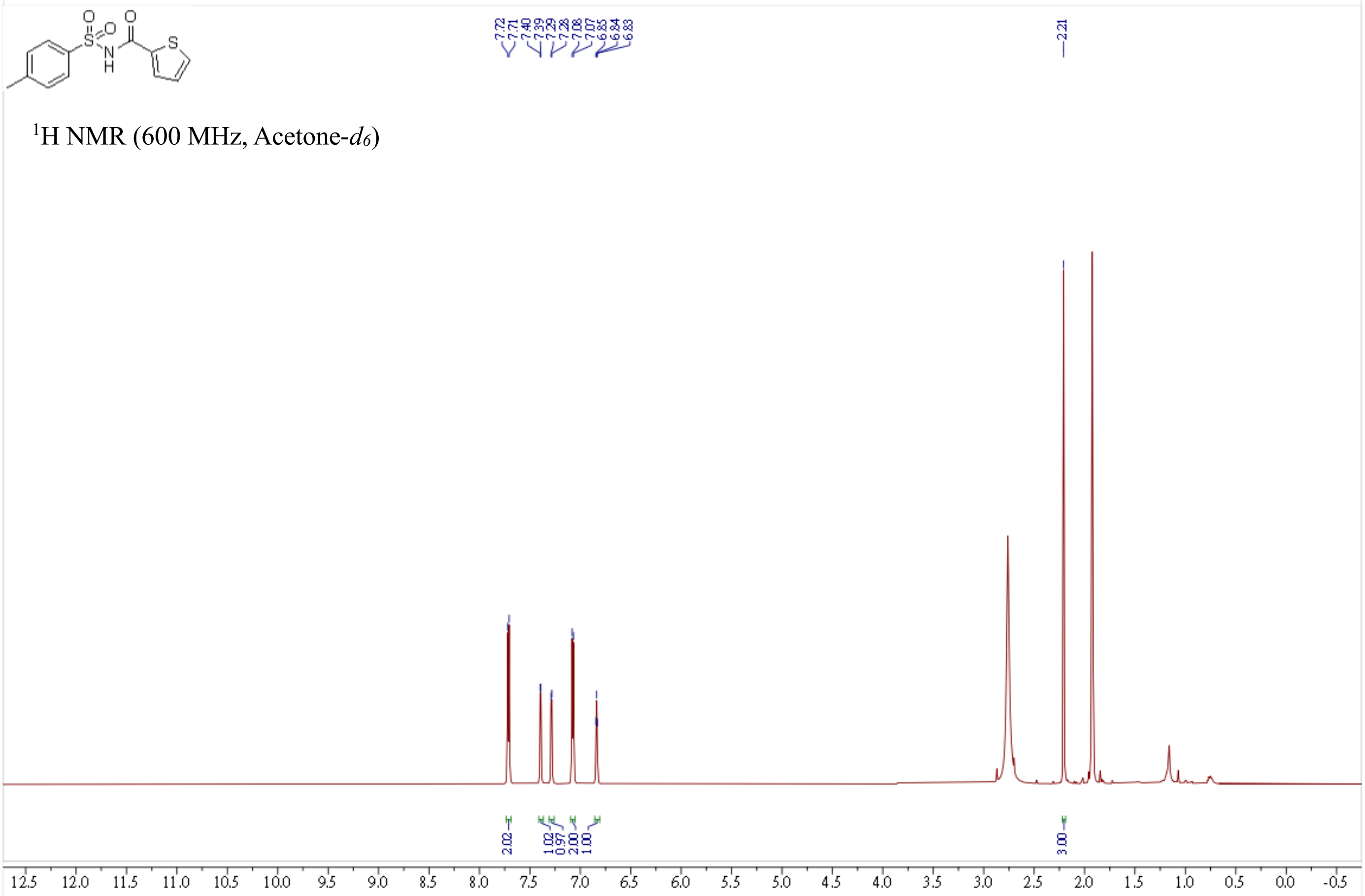

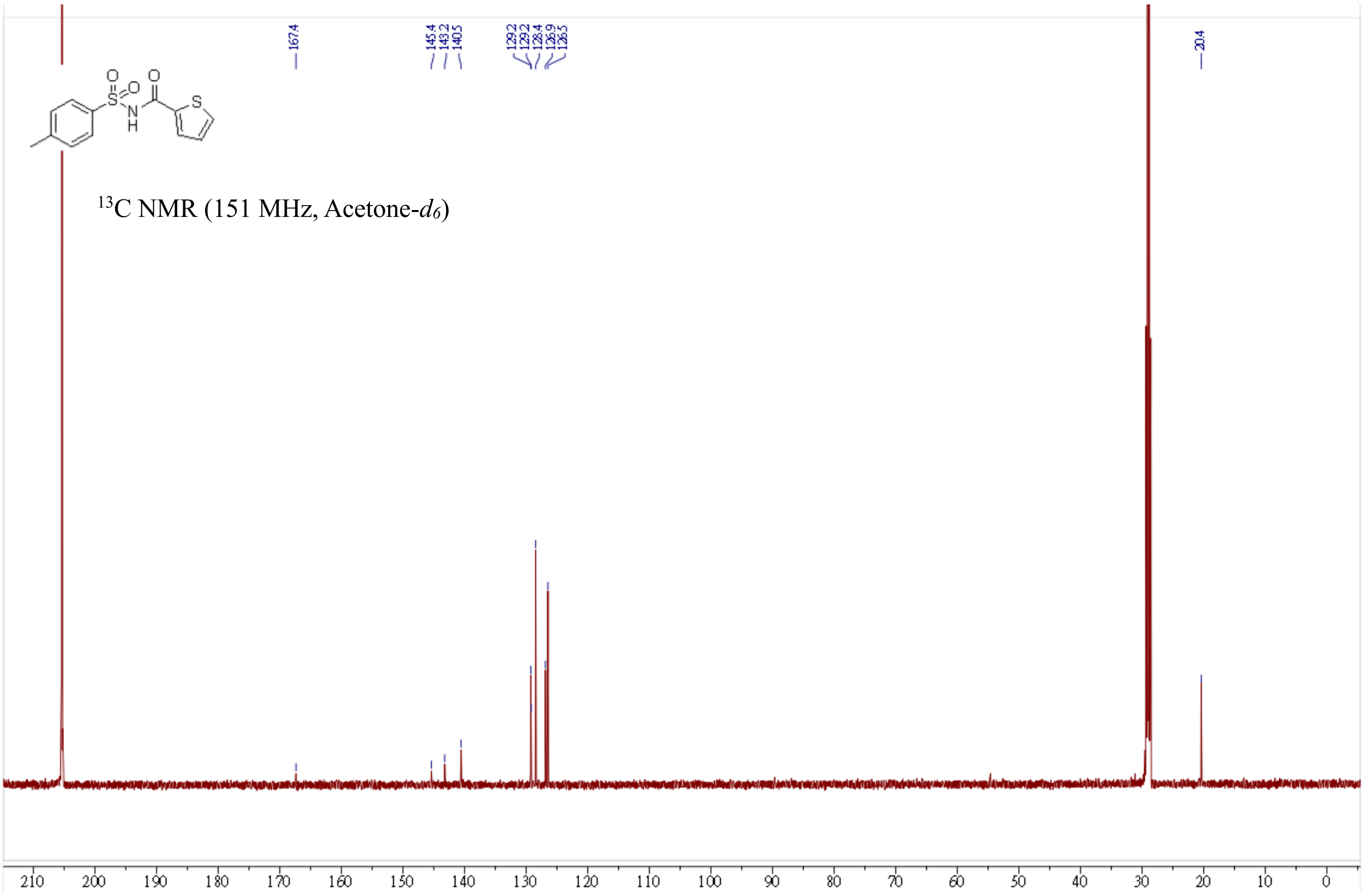

5-methyl-*N*-tosylisoxazole-3-carboxamide (3n)

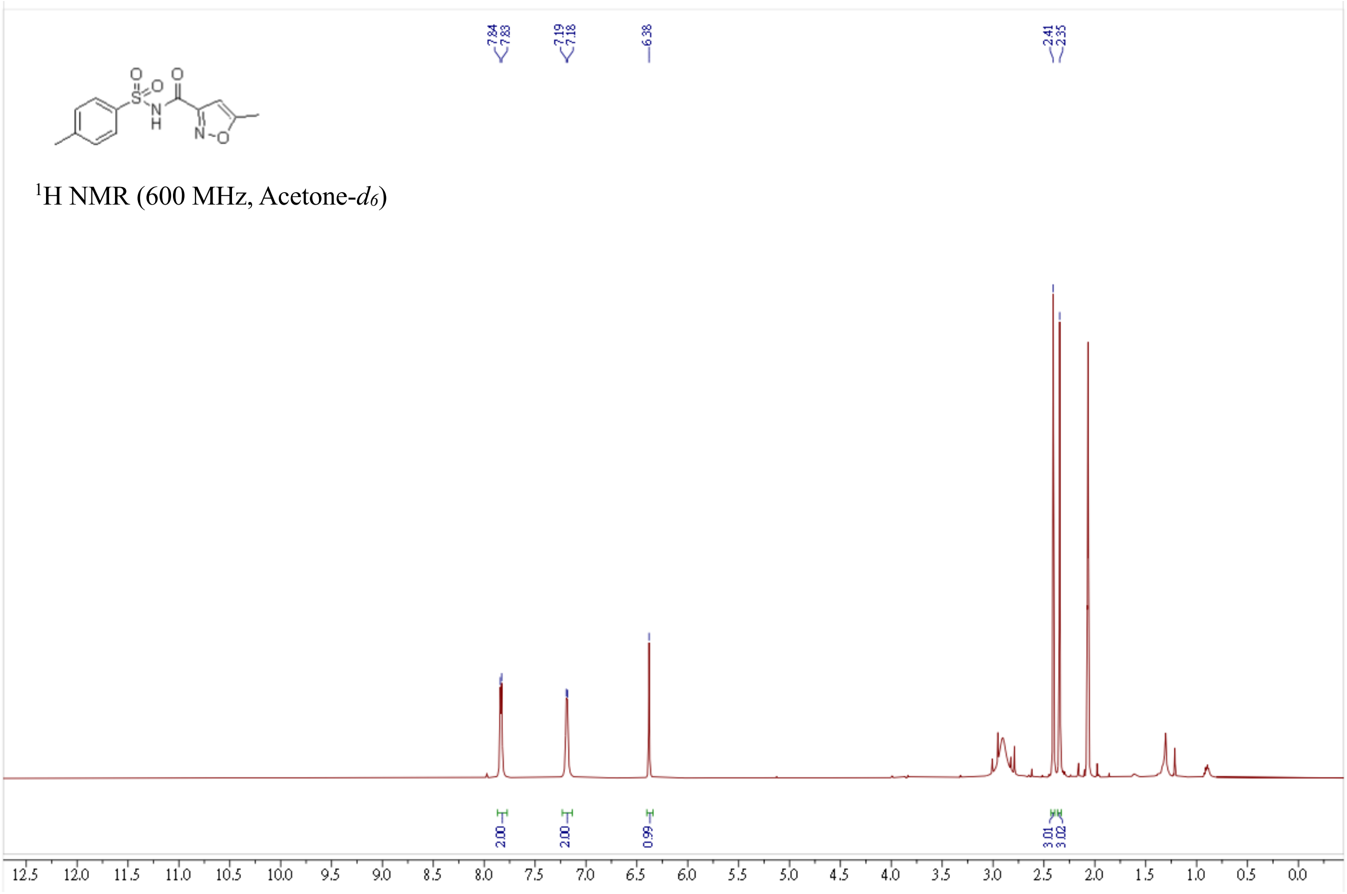

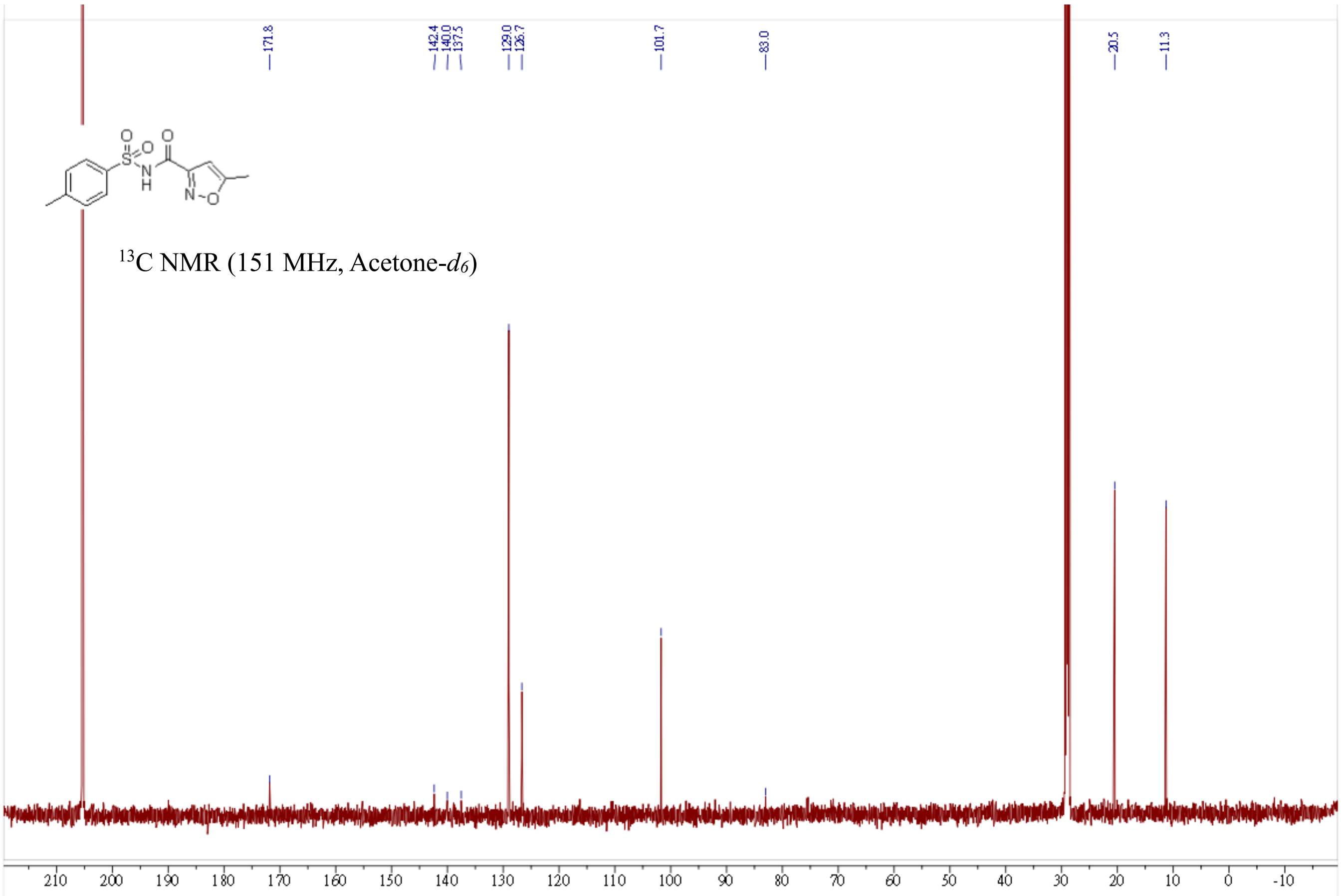

benzyl tosylcarbamate (3o)

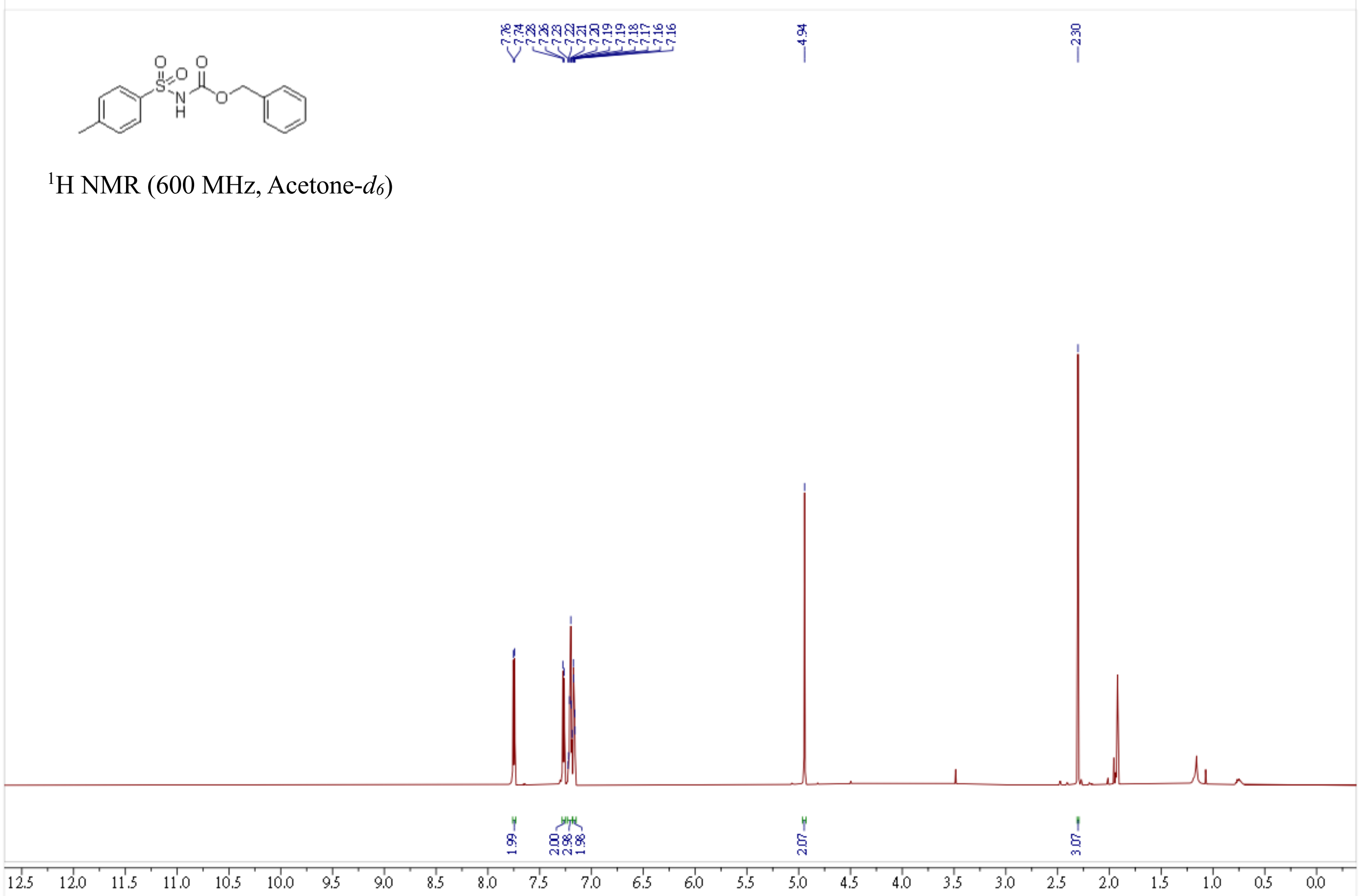

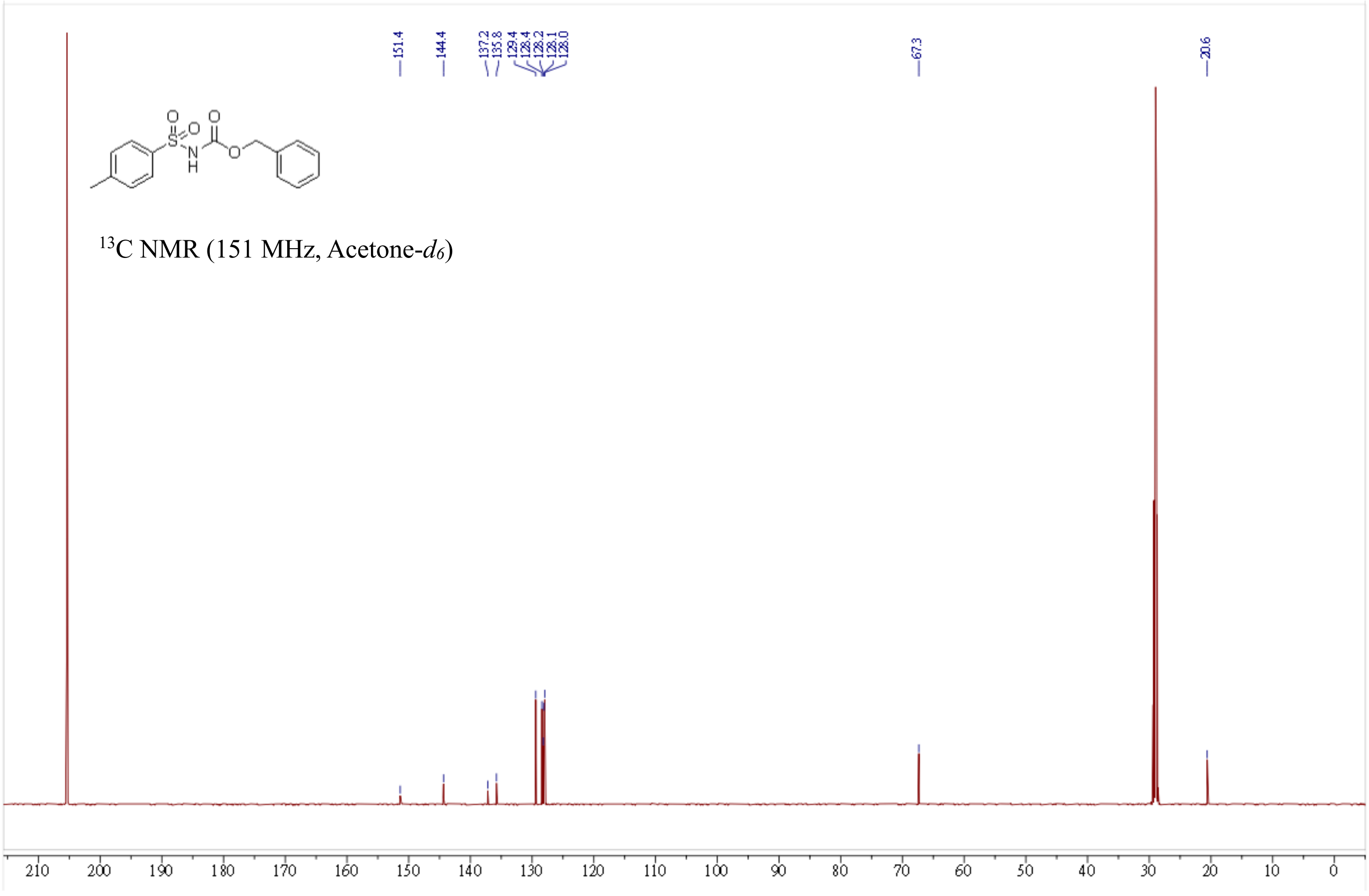

(9H-fluoren-9-yl)methyl tosylcarbamate (3p)

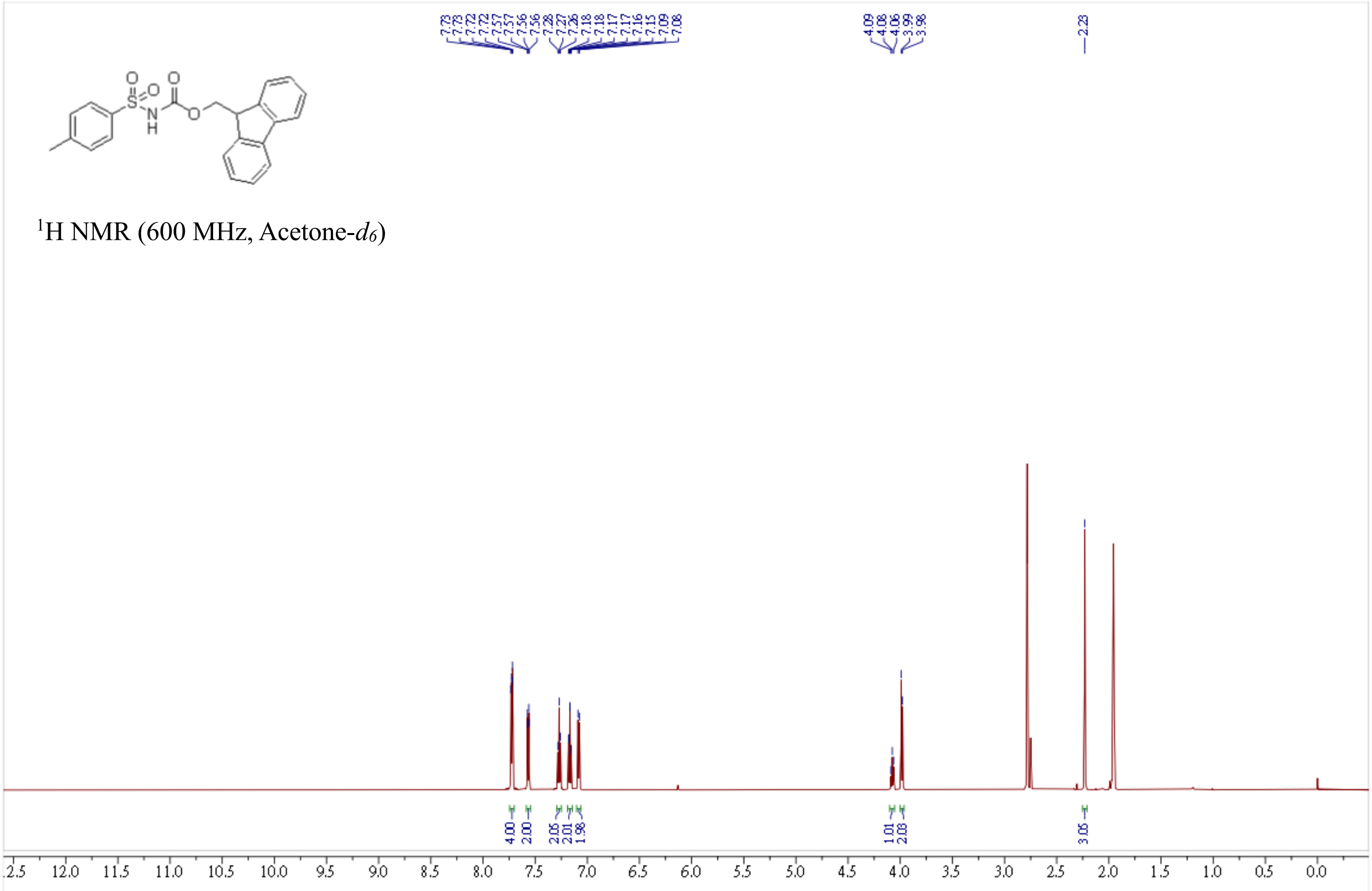

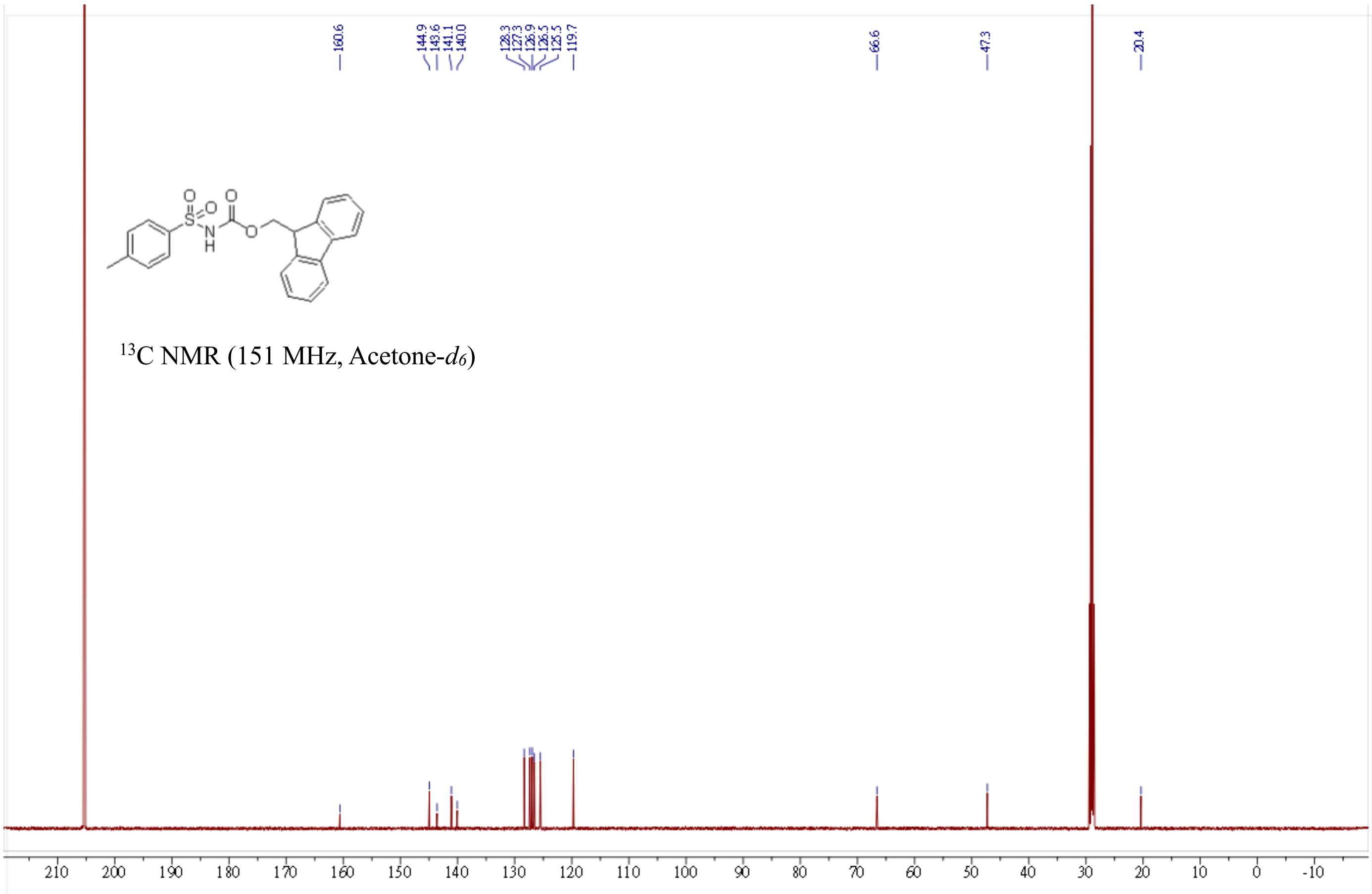

tert-butyl tosylcarbamate (3q)

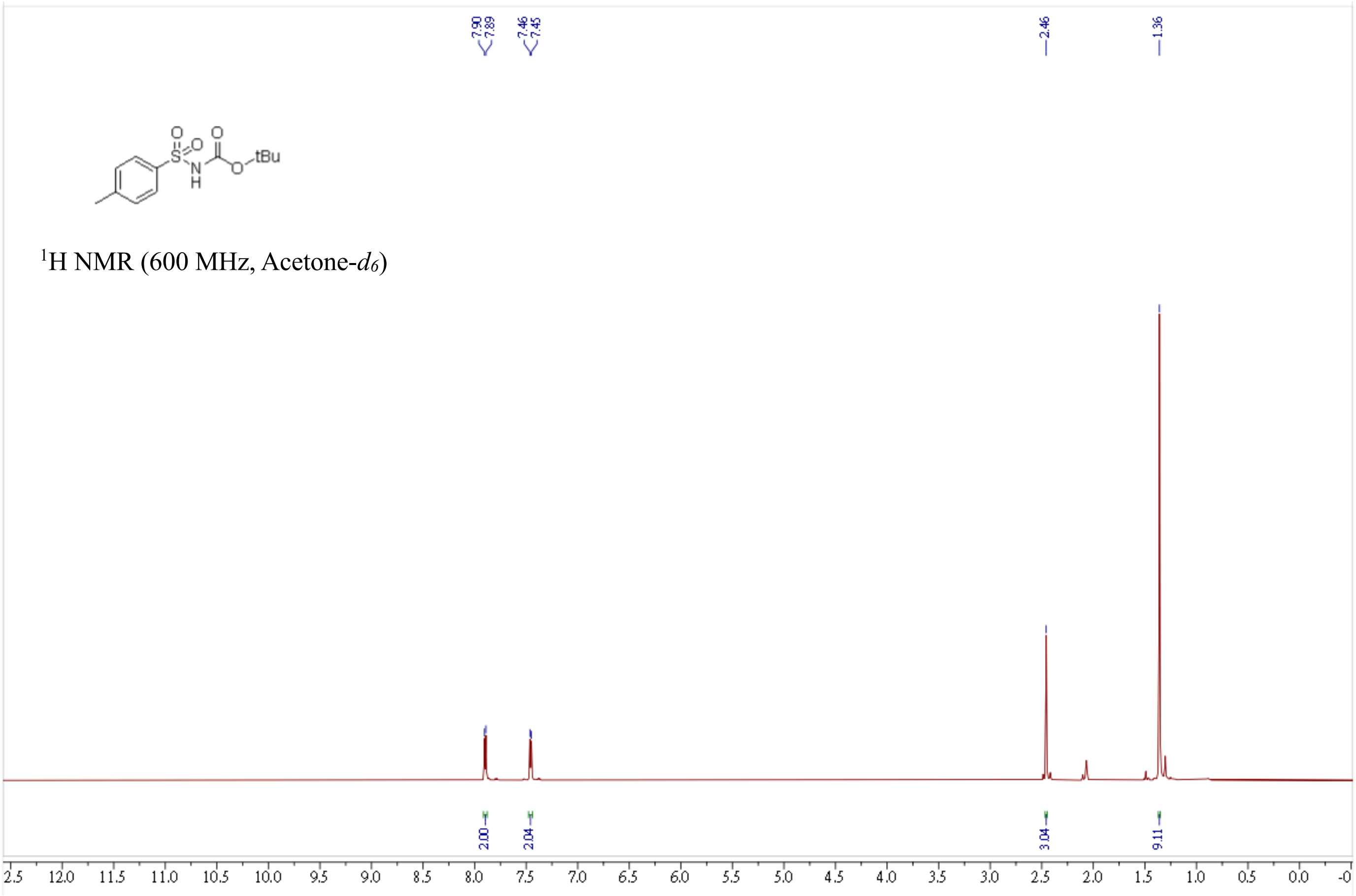

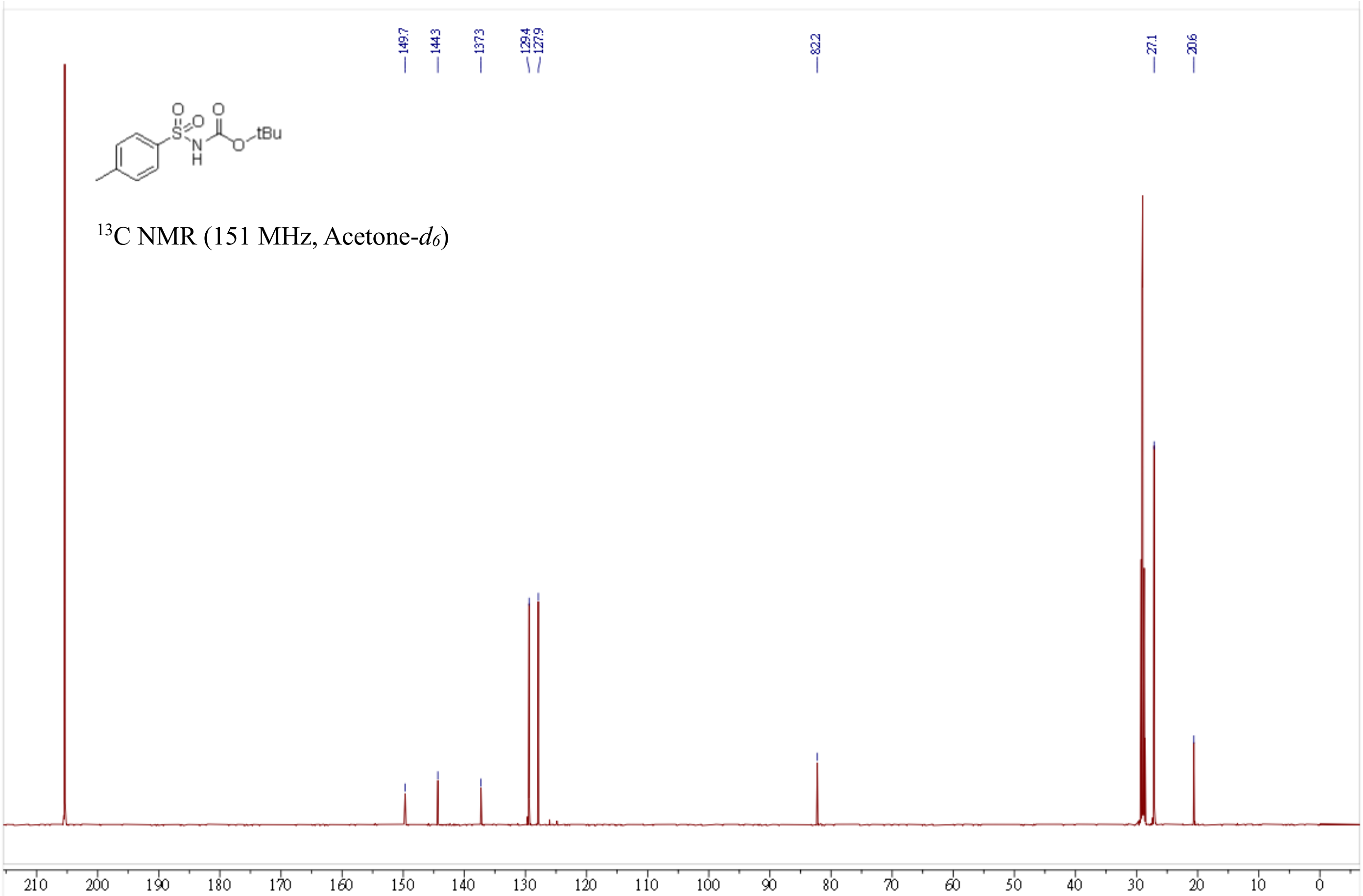

4-(tert-butyl)-*N*-((4-chlorophenyl)sulfonyl)benzamide (3r)

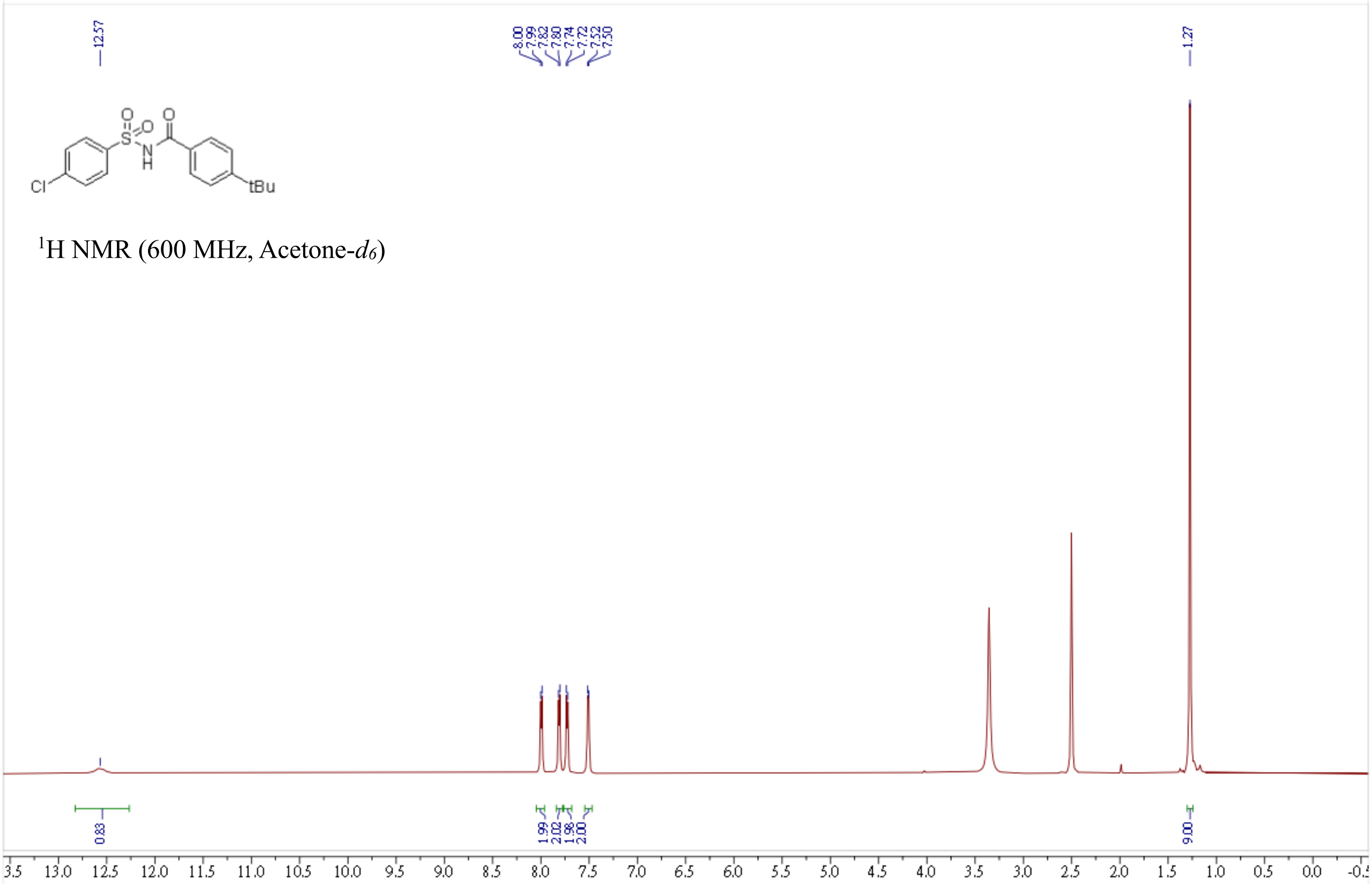

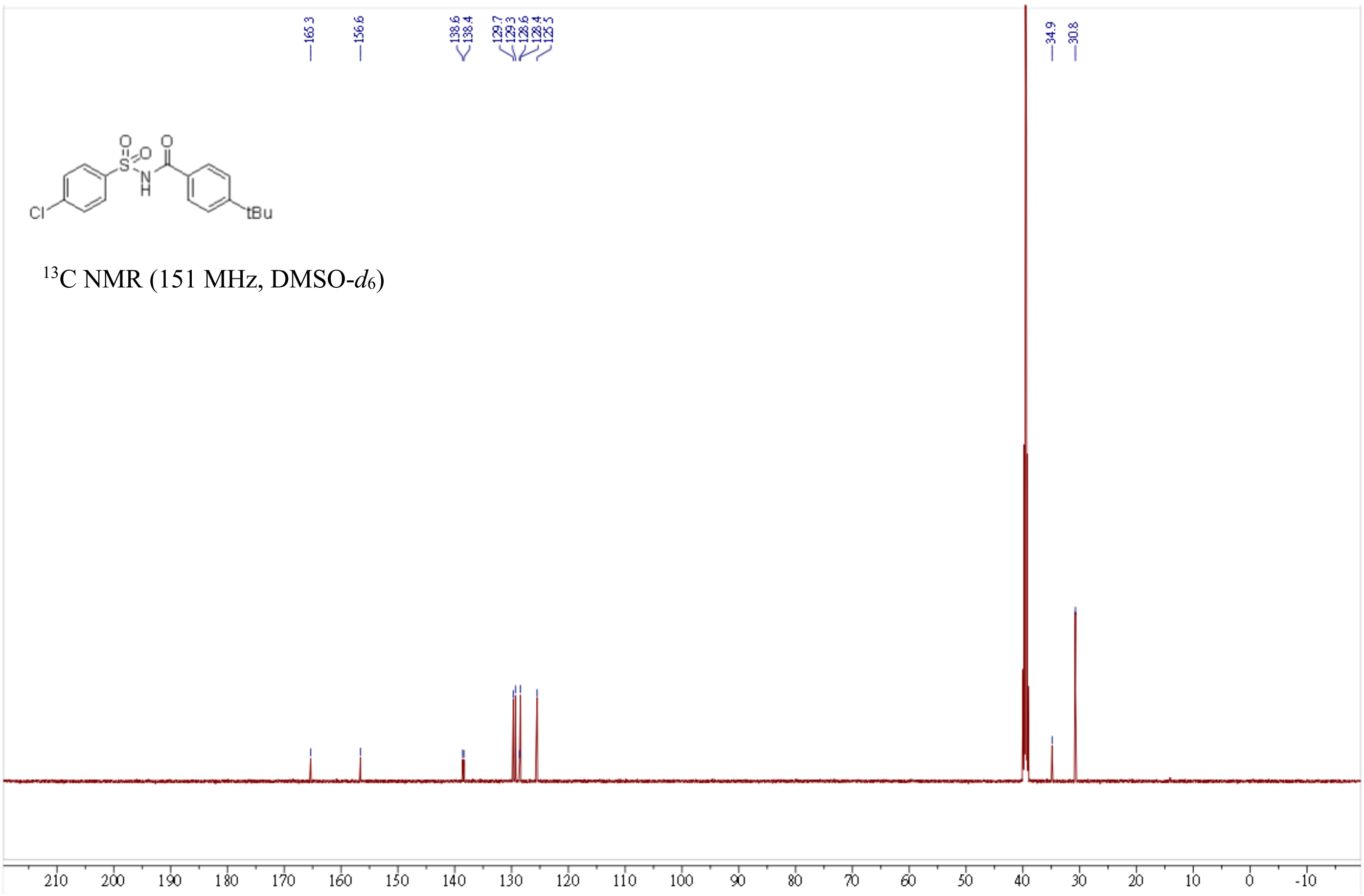

N-((4-bromophenyl)sulfonyl)-4-(tert-butyl)benzamide (3s)

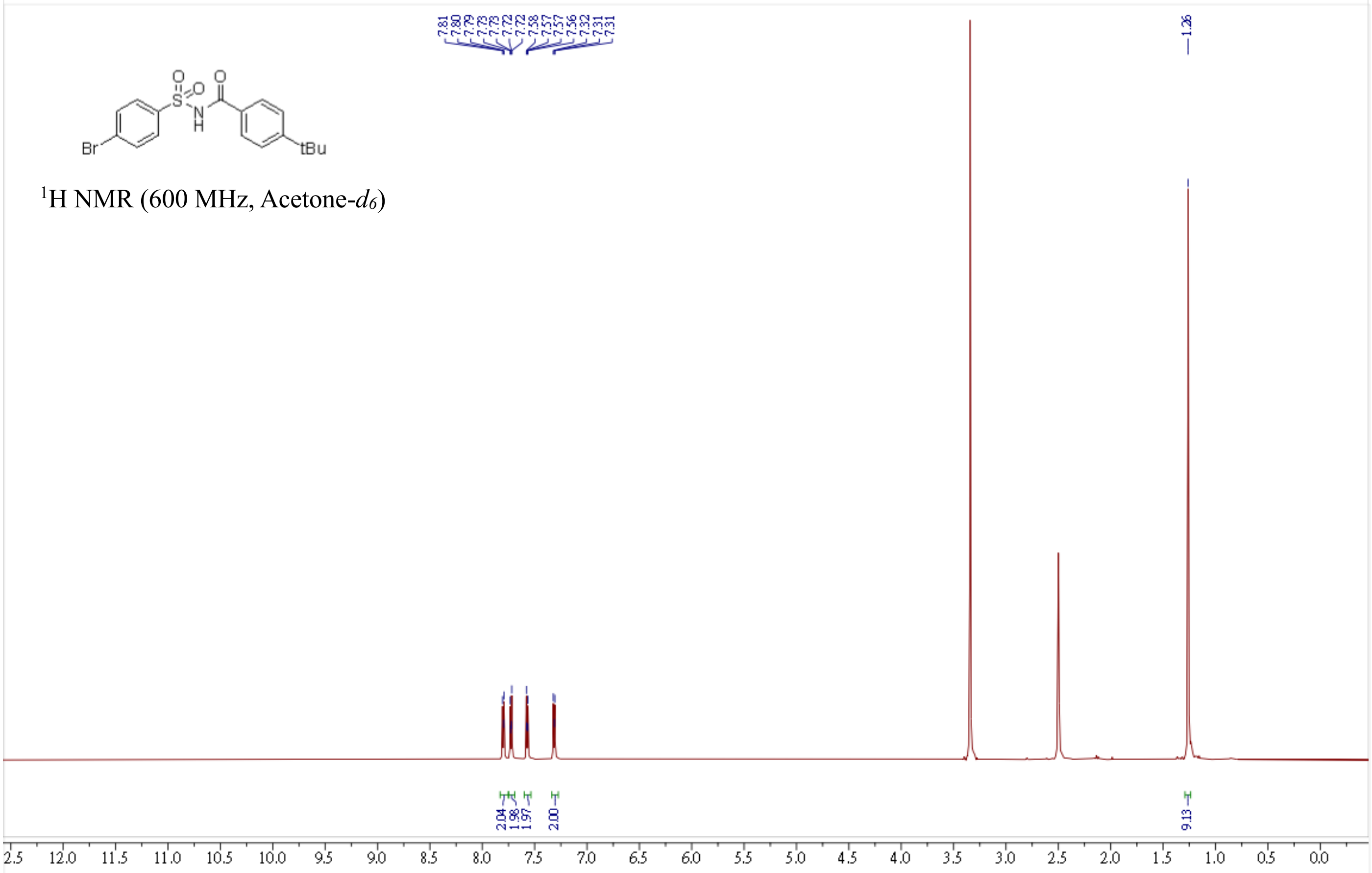

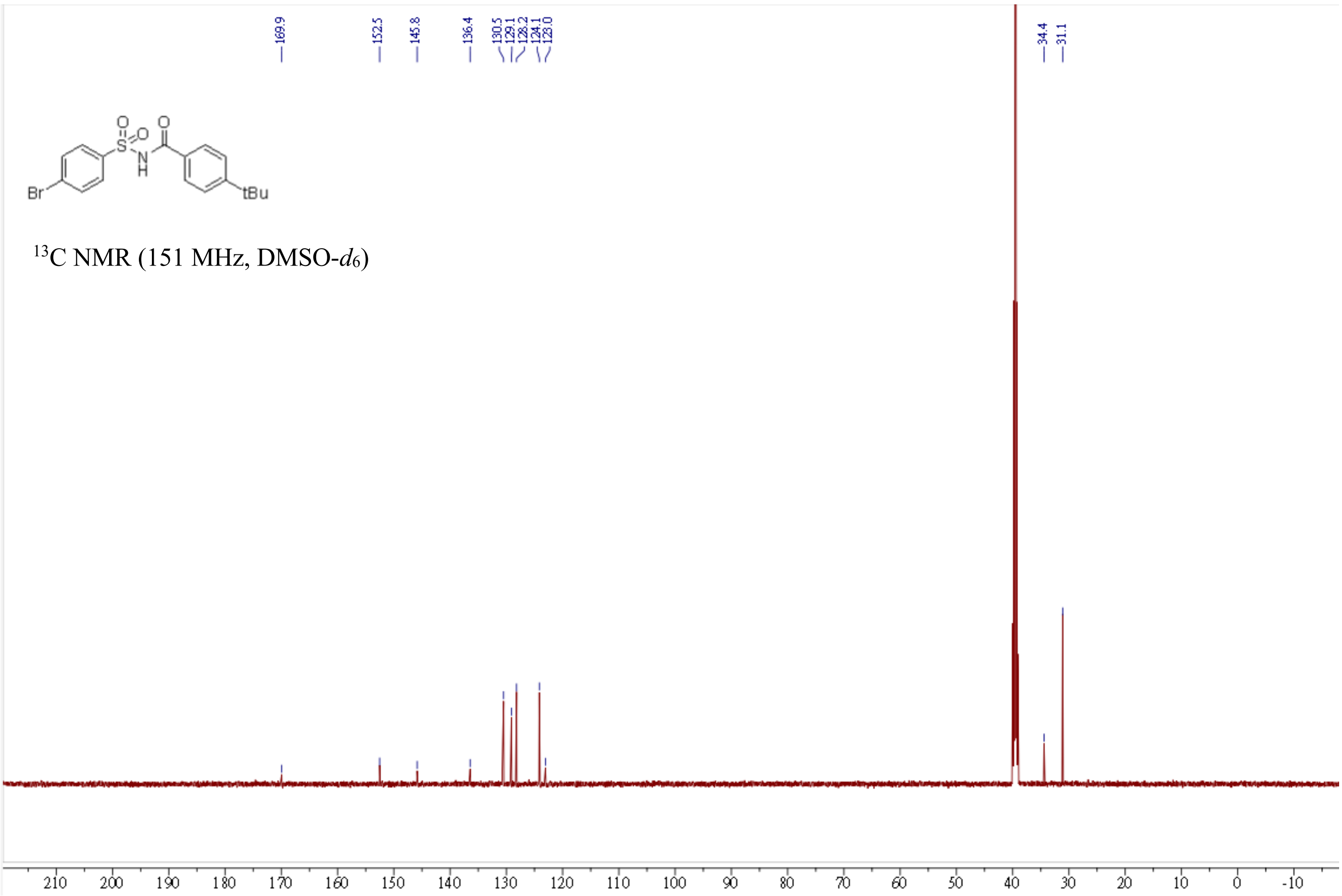

4-(*tert*-butyl)-*N*-((4-methoxyphenyl)sulfonyl)benzamide (3t)

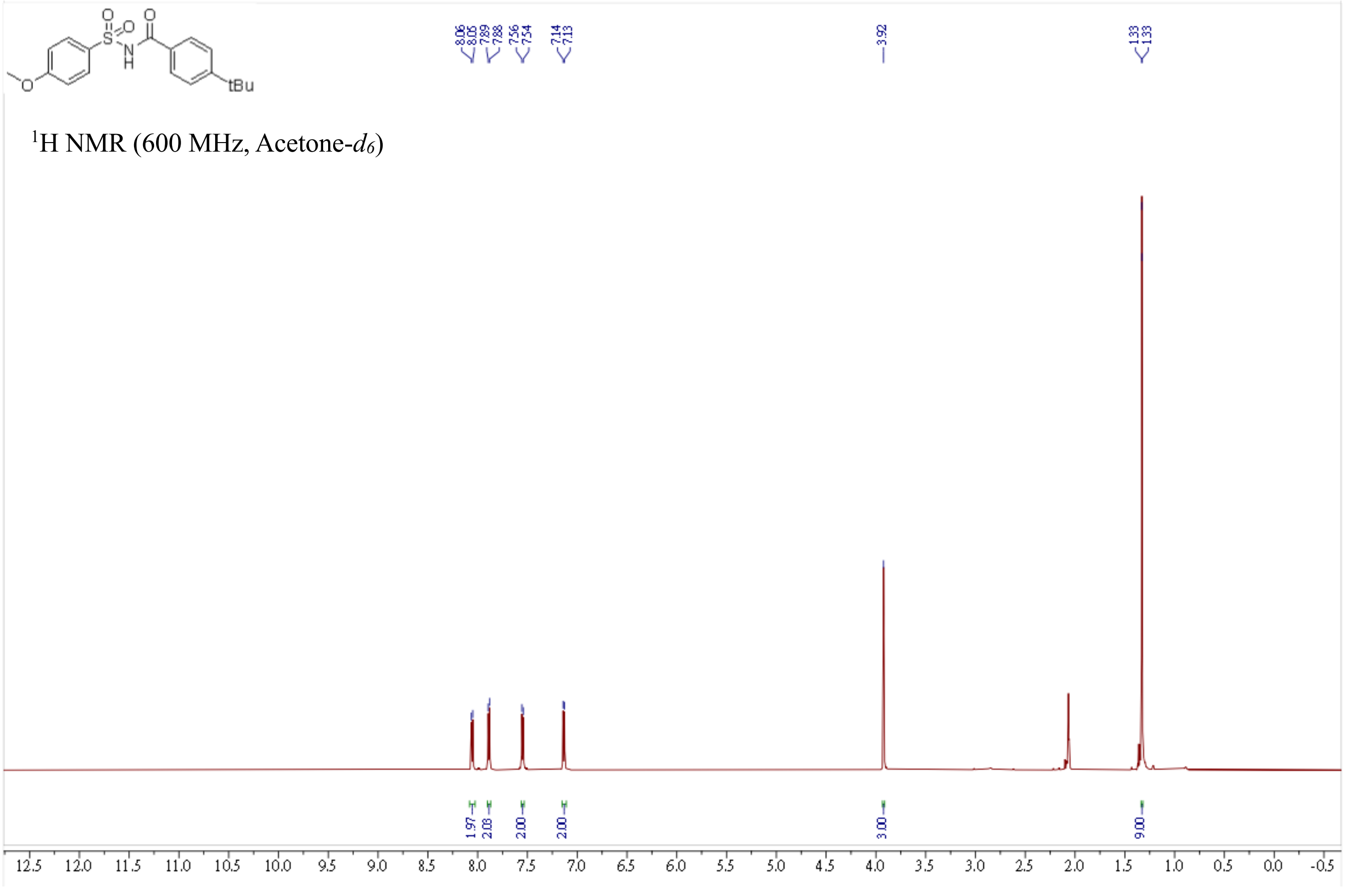

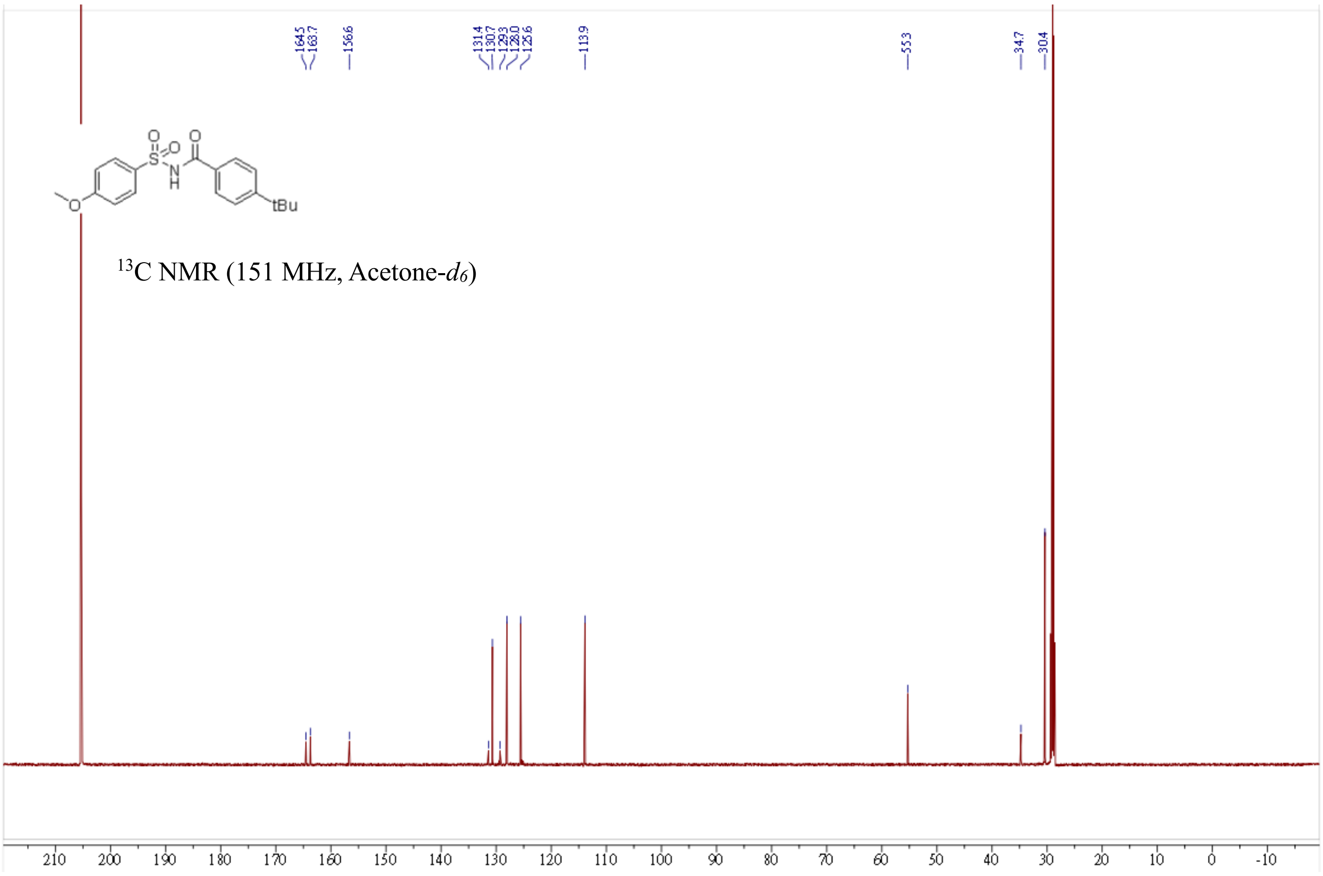

4-(*tert*-butyl)-*N*-(quinolin-8-ylsulfonyl)benzamide (3u)

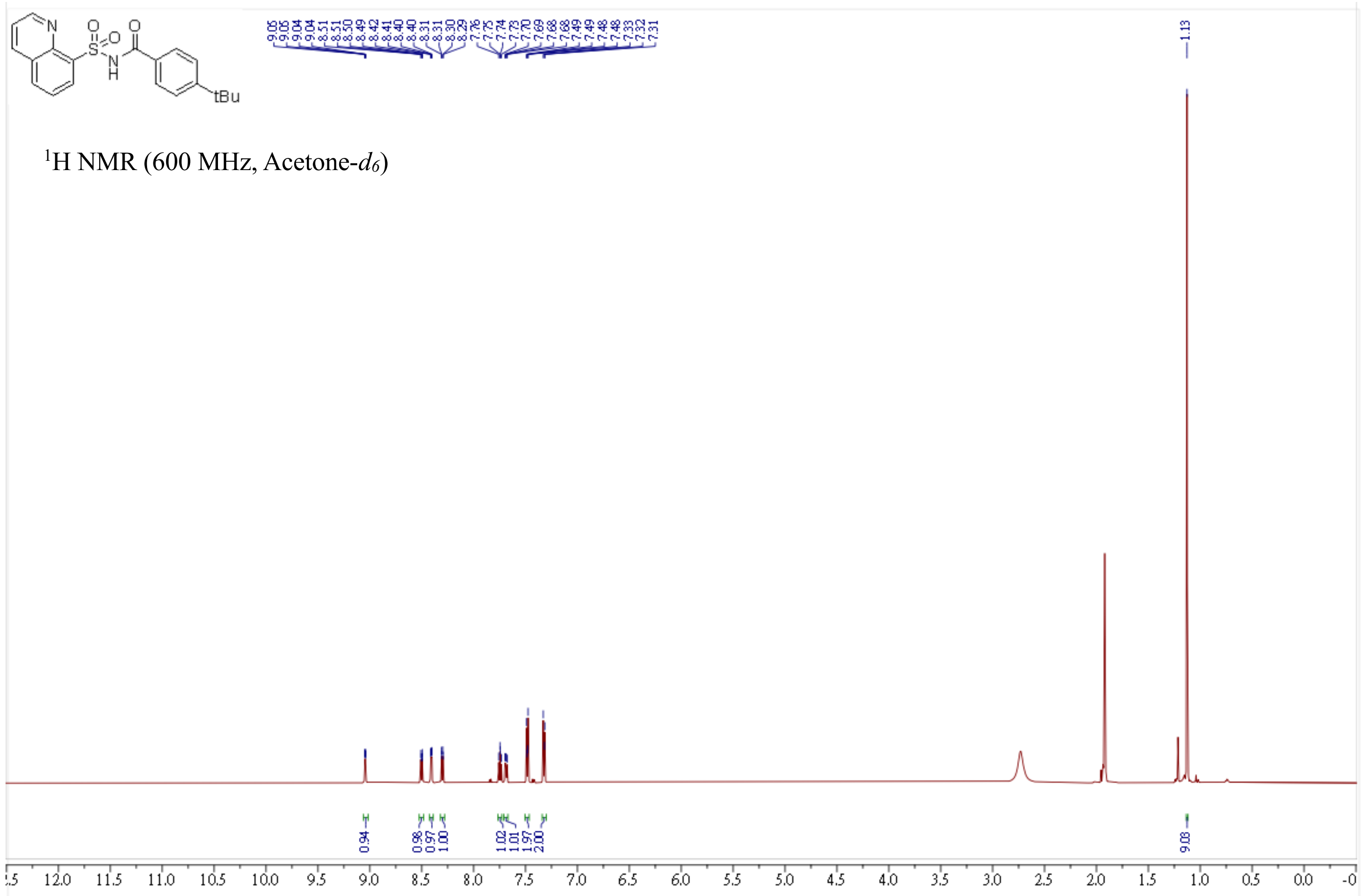

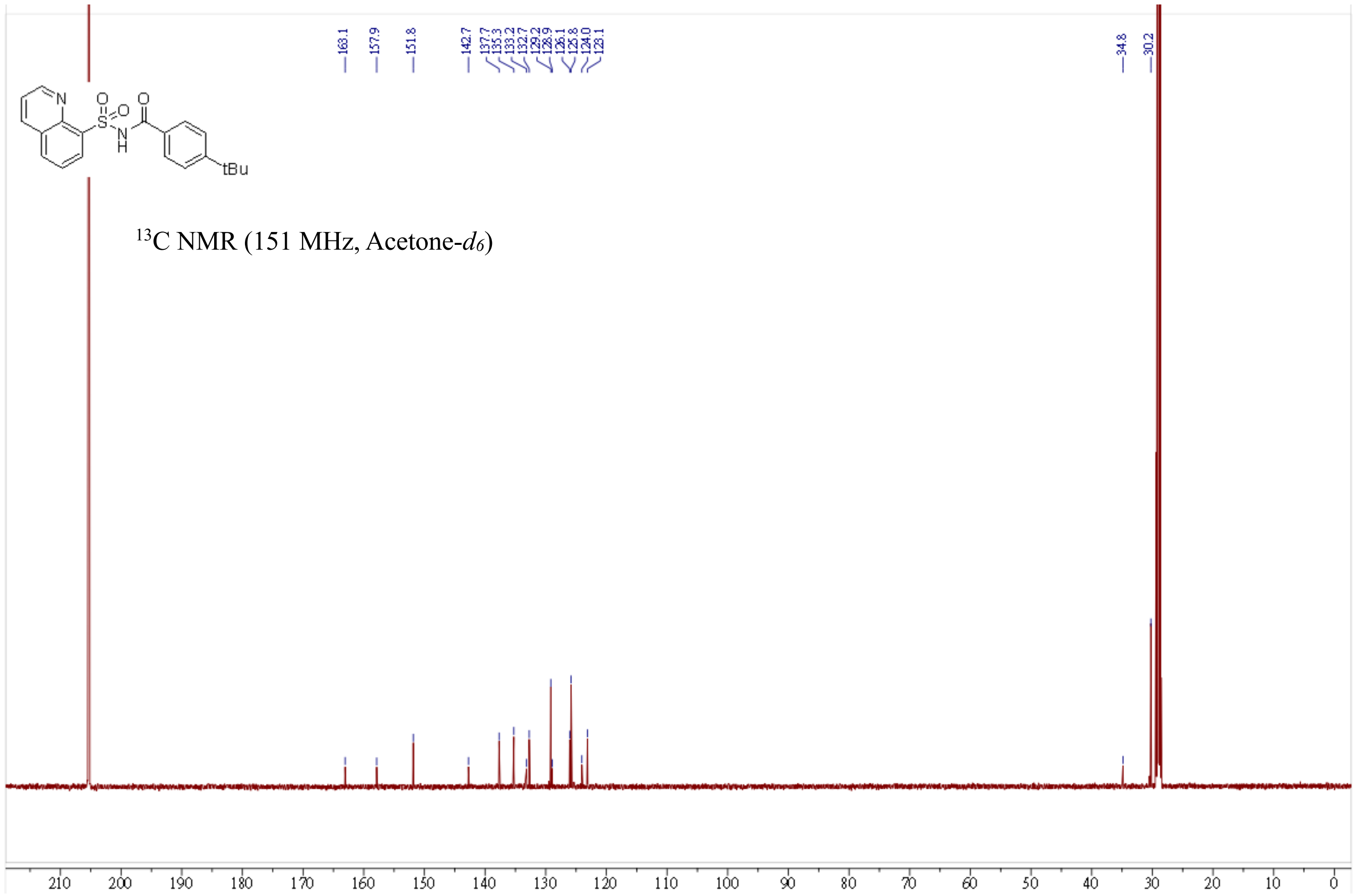

4-(*tert*-butyl)-*N*-((3,5-dimethylisoxazol-4-yl)sulfonyl)benzamide (3v)

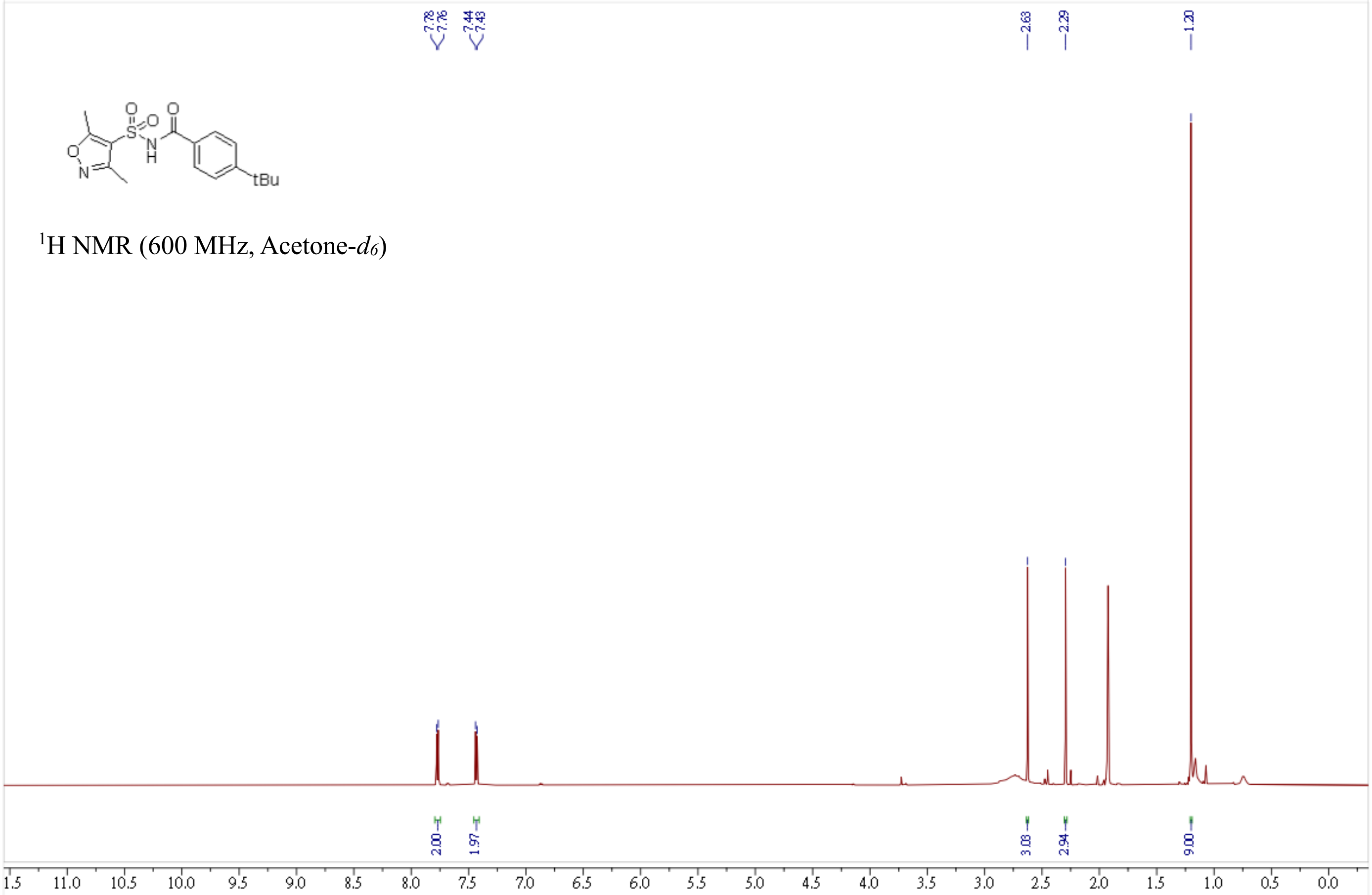

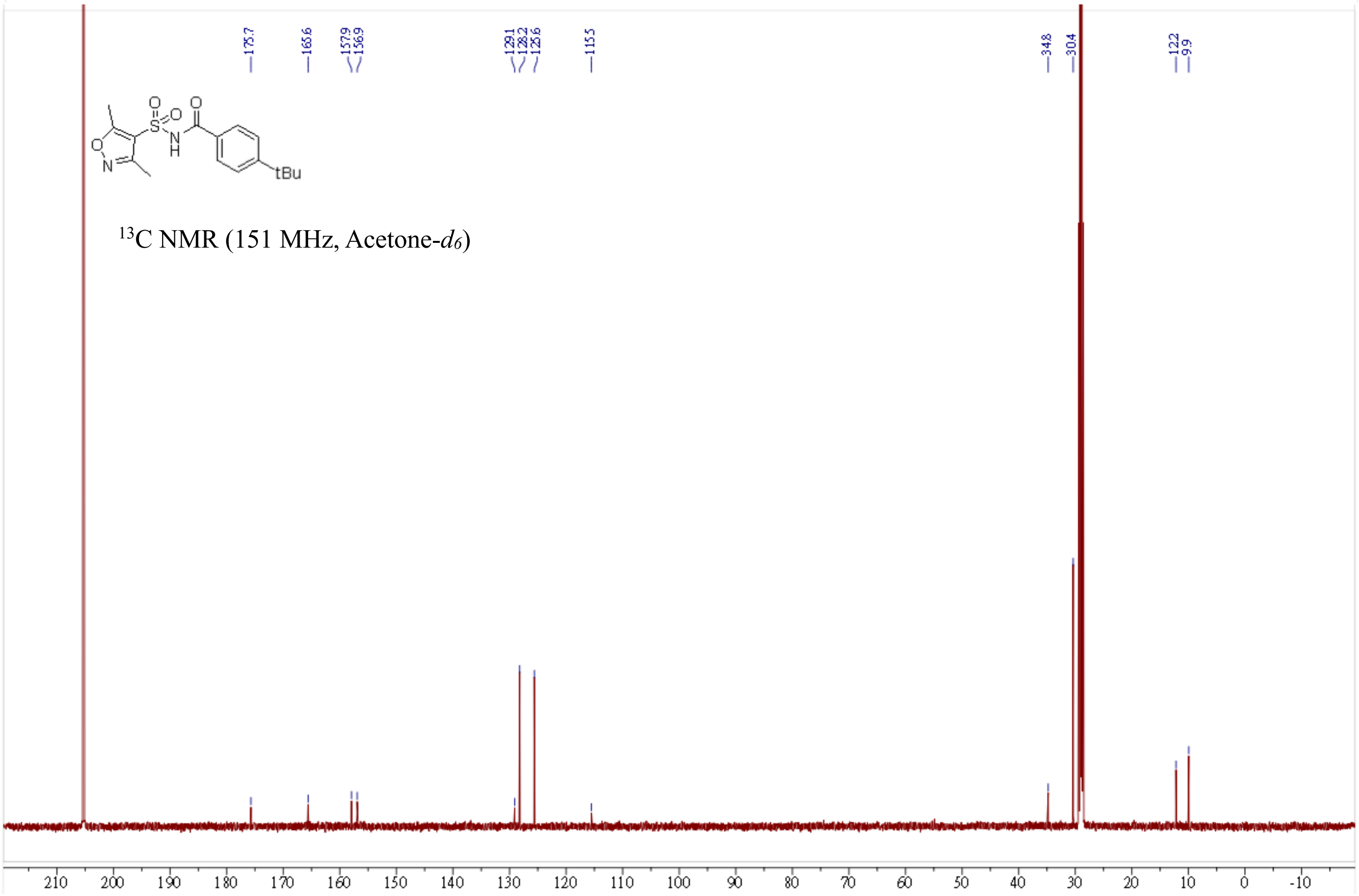

4-(*tert*-butyl)-*N*-(ethylsulfonyl)benzamide (3w)

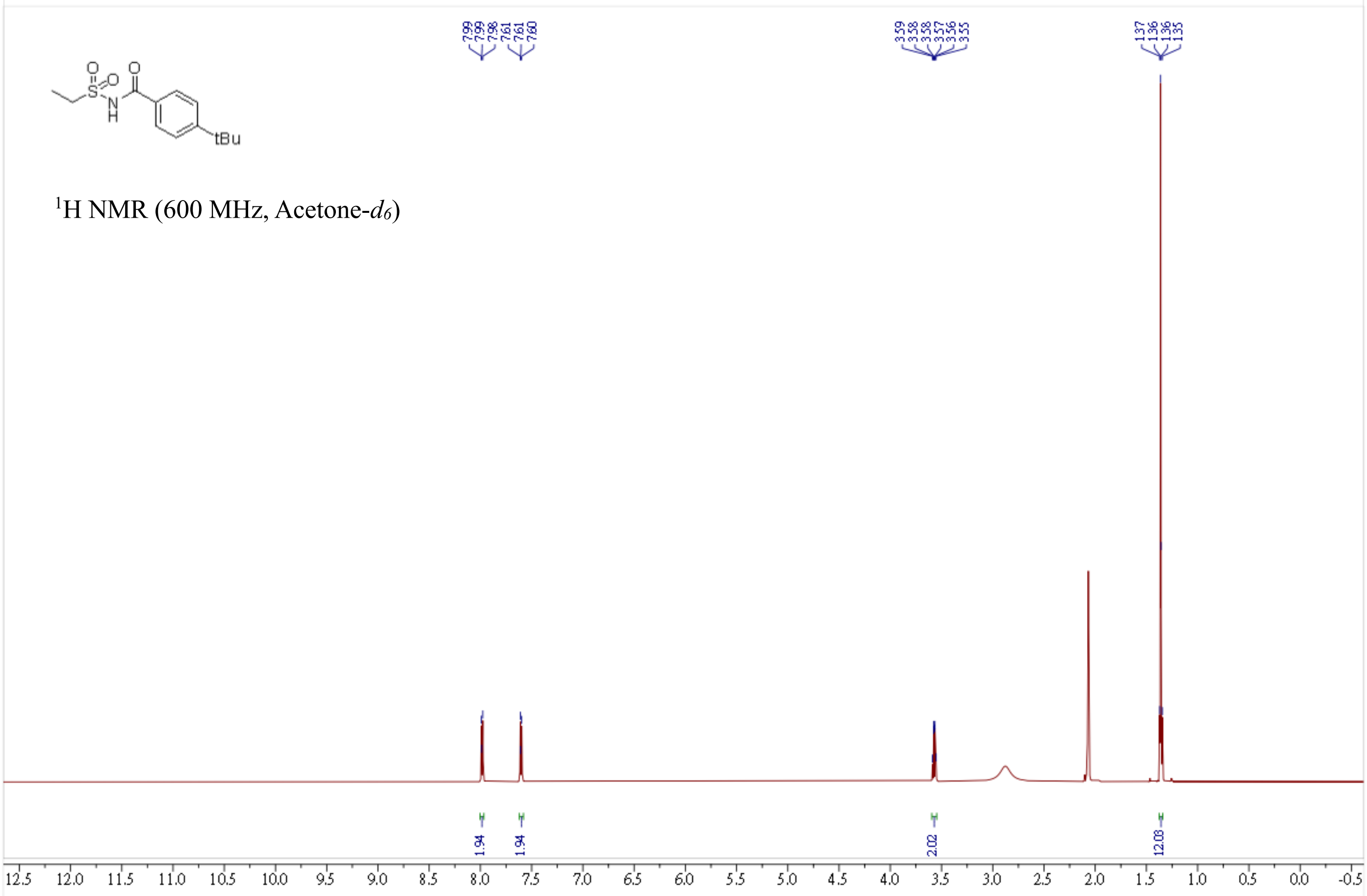

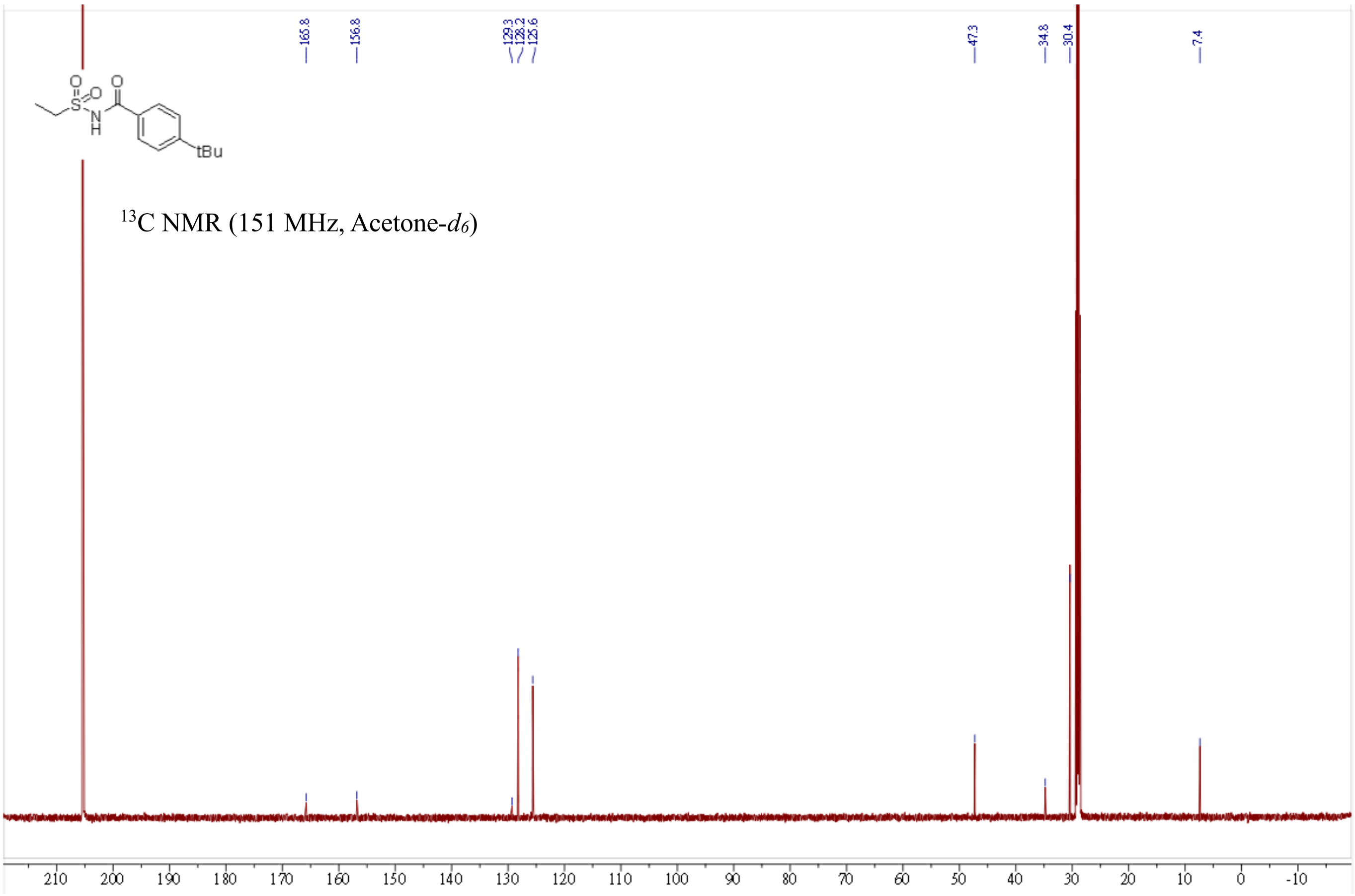

methyl 3-(*N*-(4-(*tert*-butyl)benzoyl)sulfamoyl)propanoate (3x)

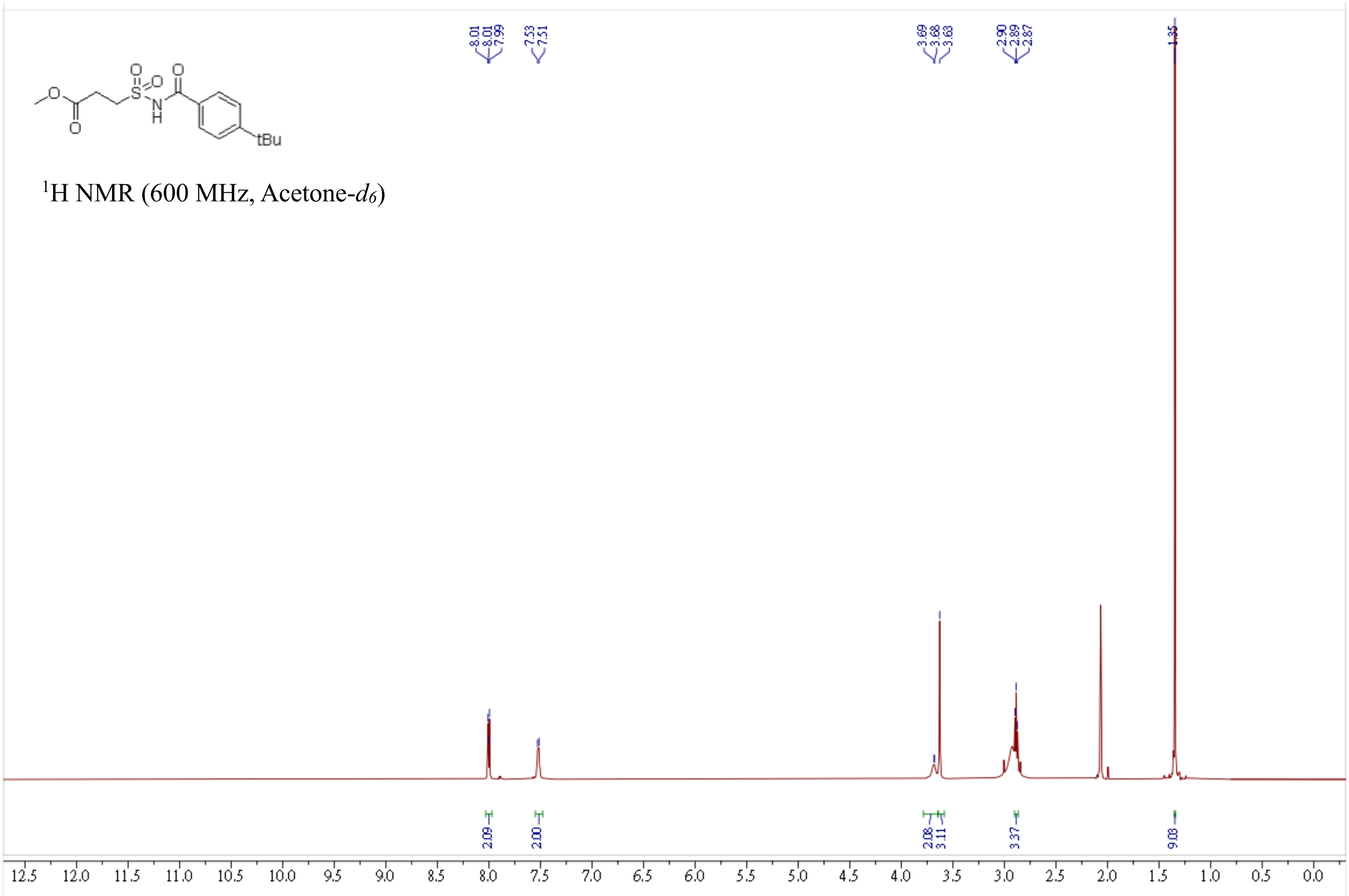

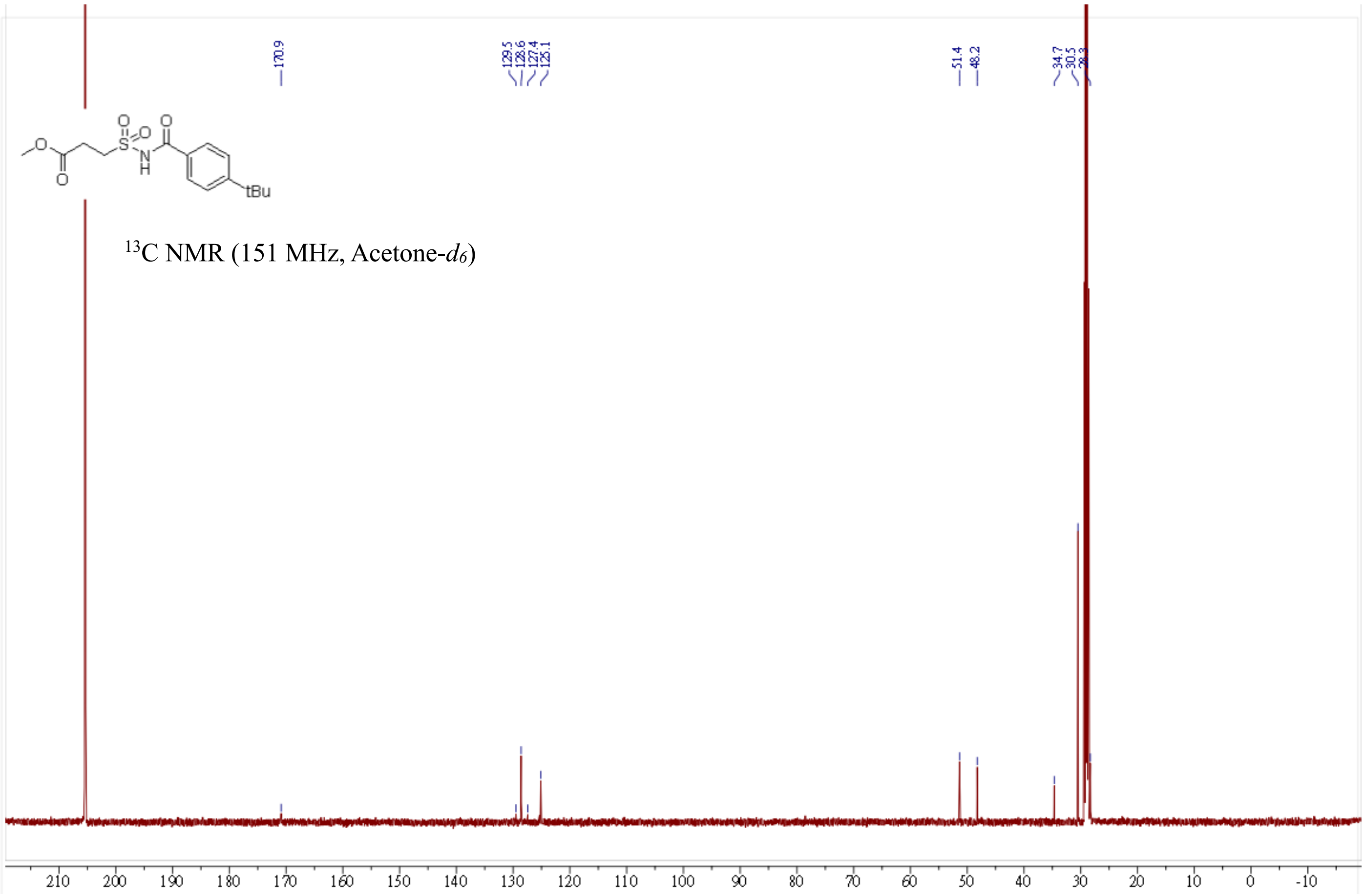

4-(*tert*-butyl)-*N*-(cyclopropylsulfonyl)benzamide (3y)

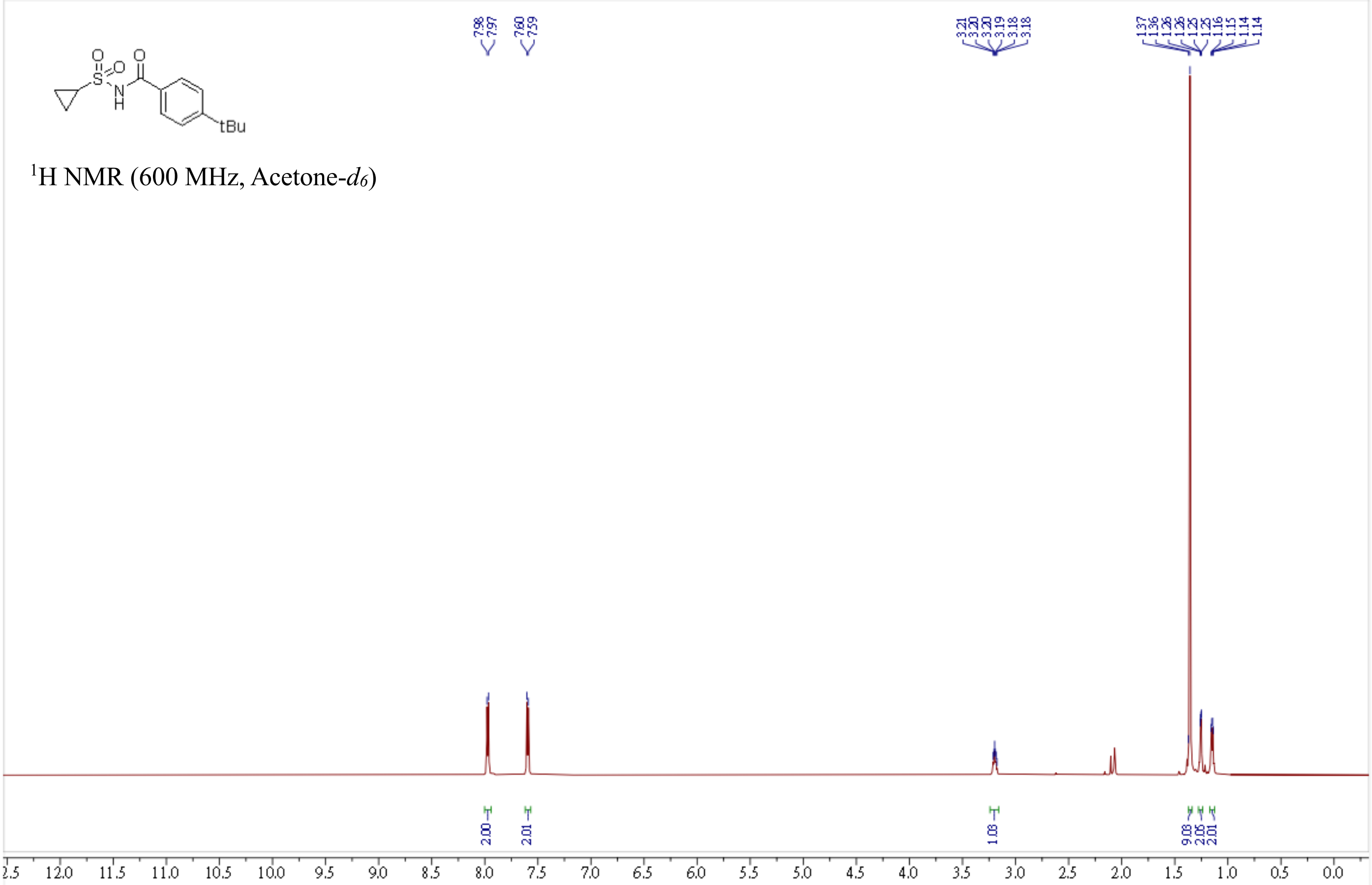

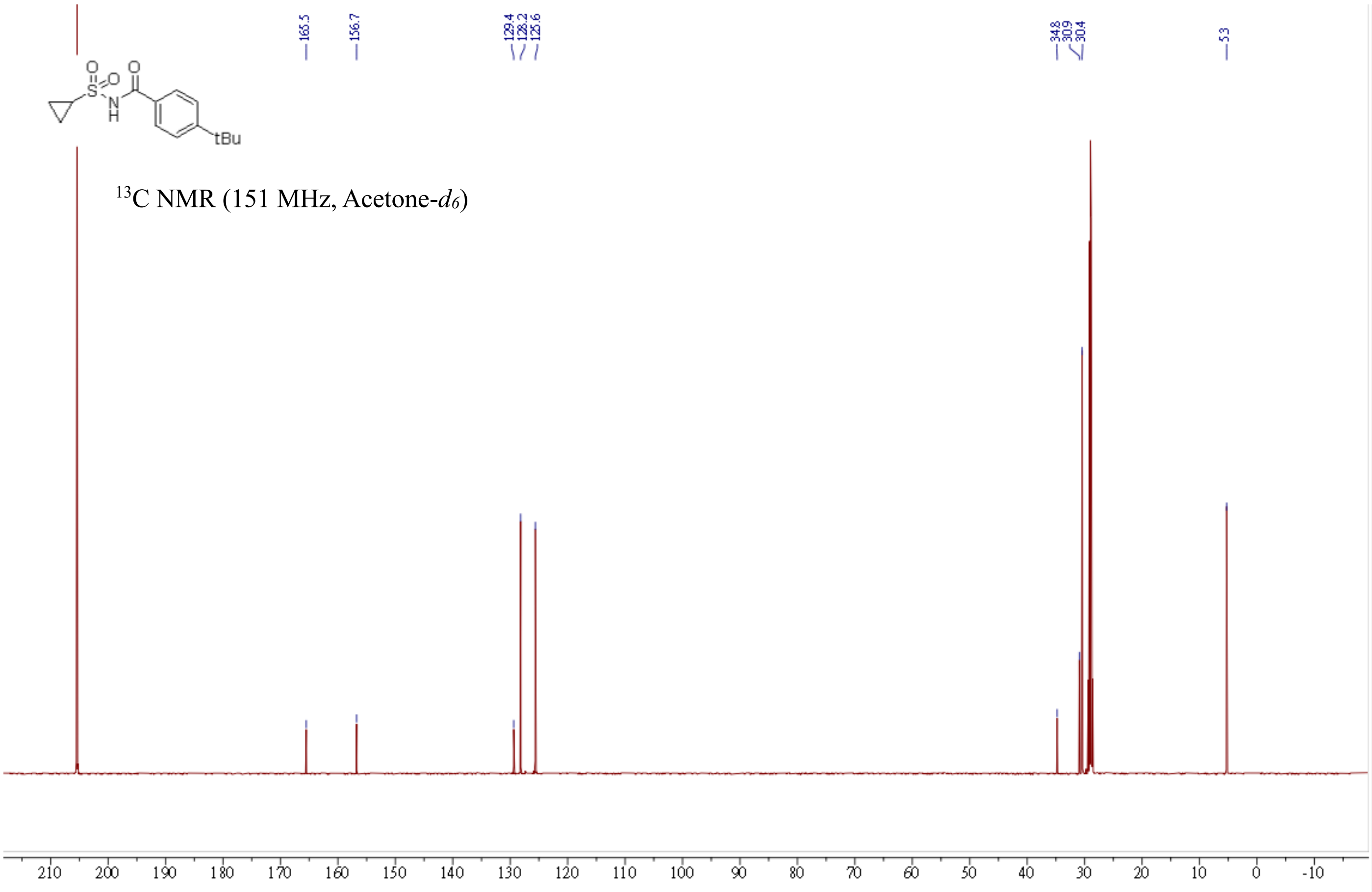

4-(*tert*-butyl)-*N*-((difluoromethyl)sulfonyl)benzamide (3z)

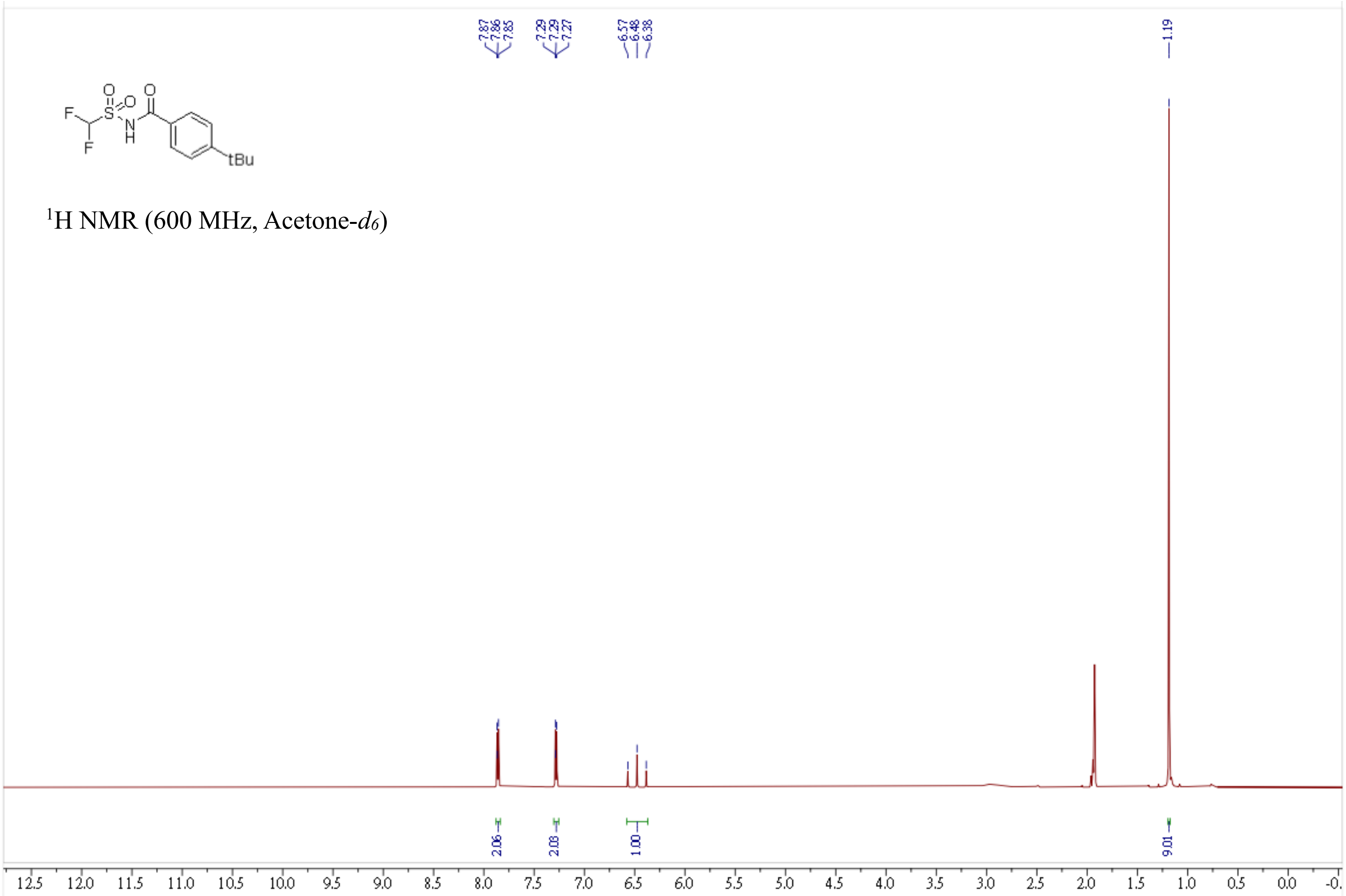

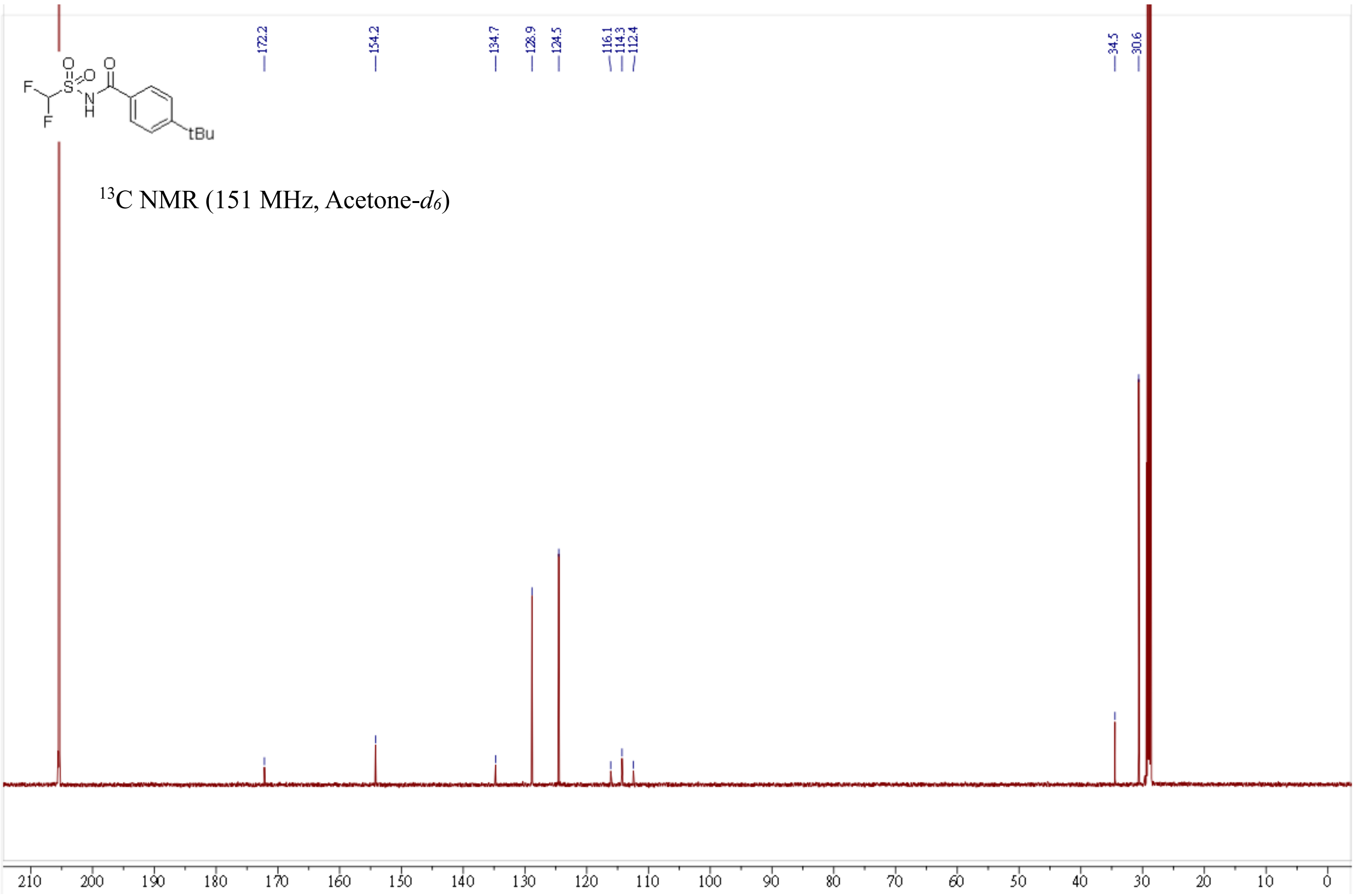

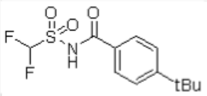

^{19}F NMR (565 MHz, Acetone-d_6)

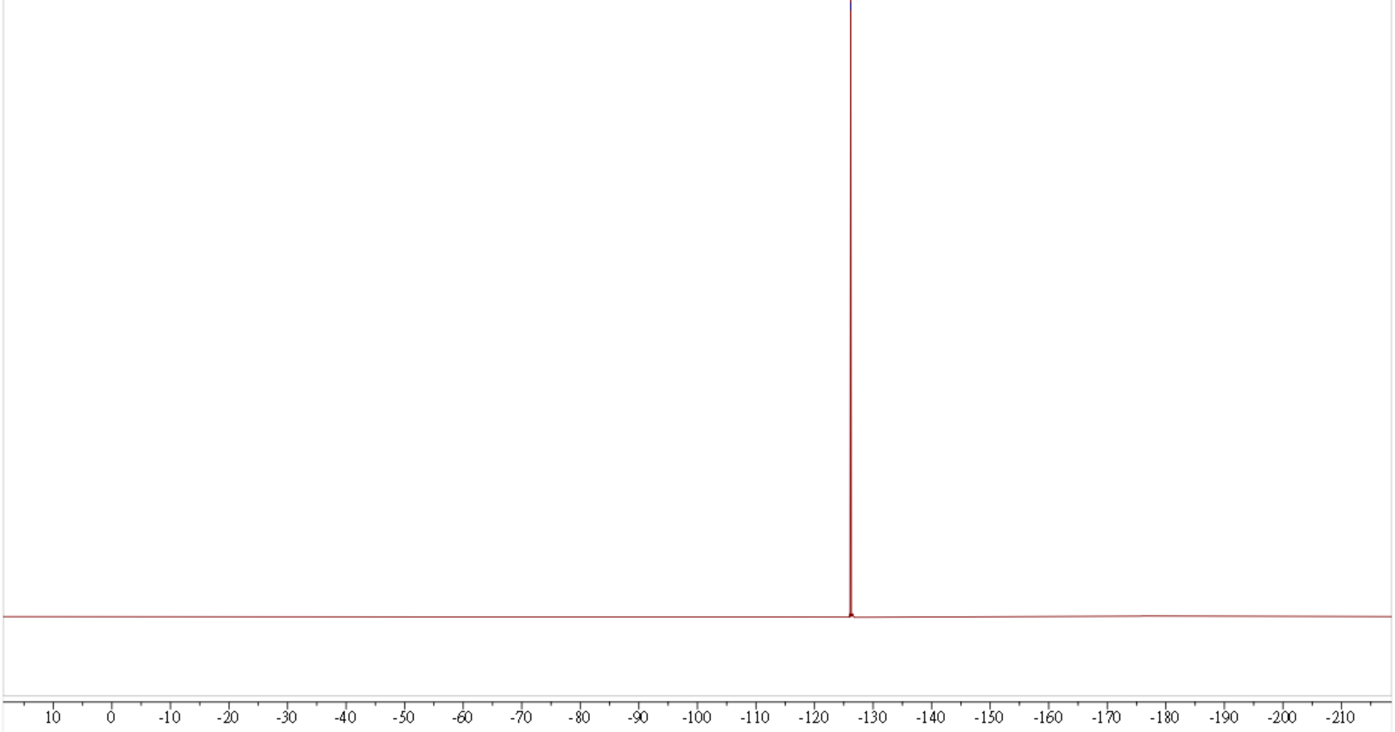

4-(*tert*-butyl)-*N*-(((7,7-dimethyl-2-oxobicyclo[2.2.1]heptan-1-yl)methyl)sulfonyl)benzamide (3aa)

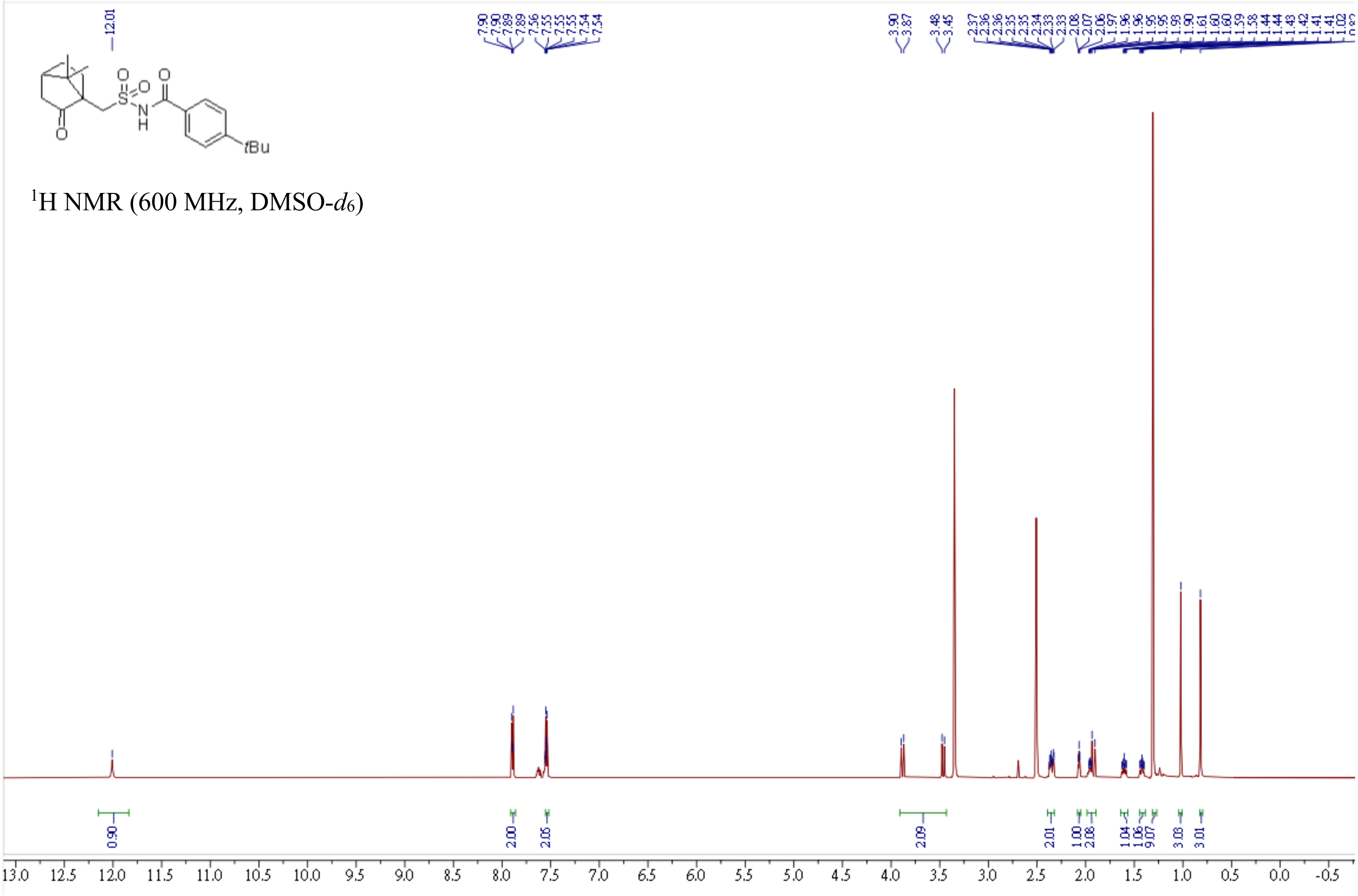

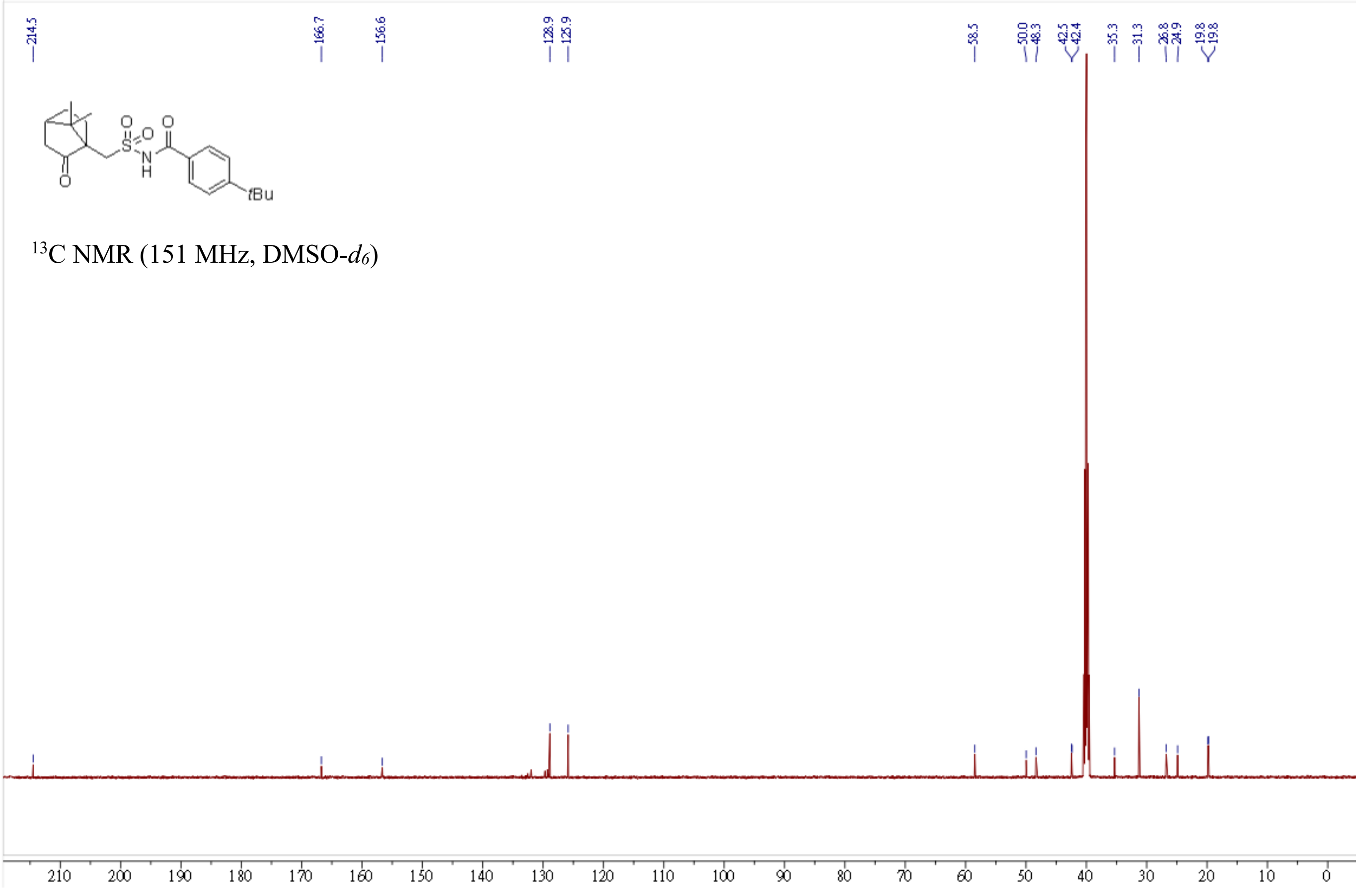

N-(allylsulfonyl)-4-(*tert*-butyl)benzamide (3ab)

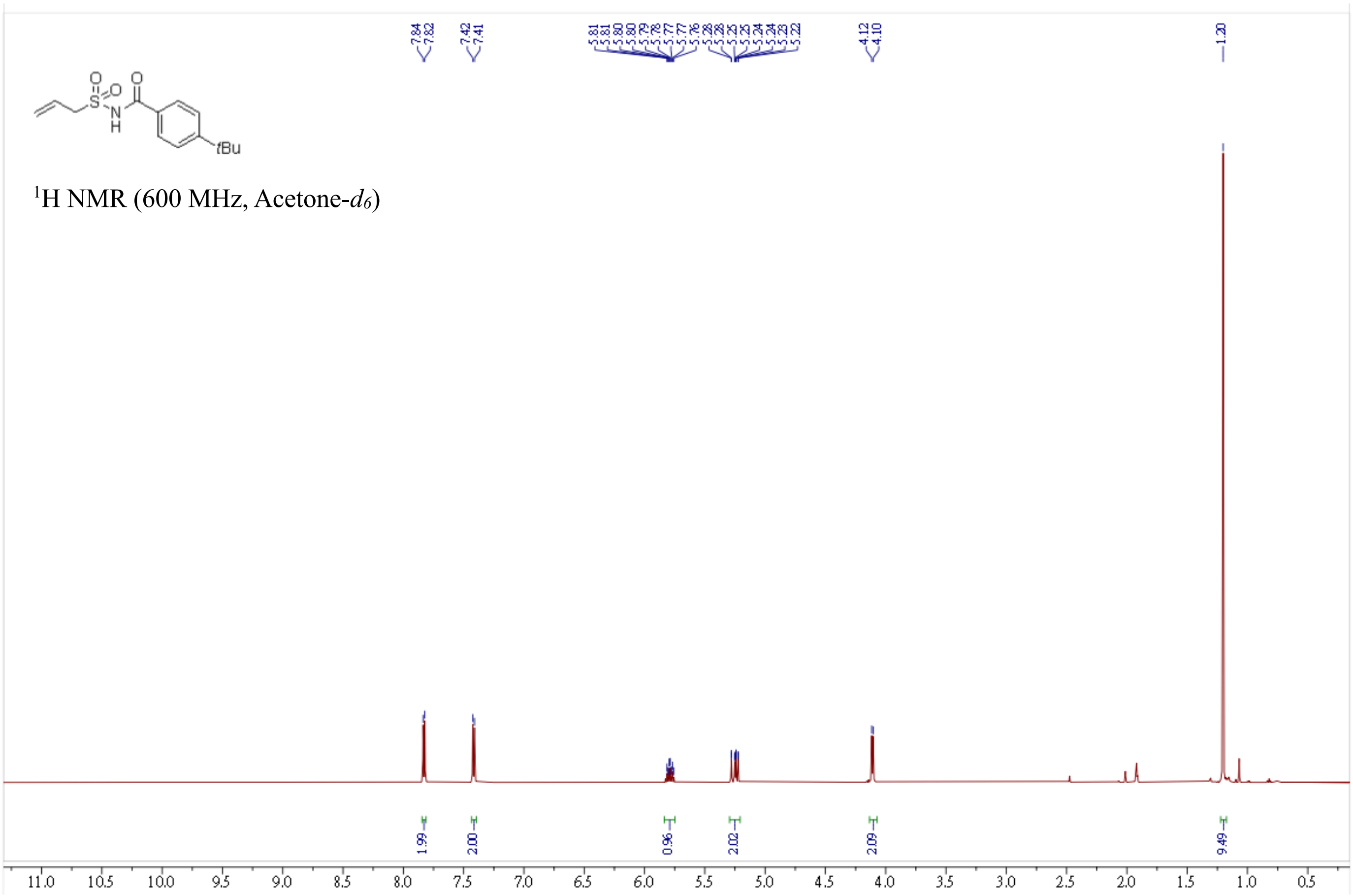

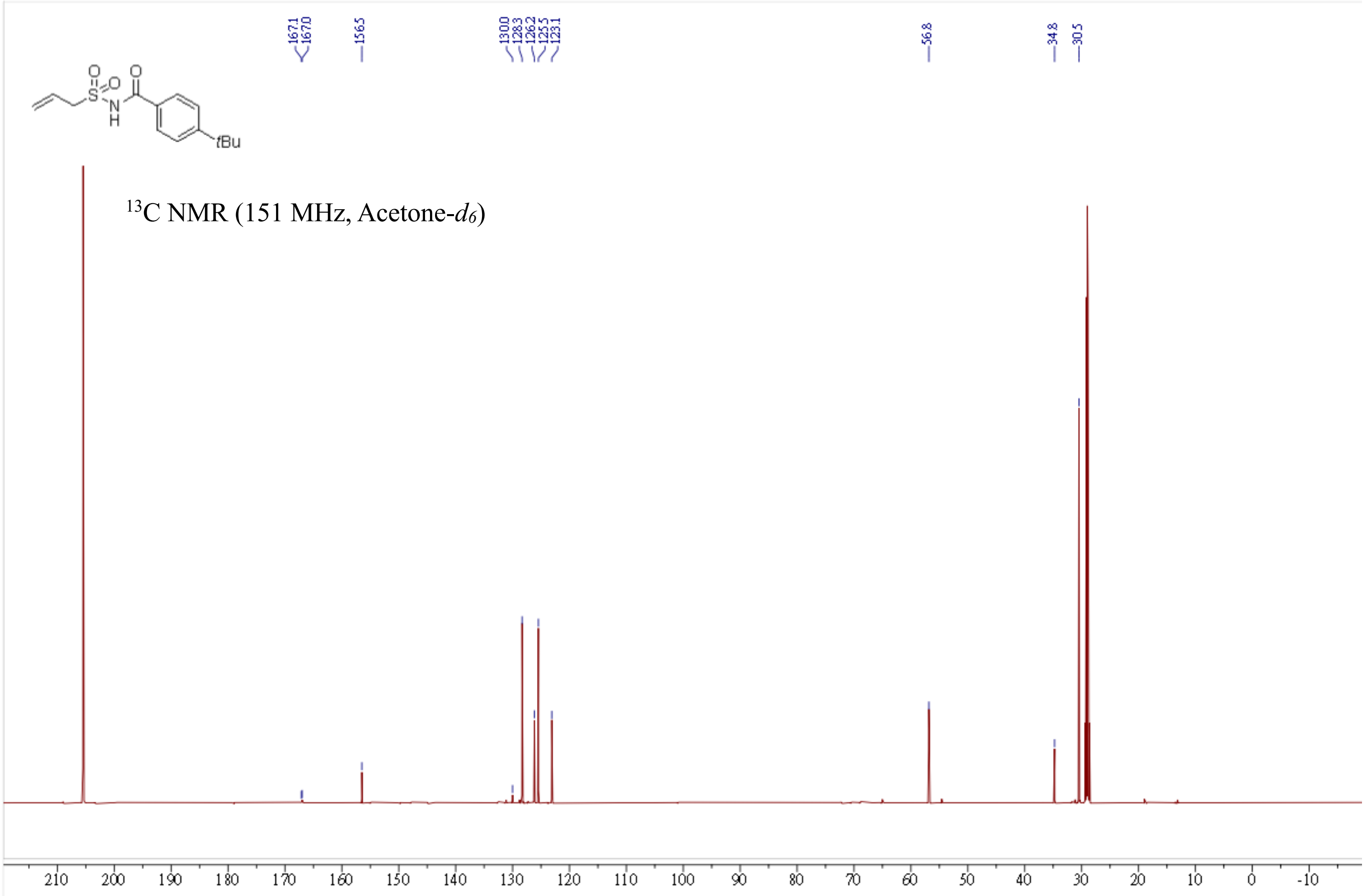

4-(*tert*-butyl)-*N*-((trifluoromethyl)sulfonyl)benzamide (3ac)

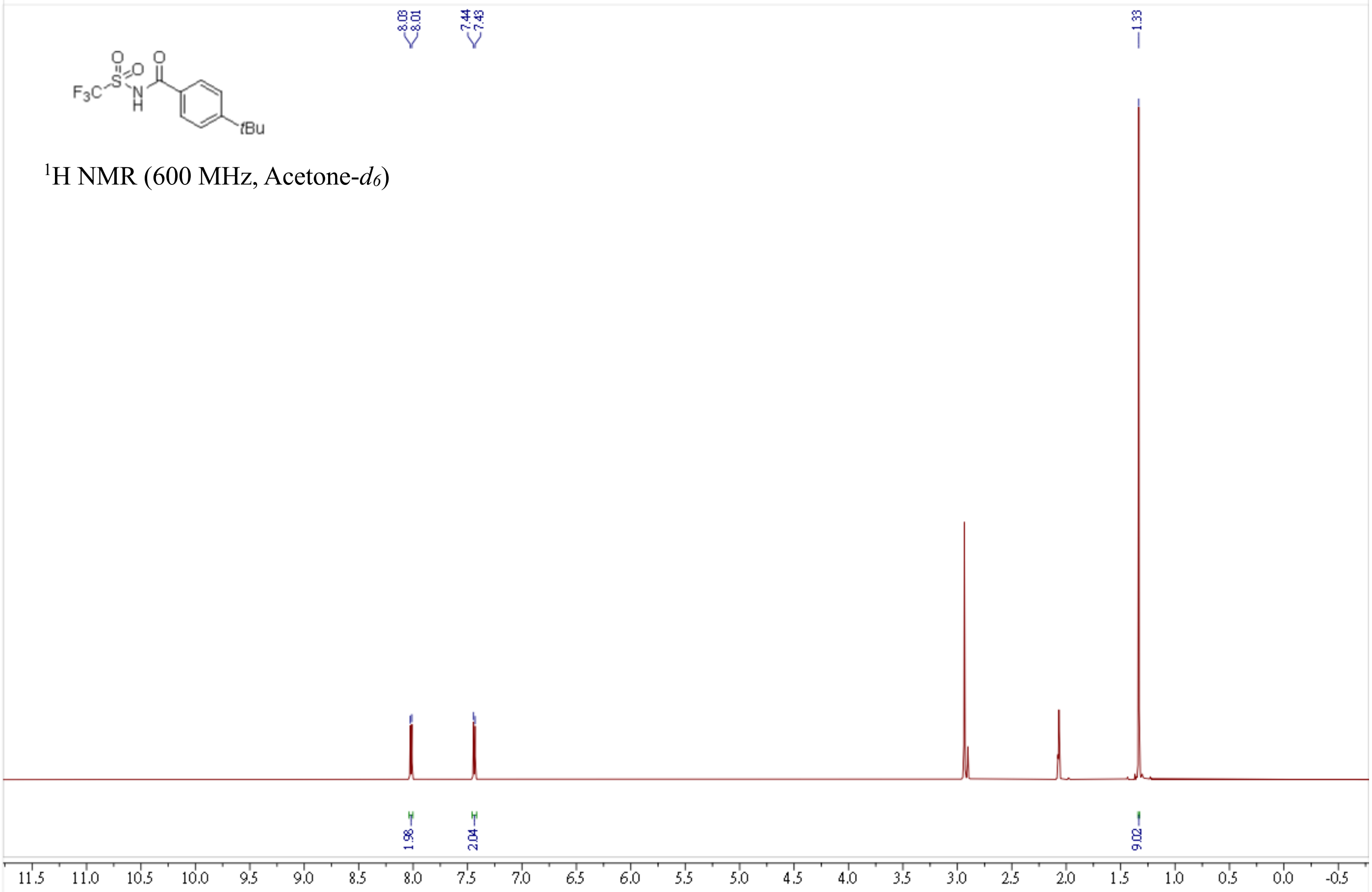

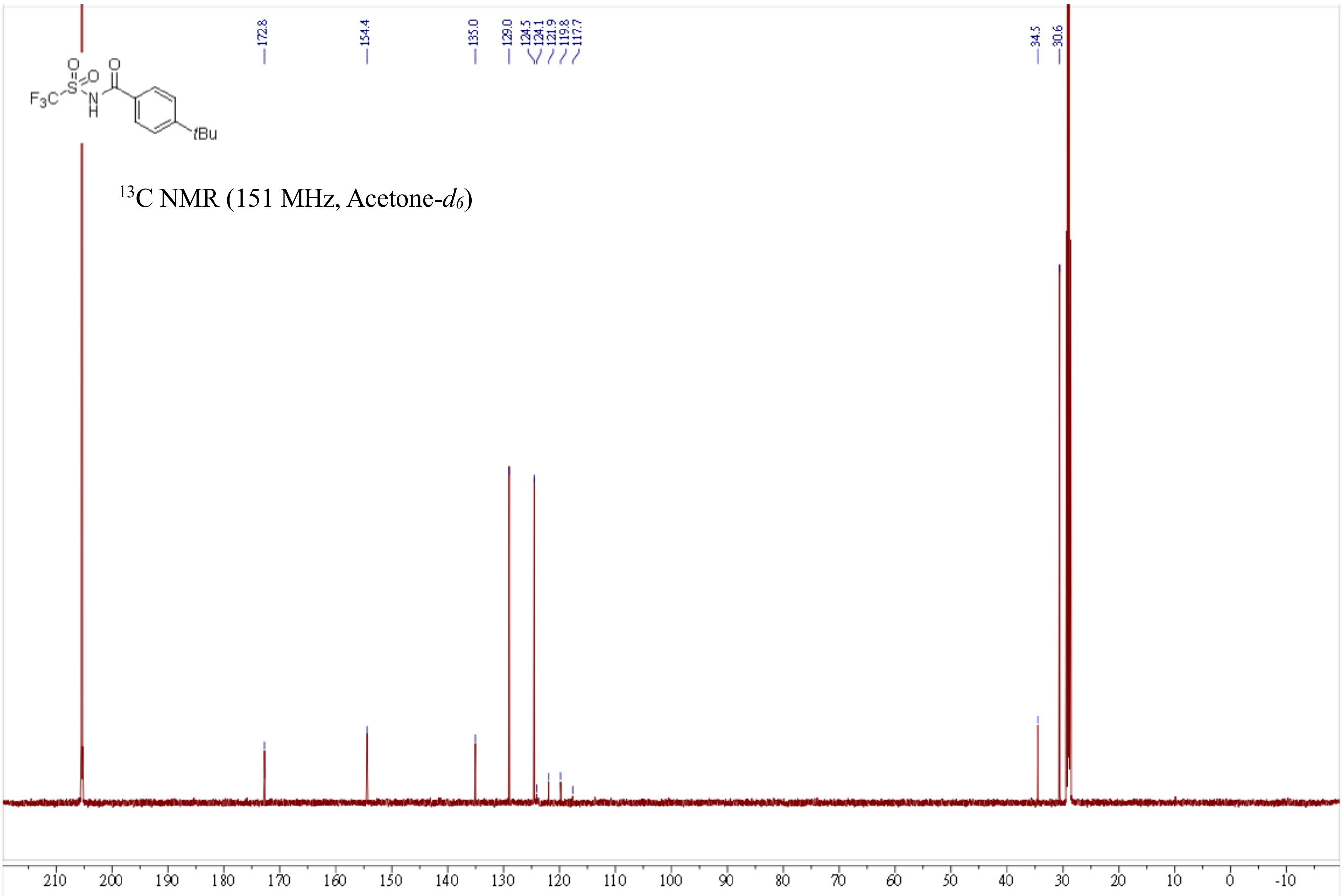

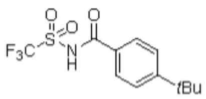

^{19}F NMR (565 MHz, Acetone-d_6)

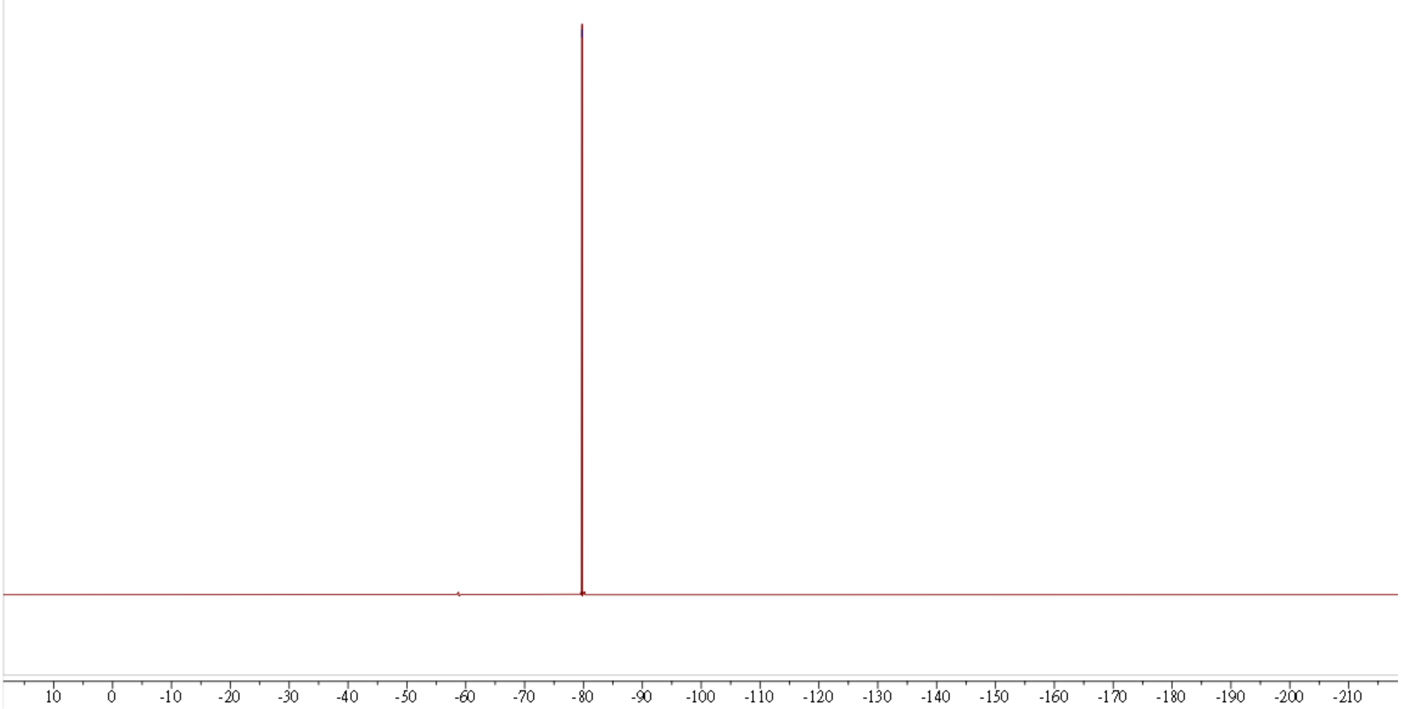

4-(*tert*-butyl)-*N*-((4-cyanophenyl)sulfonyl)benzamide (3ad)

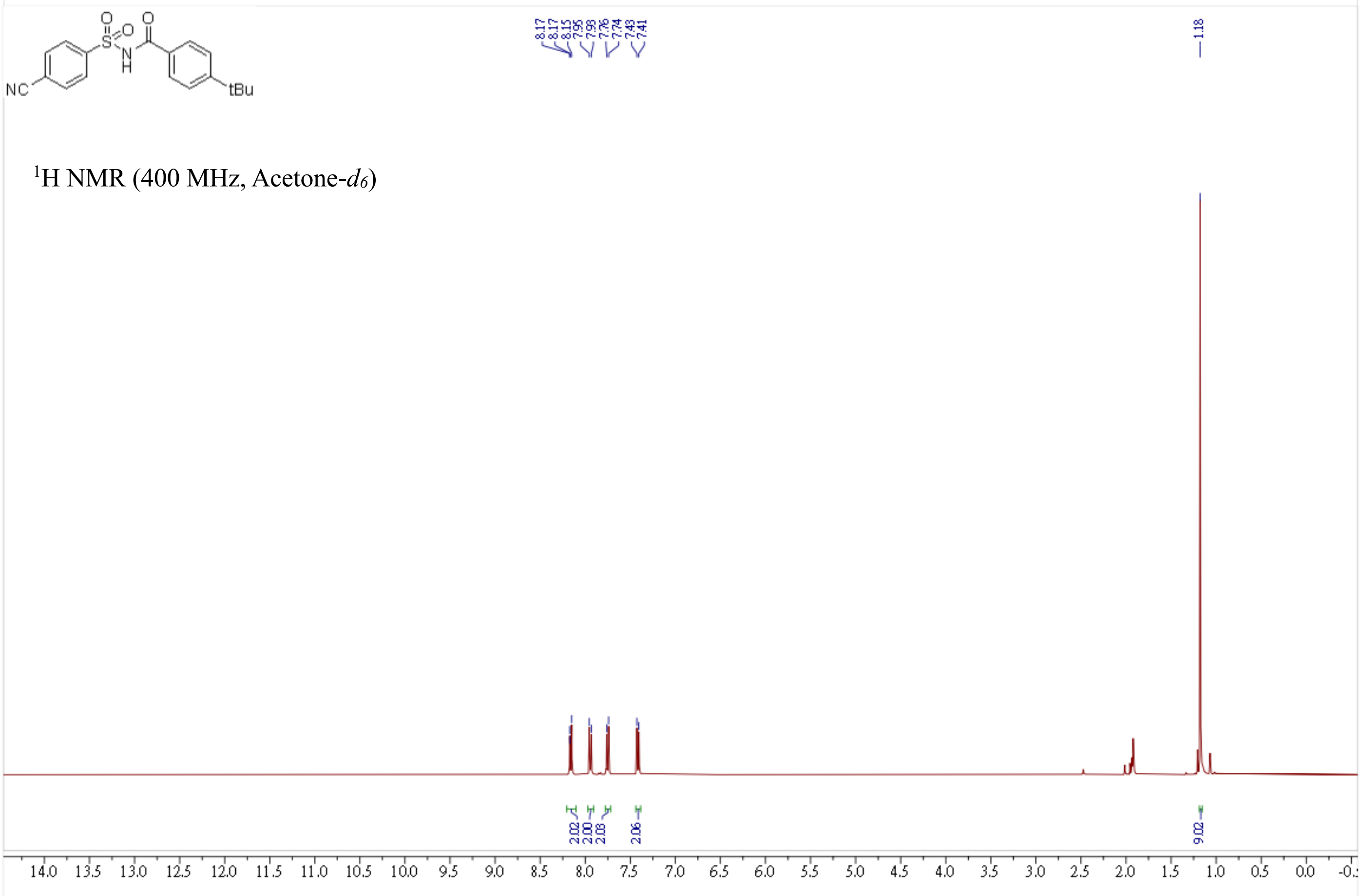

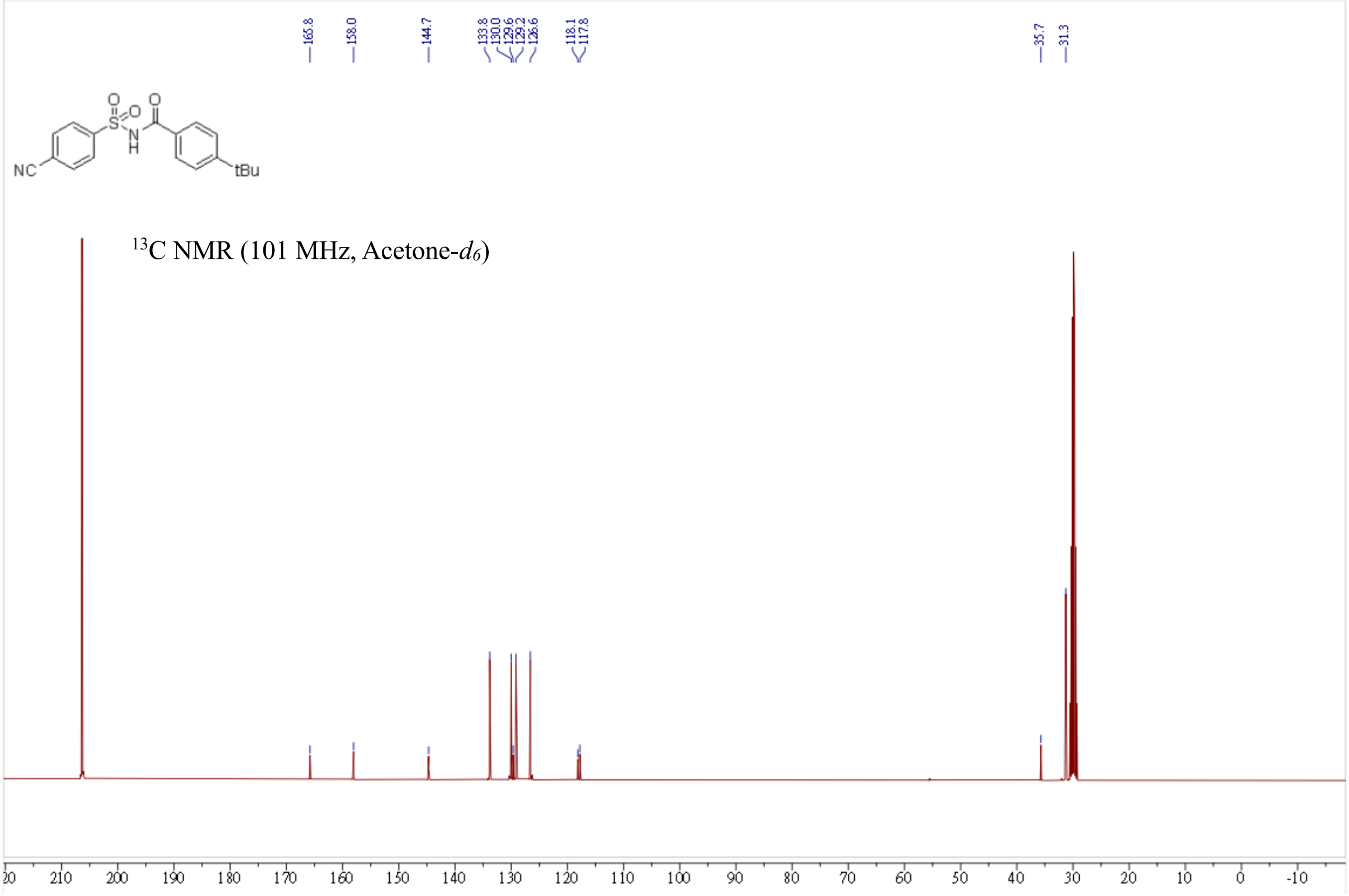

B. Cyano-based Photocatalyst

2,3,5,6-tetra(9H-carbazol-9-yl)benzonitrile (4CzBN)

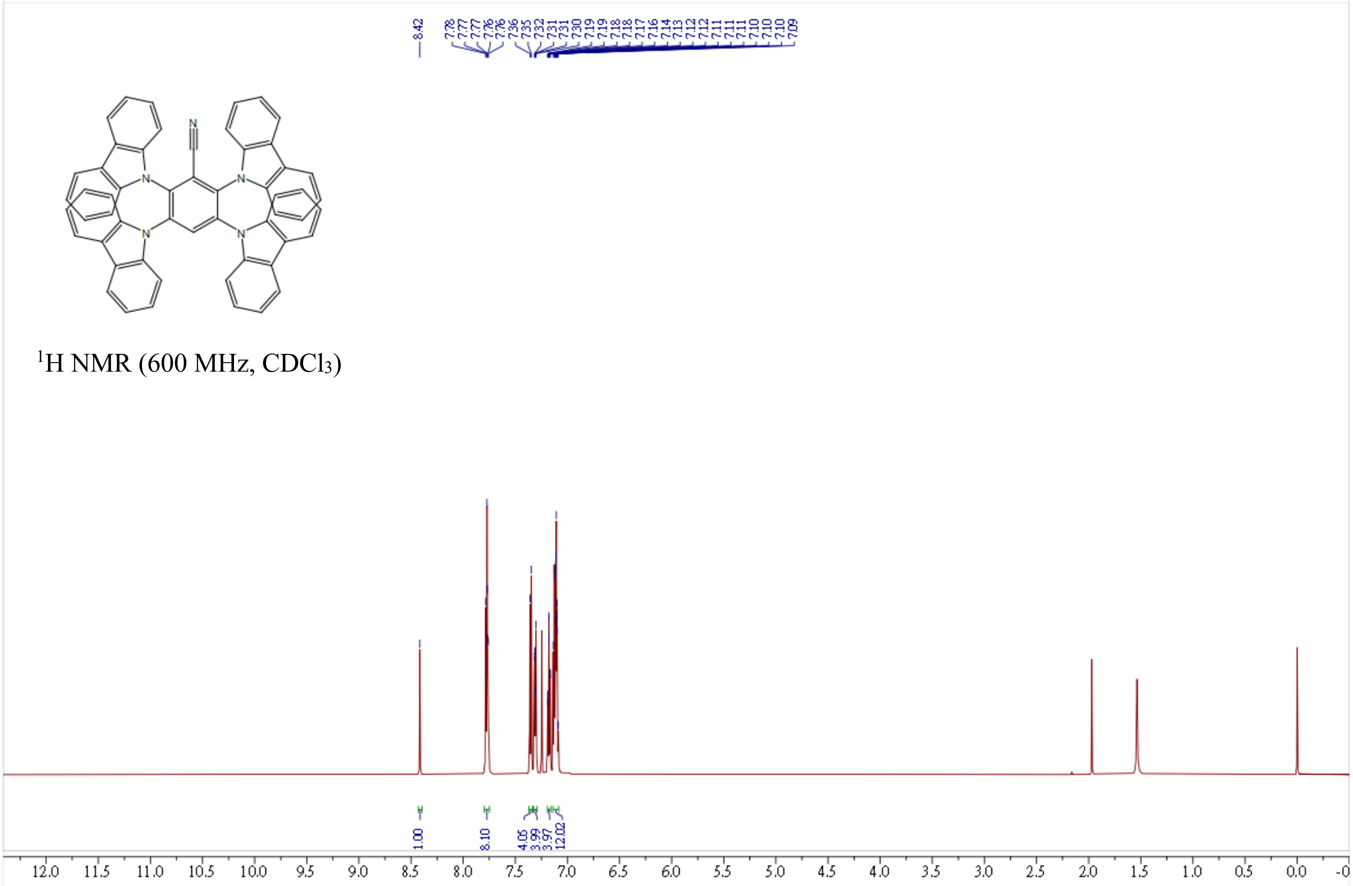

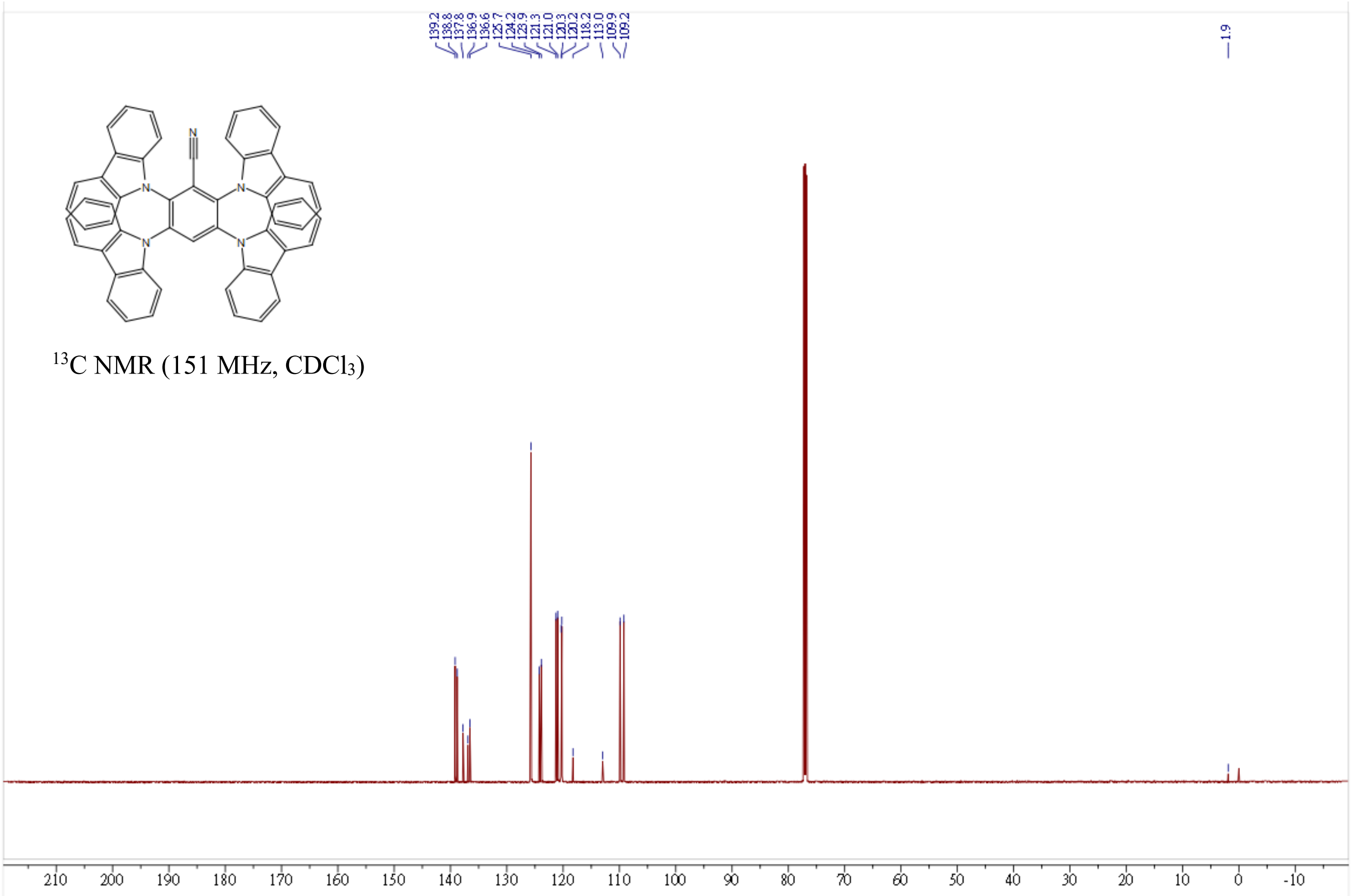

C. Sodium Organosulfinate

Sodium (7,7-dimethyl-2-oxobicyclo[2.2.1]heptan-1-yl)methanesulfinate

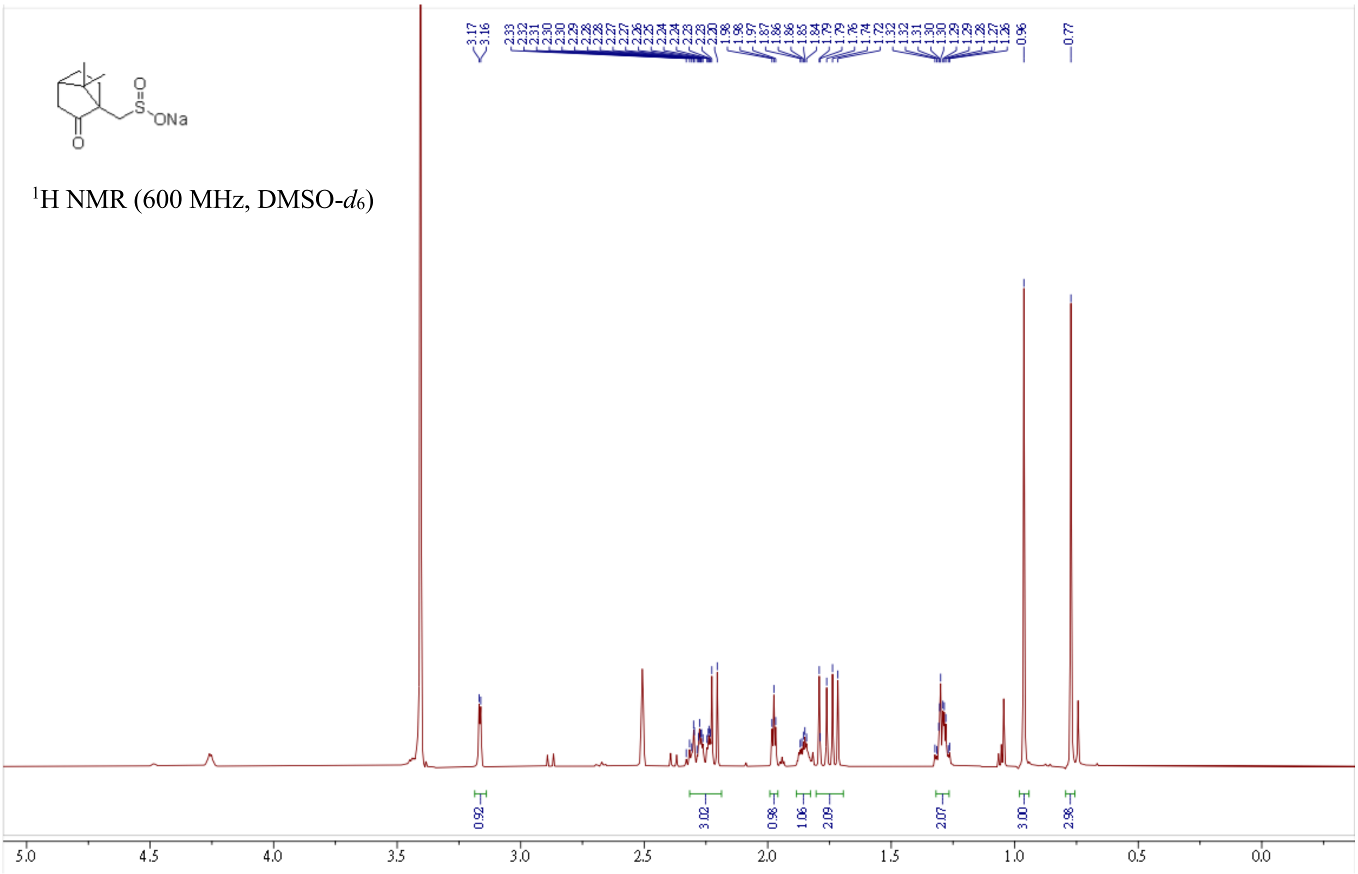

D. Benzohydroxamic acids / Hydroxycarbamates

_N_-hydroxy-4-methoxybenzamide (1a)

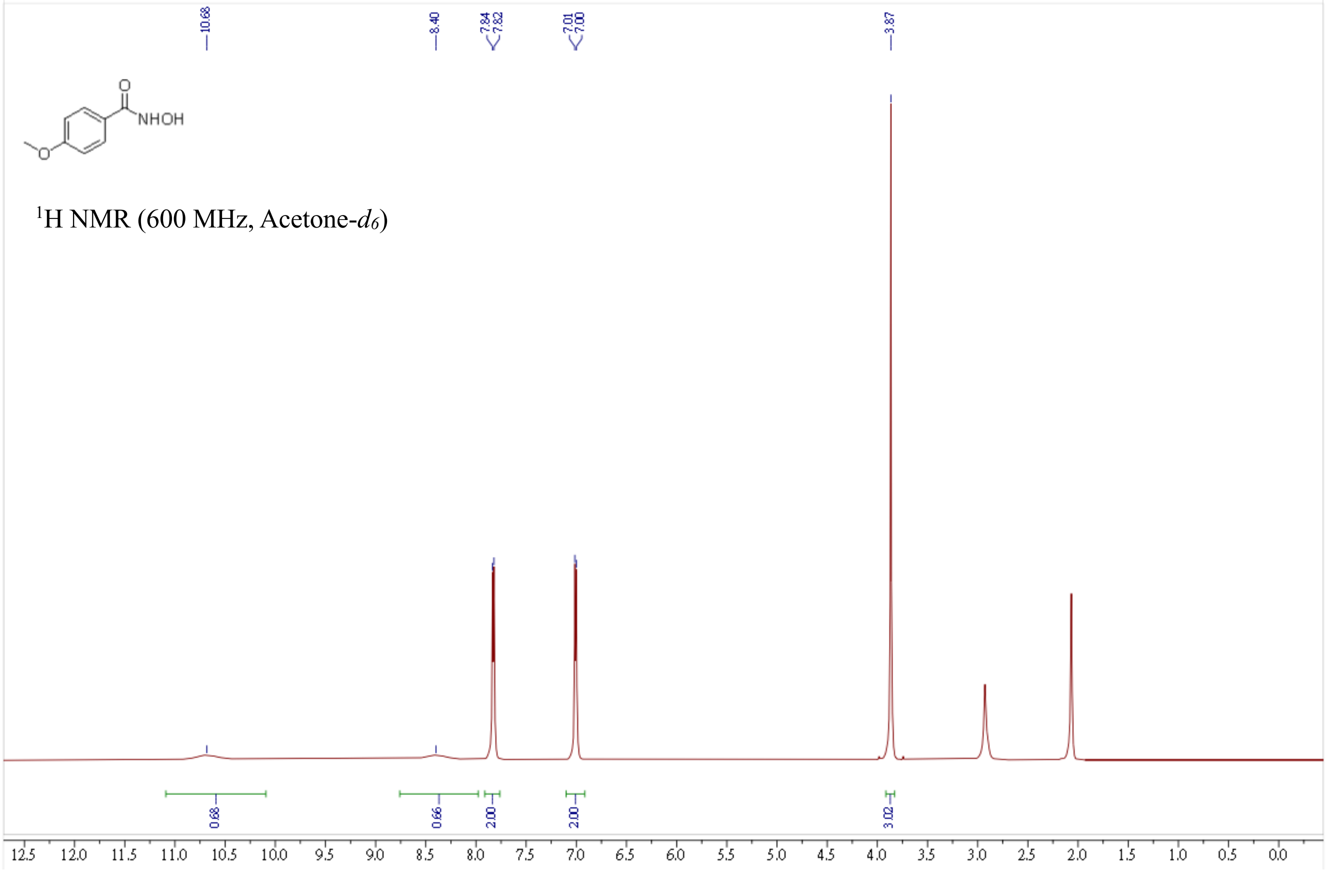

_N_-hydroxy-2-methoxybenzamide (1b)

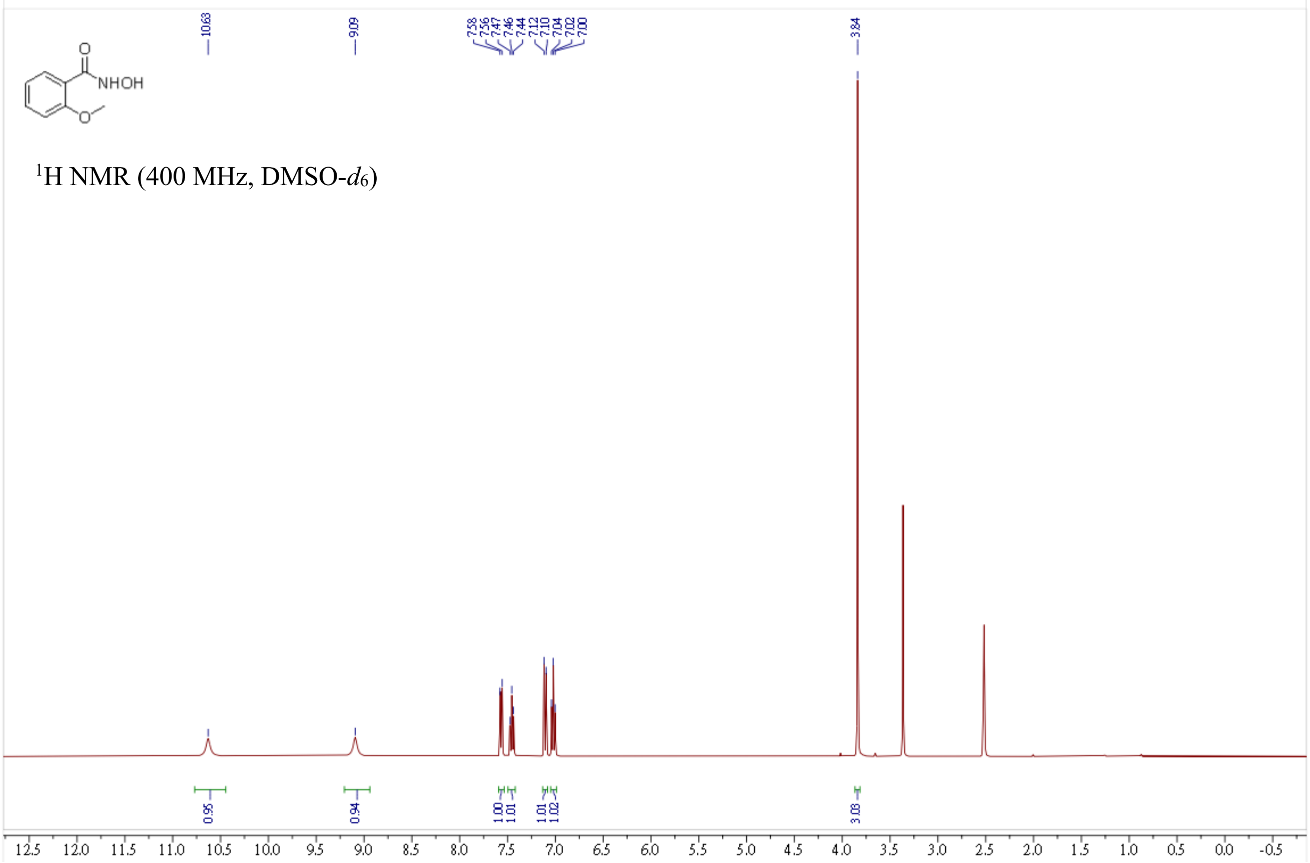

4-(*tert*-butyl)-*N*-hydroxybenzamide (1d)

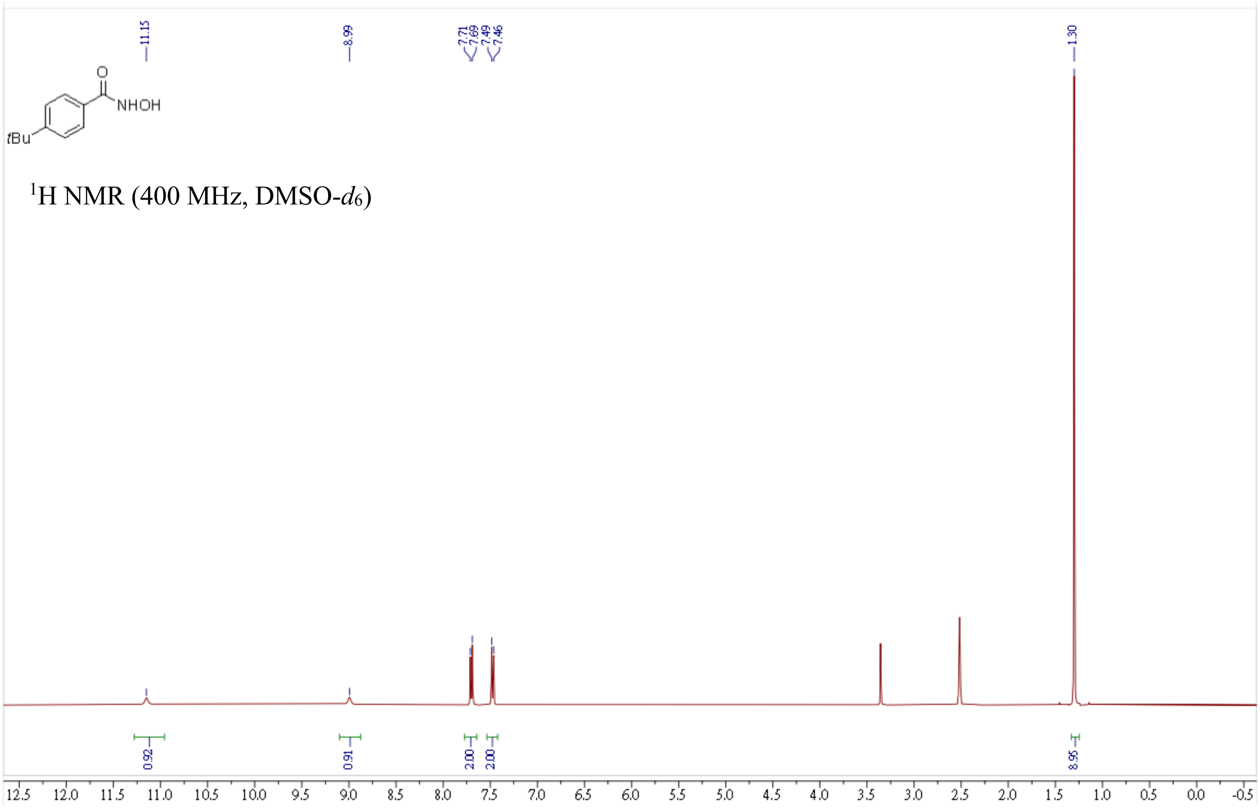

***N*-hydroxy-4-(trifluoromethoxy)benzamide (1e)**

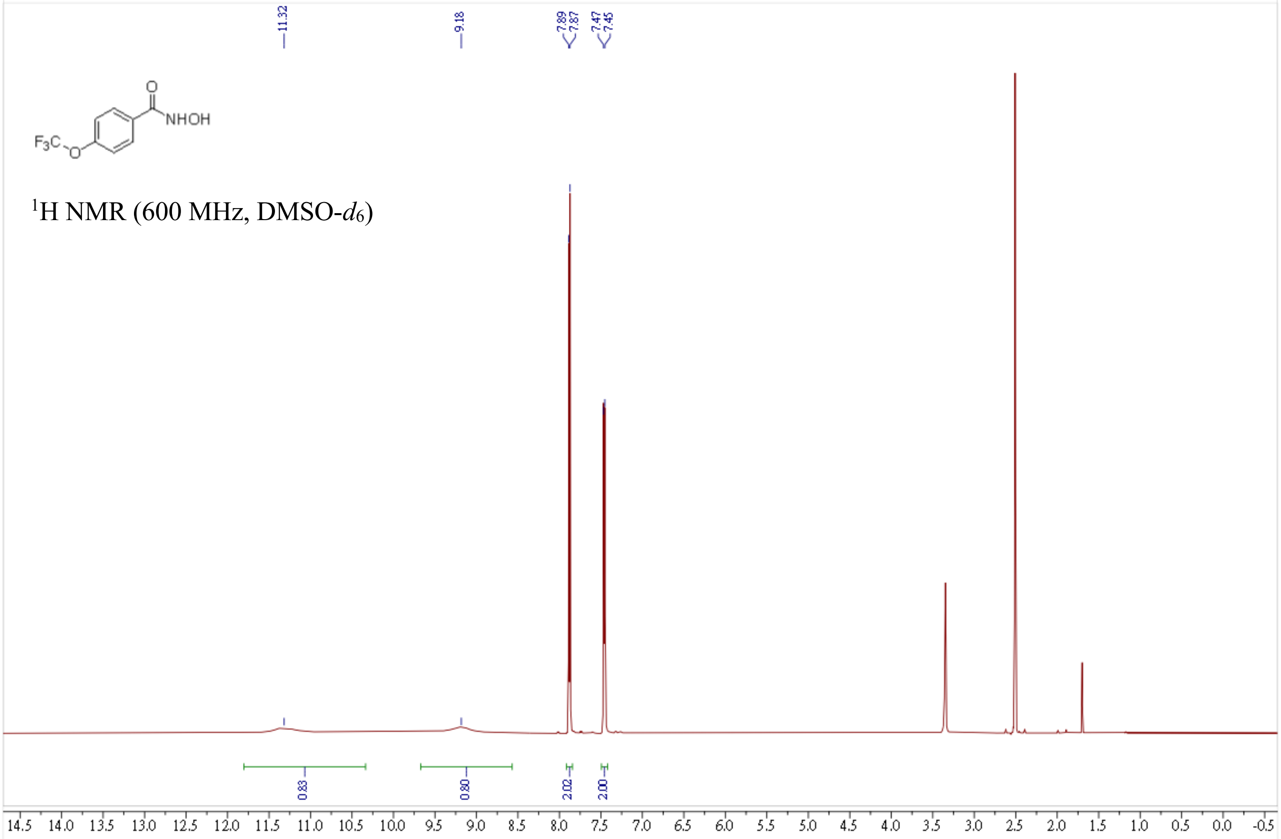

4-fluoro-*N*-hydroxybenzamide (1f)

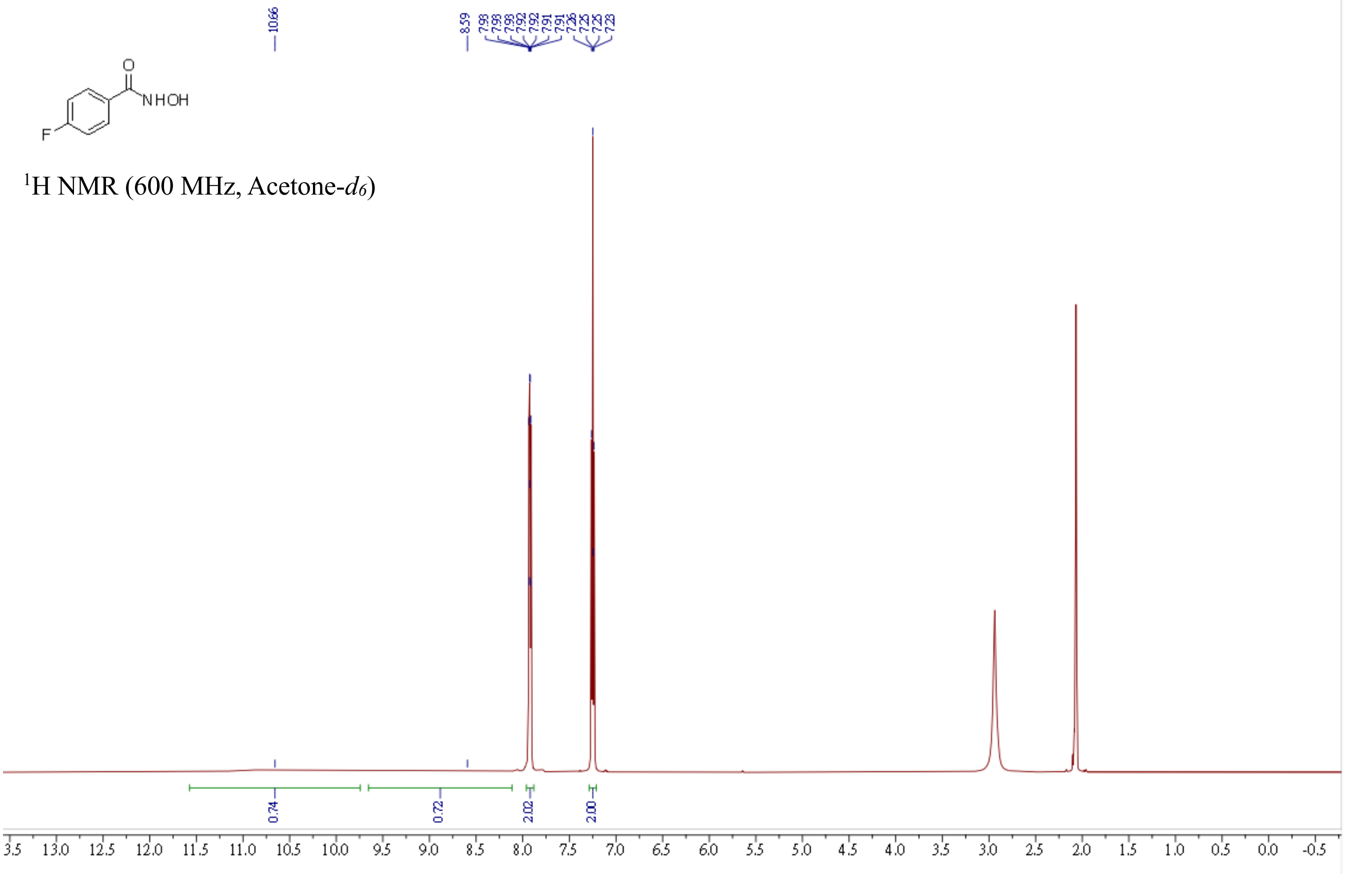

4-ethynyl-*N*-hydroxybenzamide (1g)

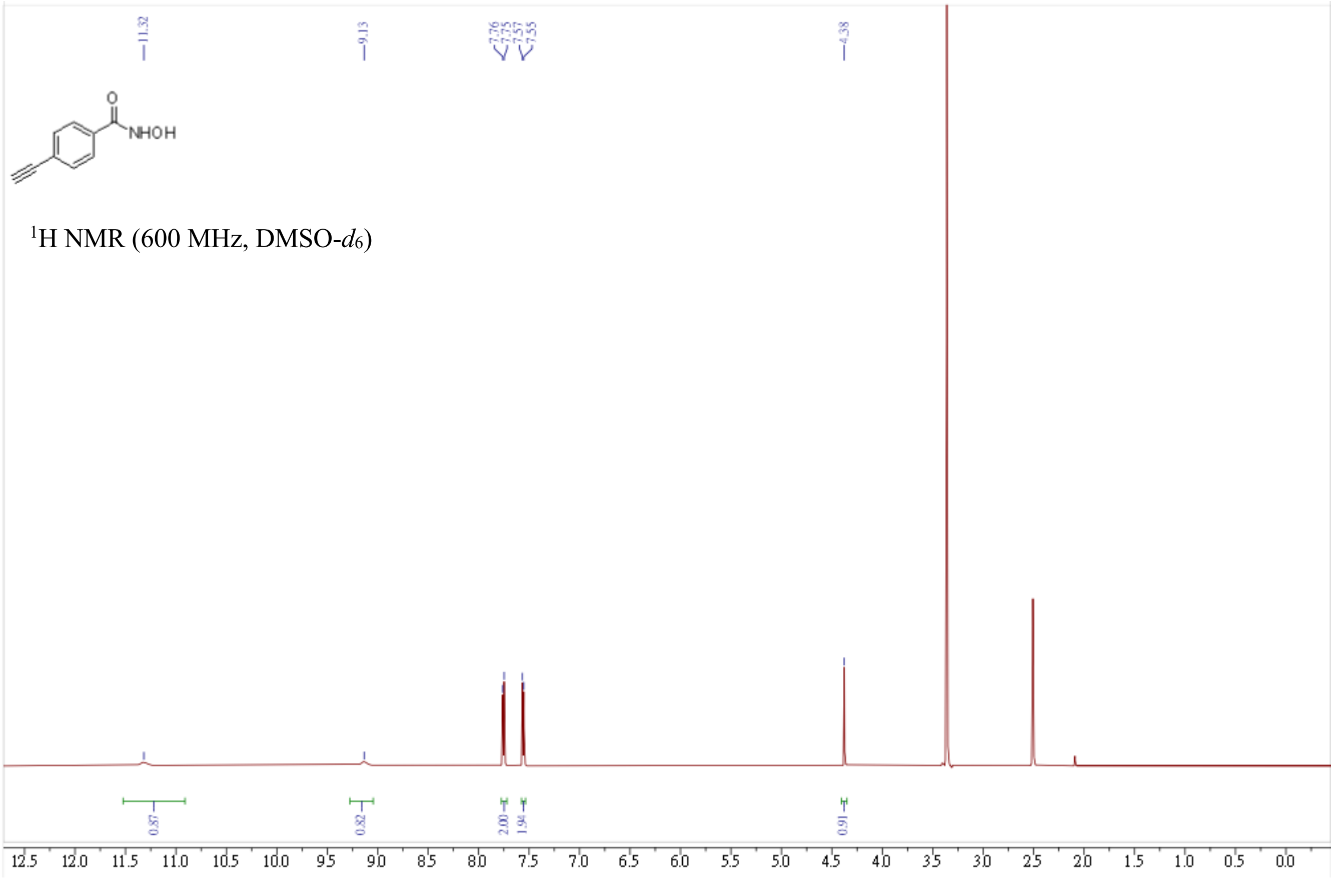

N,4-dihydroxybenzamide (1h)

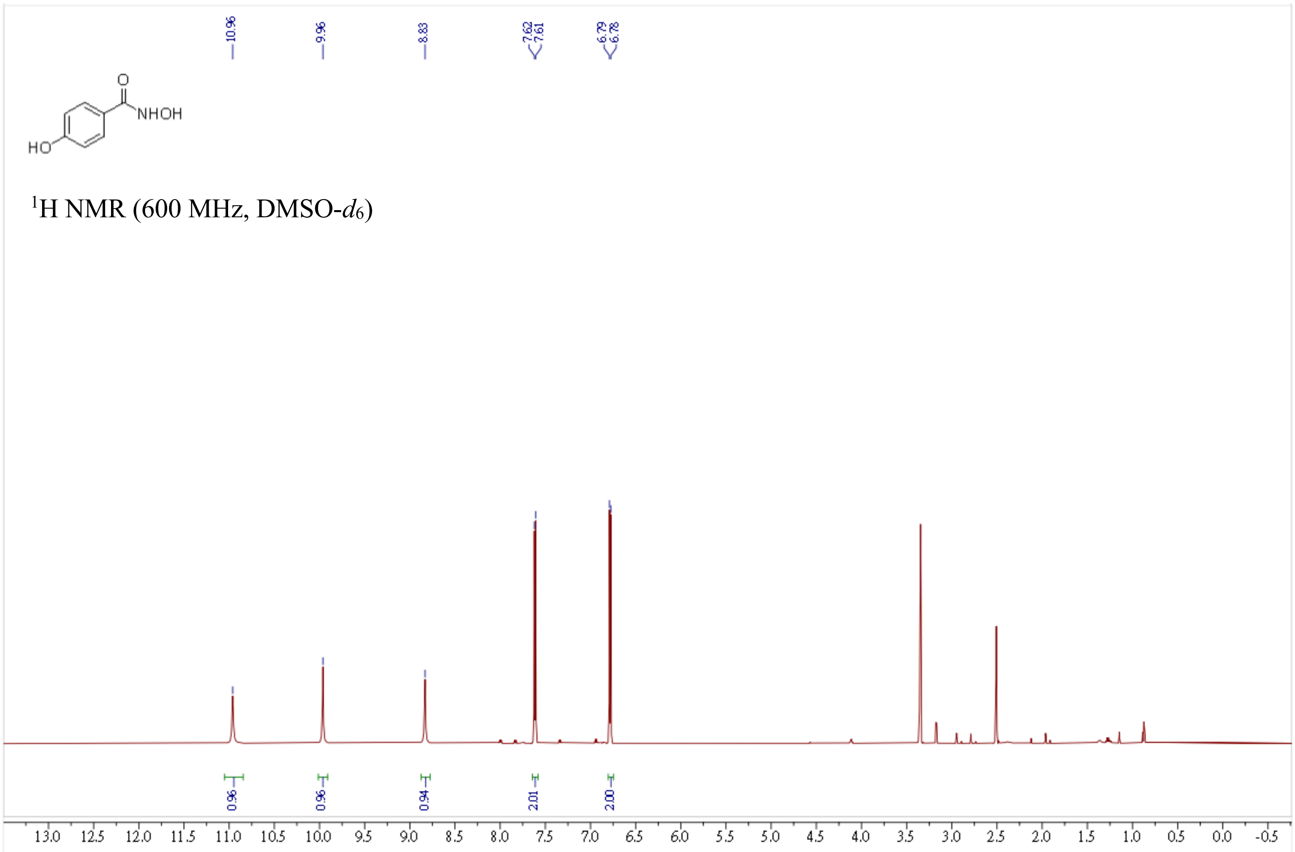

3,5-dichloro-*N*-hydroxybenzamide (1j)

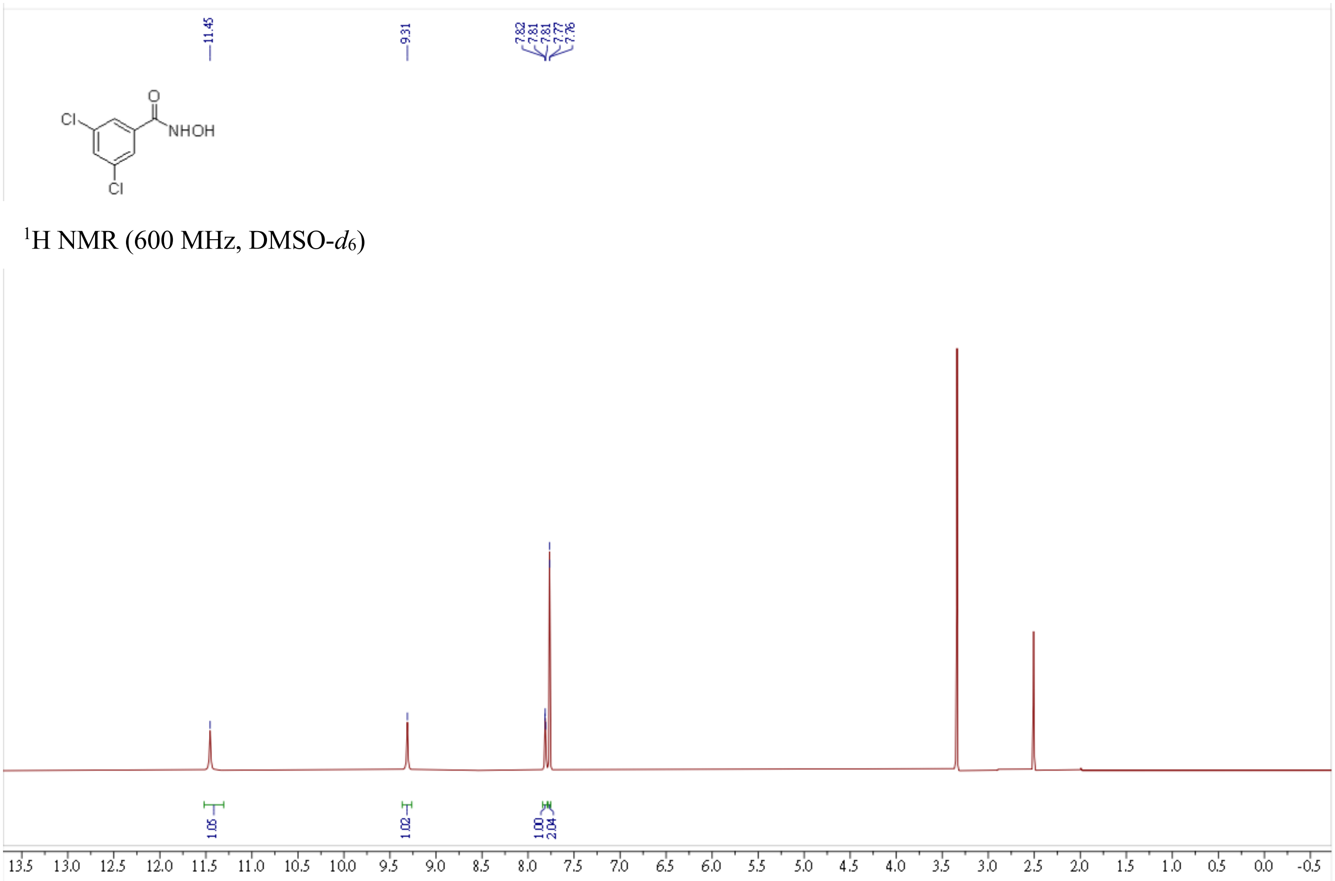

N-hydroxy-2-naphthamide (1k)

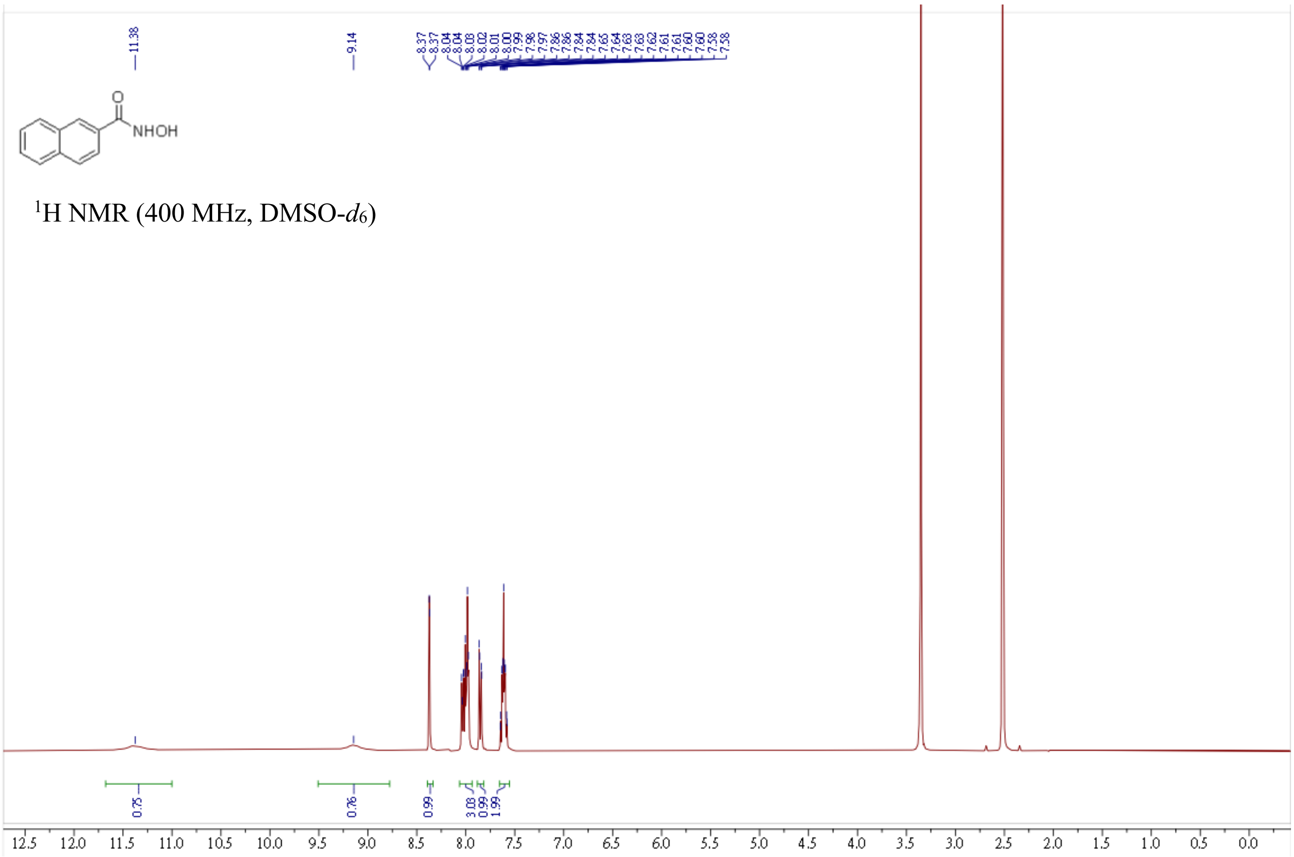

***N*-hydroxybenzofuran-5-carboxamide (1l)**

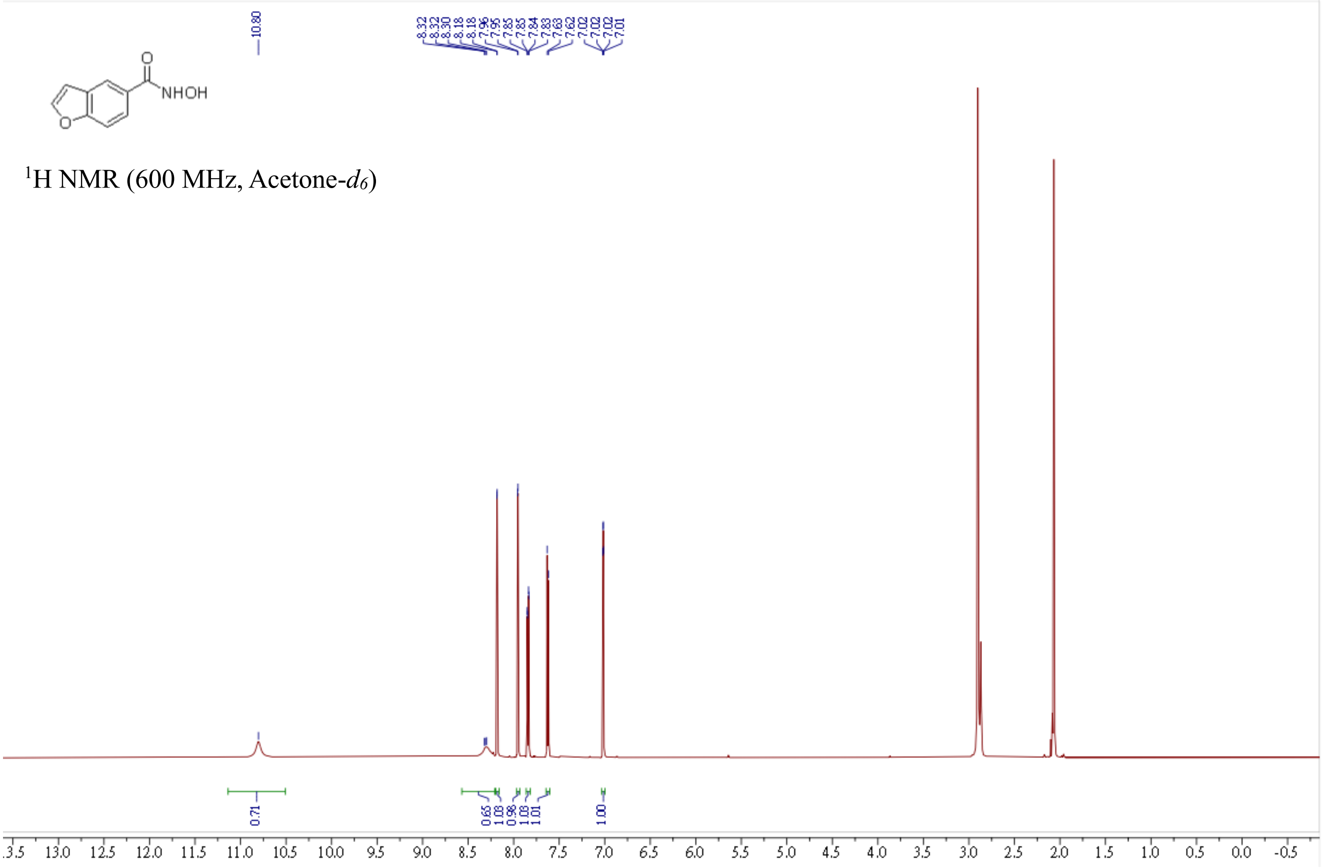

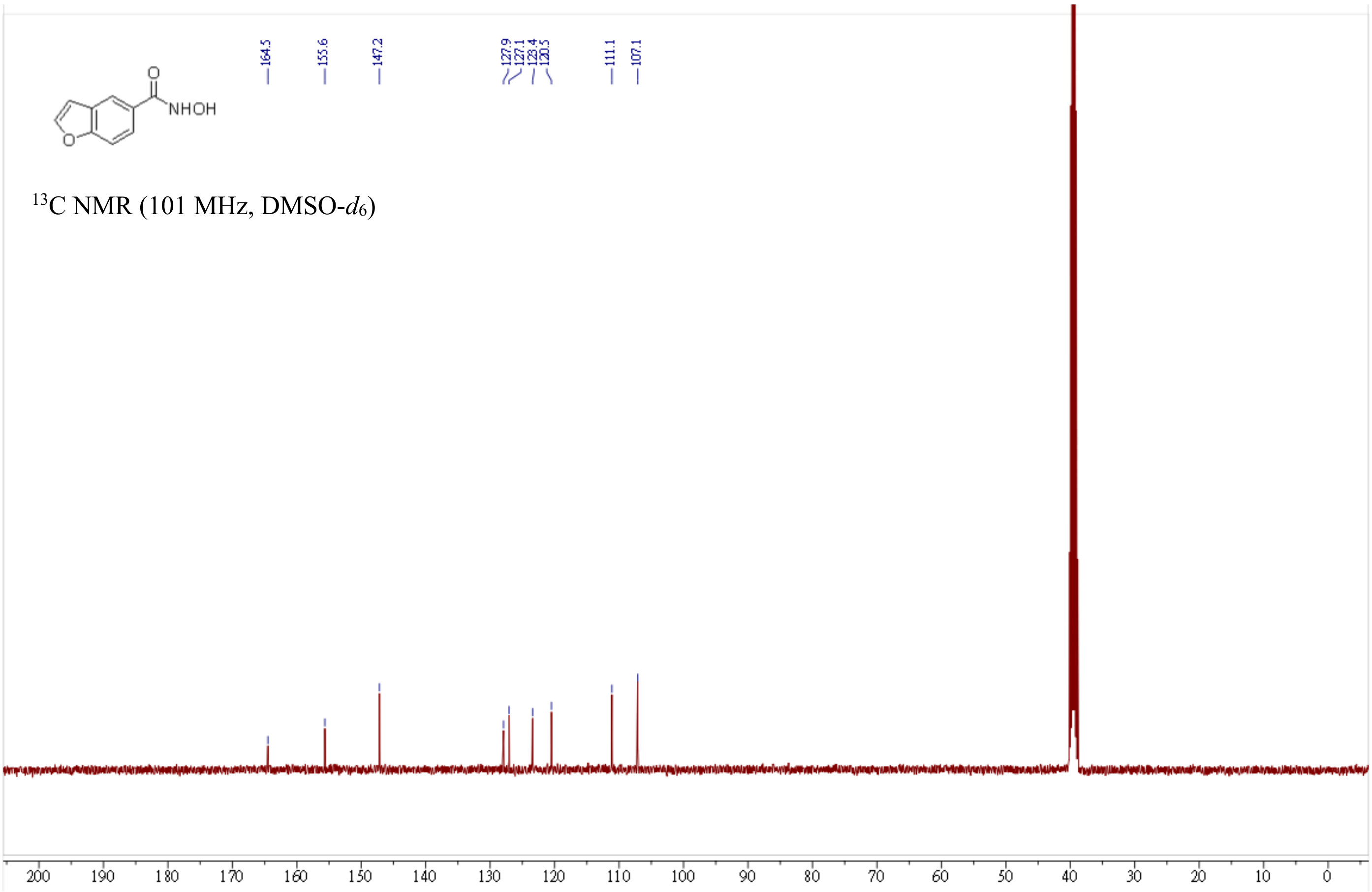

N-hydroxythiophene-2-carboxamide (1m)

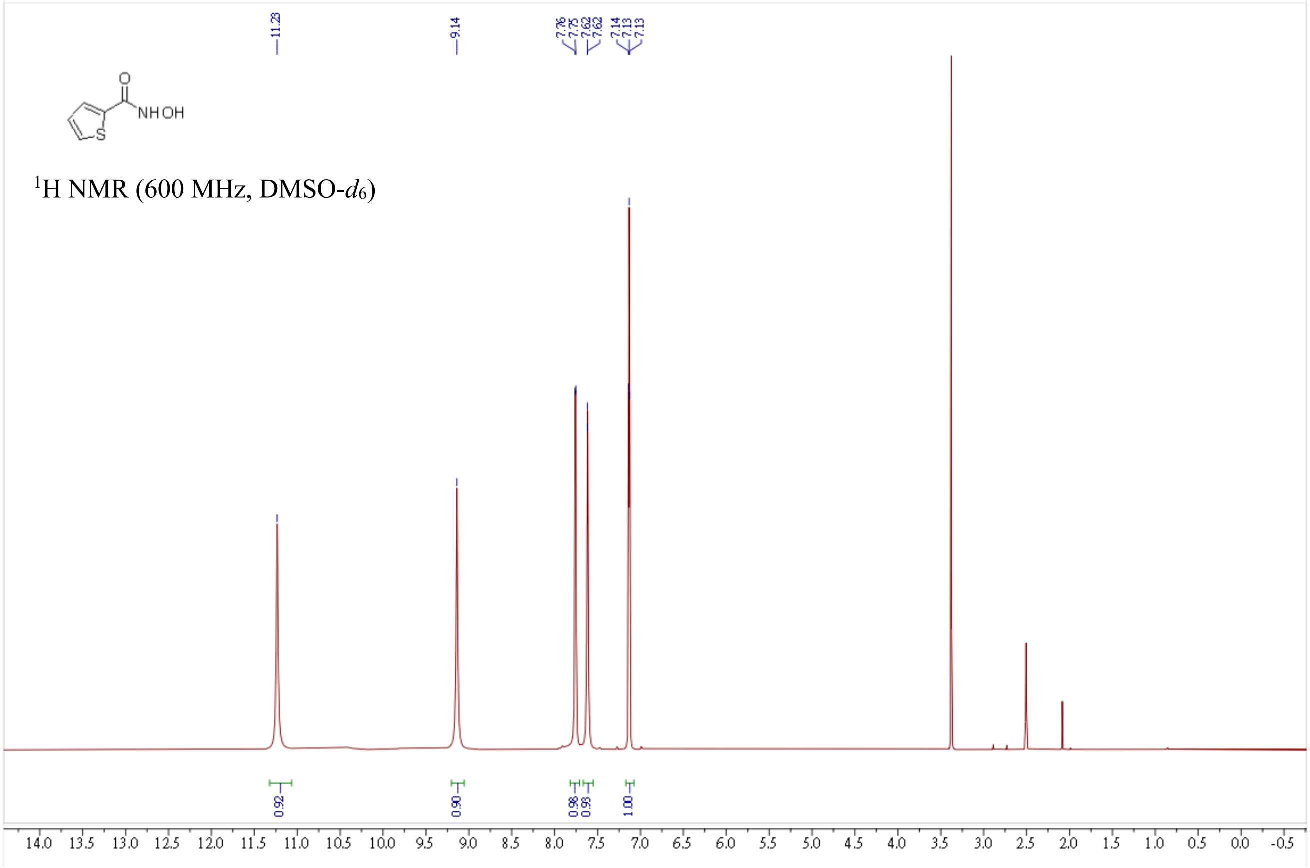

N-hydroxy-5-methylisoxazole-3-carboxamide (1n)

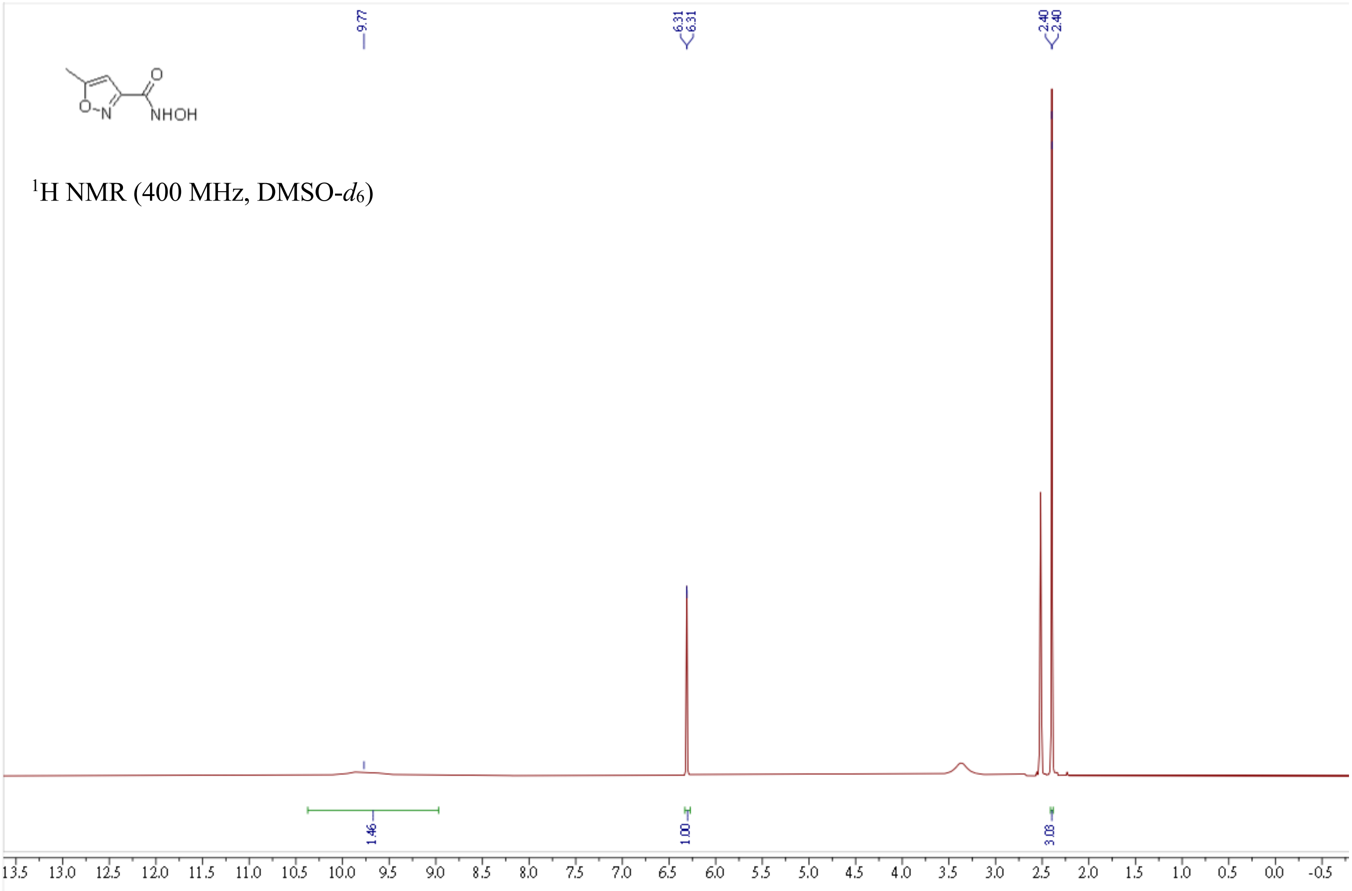

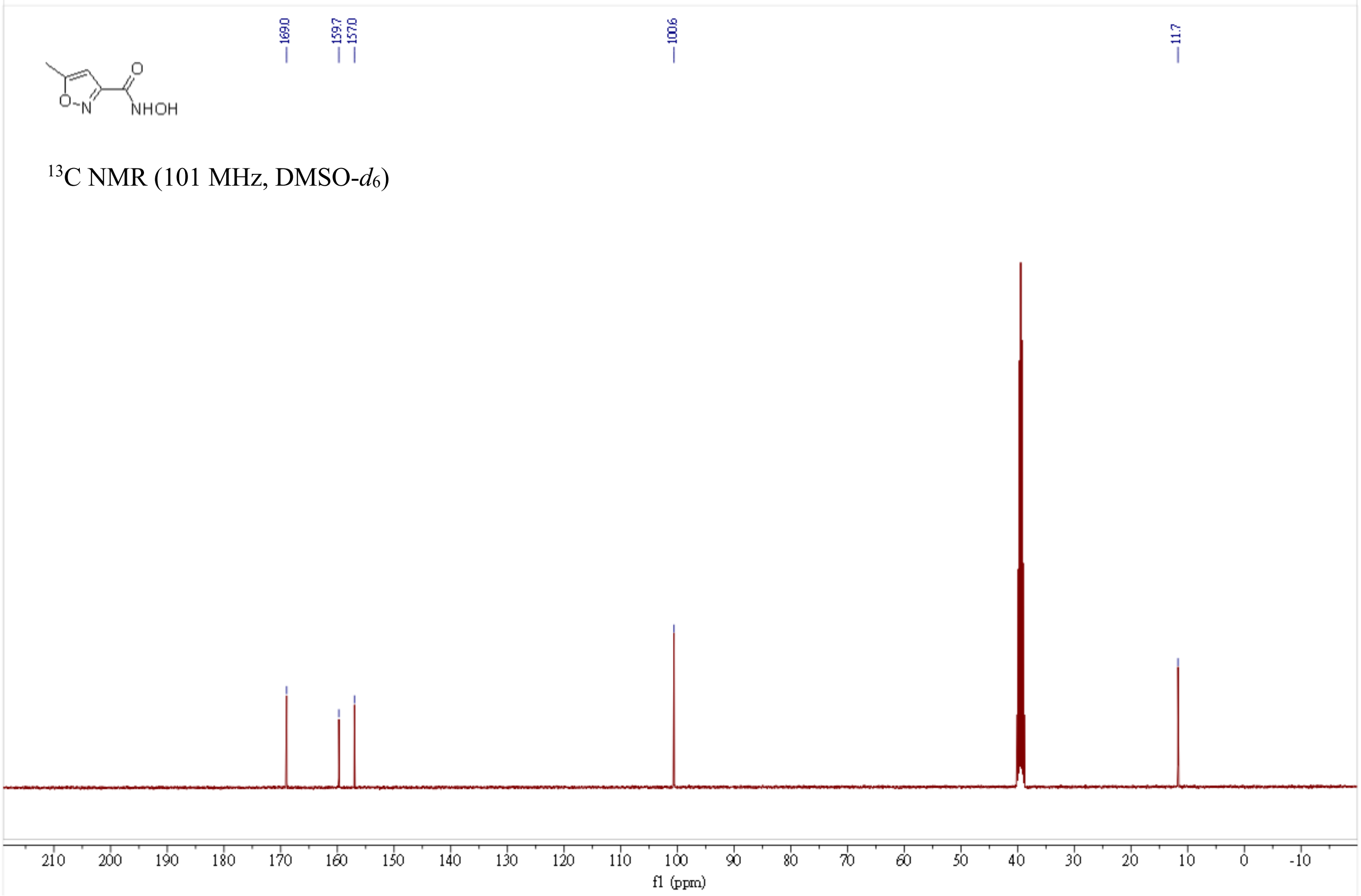

(9H-fluoren-9-yl)methyl hydroxycarbamate (1p)

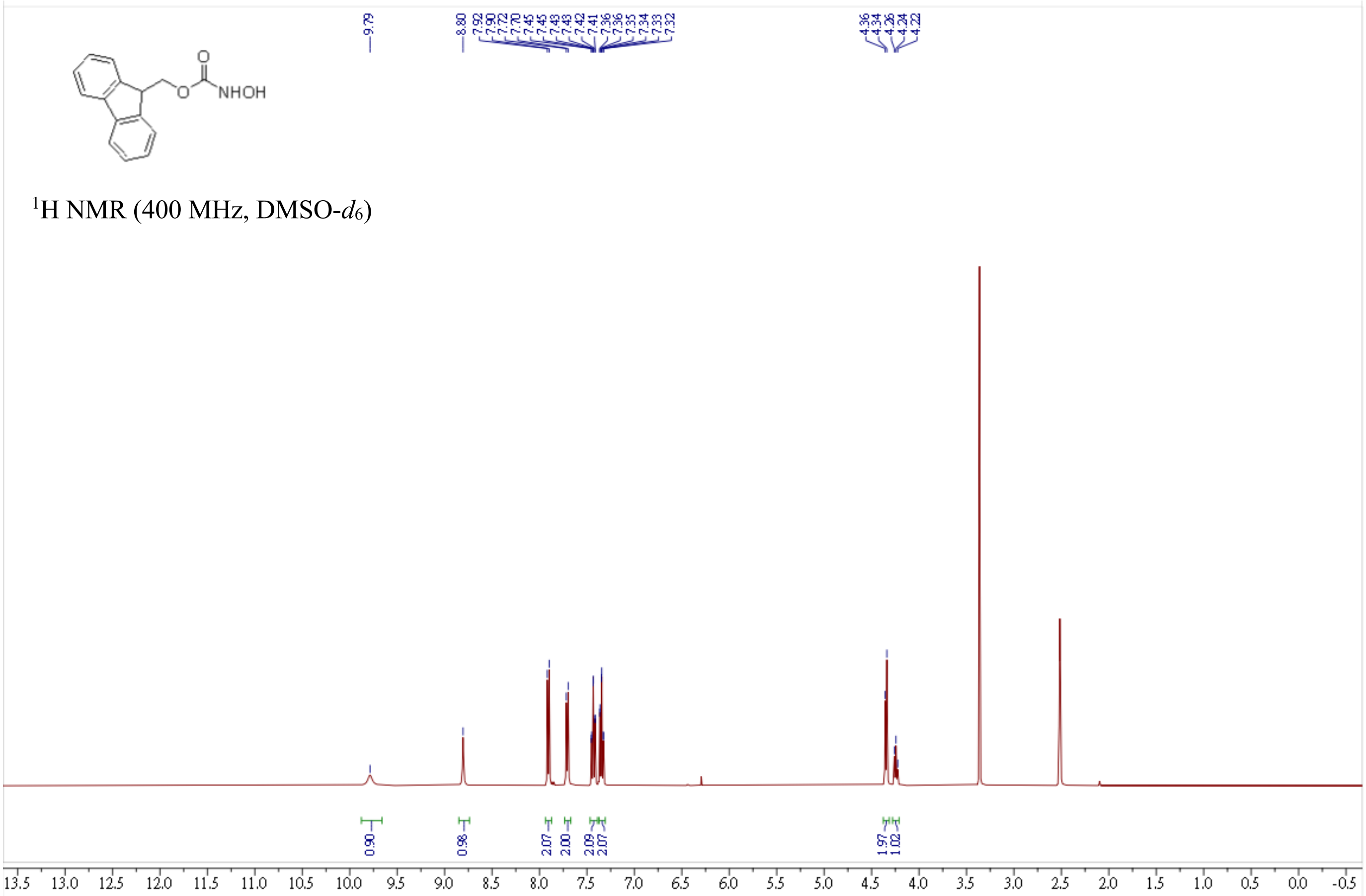

References

(1) Zhang, D.; Cai, M.; Bin, Z.; Zhang, Y.; Zhang, D.; Duan, L. Highly efficient blue thermally activated delayed fluorescent OLEDs with record-low driving voltages utilizing high triplet energy hosts with small singlet-triplet splittings. *Chem Sci* **2016**, *7* (5), 3355-3363.

(2) Zhang, D.; Cai, M.; Zhang, Y.; Zhang, D.; Duan, L. Sterically shielded blue thermally activated delayed fluorescence emitters with improved efficiency and stability. *Materials Horizons* **2016**, *3* (2), 145-151.

(3) Berger, A. L.; Donabauer, K.; König, B. Photocatalytic carbanion generation from C–H bonds–reductant free Barbier/Grignard-type reactions. *Chemical Science* **2019**, *10* (48), 10991-10996.

(4) Huang, H.-M.; Bellotti, P.; Kim, S.; Zhang, X.; Glorius, F. Catalytic multicomponent reaction involving a ketyl-type radical. *Nature Synthesis* **2022**, *1* (6), 464-474.

(5) Huang, W.; Wang, S.; Li, M.; Zhao, L.; Peng, M.; Kang, C.; Jiang, G.; Ji, F. Electrochemical N-acylation of sulfoximine with hydroxamic acid. *The Journal of Organic Chemistry* **2023**, *88* (24), 17511-17520.

(6) He, X.-L.; Ma, X.-R.; Yan, N.; Zhang, X.-W. Gold-catalyzed [4+ 1] heterocyclization of hydroxamic acid and nonactivated alkyne: a protocol to construct 5-methyl-1, 4, 2-dioxazole. *The Journal of Organic Chemistry* **2022**, *88* (1), 433-441.

(7) Wang, R.; Chen, Y.; Fei, B.; Hu, J.; Chen, J.; Luo, Y.; Xia, Y. Condition-Controlled O-Acylation and N–O Bond Reduction of Hydroximic Acids with Thioacetic Acid. *Org. Lett.* **2023**, *25* (17), 2970-2974.

(8) Suzuki, T.; Ota, Y.; Ri, M.; Bando, M.; Gotoh, A.; Itoh, Y.; Tsumoto, H.; Tatum, P. R.; Mizukami, T.; Nakagawa, H. Rapid discovery of highly potent and selective inhibitors of histone deacetylase 8 using click chemistry to generate candidate libraries. *J. Med. Chem.* **2012**, *55* (22), 9562-9575.

(9) Johann, T.; Keth, J.; Bros, M.; Frey, H. A general concept for the introduction of hydroxamic acids into polymers. *Chemical science* **2019**, *10* (29), 7009-7022.

(10) Ahmed, M.; Nencetti, S.; Mazzoni, M. R.; Porchia, F.; Antonelli, F.; Lapucci, A. Allosteric inhibition of [125I] ET-1 binding to ETA receptors by aldoxime and hydroxamic acid derivatives. *Medicinal chemistry* **2008**, *4* (4), 298-308.

(11) Harris, L.; Mee, S. P.; Furneaux, R. H.; Gainsford, G. J.; Luxenburger, A. Alkyl 4-chlorobenzoyloxycarbamates as highly effective nitrogen source reagents for the base-free, intermolecular aminohydroxylation reaction. *The Journal of organic chemistry* **2011**, *76* (2), 358-372.
